# Generalized Stomatal Optimization of Evolutionary Fitness Proxies for Predicting Plant Gas Exchange Under Drought, Heatwaves, and Elevated CO_2_

**DOI:** 10.1111/gcb.70049

**Published:** 2025-01-28

**Authors:** Aaron Potkay, Antoine Cabon, Richard L. Peters, Patrick Fonti, Gerard Sapes, Anna Sala, Artur Stefanski, Ethan Butler, Raimundo Bermudez, Rebecca Montgomery, Peter B. Reich, Xue Feng

**Affiliations:** ^1^ Department of Civil, Environmental, and Geo‐Engineering University of Minnesota Minneapolis Minnesota USA; ^2^ Saint Anthony Falls Laboratory University of Minnesota Minneapolis Minnesota USA; ^3^ Research Unit Forest Dynamics Swiss Federal Research Institute WSL Birmensdorf Switzerland; ^4^ TUM School of Life Sciences Technical University of Munich Freising Germany; ^5^ Agronomy Department University of Florida Gainesville Florida USA; ^6^ Division of Biological Sciences University of Montana Missoula Montana USA; ^7^ Department of Forest Resources University of Minnesota St. Paul Minnesota USA; ^8^ Hawkesbury Institute for the Environment Western Sydney University Penrith New South Wales Australia; ^9^ Institute for Global Change Biology, and School for the Environment and Sustainability University of Michigan Ann Arbor Michigan USA

**Keywords:** capacitance, dynamic optimization, leaf pressure–volume, photosynthesis, stomatal optimality, tissue water relations, transpiration, turgor maintenance

## Abstract

Stomata control plant water loss and photosynthetic carbon gain. Developing more generalized and accurate stomatal models is essential for earth system models and predicting responses under novel environmental conditions associated with global change. Plant optimality theories offer one promising approach, but most such theories assume that stomatal conductance maximizes photosynthetic net carbon assimilation subject to some cost or *constraint* of water. We move beyond this approach by developing a new, generalized optimality theory of stomatal conductance, optimizing any non‐foliar proxy that requires water and carbon reserves, like growth, survival, and reproduction. We overcome two prior limitations. First, we reconcile the computational efficiency of *instantaneous* optimization with a more biologically meaningful *dynamic feedback* optimization over plant lifespans. Second, we incorporate *non‐steady‐state* physics in the optimization to account for the temporal changes in the water, carbon, and energy storage within a plant and its environment that occur over the timescales that stomata act, contrary to previous theories. Our optimal stomatal conductance compares well to observations from seedlings, saplings, and mature trees from field and greenhouse experiments. Our model predicts predispositions to mortality during the 2018 European drought and captures realistic responses to environmental cues, including the partial alleviation of heat stress by evaporative cooling and the negative effect of accumulating foliar soluble carbohydrates, promoting closure under elevated CO_2_. We advance stomatal optimality theory by incorporating generalized evolutionary fitness proxies and enhance its utility without compromising its realism, offering promise for future models to more realistically and accurately predict global carbon and water fluxes.

## Graphical abstract

We develop a new generalized optimality theory of stomatal conductance that optimizes any non‐foliar fitness proxy that requires water and carbon reserves, like growth, survival, and reproduction. We consider the legacy effects of current resource use on future resource supply and plant performance (dynamic feedback optimization) and the changes in water, carbon, and energy reserves within a plant and its environment (non‐steady‐state physics). Our optimal stomatal conductance compares well to observations and captures realistic responses to drought, heatwaves, and elevated atmospheric CO_2_ concentrations. Our theory offers a promising pathway to more realistically predict carbon and water fluxes under global change.

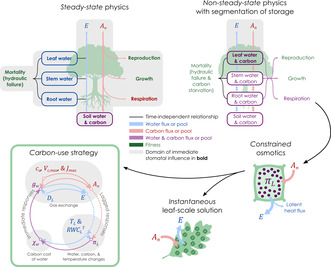

## 1 Introduction

Stomata are pores on leaves that open and close to regulate the loss of water and photosynthetic influx of CO_2_. They play a central role in regulating global water and carbon cycles. The conductance of water and CO_2_ through these pores increases with light and declines with vapor pressure deficit (VPD), elevated CO_2_ concentrations, and hydraulic stress (Ball, Woodrow, and Berry 1987; Manzoni et al. 2011). Classically, the effects of stomata on the water and carbon cycles have been predicted in ecosystem models using empirical relationships (Ball, Woodrow, and Berry 1987; Leuning 1995). Recently, plant optimality approaches have been called for to improve ecosystem models by reducing the number of empirical parameters that cannot be independently measured and by improving prediction, especially under novel environmental conditions (Franklin et al. 2020; Harrison et al. 2021). Indeed, optimality approaches for predicting stomatal conductance are gradually replacing empirical relationships in large‐scale ecosystem models (De Kauwe et al. 2015a; Eller et al. 2020; Sabot et al. 2020; Wang and Frankenberg, 2022).

Plant optimality models predict plant traits as the values that maximize an *objective* or mathematical proxy for eco‐evolutionary fitness (Franklin et al. 2020) since natural selection maximizes fitness. Fitness proxy refers to a metric that represents and is positively correlated with fitness, such as photosynthetic carbon gain, growth, survival, and reproduction over lifetimes. Stomatal optimality models suggest that stomata open and close to balance a tradeoff between a profit (typically photosynthetic carbon gain) and some combination of *costs* and *constraints* (typically some metric of hydraulic stress) (Box 1). In the past decade, many stomatal optimality models have been introduced to predict transpiration and photosynthetic assimilation rates (Medlyn et al. 2011; Wang et al. 2020; Sabot et al. 2022a) as well as productivity and mortality (Sperry et al. 2019; De Kauwe et al. 2020; Venturas et al. 2021; Quetin et al. 2023). Many of them predict stomatal conductance comparably well (Wang et al. 2020; Bassiouni and Vico 2021; Sabot et al. 2022a) and predict similar stomatal responses to environmental cues as one another (Dewar et al. 2018; Wang et al. 2020; Potkay and Feng 2023a). However, their ability to capture observed trends through calibration has obscured important differences in their optimization *objectives*, timescales, and various other assumptions. These theoretical differences can ultimately undermine our ability to predict future ecosystem dynamics and make sense of biological behaviors (Buckley 2023). The validity of optimality models and their predictions, especially under novel environmental conditions, rest on the appropriateness of their rarely scrutinized underlying theories and assumptions (Parker and Maynard Smith 1990; Berninger, Mäkelä, and Hari 1996). Commonly, new stomata optimality models are developed by gradually modifying older models. These modifications have often taken the form of incorporating more physiological mechanisms into their fitness proxy, such as whole shoot respiration (Prentice et al. 2014; Potkay and Feng 2023a, 2023b), xylem embolism (Wolf, Anderegg, and Pacala 2016; Sperry et al. 2017; Eller et al. 2020), non‐stomatal limitations to photosynthesis (Dewar et al. 2018), phloem transport (Hölttä et al. 2017; Dewar, Hölttä, and Salmon 2022), and carbohydrate storage and growth (Potkay and Feng 2023a, 2023b). However, there is little discourse about what mechanisms are relevant to the stomatal optimization problem, and whether they may unnecessarily burden model parameterization. For example, recent studies suggest that stomatal optimality models were not improved by the inclusion of xylem hydraulic conductance and embolism in their *objectives* (Bassiouni and Vico 2021; Hawkins et al. 2022), instead adding model uncertainty. Most importantly, recent advances in stomatal optimization models have refrained from deliberating on the choice of proxy for evolutionary fitness. Consequently, we have yet to theoretically and mathematically formulate the stomatal optimization problem with a general proxy of evolutionary fitness in the optimization *objective*.

BOX 1 ***Instantaneous* versus *dynamic feedback* optimization and *non‐steady‐state* versus *steady‐state* physics.**

**Table:** 

**Stomatal optimization approaches** Stomatal optimization (or optimality) approaches hypothesize that stomata open and close to maximize evolutionary fitness. Different stomata theories postulate different mathematical *objectives* (O) to represent fitness as well as different timescales over which *objectives* are maximized (either *instantaneous* or *dynamic feedback*). Optimization often involves defining Lagrange multipliers, which are nonphysical representations of the “shadow cost” of resource use necessary to solve the optimization problem.	
***Instantaneous*** Stomatal conductance ($g_{w}$) is optimized to maximize an *objective* (O) without consideration of time or resources: $\max_{g_{w}}\left[O\right]$ The solution to an *instantaneous* stomatal optimization problem is the $g_{w}$ that satisfies $\frac{dO}{dg_{w}}=0$. Lagrange multipliers are not necessarily present. When present, they are constant by definition.	***Dynamic feedback*** Stomatal conductance ($g_{w}$) is optimized to maximize an *objective* (O) over time with *constraints* on how the relevant resources ($X_{i}$ for *i*th resource) vary dynamically to describe resource conservation: $\max_{g_{w}}\left[\int O\;\mathrm{dt}\right]$ *Dynamic feedback* optimizations explicitly consider how current strategies will impact the future availability of resources and thus future trajectories of the *objective*. The solution to a *dynamic feedback* stomatal optimization problem requires following the calculus of variations. Through the calculus of variations, Lagrange multipliers ($\eta_{i}$) emerge for each resource ($X_{i}$). Their values vary dynamically according to the availability of the resources. Solving the calculus of variations requires defining a Hamiltonian (H) (or alternatively an augmented Lagrangian, which we use in our Supporting Information) in terms of the *objective* (O), the Lagrange multipliers ($\eta_{i}$), and the rate of change on the *constrained* resources (${\dot{X}}_{i}=\frac{dX_{i}}{\mathrm{dt}}$): $H=O+\sum_{i}\eta_{i}{\dot{X}}_{i}$, Hence, the solution to a *dynamic feedback* optimization problem will depend on $\frac{dX_{i}}{\mathrm{dt}}$ terms.
***Non‐steady‐state* vs. *steady‐state* physics**	
***Non‐steady‐state* physics** A mathematical description of physics in which the capacitances or storage of physical quantities like mass and energy are included. For plants, these physical quantities include tissue water contents, water potentials, osmolyte and carbohydrate concentrations (S2 in Supporting Information), temperature, and soil water content. *Non‐steady‐state* considers how these physical quantities change in time, often under varying environmental conditions. For example, the temporal change in a physical quantity, *x*, is generally given by an expression of the form, $c\frac{\mathrm{dx}}{\mathrm{dt}}=i-o$, where *t* is time, $\frac{\mathrm{dx}}{\mathrm{dt}}$ is the rate at which the physical quantity changes, *c* is the capacitance that represents the effects of storage on how quickly *x* can change, and *i* and *o* are input and output terms to the system.	***Steady‐state* physics** A mathematical abstraction that simplifies physics by assuming that physical quantities have ceased changing as if an indefinite amount of time had passed under static environmental conditions. *Steady‐state* physics is mathematically equivalent to *non‐steady‐state physics* with zero capacitance, storage, or legacy effects. In *steady‐state*, the physical quantity, *x*, is assumed to no longer change ($\frac{\mathrm{dx}}{\mathrm{dt}}=0$). Hence, inputs and outputs to the system are equal (i=o).

### 1.1 Current Limitations of Stomatal Optimality Models

Optimality models are premised upon natural selection and fitness maximization. Thus, their validity should depend on their choice of fitness proxy and how well their mathematical *objective* represents fitness. The ideal fitness proxy should be reproductive success over a plant's lifetime (Taylor et al. 1974; Mäkelä et al. 2002). However, the choice of proxy for evolutionary fitness is limited by our ability to accurately represent such proxy mathematically. Reproduction‐maximization has been restricted to predicting plant traits that are presumed static within plant life stages (e.g., carbon allocation fractions to different organs; Dybzinski et al. 2011; Farrior et al. 2013) or to dynamic traits of plants with annual life cycles (King and Roughgarden 1982a, 1982b; Chiariello and Roughgarden 1984). A less direct but useful fitness proxy is growth integrated over time (i.e., biomass) since it directly reflects plants' abilities to compete for resources (light, water, and nutrients) (King 1990; Franklin 2007; Towers et al. 2024) and reproduction (Obeso 2002). By integrating either reproductive success or growth rates over time, these proxies implicitly account for survival because plants must survive now to reproduce and grow in the future. However, due to our limited ability to represent the physiological controls on reproduction and growth, stomatal optimality models have overwhelmingly used photosynthetic carbon assimilation ($A_{n}$, mol∙m^−2^∙s^−1^; Cowan and Farquhar 1977; Dewar et al. 2018) or the difference between assimilation and some conjectured *hydraulic cost* (Θ, mol∙m^−2^∙s^−1^; e.g., soil–plant conductance loss; Wolf, Anderegg, and Pacala 2016; Sperry et al. 2017) as the default fitness proxy. The choice of photosynthesis as a fitness proxy is often justified by assuming photosynthesis to be coupled with growth (Givnish and Vermeij 1976; Cowan 1982; Friend 1995). In reality, photosynthesis and growth are decoupled over the short timescales that stomata act (Fatichi, Leuzinger, and Körner 2014; Cabon et al. 2022; Oswald and Aubrey 2023). Only in our recent work has growth been explicitly introduced as a fitness proxy in stomatal optimality models (Potkay and Feng 2023a, 2023b).

Trusting stomatal optimality models' predictions under novel environmental conditions, especially those related to global change, requires incorporating more realistic fitness proxies like growth and reproduction into the optimization problem. To do so, stomatal optimality models must overcome two theoretical limitations. They must be premised upon (i) a timescale of optimization that encompasses plant lifespans and (ii) the impacts that stomata have on the plant water, carbon, and energy storage that control fitness proxies.

To overcome the first limitation, we propose a new method to integrate the realism of *dynamic feedback* optimization with the simplicity of *instantaneous* optimization. To elaborate, the earliest stomatal optimality models were framed as *constrained* optimization problems (Box 1; Cowan and Farquhar 1977; Cowan 1982, 1986; Mäkelä, Berninger, and Hari 1996), in which the *objective* is maximized over a period of time. These problems, classified by Feng et al. (2022) as *dynamic feedback* optimization, incorporate *constraints* describing resource conservation and thus explicitly consider how current resource‐use will impact the future availability of resources. However, *instantaneous* optimization models have risen in popularity in response to the demand for incorporating plant hydraulic parameters (Wolf, Anderegg, and Pacala 2016; Sperry et al. 2017; Eller et al. 2020; Joshi et al. 2022) and due to their simplicity. These *instantaneous* optimization models describe stomatal behavior by maximizing an *objective* at a single instance in time without considering plant lifetimes and the future impacts of such behavior. *Instantaneous* optimization models are less computationally demanding and easier to use than *dynamic feedback* optimization models. For this reason, they are preferentially integrated into ecosystem models (Eller et al. 2020; Sabot et al. 2020; Wang and Frankenberg 2022). However, *instantaneous* optimization models are suboptimal in terms of maximizing their *objectives* over time in comparison to *dynamic feedback* optimization models because they cannot account for the legacy effects of current resource use (Feng et al. 2022; Buckley, Frehner, and Bailey 2023). Such legacy effects include depletion of a limited resource (e.g., water; Cowan and Farquhar 1977; Cowan 1982, 1986; Mäkelä, Berninger, and Hari 1996; Manzoni et al. 2013a), damage to the plant hydraulic transport system (Mrad et al. 2019; Lu et al. 2020), and temperature modulation through transpiration. Current *instantaneous* optimization models are incapable of describing a class of stomata behaviors involving current–future tradeoffs, including aggressive water‐use strategies that plants adopt under high temperatures to reduce their risk of heat‐induced foliar mortality through evaporative cooling (Blonder et al. 2023; Mills, Bartlett, and Buckley 2024). These limitations of *instantaneous* models are fundamental to their framework since *instantaneous* models inherently lack a sense of time and the future welfare of the plant. Hence, there is a need for *dynamic feedback* optimization models for stomatal conductance that are as computationally efficient and accessible as their *instantaneous* counterparts.

**FIGURE 1:**
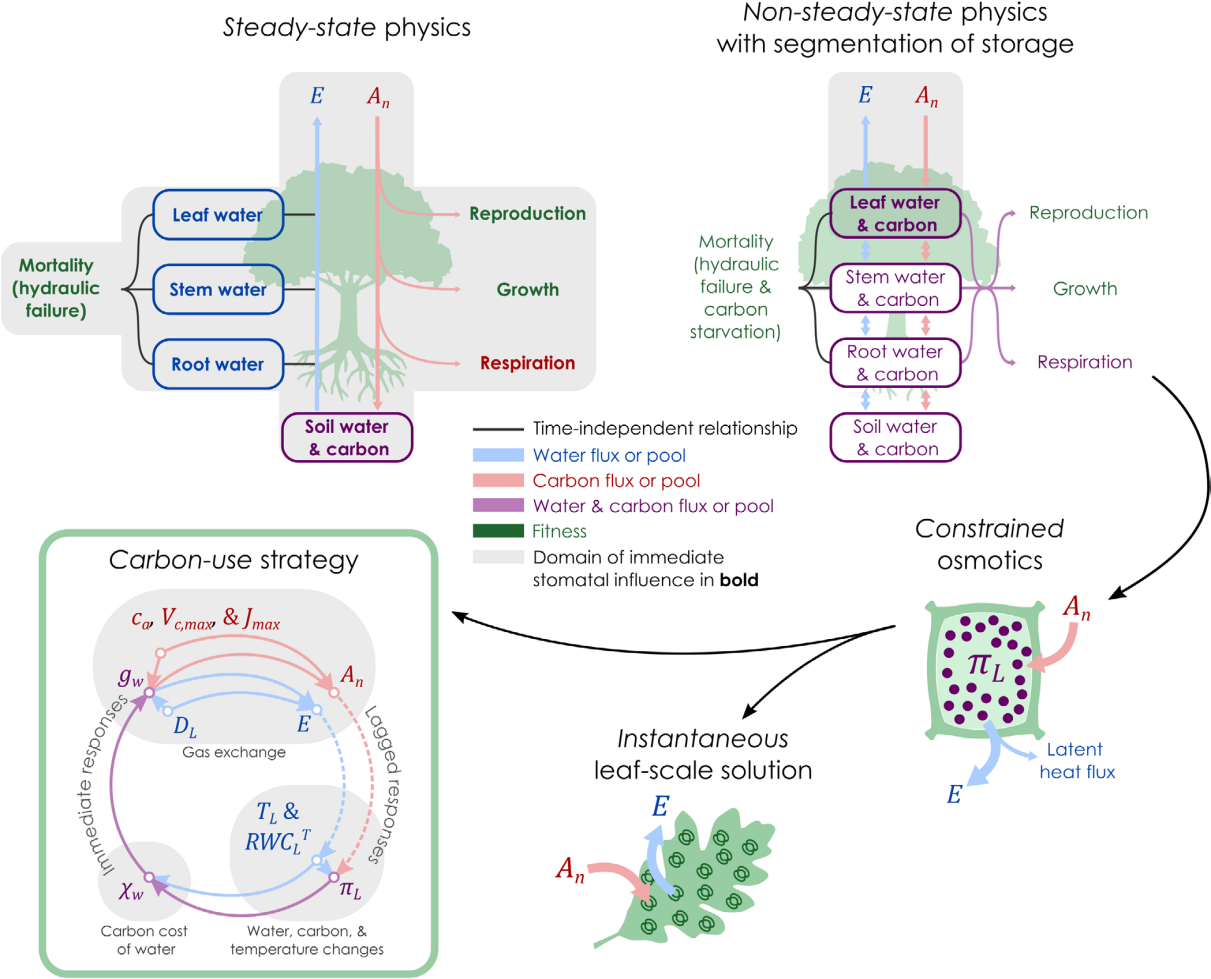
Schematic representation of our model for predicting optimal stomatal conductance ($g_{w}$). At the top, we show differences between the modeling frameworks of traditional models with *steady‐state* physics and our more realistic model with *non‐steady‐state* physics. In the traditional modeling framework with *steady‐state* physics, there is no change in water and carbon storage of plant tissues, so transpiration (E) pulls water directly from soil water storage, and photosynthetic net carbon assimilation ($A_{n}$) is immediately partitioned between reproduction, growth, respiration, and exudation to soils without buffering from carbon storage. Although there is no temporal change in tissue water storage under *steady‐state* physics by definition, there is a single hydration status for each tissue that mathematically varies with the transpiration rate since the transpiration is proportional to the sap flux rate under *steady‐state* physics, since a given sap flux rate is associated with a gradient in water potentials (i.e., Darcy's law), and since water potentials and tissue water contents are related independently of time (i.e., PV theory). Mortality by hydraulic failure may be predicted from these *steady‐state* water potentials and tissue water contents. Hence, according to the traditional modeling framework, stomatal action immediately influences the whole plant and its fitness (reproduction, growth, and survival) (gray‐shaded areas). In our new framework with *non‐steady‐state* physics, transpiration (E) pulls water directly from leaf water storage, meaning that transpiration immediately affects the rate of change of the leaf water potential and leaf water content, but does not immediately affect the leaf water potential or leaf water content per se or the water storage of the rest of the plant that is segmented from the leaf. Similarly, net carbon assimilation ($A_{n}$) immediately affects the rate of change of leaf carbon stores, but not the leaf carbon per se or the carbon storage of the rest of other tissues. Hence, the domain of immediate stomatal influence is restricted to the leaf in the new modeling framework (gray‐shaded area) without immediately influencing fitness. At the cell scale, $A_{n}$ adds solutes, while E concentrates the solutes and cools the leaf, thereby immediately affecting the leaf mesophyll osmotic potential ($\pi_{L}$), turgor pressure, and total water potential, which when considered as single *constraints* on the *dynamic* optimization problem, lead to an effectively *instantaneous* solution for the optimal stomatal behavior at the leaf scale. E cools and dehydrates leaves, lowering their temperature ($T_{L}^{K}$) and total relative water content (${\mathrm{RWC}}_{L}^{T}$), thereby affecting $\pi_{L}$. Simultaneously, $A_{n}$ affects $\pi_{L}$ by adding solutes. $\pi_{L}$, $T_{L}^{K}$, and ${\mathrm{RWC}}_{L}^{T}$ define the *marginal carbon cost of water* ($\chi_{w}$). $g_{w}$ is defined by $\chi_{w}$, the leaf‐to‐air vapor pressure deficit ($D_{L}$), and the leaf photosynthetic capacities ($V_{c,\max}$ & $J_{\max}$). E depends on $g_{w}$ and $D_{L}$, while $A_{n}$ depends on $g_{w},V_{c,\max}$, and $J_{\max}$. Solid arrows indicate immediate effects, while dashed arrows indicate lagged effects.

Overcoming the second limitation requires that the stomatal optimality model adopt *non‐steady‐state* physics to incorporate the changes in plant water, carbon, and temperature that occur over the timescales that stomata operate (Box 1; S2 in Supporting Information). Instead, many stomata models currently use *steady‐state* physics—an abstraction of *non‐steady‐state* physics that simplifies the mathematics by ignoring temporal changes in mass and energy reserves—to describe water, carbon, and temperature within plants (Box 1; Figure 1). Hence, past stomata models typically do not consider the effects of plant water, carbon, and energy storage on stomatal conductance, including non‐optimality models (Jones and Sutherland 1991; Sperry and Love 2015; Sperry et al. 2016; Carminati and Javaux 2020), *instantaneous* optimizations (Sperry et al. 2017; Eller et al. 2020; Joshi et al. 2022), and *dynamic feedback* optimizations (Cowan 1982, 1986; Mäkelä, Berninger, and Hari 1996; Manzoni et al. 2013a) with few exceptions (Bartlett, Detto, and Pacala 2019; Potkay and Feng 2023a, 2023b). A *steady‐state* interpretation of plant hydraulics is mathematically necessary for past stomatal optimality models because it is only under *steady‐state* that their *objectives* or *constraints* depend on stomatal conductance, including losses of soil water (Cowan 1982, 1986; Mäkelä, Berninger, and Hari 1996), soil–plant or plant conductance (Sperry et al. 2017; Eller et al. 2020), and leaf water potential (Wolf, Anderegg, and Pacala 2016; Dewar et al. 2018; Joshi et al. 2022). *Non‐steady‐state* physics should be incorporated into stomatal optimality theories because stomatal conductance equilibrates with environmental conditions faster than plant water and caron storage. Stomatal conductance typically equilibrates within a few minutes to a few hours (Xiong, Douthe, and Flexas 2018), whereas reaching *steady‐state* can take up to a couple of days for the water storage of large trees (Hunt and Nobel 1987; Hunt, Running, and Federer 1991) and longer for carbon transport dynamics (Nikinmaa, Sievänen, and Hölttä 2014). S*teady‐state* physics exaggerates the domain immediately influenced by stomata to the entire plant, its reproduction, growth, and survival. Conversely, *non‐steady‐state* physics restricts this domain to the leaf because leaf gas exchange immediately affects only how quickly leaf water and carbon stores and leaf temperature change over time (i.e., their time‐derivatives; S2 and S4 in Supporting Information) but not their values per se or the rest of the plant (Figure 1).

### 1.2 A New Generalized Stomatal Optimality Theory

Here, we derive the optimal stomatal behavior that optimizes a generalized fitness proxy, including reproduction, survival, and growth, which is possible without having to explicitly define the fitness proxy mathematically. This mathematical simplification is made possible when the optimization problem is considered as a *dynamic feedback* optimization with *non‐steady‐state* physics (Box 1; Figure 1; S1 in Supporting Information). The solution gives the optimal stomatal conductance to water vapor ($g_{w}$, mol∙m^−2^∙s^−1^) that optimizes any *objective* (O) (or fitness proxy) that depends on plant water and carbon storage, including growth, survival, and reproduction, over a period of time (t, s). The only prerequisite for the *objective* is that it is not formulated in terms of photosynthetic carbon assimilation ($A_{n}$, mol∙m^−2^∙s^−1^), transpiration (E, mol∙m^−2^∙s^−1^), or other terms that instantaneously depend on $A_{n}$ or E (e.g., time‐derivative of leaf water, leaf carbon storage, or leaf temperature). Accordingly, transpiration and photosynthesis are interpreted as having an indirect influence on evolutionary fitness and its proxies by modifying plants' water and carbon storage. However, transpiration and photosynthesis themselves—representing the fluxes of water or carbon that pass through plants—are not the goal per se of evolution. By accounting for *non‐steady‐state* physics (Box 1), we enable a role for plants' storage of water, carbon, and heat in the optimization (Figure 1), which are incorporated as *constraints* on the *dynamic feedback* optimization problem. Mathematically, our optimization problem is

(1) $$\max_{g_{w}}\left[\int O\;\mathrm{dt}\right]$$

Furthermore, we demonstrate four practical applications of its solution. First, for the case with many non‐foliar *constraints* on the optimization (e.g., water and carbon in soils, roots, and stems) and a single foliar *constraint*, representing both leaf water and carbon (i.e., leaf mesophyll osmotic potential, turgor, and total water potential), the *dynamic feedback* optimization simplifies to an effectively *instantaneous* solution under *non‐steady‐state*, enabling more efficient computation like other *instantaneous* models. This solution is equivalent to *instantaneously* maximizing the rate of change of mesophyll turgor pressure or total water potential or minimizing the rate of change of mesophyll osmotic pressure (i.e., maximizing the rate of symplastic solute concentration change) depending on the choice of *constraint* (Figure 1; S4.7 in Supporting Information). Second, our model predicts stomatal conductance generally as well as other stomata models for ponderosa pine (*Pinus ponderosa Dougl. ex Laws*.) seedlings, red maple (*Acer rubrum L*.) and red oak (*Quercus rubra L*.) saplings, and mature Norway spruce ( *Picea abies* (*L*.) *Karst*.) and European larch (*Larix decidua Mill*) trees from field and greenhouse experiments, while making more appropriate assumptions about fitness, its proxies, and the influence of stomata. Third, our model captures realistic stomatal responses to novel environmental cues, including stomatal opening under excessive heat, which was not possible with previous optimality models (Aparecido et al. 2020; Marchin et al. 2023) due to their *instantaneous* nature (Blonder et al. 2023; Mills, Bartlett, and Buckley 2024). Finally, we are able to predict productivity and mortality under drought using the 2018 European drought as a case study.

## 2 Materials and Methods

### 2.1 The Model

#### 2.1.1 Formulation of the Optimization Problem

We consider a plant with reserves of water and carbon distributed throughout its canopy, stem, roots, and adjacent soil. We discretize these reserves into distinct pools. A simple discretization of reserves is shown in Figure 1 with a total of 8 pools (water and carbon pools for soil, roots, stems, and leaves). More complex schemes with additional discretization (e.g., branches vs. boles, upper vs. lower canopy, and distinct patches of a leaf) are possible but do not alter the solution. We assume that changes in each of the non‐foliar water and carbon pools form *constraints* on the optimization (6 non‐foliar *constraints* in total). Based on past work that posed leaf osmotic potential ($\pi_{L}$ , MPa; Table S1 for symbols) as a *cost* to stomatal opening (Deans et al. 2020), we focus on the assumption that mesophyll osmotic potential is the single foliar *constraint*, representing both leaf water and carbon. We make this assumption of a single foliar *constraint* that reflects both leaf water and carbon status because it is critical to achieving our generalized solution below (Box 2), and because currently hypothesized stomatal sensing mechanisms (e.g., mesophyll sugar concentrations, turgor, and water content; Lawson et al. 2014; Sack, John, and Buckley 2018; Buckley 2019) integrate both water and carbon status (Martinez‐Vilalta et al. 2019; Sapes et al. 2021). In our Supporting Information (S4), we consider leaf turgor and total water potential as alternative single foliar *constraints*. Hence, our model considers how stomatal conductance impacts future leaf osmotic potentials (or turgor or total water potential) through photosynthesis and transpiration while optimizing O over time (Equation 1). Photosynthesis causes solutes to accumulate in leaves, making osmotic potential more negative. Meanwhile, transpiration concentrates leaf solutes and cools leaves, which decrease and increase the osmotic potential, respectively.

In our Supporting Information (S1), we solve our *dynamic feedback* optimization problem for the optimal stomatal conductance under *non‐steady‐state* physics through the calculus of variations (Witelski and Bowen 2015). Our solution is defined in terms of the *marginal carbon cost of water* ($\chi_{w}$, mol∙mol^−1^; Table S1),

(2) $$\chi_{w}=-\frac{{\left.\frac{\partial {\dot{\pi }}_{L}}{\partial E}\right|}_{t}}{{\left.\frac{\partial {\dot{\pi }}_{L}}{\partial A_{n}}\right|}_{t}}$$

where the subscripts of *t* denote that the partial derivatives are evaluated with time held constant, and ${\dot{\pi }}_{L}=\frac{d\pi_{L}}{\mathrm{dt}}$ is the rate of leaf mesophyll osmotic potential change (MPa∙s^−1^), which appears because the calculus of variations (Witelski and Bowen 2015) requires defining an augmented Lagrangian (or Hamiltonian) in terms of the rate of change of the *constraints* (Box 1). A shorter, simplified derivation of Equation (2) without explicit consideration of non‐foliar water and carbon pools is shown in Box 2. In our Supporting Information (S4.7), we show that this strategy is equivalent to *instantaneously* minimizing the rate of change of leaf osmotic pressure (${\dot{\pi }}_{L}$) (i.e., maximizing the rate of symplastic solute concentration change, since osmotic potential is negative). In alternative formulations with leaf turgor pressure or total water potential as the single foliar *constraint*, the solutions are equivalent to maximizing the rate of change of leaf hydration.

BOX 2 **Simplified derivation of Equation (2).**

**Simplified derivation of the *marginal carbon cost of water***

To solve for the optimal stomatal conductance ($g_{w}$) that optimizes the *objective* (O) over time (Equation 1), we must solve the calculus of variations (sometimes called Pontryagin's maximum principle; Witelski and Bowen 2015), which states that the optimal stomatal conductance is equivalent to the stomatal conductance that maximizes a Hamiltonian (H; Box 1). For a simplified plant system, considering only leaf osmotic pressure ($\pi_{L}$) as the sole *constraint* (i.e., resource affected dynamically by stomata), our Hamiltonian is

(2.1) $$H=O+\eta_{1}{\dot{\pi }}_{L}$$

where ${\dot{\pi }}_{L}$ is the rate of change of leaf osmotic pressure, and $\eta_{1}$ is the Lagrange multiplier for the osmotic potential (Box 1). Our full derivation (S1 in Supporting Information) differs from this simplified derivation because one foliar *constraint* is assumed a priori here, while the full derivation assumes many *constraints* and shows that only foliar *constraints* determine the solution. To maximize the Hamiltonian with respect to stomatal conductance, we set the partial derivative of the Hamiltonian with respect to stomatal conductance equal to zero,

(2.2) $$0={\left.\frac{\partial H}{\partial g_{w}}\right|}_{t}={\left.\frac{\partial O}{\partial g_{w}}\right|}_{t}+\eta_{1}{\left.\frac{\partial {\dot{\pi }}_{L}}{\partial g_{w}}\right|}_{t}$$

where the subscripts of *t* denote that the partial derivatives are evaluated with time held constant. Stomatal conductance controls photosynthetic net carbon assimilation ($A_{n}$) and transpiration (E), both of which affect the rate of change of leaf osmotic pressure, so Equation (2.2) may be rewritten through the chain rule as

(2.3) $$0={\left.\frac{\partial O}{\partial A_{n}}\right|}_{t}\frac{\partial A_{n}}{\partial g_{w}}+{\left.\frac{\partial O}{\partial E}\right|}_{t}\frac{\partial E}{\partial g_{w}}+\eta_{1}{\left.\frac{\partial {\dot{\pi }}_{L}}{\partial A_{n}}\right|}_{t}\frac{\partial A_{n}}{\partial g_{w}}+\eta_{1}{\left.\frac{\partial {\dot{\pi }}_{L}}{\partial E}\right|}_{t}\frac{\partial E}{\partial g_{w}}$$

At any given time, the *objective* depends on the stores of carbon and water within the plant but does not depend on the fluxes of carbon and water into and out of the leaf. Changes in the photosynthesis and transpiration rates at a moment in time ($t=t^{*}$) due to a coinciding change in stomatal conductance will affect the amount of carbon and water stored inside the plant at all future times after the moment of stomatal conductance change ($t>t^{*}$). However, regardless of how photosynthesis and transpiration rates change, the amount of water and carbon at this moment ($t^{*}$) is effectively fixed because changes in plant carbon and water reserves require time to pass. In this sense, the amount of water and carbon at this moment ($t^{*}$) are like the initial boundary conditions of partial differential equations used to describe dynamic systems. No matter what happens after the initial condition, the initial condition is fixed at its initial conditions by definition. This reasoning is the same as reasoning from the field of Lagrangian mechanics in which a particles' position (x) is not changed by a change in a particle's velocity ($v=\dot{x}$) when time does not pass (${\left.\frac{\partial x}{\partial v}\right|}_{t}=0$) That is, position and velocity are treated as independent variables in Lagrangian mechanics and the calculus of variations (Witelski and Bowen 2015). By analogy, plant carbon and water reserves are treated as independent from their corresponding gas exchange rates ($A_{n}$ and E) in our derivation. Hence, the value of the *objective* at one moment in time is also fixed with respect to changes in photosynthesis and transpiration rates (${\left.\frac{\partial O}{\partial A_{n}}\right|}_{t}=0$, ${\left.\frac{\partial O}{\partial E}\right|}_{t}=0$). Hence, Equation (2.3) simplifies to

(2.4) $$0=\eta_{1}{\left.\frac{\partial {\dot{\pi }}_{L}}{\partial A_{n}}\right|}_{t}\frac{\partial A_{n}}{\partial g_{w}}+\eta_{1}{\left.\frac{\partial {\dot{\pi }}_{L}}{\partial E}\right|}_{t}\frac{\partial E}{\partial g_{w}}$$

The exact value of the Lagrange multiplier is inconsequential because Equation (2.4) may be rearranged into another expression without it, yielding a solution that is equivalent to our full solution (Equations 2 and 5),

(2.5) $$\chi_{w}=\frac{\frac{\partial A_{n}}{\partial g_{w}}}{\frac{\partial E}{\partial g_{w}}}=-\frac{{\left.\frac{\partial {\dot{\pi }}_{L}}{\partial E}\right|}_{t}}{{\left.\frac{\partial {\dot{\pi }}_{L}}{\partial A_{n}}\right|}_{t}}$$

This solution defines a setpoint for the *marginal carbon profit of water* ($\left(\partial A_{n}/\partial g_{w}\right)/\left(\partial E/\partial g_{w}\right)$) called the *marginal carbon cost of water* ($\chi_{w}$). The *marginal profit* and *cost* are equal when the actual stomatal conductance equals the optimal conductance.

Our solution (Equation 2) is effectively *instantaneous* in form despite having been premised as a *dynamic feedback* problem. Typically, the solution of *dynamic feedback* optimization problems is defined in terms of one or more Lagrange multipliers that change over time (Box 1). Indeed, we posed our problem with dynamic Lagrange multipliers for the single foliar and each non‐foliar *constraint* because stomatal optimality theories should consider the resources of the entire plant and its environment. However, these Lagrange multipliers have all been eliminated during the calculus of variations through the mathematics of *non‐steady‐state* physics, under which the extent of immediate stomatal influence is constrained to the leaf (Figure 1). As shown in our Supporting Information (S1), non‐foliar Lagrange multipliers are mathematically irrelevant because, under *non‐steady‐state*, changing the stomatal conductance cannot affect non‐foliar organs distal to the leaf. For example, in the stem, carbon reserves locally support stem growth and respiration as well as the development of reproductive structures and immature leaves. These stem carbon reserves accumulate over time when leaf‐to‐stem carbon transport (e.g., phloem loading) exceeds the sum of stem‐to‐root phloem transport and shoot growth and respiration (Table S2.1; e.g., Thornley 1991; Dewar 1993; Daudet et al. 2002; Lacointe and Minchin 2008; De Schepper and Steppe, 2010) and deplete otherwise. All these processes that affect stem carbon dynamics (i.e., leaf‐to‐stem carbon transport, phloem loading, stem growth, and respiration) depend *instantaneously* on water and carbon reserves (Münch 1930; Borstlap and Schuurmans 2004; Steppe et al. 2006; De Schepper and Steppe 2010; Schiestl‐Aalto et al. 2015; Stanfield and Bartlett 2022; Friend, Eckes‐Shephard, and Tupker 2022; Oswald and Aubrey 2023), but not on stomatal conductance per se. Therefore, due to the limited influence of the stomata conductance on *instantaneous* stem (and analogously, root and soil) dynamics, the Lagrange multipliers for the stem (and root and soil) become mathematically irrelevant in our *non‐steady‐state* theory, leaving only Lagrange multipliers for the leaf relevant to the *non‐steady‐state* optimization problem. For the case of a single foliar *constraint* (but not multiple), we show in our Supporting Information (S1) and Box 2 that the exact value of the corresponding single foliar Lagrange multiplier is irrelevant to $\chi_{w}$ (Equation S1.8). Hence, the Lagrange multipliers do not affect the optimal stomatal strategy under *non‐steady‐state* physics, and our solution is effectively *instantaneous* since *instantaneous* optimization problems lack dynamic Lagrange multipliers (Box 1). Its leaf‐localized behavior is consistent with other non‐optimality‐based, hydromechanical models of stomatal conductance (Buckley, Mott, and Farquhar 2003; Buckley, Turnbull, and Adams 2012) as well as several leaf‐localized physiological phenomena. These phenomena include that the hormone responsible for stomatal closure, abscisic acid (ABA), is mainly synthesized in the leaf in response to changes in leaf turgor pressure or leaf water content (McAdam and Brodribb 2016, 2018; Sack, John, and Buckley 2018; Zhang et al. 2018) and that stomatal closure and ABA synthesis precede soil drying and loss of soil–plant hydraulic conductance (Bourbia and Brodribb 2024; Manandhar et al. 2024).

It is interesting that Equation (2) does not contain any terms related to the *objective* (O) that depends on plant carbon and water reserves. This behavior is predicted because, under *non‐steady‐state* conditions, opening or closing stomata affects the future, but not the current, size of reserves and fitness. Opening or closing stomata affects the rate of change of foliar carbon and water reserves and thus the future trajectory of these reserves and fitness. However, there is no immediate effect on the reserves and fitness at the moment of stomatal change. However, one could argue that if the *objective* does not define the optimal stomatal strategy, could one define an *objective* that has no ecological meaning and achieve the same solution (Equation 2)? This is not the case. The *objective* must have ecological meaning because our mathematical solution is defined primarily by the *constraints* on water and carbon reserves. Those specific *constraints* should be considered only when optimizing an *objective* that depends on water and carbon reserves, such as growth, survival, and reproduction. *Objectives* without ecological meaning would render optimization problems (i.e., their Hamiltonian, H; Box 1) irrelevant to water and carbon reserves and thus would not give our solution.

We believe that the ecological meaning of our optimal stomatal strategy is related to phloem loading and its limitations on plant functioning. Both stomata and phloem loading are located in the leaf and are metabolically regulated by leaf sugar concentrations (Borstlap and Schuurmans 2004; Kelly et al. 2013; Lawson et al. 2014; Miehe et al. 2023). Thus, stomata and phloem loading could be physiologically coordinated together, as previously hypothesized (Nikinmaa et al. 2013; Hölttä et al. 2017; Huang et al. 2018). Phloem loading depends *instantaneously* on osmotic potential through leaf sugar concentration and turgor pressure (Münch 1930; Borstlap and Schuurmans 2004, Stanfield and Bartlett 2022), and thus Equation (2) is also the solution that maximizes loading of sugars from the mesophyll into the phloem over time (i.e., Equation 2 applies when O in Equation (1) is defined as the phloem loading rate). Once loaded from the mesophyll into the phloem, sugars may be transported to sites of growth, repair, and reproduction to enhance fitness.

#### 2.1.2 Relation Between Marginal Carbon Cost of Water and Plant Traits

We further express Equation (2) in terms of physical properties and plant traits through leaf pressure–volume (PV) theory (Tyree and Hamel, 1972; Campbell et al. 1979; Bartlett, Scoffoni, and Sack 2012) and expressions of mass and energy conservation (S4 in Supporting Information; Table S1 for symbols). The relevant physical properties and leaf traits include the leaf apoplastic fraction ($a_{f}$, m^3^·m^−3^), the leaf symplastic osmotic potential ($\pi_{L}$, MPa), their values at full hydration ($a_{f,0}$, m^3^·m^−3^, and $\pi_{L,0}$, MPa), the leaf elastic modulus at full hydration ($\varepsilon_{L,0}$, MPa), the bulk or total leaf relative water content (${\mathrm{RWC}}_{L}^{T}$, m^3^·m^−3^), the leaf saturated water content (${\mathrm{SWC}}_{L}$, kg·kg^−1^; the leaf water mass at full hydration normalized by the dry mass; Nadal et al. 2023), leaf temperature ($T_{L}^{K}$; K), and the leaf dry matter thermal capacitance ($c_{\mathrm{DM}}$, J·K^−1^·kg^−1^). Our expanded solution to Equation (2) is

(3) $$\chi_{w}=\left(1-a_{f,0}\right)\frac{\alpha m_{w}\left|\pi_{L,0}\right|}{\rho_{w}\mathrm{\varpi R}T_{L}^{K}}\left[\frac{{\mathrm{SWC}}_{L}}{{\mathrm{SWC}}_{L}∙{\mathrm{RWC}}_{L}^{T}∙c_{w}+c_{\mathrm{DM}}}∙\frac{\Lambda_{E}}{T_{L}^{K}}-\frac{1}{{\mathrm{RWC}}_{L}^{T}+a_{f}\Delta }\right]$$

where $m_{w}$, $\rho_{w}$, $c_{w}$, and $\Lambda_{E}$ are the molar mass (kg·mol^−1^), density (kg·m^−3^), thermal capacitance (J·K^−1^·kg^−1^), and latent heat of vaporization (J·kg^−1^) of water, ϖ is a conversion factor for converting between units of Pascals to Megapascals (MPa·Pa^−1^), and R is the universal gas constant (J·mol^−1^·K^−1^). Formally, α (mol·mol^−1^) is a constant that describes an assumed relationship between the rate of whole‐leaf net carbon assimilation ($a_{L}A_{n}$, mol·s^−1^, where $a_{L}$ is the leaf area; m^2^) to the molar production rate of the first osmolyte generated photosynthetically inside the leaf symplasm (${\dot{n}}_{L}^{S,A_{n}}$; mol·s^−1^), potentially before sugar or starch synthesis (e.g., triose phosphate; Equation S4.1.15). It is the number of carbon atoms in a molecule of the first osmolyte generated photosynthetically. We calibrate α since our optimality theory does not specify factors that determine its value, such as the identity of the first osmolyte, its location and scale within the symplast (e.g., chloroplast vs. cytoplasm; S4.1 of Supporting Information), and how stomata sense leaf status (Mott, Sibbernsen, and Shope 2008; Lawson et al. 2014; Sack, John, and Buckley 2018; Buckley 2019). We model the apoplastic fraction as a Weibull function of the osmotic potential (Campbell et al. 1979; Andersen, Jensen, and Losch 1991; Urban, Jaffrin, and Chraibi 1993) with $a_{f,\max}$ (m^3^·m^−3^), $\pi_{L}^{*}$ (MPa), and β (unitless) as shape parameters ($a_{f}=a_{f,\max}\exp\left[-{\left(\frac{\pi_{L}}{\pi_{L}^{*}}\right)}^{\beta }\right]$), for which the Δ term in Equation (3) is unitless shorthand for

(4) $$\Delta =-\frac{\varepsilon_{L,0}}{\varepsilon_{L,0}-\pi_{L,0}}\left[\frac{\beta a_{f,0}}{1-a_{f,0}}{\left(\frac{\pi_{L,0}}{\pi_{L}^{*}}\right)}^{\beta }+1\right]$$

Alternative formulations with leaf turgor or total water potential as the single foliar *constraint* have similar solutions for the *marginal cost* as Equation (3), differing only in the last fractional term inside the square brackets (S4.5 and S4.6 in Supporting Information). Equation (3) shows that the *marginal carbon cost of water* varies with leaf temperature ($T_{L}^{K}$) and total leaf water potential ($\psi_{L}$, MPa). The ${\mathrm{RWC}}_{L}^{T}$ and $a_{f}$ terms reflect $\psi_{L}$ through PV theory. The temperature‐dependent terms are the latent heat of vaporization ($\Lambda_{E}$) and the osmotic potentials at current and full hydration ($\pi_{L}$ and $\pi_{L,0}$), which control apoplastic fractions ($a_{f}$ and $a_{f,0}$), producing a weak temperature‐dependence for the apoplastic fractions and Δ. The $\frac{\left|\pi_{L,0}\right|}{\mathrm{\varpi R}T_{L}^{K}}$ term represents an effective symplastic solute concentration at full hydration (mol·m^−3^) that is temperature‐independent despite containing a temperature term (because $\pi_{L,0}\propto T_{L}^{K}$). It explains why more negative osmotic potentials cause stomatal closure (larger $\chi_{w}$; Qiu and Katul 2020; Song et al. 2023). The strongest temperature response is due to the $\frac{\Lambda_{E}}{T_{L}^{K}}$ term.

The *marginal cost* (Equations 2 and 3) translates into an optimal stomatal conductance through the *marginal carbon profit of water* ($\lambda \equiv \left(\partial A_{n}/\partial g_{w}\right)/\left(\partial E/\partial g_{w}\right)$, mol·mol^−1^), given that the *marginal cost* is equivalent to the *marginal profit* when stomata behave optimally (S3 in Supporting Information),

(5) $$\lambda =\chi_{w}$$

Unlike the *marginal cost*, the *marginal profit* is a property of gas exchange and photosynthesis and is independent of the optimization problem (Buckley, Sack, and Farquhar 2017). We use a modified form of Dewar et al. (2018) solution to translate the *marginal profit* into a stomatal conductance for a generic photosynthesis model ($A_{n}=f_{0}\frac{c_{i}-\Gamma^{*}}{c_{i}+\gamma }-R_{d}$, where $c_{i}$ is the leaf intercellular CO_2_ partial pressure (mol·mol^−1^), $\Gamma^{*}$ is the CO_2_ photosynthetic compensation point (mol·mol^−1^), $R_{d}$ is the respiration rate (mol·m^−2^·s^−1^), and $f_{0}$ (mol·m^−2^·s^−1^) and γ (mol·mol^−1^) are parameters). This solution assumes negligible boundary layer and mesophyll resistance (S3.3 in Supporting Information), making it appropriate for leaf‐scale gas exchange measurements,

(6) $$g_{w}=\frac{1.6}{\left(1-x\right)\left(c_{a}-\Gamma^{*}\right)}\left[f_{0}\frac{x}{x+\frac{y}{z}}-R_{d}\right]$$

where x , y , and z are unitless shorthand for

(7) $$x=\frac{c_{i}-\Gamma^{*}}{c_{a}-\Gamma^{*}}=\frac{1-y\frac{R_{d}}{f_{0}}-\sqrt{{\left(1-y\frac{R_{d}}{f_{0}}\right)}^{2}-\left[1-z\left(1-\frac{R_{d}}{f_{0}}\right)\right]\left[1+y\left(\frac{R_{d}}{f_{0}}\frac{y}{z}-1\right)\right]}}{1-z\left(1-\frac{R_{d}}{f_{0}}\right)}$$

(8) $$y=\frac{1.6\lambda \frac{D_{L}}{P_{\mathrm{atm}}}}{c_{a}-\Gamma^{*}}$$

(9) $$z=\frac{1.6\lambda \frac{D_{L}}{P_{\mathrm{atm}}}}{\Gamma^{*}+\gamma }$$

and $c_{a}$ is the air CO_2_ partial pressures (mol∙mol^−1^), $D_{L}$ is the leaf‐to‐air vapor pressure deficit (kPa), and $P_{\mathrm{atm}}$ is atmospheric pressure (kPa). For the classic Farquhar, von Caemmerer, and Berry (1980) photosynthesis model, $f_{0}=V_{c,\max}$ and $\gamma =K_{c}\left(1+\frac{o_{i}}{K_{o}}\right)$ under carboxylation‐limited conditions, where $V_{c,\max}$ is the maximum carboxylation rate (mol∙m^−2^∙s^−1^), $K_{c}$ (mol∙mol^−1^) and $K_{o}$ (mol∙mol^−1^) are Michaelis–Menten constants for carboxylation and oxygenation, and $o_{i}$ is the leaf intercellular O_2_ partial pressure (mol∙mol^−1^). Under electron transport‐limited conditions, $f_{0}=\frac{J}{4}$ and $\gamma =2\Gamma^{*}$ , where J is the electron transport rate (mol∙m^−2^∙s^−1^). In our Supporting Information (S3.3), we derive an alternative equation for $g_{w}$ in terms of λ that assumes that CO_2_ diffusion through stomata and biological carbon fixation are decoupled (i.e., *non‐steady‐state* internal leaf CO_2_ concentrations; Hari et al. 1986). We first solve for the *marginal cost* (Equations 3 and 4), then determine the value of the *marginal profit* by its equivalence to the *marginal cost* (Equation 5), and lastly solve for the stomatal conductance from the *marginal profit* (Equations (6), (7), (8), (9)).

The system of equations (Equations (3), (4), (5), (6), (7), (8), (9)) that make up our optimal stomata solution contains a negative feedback loop (Figure 1) that helps to maintain the homeostasis of foliar non‐structural carbon compounds and photosynthesis rates. Over long timescales, large $A_{n}$ causes photosynthates to accumulate, and $\pi_{L}$ and $\pi_{L,0}$ become more negative (Equations S4.2.5 and S4.3.10), causing an increase in $\chi_{w}$ and λ (Equations 3 and 5), closing stomata and diminishing $A_{n}$ (Equations (6), (7), (8), (9)). This strategy has previously been predicted by a growth‐maximizing stomatal model, which was called the *carbon‐use* strategy (Potkay and Feng 2023a, 2023b, Figure 1). In the previous growth‐maximizing model, the *carbon‐use* strategy resulted from mathematical terms for both whole‐plant carbon storage itself and a dynamic Lagrange multiplier for carbon storage. Whereas in our solution, this *carbon‐use strategy* arises from leaf carbon storage (i.e., the $\pi_{L,0}$ term in Equation 3) and its feedback to assimilation and stomatal conductance, and not because of any Lagrange multipliers. Here, the *carbon‐use* strategy keeps osmotic potential as stable as possible over longer timescales, thereby also stabilizing leaf solute concentration. This long‐term *carbon‐use* strategy has the opposite effect from the optimal stomatal strategy over short timescales, which *instantaneously* maximizes the rate of solute concentration variation at any one moment (S4.7 of Supporting Information). By doing so, the *carbon‐use* strategy should enhance the total phloem loading integrated over time. That is because it is more optimal to continuously load sugars at a stable rate, requiring stable osmotic potentials, than to alternate between periods of high and low loading activity. Although we expect $\pi_{L,0}$ to change over long timescales (Equation S4.2.4 in Supporting Information) in response to leaf gas exchange, phloem loading, and osmotic adjustment, we treat its value normalized to a reference temperature of 25°C ($\pi_{L,0,25{}^{\circ}C}$, MPa) as a constant, though variable among treatments, in our analyses for simplicity and because observed seasonal changes in $\pi_{L,0}$ are generally moderate (Bartlett et al. 2014). For best model practices, see our Supporting Information (S8).

### 2.2 Overview of Data and Parameter Estimation

We fitted our model (Equations (3), (4), (5), (6), (7), (8), (9)) to measurements of stomatal conductance and leaf water potential taken on a range of species from both controlled and field settings (S5 in Supporting Information), including red maple (*Acer rubrum L*.) and red oak (*Quercus rubra L*.) saplings from the Boreal Forest Warming at an Ecotone in Danger (B4WarmED) field experiment in northern Minnesota (Rich et al. 2015; Stefanski et al. 2023), ponderosa pine ( *Pinus ponderosa* ) seedlings from a greenhouse experiment (Sapes and Sala 2021), and mature Norway spruce ( *Picea abies* ) and European larch ( *Larix decidua* ) trees from stands in the Swiss Alps (hereafter called the Lötschental sites; Peters et al. 2021). For the B4WarmED experiment, treatments refer to six combinations of two rainfall manipulations and three temperature treatments. For the greenhouse experiment, treatments refer to two genetically differentiated populations known as the North Plateau (NP) and Northern Rocky Mountain (RM) population. For the Lötschental sites, treatments refer to different individual trees from mesic and xeric stands across an elevation gradient (1300–2200 m asl). When not directly measured, photosynthetic capacities were estimated by the “one‐point method” (De Kauwe et al. 2016; S5 in Supporting Information).

We used the DREAM_(KZS) algorithm (Zhang et al. 2020), a Markov Chain Monte Carlo (MCMC) algorithm and a modified version of the Differential Evolution Adaptive Metropolis (DREAM) algorithm (Vrugt 2016), to fit our model (Equations (3), (4), (5), (6), (7), (8), (9)) with seven parameters (species‐specific: $a_{f,\max},\varepsilon_{L,\max},\pi_{L}^{*},\beta ,{\mathrm{SWC}}_{L},\alpha$, treatment‐specific: $\pi_{L,0,25{}^{\circ}C}$) to the observed stomatal conductance. Six parameters ($a_{f,\max},\varepsilon_{L,\max},\pi_{L}^{*},\beta ,{\mathrm{SWC}}_{L},\alpha$) were assumed to be constant within species since PV traits of sunlit mature leaves tend to vary little within species (Browne et al. 2023). One parameter, the leaf osmotic potential at full hydration and standard temperature of 25°C ($\pi_{L,0,25{}^{\circ}C}$), was allowed to vary among treatments, since the *carbon‐use* strategy suggests that $\pi_{L,0}$ varies among individuals of different environments. DREAM_(KZS), its parameters, our method of differentiating between species‐specific and treatment‐specific parameters (Figure S1), and prior distributions are detailed in the Supporting Information (S6). Parameter combinations were discarded when they did not result in near‐complete stomatal closure at the leaf wilting point (Figure S2; S6.2–S6.3 in Supporting Information).

### 2.3 Comparison to the USO Model

We compare our model's fit to that of the widely used Universal Stomatal Optimization (USO) model (Medlyn et al. 2011) to test the quality of our model's predictions (S6.4 in Supporting Information). The USO model's sole parameter ($g_{1}$; kPa^0.5^) was calibrated twice for each species and treatment. First, we found the $g_{1}$ that minimized the root‐mean square error (RMSE) between observed and predicted $g_{w}$ (Equation S6.4.2). This calibration is consistent with how we parameterized our model using DREAM_(KZS) because DREAM_(KZS) maximizes likelihood, which is generally inversely‐proportional to RMSE (Hodson 2022). Second, we statistically modeled log‐transformed point‐estimates of $g_{1}$ through linear mixed‐effects models (S6.4 in Supporting Information) following Potkay, Sloan, and Feng (2024) to identify environmentally controlled variations in $g_{1}$, which in negatively related to the *marginal profit* (λ; Medlyn et al. 2011) and thus also the *marginal cost* ($\chi_{w}$).

### 2.4 Environmental Responses

We confirmed that our model captures realistic stomatal responses to leaf water potential, temperature, and osmotic potential at full hydration, a proxy for foliar carbohydrate storage (all else constant; S7.1 in Supporting Information). Ponderosa pine was chosen for these predictions because our analyses required additional measurements that were performed only on ponderosa pine (Sapes and Sala 2021; S7 in Supporting Information). Whereas independent estimates of photosynthetic capacities were used in calibration, photosynthetic capacities were modeled as sigmoidal functions of leaf water potential (Tuzet, Perrier, and Leuning 2003; Equation S7.1) for these and subsequent simulations. We compared these simulations to predictions by the classic stomatal optimality model of Cowan and Farquhar (1977) because it captures only the indirect effects of temperature (temperature‐dependent photosynthetic parameters and constant $\chi_{w}$; S7.4 in Supporting Information).

Since VPD may be altered by changing air temperature or relative humidity (RH), we developed a minimalist whole‐plant model of leaf energy balance and plant hydraulics (S7.2 in Supporting Information) using our stomata model (Equations (3), (4), (5), (6), (7), (8), (9)) to predict stomatal conductance, transpiration, leaf temperature, and leaf relative water content when varying either only RH or only air temperature (all else constant). For these simulations, whole soil–plant conductance for ponderosa pine was estimated from measurements of transpiration and soil–leaf water potential differences and modeled as an exponential function of leaf water potential with a Q_10_‐type temperature dependence (Equation S7.2.5; Figure S3). For predictions at temperatures greater than observed, the temperature‐dependence of photosynthetic capacities was modified to include a thermal optimum (Kattge and Knorr 2007). We compared these predictions by our model to those by the Cowan and Farquhar (1977) model and Sperry et al. (2017) Gain‐Risk model (S7.4 in Supporting Information) because it exemplifies recent approaches in stomatal optimality modeling by *instantaneously* maximizing the weighted sum of photosynthesis and soil–xylem hydraulic conductance, each weighted by their respective maximums.

### 2.5 Ecosystem Scale Predictions

Predictions of ecosystem productivity and tree mortality are fundamental to answering questions about basic biology, ecosystem management, and climate‐feedback (McDowell et al. 2022). We demonstrate our stomatal optimality model's use by predicting ecosystem productivity and mortality of Norway spruce‐dominated forest at the Oberbärenburg (DE‐Obe) eddy covariance site in Germany during the 2018 European drought with a modified version of our minimalist whole‐plant model (S7.5 in Supporting Information) forced with observed environmental conditions (Figure 4a). Summer 2018 was exceptionally hot and dry in northwestern Europe (Buras, Rammig, and Zang 2020; Peters et al. 2020), during which time ecosystem‐level carbon fluxes diminished (Smith et al. 2020), and widespread forest mortality was observed (Schuldt et al. 2020).

For these ecosystem scale predictions, we extended our minimalist whole‐plant model to predict changes in soil moisture and to account for light limitations and the effect of leaf area phenology on soil–plant conductance (S7.5 in Supporting Information). We used the stomatal parameters that we determined for Norway spruce at the Lötschental site and calibrated several other parameters related to plant hydraulics, soil evaporation, and photosynthetic capacity for the prior 2017 non‐drought year (S7.5.4 in Supporting Information). Soil hydraulic properties were predicted by pedotransfer functions developed for German forest soils (Teepe, Dilling, and Beese 2003) using soil properties from SoilGrids (https://soilgrids.org/). Observed carbon assimilation and evapotranspiration were independently estimated from the site's eddy covariance measurements of gross primary productivity and latent heat (Bernhofer et al. 2022), which we converted to leaf area‐specific rates through Terra‐MODIS/Aqua‐MODIS/VIIRS‐derived leaf area index (Pu et al. 2023a, 2023b). We compare the predictions of our model to Sperry et al. (2017) Gain‐Risk model, which exemplifies recent stomatal optimality approaches. Neither model was finely tuned to match observations, and we focus on their abilities to capture fundamental processes. Both models were parameterized with the same parameter values estimated from the 2017 non‐drought year (S7.5.4 in Supporting Information).

## 3 Results

### 3.1 Testing the Model

Agreement between observed and predicted stomatal conductances using the best parameters from the MCMC (Figure S4) was best for ponderosa pine (*r* ^2^ = 0.50; RMSE = 0.044 mol·m^−2^·s^−1^), European larch (*r* ^2^ = 0.43; RMSE = 0.070 mol·m^−2^·s^−1^), and Norway spruce (*r* ^2^ = 0.41; RMSE = 0.020 mol·m^−2^·s^−1^). Performance was worse for red maple (*r* ^2^ = 0.35; RMSE = 0.040 mol·m^−2^·s^−1^) and red oak (*r* ^2^ = 0.18; RMSE = 0.077 mol·m^−2^·s^−1^) from the B4WarmED experiment. Observed and predicted stomatal conductances were significantly correlated for all species (*p* < 0.001). Parameters were often intercorrelated; nonetheless, posterior distributions were nonuniformly distributed with clear modes (Figures S5–S9). Within each species, differences in $\pi_{L,0,25{}^{\circ}C}$ among treatments were sometimes significant but always small (Figure S10). Predicted apoplast fractions were realistic (Bartlett, Scoffoni, and Sack 2012; Figure S11).

Our model generally performed as well as, if not slightly better than, the USO model in terms of RMSE and *r* ^2^ (Figure S12; S6.4 in Supporting Information). Variations in $g_{1}$ (and thus $\chi_{w}$) point estimates were often only weakly explained by environmental conditions in our datasets, and their correlations were sometimes unrealistic for some species (Figure S13; S6.4 in Supporting Information). We also tested an alternative version of our model that extends *non‐steady‐state* physics to leaf internal CO_2_ partial pressures ($c_{i}$, mol·mol^−1^) (Hari et al. 1986; S3.2 in Supporting Information), altering the mathematical meaning for the *marginal profit* (Equation S3.2.3) and optimal stomatal conductance (Equation S3.2.7 instead of Equations (6), (7), (8), (9); Figure S14). It had no consistent effect on the quality of predictions (Figures S15–S20).

### 3.2 Environmental Responses

**FIGURE 2:**
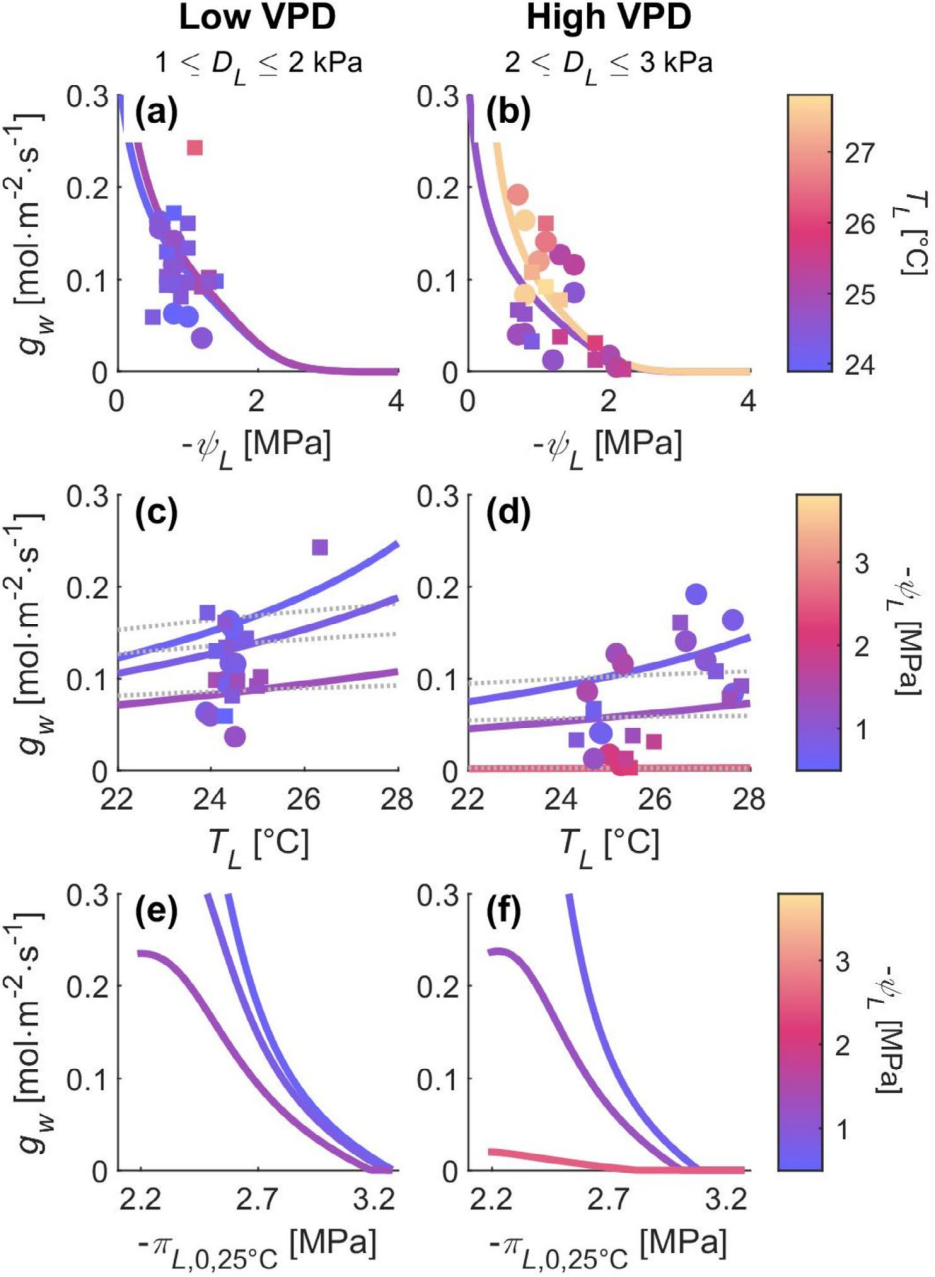
Observations (markers) and predicted responses (lines) of stomatal conductance ($g_{w}$) to leaf water potential ($\psi_{L}$; a, b), leaf temperature ($T_{L}$; c, d), and osmotic potential at full hydration at a standard temperature of 25°C ($\pi_{L,0,25{}^{\circ}C}$; e, f) at low VPD (1 ≤ $D_{L}$ ≤ 2 kPa for observations; 1.5 kPa for predictions; a, c, e) and high VPD (2 ≤ $D_{L}$ ≤ 3 kPa for observations; 2.5 kPa for predictions; b, d, f) for ponderosa pine seedlings. In (a) and (b), marker and line colors denote leaf temperature, and the two lines show predictions at different leaf temperatures chosen as the 10th and 90th quantiles of observed leaf temperatures within the respective VPD bin. In (c) through (f), marker and line colors denote leaf water potential, and the three lines show predictions at different leaf water potentials chosen as the 10th, 50th, and 90th quantiles of observed leaf water potentials within the respective VPD bin. In (c) and (d), predictions of temperature‐responses due solely to the temperature‐response of photosynthetic parameters are also shown (i.e., constant $\chi_{w}$ with respect to temperature; thin dotted lines). In (e) and (f), $\pi_{L,0,25{}^{\circ}C}$ was varied between 0.8–1.2 times the mean of its fitted values averaged among treatments, where the range is based on the variable's natural range post‐drought (Bartlett et al. 2014). See Section S7.1 in Supporting Information for further detail.

Like observations, predicted stomatal conductances declined as leaves were dehydrated, although less so at warmer leaf temperatures (Figure 2a,b). Stomatal conductance increased with leaf temperature when leaves were sufficiently hydrated (Figure 2c,d). Stomatal conductance decreased as leaves accumulated more soluble carbohydrates and as their osmotic potentials became more negative (Figure 2e,f).

**FIGURE 3:**
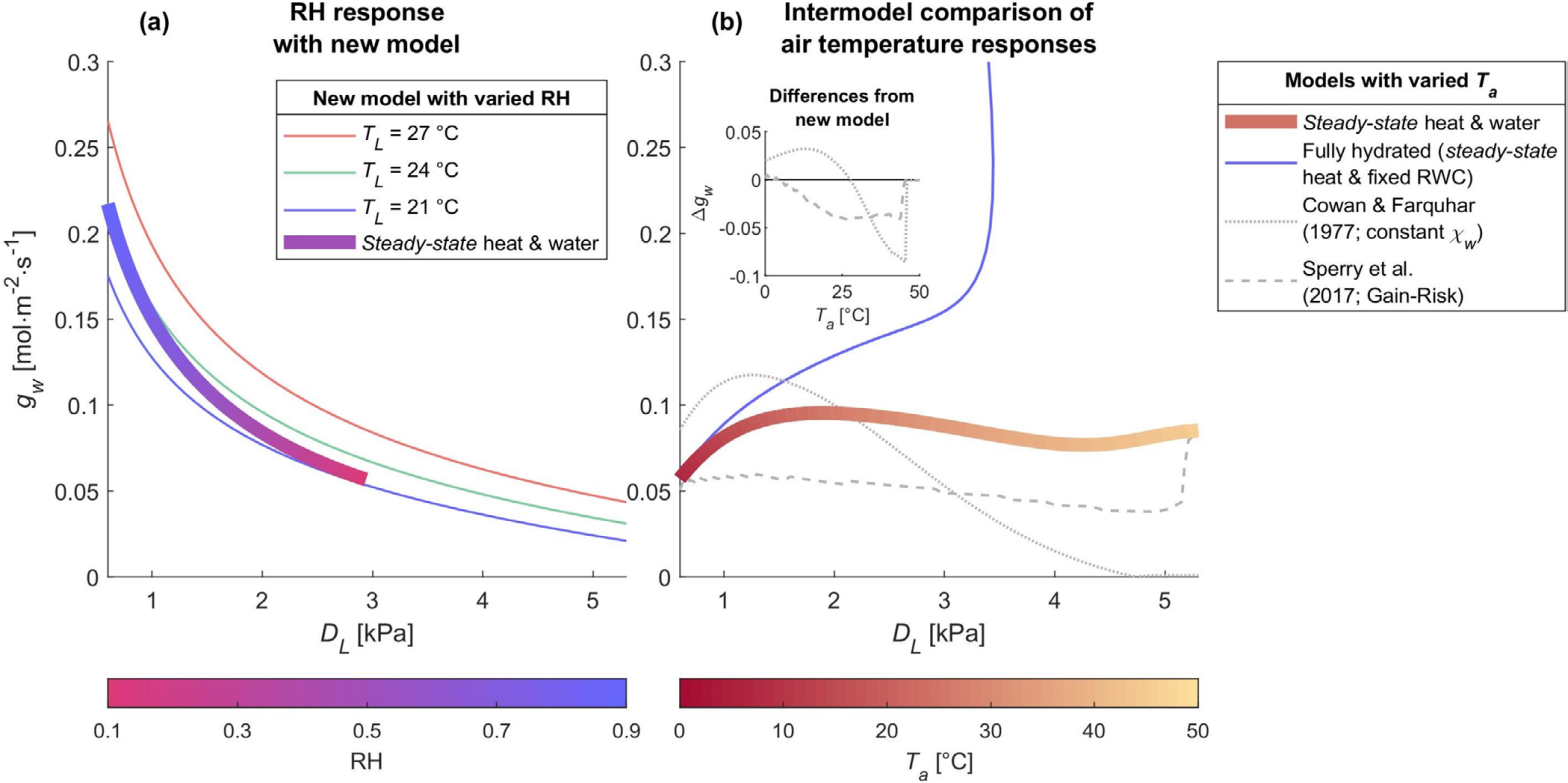
Predicted response of stomatal conductance ($g_{w}$) to VPD ($D_{L}$) for ponderosa pine. Thin blue, green, and red lines in (a) show the response at constant leaf temperature ($T_{L}$; 21°C, 24°C, and 27°C) and full leaf hydration (${\mathrm{RWC}}_{L}^{T}=1$). Thick lines show responses with $T_{L}$ predicted from *steady‐state* leaf heat balance and *steady‐state* plant hydraulics with either (a) constant air temperature ($T_{a}$) of 24°C and varied relative humidity (RH) or (b) a constant RH of 0.5 and varied $T_{a}$. Thin blue solid line in (b) shows the response with leaf temperatures predicted from *steady‐state* leaf heat balance and full leaf hydration (${\mathrm{RWC}}_{L}^{T}=1$) for varied $T_{a}$ and constant RH of 0.5. Thin gray dotted line in (b) shows the temperature‐responses due solely to changes in photosynthetic parameters (i.e., constant $\chi_{w}$) according to Cowan and Farquhar (1977), and the thin gray dotted line in (b) shows the predictions of the Gain‐Risk model of Sperry et al. (2017), both of which were predicted for varied $T_{a}$ and a constant RH of 0.5 and include *steady‐state* leaf heat balance and *steady‐state* plant hydraulics. Subplot in (b) shows the same results expressed in terms of model differences from the *steady‐state* results with our model and $T_{a}$. In simulations labeled as fully hydrated, photosynthetic capacities vary only with $T_{L}$, since leaf hydration is constant, while in all other simulations (including constant $\chi_{w}$ and Gain‐Risk), photosynthetic capacities vary with both $T_{L}$ and leaf hydration. See Section S7.2 in Supporting Information for further details.

Our model's direct temperature‐dependence on the *marginal cost* (Equation 3) causes additional stomatal opening (Figures 2a–d and 3) at high temperatures compared to the model of Cowan and Farquhar (1977); thin gray dotted lines in Figures 2c,d and 3b, especially when leaves are more hydrated (Figures 2c,d and 3b). Under constant leaf temperatures and foliar hydration, our model predicts that stomatal conductance is larger and slightly less sensitive to VPD at warmer temperatures (Figure 3a), thin lines; VPD sensitivity quantified by the m metric (Oren et al. 1999; Katul, Palmroth, and Oren 2009).

Varying RH with constant air temperature, stomata continuously closed as VPD rose in a manner that was more sensitive to VPD (m = 0.64) than if leaf temperature and hydration status were constant (0.47 ≤ m ≤ 0.51; Figure 3, thick lines). Conversely, varying air temperature with constant RH, stomatal conductance followed a generally concave‐down shape for air temperatures less than 40°C (Drake et al. 2018), peaking at a VPD of ~2 kPa and an air temperature of ~25°C. For air temperatures cooler than 25°C, stomata closed as temperatures fell, despite low VPD, due to slowing photosynthetic activity. At air temperatures warmer than 25°C, rises in VPD lead to increasing hydraulic stress, dehydration, and stomatal closure (Figure S21c). For temperatures greater than 40°C, stomata opened as temperature rose to partially cool leaves (Figure 3b). This overall response to temperature is consistent with observations of stomatal conductance peaking between 20°C and 30°C with some studies reporting additional stomatal opening at extreme temperatures (> 40°C; Way et al. 2011; Slot, Garcia, and Winter 2016). The Cowan and Farquhar (1977) model also captured this peaked response but predicted complete stomatal closure at high temperatures (Figure 3b), unlike past reports (Way et al. 2011; Slot, Garcia, and Winter 2016). Unlike past observations and our model, Sperry et al. (2017) Gain‐Risk model (thin gray dashed line in Figure 3b) generally predicts a weak negative response of stomatal conductance to air temperature with a shallow peak at low temperatures and an unrealistic sudden spike in stomatal conductance at extreme temperatures.

When leaf water contents were forced constant to prevent hydraulic stress, stomatal conductance increased continuously with air temperature (thin blue solid line in Figure 3b), like past observations (Drake et al. 2018; Aparecido et al. 2020), despite rising VPD, suggesting that hydraulic stress causes stomatal closure under extreme heat rather than heat per se (Urban et al. 2017; Marchin et al. 2022; Stefanski et al. 2023). When leaf water contents varied as predicted by our minimalist whole‐plant model, leaf temperatures were never warmer than air temperatures by 2°C or more under varying air temperatures (Figure S21a), and this difference lessened as air temperatures and transpiration increased. In general, transpiration and leaf water potential gradually approached their critical values (at which transpiration is maximal; Sperry et al. 1998) as air temperatures rose, reflecting increasingly risky water use, and ultimately reaching their critical values at ~45°C (Figure S21b,c). Within a limited range of air temperatures (~±3°C) centered around the photosynthetic thermal optimum (~35°C), water use briefly became less risky as temperatures increased. We further analyzed our minimalist model's predictions of supercritical water potentials (S7.2 in Supporting Information). For temperatures warmer than ~45°C, our model predicts unstable supercritical water potentials (Figure S22a,c) due to evaporative demand exceeding hydraulic supply (Figure S22g), leading to desiccation. For moderate temperatures and drought, our model predicts stable supercritical water potentials (Figure S22a,c), in which hydraulic supply satisfies evaporative demand at a supercritical leaf water potential (Figure S22f). Observations of leaf water potentials from ponderosa pine supported our model's prediction of the existence of supercritical water potentials (Figure S22d; S7.3 in Supporting Information). However, due to the simplifications of our plant hydraulic scheme, these supercritical water potentials may better reflect near‐critical water potentials that are similarly associated with high percent loss conductance (PLC) of the hydraulic pathway (see Section 4 below).

### 3.3 Ecosystem Scale Productivity and Mortality Predictions Under Drought

**FIGURE 4:**
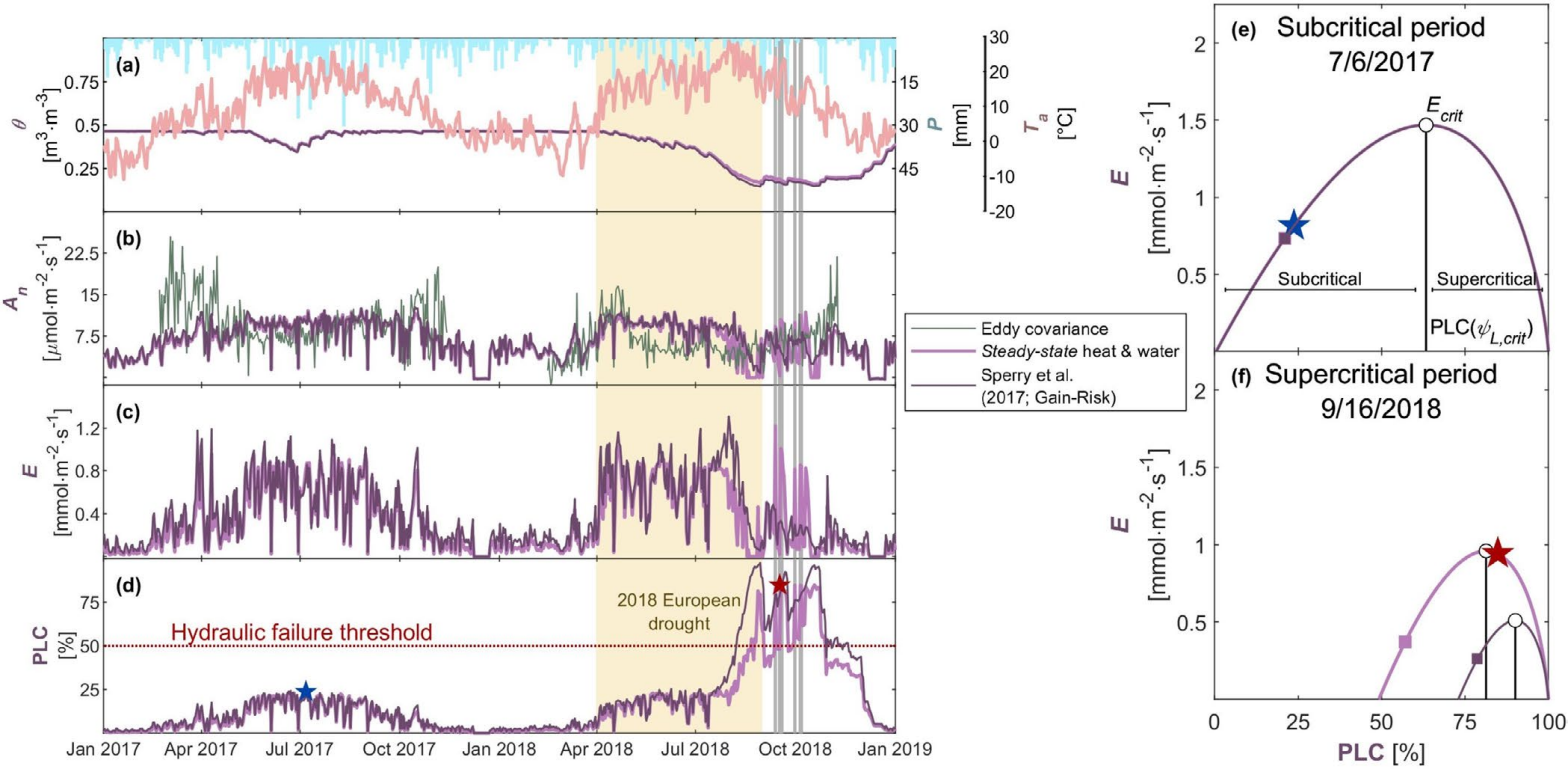
Midday (12 noon) ecosystem predictions at the Norway Spruce‐dominated Oberbärenburg eddy covariance site in Germany between 2017 and 2018 with our model (lighter thick lines in b–f) and the Gain‐Risk model of Sperry et al. (2017; darker thin lines in b–f). (a) Observed environmental conditions, including precipitation (*P*; blue line) and air temperature (*T*_*a*_; red line), and predicted volumetric soil moisture (*θ*; purple lines). (b) Simulated leaf area‐specific net carbon assimilation (*A*_*n*_; purple lines) and eddy covariance and LAI‐based estimate (dark green line, shown when LAI ≥ 0.6). (c) Predicted leaf area‐specific transpiration (*E*) (purple lines). (d) Simulated percent loss conductance (PLC; purple lines), which is a predictor of mortality. In (a–d), the 2018 drought is shaded beige based on SPEI < −1 (Vicente‐Serrano, Beguería, and López‐Moreno 2010), and the days on which our model simulates supercritical water potentials are shaded gray. The models' predicted relationships between transpiration (*E*) and PLC are shown for 2 days during (e) the 2017 non‐drought year (blue star in d; 7/6/2017) and (f) the 2018 drought year (red star in d; 9/16/2018). In (e, f), light and dark lines are the potential *E*‐PLC relationships for our model and the Gain‐Risk model, respectively, which overlap in (e) due to nearly identical soil moisture in 2017. The maximum transpiration rate is the so‐called critical transpiration rate (*E*_*crit*_), which corresponds to a critical leaf water potential (*ψ*_*L,crit*_) and its corresponding critical PLC. Subcritical and supercritical water potentials refer to leaf water potentials less and more negative than the critical water potential, respectively. In (e, f), stars are the optimal *E* and PLC values predicted by our model, dark squares are the optimal *E* and PLC values predicted by the Gain‐Risk model, and light squares are the optimal *E* and PLC values that would be predicted by the Gain‐Risk model if using the same soil moisture as our model.

The Gain‐Risk model and our model predict near identical midday net carbon assimilation and transpiration in the 2017 non‐drought year (Figure 4b,c). The inter‐model differences for 2017 are even smaller when evaluated in terms of daily GPP and daily evapotranspiration (Figure S23). Both models underpredict leaf area‐specific carbon assimilation at the beginning and end of each year when LAI is small (Figure 4b,c) and perform better at these low‐LAI times when evaluated in terms of ground area‐specific fluxes (Figure S23), suggesting that the models' underprediction results in part from the normalization of ground area‐specific fluxes by small LAI (S7.5 in Supporting Information). Although our simulations considered LAI seasonality (S7.5 in Supporting Information), accounting for more aspects of phenology and trait dynamics (e.g., photosynthetic capacities and PV traits) would likely improve both model's predictions (Mäkelä et al. 2004; Sabot et al. 2022b; Flo et al. 2024). Both models predict subcritical leaf water potentials during non‐stressed periods (Figure 4e). In July 2018, the two models diverged. The Gain‐Risk model predicts more aggressive water use and sustained photosynthesis than our model during early, moderate drought (June 2018) and the opposite during late, and severe drought (July 2018; Figure 4b,c; Figure S20). The more aggressive water‐use of the Gain‐Risk model during the early phases of drought resulted in faster depletion of soil water storage (Figure 4a) and faster losses in plant–soil conductance (PLC; Figure 4d) than our model. For an assumed PLC mortality threshold of 50% for conifers (Brodribb and Cochard 2009), the Gain‐Risk model predicts mortality 2 weeks before our model does (both in August; Figure 4d). For an alternative PLC threshold of 85% (Venturas et al. 2018; Hammond et al. 2019), the Gain‐Risk model predicts mortality in mid‐August, while our model predicts mortality nearly a full month later in mid‐September after the drought. In late August and September 2018, our model predicted stable supercritical water potentials (Figure 4f; vertical gray bars in Figure 4a–d), causing the PLC predicted by our model to slightly exceed that of the Gain‐Risk model despite greater soil drying in the Gain‐Risk model simulations.

## 4 Discussion

Forecasting the future of global water and carbon cycles requires models of stomatal conductance. Large‐scale ecosystem models increasingly rely on plant optimality models to predict stomatal conductance (De Kauwe et al. 2015b; Eller et al. 2020; Sabot et al. 2020; Wang and Frankenberg 2022). The reliability of these optimality models under unobserved, future environmental conditions is contingent on the appropriateness of their underlying assumptions about the optimization *objectives* of plants, their timescales, and other biological processes. By considering stomatal behavior as a *dynamic feedback* optimization problem with *non‐steady‐state* physics (Figure 1), we have developed a new stomatal optimality approach that optimizes a generalized proxy of evolutionary fitness integrated over plant lifetimes. The most essential contribution here is in our new model's ability to move beyond photosynthetic carbon assimilation as the proxy for evolutionary fitness (Cowan and Farquhar 1977; Wolf, Anderegg, and Pacala 2016; Sperry et al. 2017; Dewar et al. 2018), a default assumption adopted by stomatal optimality models since the 1970s that, before now, could not be relaxed because of their use of *steady‐state* physics. This contribution is critical because real plants operate within a *non‐steady‐state* world, like in our model.

We demonstrate the predictive ability of our optimal stomatal strategy by showing that it compares well to measurements taken on ponderosa pine ( *Pinus ponderosa* ) seedlings, red maple (*Acer rubrum L*.) and red oak (*Quercus rubra L*.) saplings, and mature Norway spruce ( *Picea abies* ) and European larch ( *Larix decidua* ) trees from a wide range of field and greenhouse experiments (Rich et al. 2015; Peters et al. 2021; Sapes and Sala 2021; Stefanski et al. 2023; Figure S4), and predicts realistic responses to various environmental cues, including hydraulic stress, temperature, and VPD (Figures 2 and 3). Our model performs as well as, if not better than, the widely‐used USO model (Medlyn et al. 2011; Figure S12). Unlike the USO model, our parameters are based on plant processes and traits most of which (all but α) can be measured independently of gas exchange. Nevertheless, many previous stomatal optimality models predict realistic stomatal conductance (Wang et al. 2020; Bassiouni and Vico 2021; Sabot et al. 2022a) and similar responses to environmental cues (Dewar et al. 2018; Wang et al. 2020; Potkay and Feng 2023a). Hence, selecting which models are most promising cannot be decided on their predictive power alone. We should also appraise them based on the appropriateness of their underlying assumptions (Parker and Maynard Smith 1990; Berninger, Mäkelä, and Hari 1996). By assuming the maximization of reproduction, growth, and survival integrated over time (i.e., *dynamic feedback* optimization) under *non‐steady‐state* physics, our model makes more realistic assumptions about fitness and the controls that stomata have on it.

The more realistic assumptions in our model allow it to offer new theoretical insights into stomata behavior compared to previous optimization models. For example, our model is the first to justify *instantaneous* behavior at the scale of individual plants, whereas previous optimality models have only justified it at the ecosystem‐scale (Wolf, Anderegg, and Pacala 2016; see S1.1 in Supporting Information for full discussion). Our model's *instantaneous* behavior resulting from *non‐steady‐state* physics suggests that soil water or competition for soil water does not exert a direct influence on stomata (unlike past stomatal optimality models; Cowan 1982, 1986; Mäkelä, Berninger, and Hari 1996; Manzoni et al. 2013a; Lu, Duursma, and Medlyn 2016; Lu et al. 2020; Mrad et al. 2019). Instead, soil water and neighboring plants' water uptake affect stomata only indirectly through leaf water potential. Other non‐stomatal mechanisms (e.g., leaf area adjustments) may cause transpiration to effectively conserve or compete for soil water (see S1.1 in Supporting Information for full discussion). Additionally, our model offers, for the first time, an explanation for the existence of a direct relationship between the *marginal carbon cost of water* or related terms (e.g., the USO model's *g*_1_) and leaf water potential without recourse to involving soil water, which is distal to stomates' physiological sensing mechanism in the leaf mesophyll (Mott, Sibbernsen, and Shope 2008; Buckley 2019). Our explanation results by assuming *non‐steady‐state* physics and treating leaf osmotic potential as a *constraint* on the *dynamic feedback* optimization (Figure 2a,b). The dependence of *marginal carbon cost* on leaf water potential is often observed in leaf gas‐exchange measurements (Manzoni et al. 2011; Zhou et al. 2013; Yang et al. 2019) and supported by ecosystem modeling (De Kauwe et al. 2015a; De Kauwe et al. 2015b; Liu et al. 2020; Bassiouni and Vico 2021; Hawkins et al. 2022; Sabot et al. 2022a) but lacks explanation. It has been presented as an empirical correction to the classic stomatal optimization by Cowan and Farquhar (1977). However, this empirical correction either invalidates Cowan and Farquhar's requirement that the *marginal cost* be constant in time and uniform in space or requires additional assumptions related to a *hydraulic cost* that has not been directly measured (Anderegg et al. 2018; Zenes et al. 2020). Like our model, soil water‐saving optimality models (Cowan 1982, 1986; Mäkelä, Berninger, and Hari 1996; Manzoni et al. 2013a; Holtzman et al. 2024) predict stomatal closure during drought. They do so through their prediction of a direct relationship between the *marginal cost* and either soil water storage or soil water potential. Hence, they can predict an indirect relationship between the *marginal cost* and leaf water potential if their relationship between the *marginal cost* and soil water is coupled with another expression that relates soil and leaf water potentials (e.g., Darcy's law). That is, they predict that the *marginal cost* may appear correlated with leaf water potential, but its changes do not result because of leaf water potential changes per se. Whereas soil water‐saving models propose that stomata close because they sense soil water losses, our model proposes that stomata close because they sense nearby physiological signals in the leaf‐like osmotic potential, turgor pressure, and water content (McAdam and Brodribb 2016, 2018; Sack, John, and Buckley 2018; Zhang et al. 2018).

The explicit relationship derived for the *marginal carbon cost of water* makes our model effective at predicting stomatal response to climate change scenarios, including under drought, elevated CO_2_ concentrations, and extreme heat. Our model predicts a *carbon‐use* strategy (Potkay and Feng 2023b) that gradually promotes more stomatal closure under elevated CO_2_ concentrations than would be predicted if the *marginal cost* were constant. Accordingly, stomata close as carbohydrates accumulate (Figures 1 and 2e,f; Kelly et al. 2013; Lawson et al. 2014) because carbon assimilation becomes less necessary to maintain minimum carbohydrate levels and to satisfy sink demands (Potkay and Feng 2023b). All else being equal, carbon assimilation rates increase under elevated CO_2_ concentrations, initially exceeding leaves' ability to export carbohydrates by phloem loading, leading to foliar soluble carbohydrates accumulating (Wullschleger, Tschaplinski, and Norby 2002). This accumulation causes the osmotic potential at full hydration ($\pi_{L,0}$) to become more negative (Morse et al. 1993; Ferris and Taylor 1994; Vivin et al. 1996) and in turn increases the *marginal cost* (Equation 3; Figure 1; Qiu and Katul 2020; Song et al. 2023), further closing stomata as CO_2_ concentrations rise (Katul, Palmroth, and Oren 2009; Katul et al. 2010; Manzoni et al. 2011; Nakad et al. 2023).

Under extreme heat, our model predicts a trade‐off between heat stress and hydraulic stress (in the absence of photosynthetic or osmotic acclimation). Accordingly, stomata open to partially cool leaves and alleviate heat stress at the expense of hydraulic efficiency and soil–xylem conductance (Figure S21). This tradeoff is enabled by a direct effect of leaf temperature on the *marginal carbon cost of water* (Equation 3) and thus stomatal conductance (Equations (5), (6), (7), (8), (9)) in addition to the indirect effects that arise from the temperature‐sensitivities of photosynthesis. This direct temperature effect predicts enhanced stomatal opening as temperature rises (Figures 2, 3), thereby partially cooling plants (Aparecido et al. 2020; Marchin et al. 2023) and delaying heat‐induced foliar mortality (Blonder et al. 2023), especially when leaves are well hydrated (Urban et al. 2017) or at extreme air temperatures (Figures 2, 3; Figure S18). These behaviors are contrasted against predictions from Sperry et al. (2017) Gain‐Risk model (thin gray dashed line in Figure 3b), which is archetypal of recent stomatal models that are explicitly designed to minimize the loss of xylem or soil–xylem conductance (Sperry and Love 2015; Sperry et al. 2016; Eller et al. 2020; Wang et al. 2020). Because of their hydraulic overemphasis, they fail to capture realistic stomatal responses to temperature that our model predicts by permitting some loss of hydraulic efficiency to respond to heat stress (Figure 3b; Figure S18), especially under extreme heatwaves (> 45°C) when plants must transpire and cool down at the expense of hydraulic safety or die by heat damage. Additionally, our model can explain how these stomatal heat responses vary with leaf economic traits, showing that leaves with shorter lifespans should express greater stomatal opening as temperatures rise as previously hypothesized (Blonder et al. 2023; Mills, Bartlett, and Buckley 2024). Our *marginal cost* declines as temperatures rise (Equation 3), opening stomata, and this opening is enhanced for leaves with greater saturated water content. Since leaf lifespan is negatively correlated with saturated water content (Reich 2014; Nadal et al. 2023), our model thus predicts a greater positive effect of temperature on stomatal conductance for leaves with a shorter lifespan. In other words, short‐lived “fast” leaves tend to respond more quickly to heating.

Finally, we present a path to incorporate our stomatal optimality model into ecosystem models for predicting future plant productivity and mortality. Our solution is as computationally efficient as other *instantaneous* stomatal optimality models that have been integrated into ecosystem models (Eller et al. 2020; Sabot et al. 2020; Wang and Frankenberg 2022), while being theoretically robust due to its framing as a *dynamic feedback* optimization that accounts for how current resource‐use will impact the future availability of resources and thus also fitness. We integrated our stomatal optimality model (Equations (3), (4), (5), (6), (7), (8), (9)) with a minimalist whole‐plant model to predict productivity and mortality during the 2018 European drought at the Oberbärenburg (DE‐Obe) eddy covariance site (Figure 4; Figure S20). Under non‐drought conditions of 2017, our model predictions were nearly identical to that of the Gain‐Risk model, which is promising since the Gain‐Risk model's predictions have been well validated at other forest sites, nearly all of which were observed under non‐drought conditions (Sabot et al. 2022a). The two models diverged during the 2018 drought, predicting different water‐use strategies, soil water drying rates, and timing of mortality events for the dense forest site, whereas the Gain‐Risk model's drought predictions have been validated for predominantly young saplings in research gardens (Venturas et al. 2018) and growth chambers (Wang et al. 2019). The Gain‐Risk model and similar *instantaneous* stomatal optimality models are being used to predict mortality (Sperry et al. 2019; Quetin et al. 2023), although their mortality predictions have yet to accurately capture observed mortality trends (De Kauwe et al. 2020; Venturas et al. 2021). We are unaware of any observations of mortality or PLC at the Oberbärenburg to compare to our simulations. Nonetheless, the first signs of mortality (crown damage) were observed at other German spruce‐dominated sites during late August and September (Obladen et al. 2021) when our model simulated lethal PLC (~85%) and supercritical water potentials that are theorized to lead to inevitable desiccation. We note that supercritical water potentials can be simulated by relatively simplistic plant hydraulic schemes like ours (Comstock and Sperry 2000; Manzoni et al. 2013b; Manzoni, Katul, and Porporato 2014), which predict transpiration to be maximal at an intermediate water potential (i.e., their critical values). However, supercritical water potentials do not emerge from more complex, spatially explicit hydraulic schemes (Ross and Bristow 1990; Sperry et al. 1998; Couvreur et al. 2018), which predict that transpiration increases monotonically as leaf water potentials become more negative, so their critical water potentials are always −∞. These and similar studies often report a finite, *operational* critical water potential at which the soil–plant hydraulic conductance is some (typically small) fraction of the maximum value possible for a given soil water potential (Sperry et al. 1998, 2017; Carminati and Javaux 2020). As the existence of finite critical water potentials can depend on the modeling scheme, our supercritical water potentials may be better interpreted as near‐critical water potentials in alternate model schemes. Nonetheless, our simulations suggest that mortality could be associated with near‐critical water potentials (McDowell et al. 2008; Sperry and Love 2015) rather than exceeding a PLC threshold per se. The Gain‐Risk model predicted lethal PLC occurring before our model did by 2–4 weeks (depending on the PLC threshold) and before those crown damage observations. In addition to our model offering a renewed understanding of the evolutionary drivers underpinning stomatal behavior, we extend its practical use within ecosystem models to improve the prediction of plant function and disfunction under novel environments.

In conclusion, we develop a new, generalized optimality theory of stomatal conductance, optimizing any non‐foliar proxy that requires water and carbon, like growth, survival, and reproduction. We reconcile the computational efficiency of *instantaneous* optimization with a more biologically meaningful *dynamic feedback* optimization that better describes fitness maximization over plant lifespans by simplifying a *dynamic feedback* setup into an effectively *instantaneous* solution by incorporating the *non‐steady‐state* physics of water, carbon, and heat within a plant. Our optimal stomatal conductance compares well to observations from seedlings, saplings, and mature trees from field and greenhouse experiments, captures realistic responses to environmental cues, and predicts mortality predispositions. We advance stomatal optimality theory by incorporating generalized evolutionary fitness proxies and enhance its utility without compromising its realism, offering promise for future models to more realistically and accurately predict global carbon and water fluxes.

## Author Contributions

**Aaron Potkay:** conceptualization, formal analysis, investigation, methodology, software, visualization, writing – original draft. **Antoine Cabon:** data curation, resources, supervision, writing – review and editing. **Richard L. Peters:** data curation, resources, supervision, writing – review and editing. **Patrick Fonti:** data curation, resources, supervision, writing – review and editing. **Gerard Sapes:** data curation, resources, supervision, writing – review and editing. **Anna Sala:** data curation, resources, supervision, writing – review and editing. **Artur Stefanski:** data curation, resources, supervision, writing – review and editing. **Ethan Butler:** data curation, resources, supervision, writing – review and editing. **Raimundo Bermudez:** data curation, resources, supervision, writing – review and editing. **Rebecca Montgomery:** data curation, resources, supervision, writing – review and editing. **Peter B. Reich:** data curation, resources, supervision, writing – review and editing. **Xue Feng:** conceptualization, supervision, writing – original draft.

## Conflicts of Interest

The authors declare no conflicts of interest.

## Supporting information


Data S1.

## Data Availability

The data and code that support the findings of this study are openly available in Zenodo at https://doi.org/10.5281/zenodo.12689369.
