# Supplementary material for: Generalized Stomatal Optimization of Evolutionary Fitness Proxies for Predicting Plant Gas Exchange Under Drought, Heatwaves, and Elevated CO2
Source: Glob Chang Biol. 2025 Jan 28;31(1):e70049. doi: 10.1111/gcb.70049 (PMC11774141; doi:10.1111/gcb.70049)
Supplement: Supplementary file 1 — Data S1. [file GCB-31-e70049-s001.pdf]

Supporting Information for

Generalized stomatal optimization of evolutionary fitness proxies for predicting plant gas exchange under drought, heatwaves, and elevated CO_2

Aaron Potkay, Antoine Cabon, Richard L. Peters, Patrick Fonti, Gerard Sapes, Anna Sala, Artur Stefanski, Ethan Butler, Raimundo Bermudez, Rebecca Montgomery, Peter B. Reich, Xue Feng

Corresponding author: Aaron Potkay

Email: ajpotk@gmail.com

This PDF file includes:

Tables S1

Figures S1 to S23

Supporting text

SI References

Table S1. Symbols and meanings of model variables and parameters presented in the main text

Symbol	Unit	Meaning
		Physical variables
m_w	kg·mol^{-1}	molar mass of water (18·10^{-3} kg·mol^{-1})
ρ_w	kg·m^{-3}	density of water (1000 kg·m^{-3})
R	J·mol^{-1}·K^{-1}	universal gas constant (8.314 J·mol^{-1}·K^{-1})
ϖ	MPa·Pa^{-1}	conversion factor for converting between units of Pascals to Megapascals (10^{-6} MPa·Pa^{-1})
		Gas exchange
A_n	mol·m^{-2}·s^{-1}	leaf area-specific photosynthetic net C assimilation ($A_n = f_0(c_i - \Gamma^*)/(c_i + \gamma) - R_d$)
D_L	kPa	leaf-to-air vapor pressure deficit (VPD)
P_{atm}	kPa	atmospheric pressure
e_L'	kPa	saturated leaf vapor pressure
e_a	kPa	air vapor pressure
$g_{w,ref}$	mol·m^{-2}·s^{-1}	reference g_w at a VPD of 1 kPa
g_w	mol·m^{-2}·s^{-1}	leaf area-specific stomata conductance
RH	-	air relative humidity
E	mol·m^{-2}·s^{-1}	leaf area-specific transpiration
m	-	normalized sensitivity of g_w to VPD (average value of $(dg_w/d\ln(D_L))/g_{w,ref}$)
		Optimization
χ_w	mol·mol^{-1}	*marginal carbon cost of* water; a nonphysical quantity resulting from the optimization problem that is equivalent to the *marginal carbon profit of water* when stomata behave optimally ($\lambda \equiv \chi_w$)
x, y, z	-	mathematical shorthand (Eq. 7-9)
$\mathbb{O}$	units vary by definition; often mol·s^{-1}	general non-foliar objective being maximized over time (Eq. 1); e.g., growth, survival, and reproduction rates
λ	mol·mol^{-1}	*marginal carbon profit of water* ($\lambda = (\partial A_n/\partial g_w)/(\partial E/\partial g_w)$); the incremental change in A_n for an incremental change in E resulting solely from change in g_w that can be estimated from gas exchange measurements
		Pressure-volume
$\dot{\pi}_L$	MPa·s^{-1}	time-rate of change of π_L ($\dot{\pi}_L = d\pi_L/dt$)
RWC_L^S	m^3·m^{-3}	leaf symplastic relative water content ($\mathrm{RWC}_L^S = (\mathrm{RWC}_L^T - a_f)/(1 - a_{f,0})$)
RWC_L^T	m^3·m^{-3}	bulk or total leaf relative water content
SWC_L	kg·kg^{-1}	leaf saturated water content; mass-ratio of water to dry matter in leaf at full hydration ($\psi_L = 0$)
$a_{f,0}$	m^3·m^{-3}	leaf apoplastic fraction at full hydration ($\psi_L = 0$)
$a_{f,max}$	m^3·m^{-3}	shape parameter for Weibull function describing a_f as function of π_L; theoretical maximum value of a_f when $\pi_L = 0$
a_f	m^3·m^{-3}	leaf apoplastic fraction
$\varepsilon_{L,0}$	MPa	leaf elastic modulus at full hydration ($\psi_L = 0$)
$\varepsilon_{L,max}$	MPa	theoretical maximum elastic modulus of the symplast; approached as turgor pressure becomes very large
$\pi_{L,0,25℃}$	MPa	leaf symplastic osmotic potential at full hydration ($\psi_L = 0$) at standardized temperature of 25°C ($\pi_{L,0,25℃} = \pi_L \cdot (298.15\ \mathrm{K})/T_L^K$)
$\pi_{L,0}$	MPa	leaf symplastic osmotic potential at full hydration ($\psi_L = 0$)
$\pi_{L,tlp}$	MPa	apparent turgor loss point ($\pi_{L,tlp} = \pi_{L,0}\varepsilon_{L,0}/(\pi_{L,0} + \varepsilon_{L,0})$)

π_L	MPa	leaf symplastic osmotic potential ($\pi_L = \pi_{L,0}/\mathrm{RWC}_L^S$)
π_L^*	MPa	shape parameter for Weibull function describing a_f as function of π_L
ψ_L	MPa	leaf water potential
Δ	-	mathematical shorthand (Eq. 4)
α	mol·mol^{-1}	number of moles of carbon in a mole of the first osmolytes generated photosynthetically (e.g., triose phosphate) before sugar or starch synthesis
β	-	shape parameter for Weibull function describing a_f as function of π_L
Thermal variables		
Λ_E	J·kg^{-1}	latent heat of vaporization of water; temperature-dependent
T_L, T_L^K	°C, K	leaf temperature
T_a	°C	air temperature
c_{DM}	J·K^{-1}·kg^{-1}	thermal capacitance of leaf dry matter
c_w	J·K^{-1}·kg^{-1}	thermal capacitance of water
Photosynthesis		
Γ^*	mol·mol^{-1}	compensation point; value of c_i at which $A_n = -R_d$
K_c	mol·mol^{-1}	Michaelis-Menten constants for carboxylation
K_o	mol·mol^{-1}	Michaelis-Menten constants for oxygenation
R_d	mol·m^{-2}·s^{-1}	leaf area-specific respiration rate
$V_{c,max}$	mol·m^{-2}·s^{-1}	leaf area-specific maximum carboxylation rate
c_a	mol·mol^{-1}	air CO_2 partial pressures
c_i	mol·mol^{-1}	leaf internal CO_2 partial pressures
f_0	mol·m^{-2}·s^{-1}	theoretical maximum leaf area-specific photosynthetic gross C assimilation rate occurring at very large c_i; $f_0 = V_{c,max}$ under carboxylation-limited conditions; $f_0 = \frac{J}{4}$ under electron transport-limited conditions
o_i	mol·mol^{-1}	leaf intercellular O_2 partial pressure
J	mol·m^{-2}·s^{-1}	leaf area-specific electron transport rate
γ	mol·mol^{-1}	Michaelis-Menten constant for photosynthesis; $\gamma = K_c\left(1 + \frac{o_i}{K_o}\right)$ under carboxylation-limited conditions; $\gamma = 2\Gamma^*$ under electron transport-limited conditions

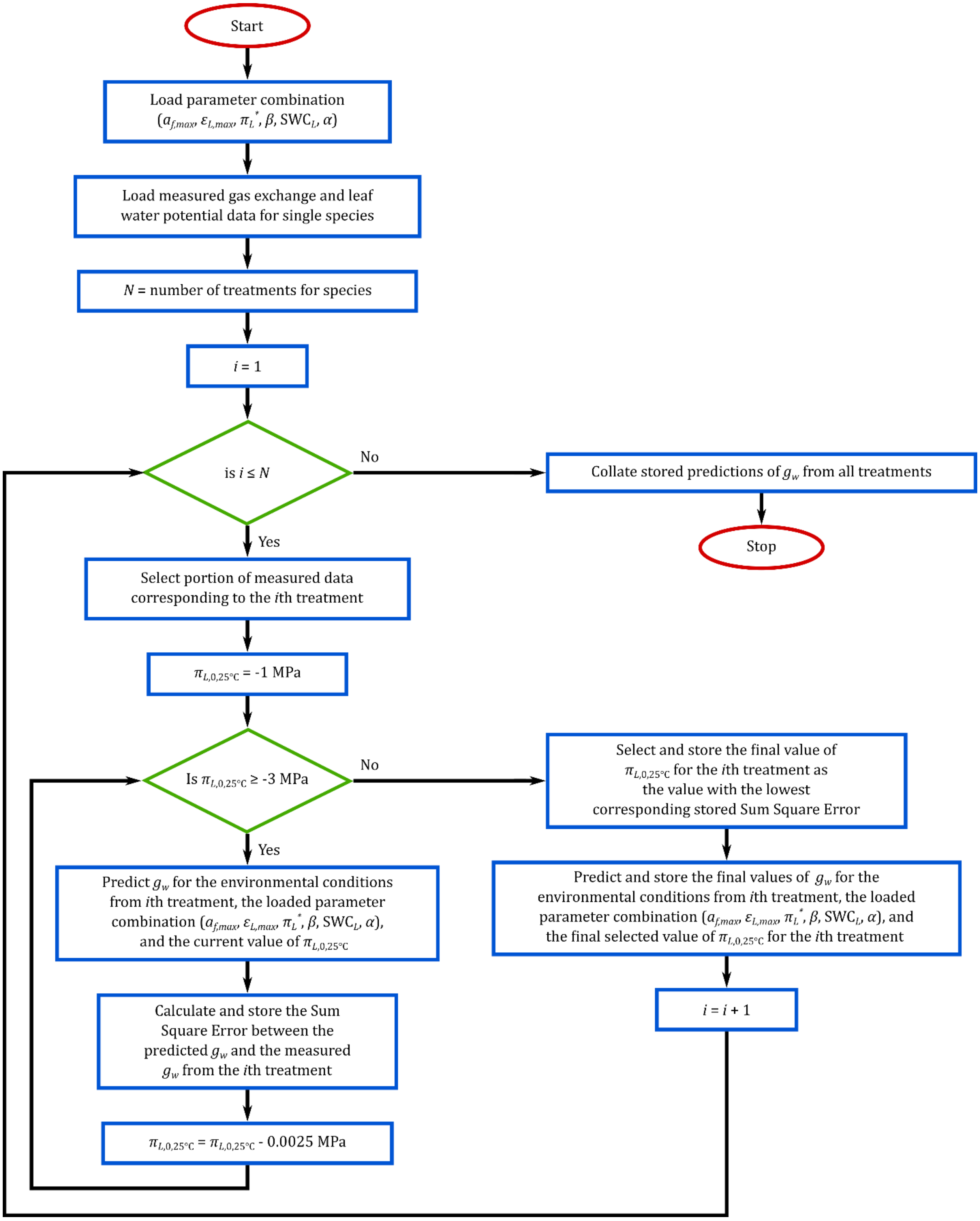

Fig. S1. Flowchart describing the algorithm used for simultaneously determining the optimal osmotic potential at full hydration at a standard temperature of 25°C, $\pi_{L,0,25°C}$, for each treatment and the stomatal conductance predictions based on those optimal $\pi_{L,0,25°C}$ for a given parameter combination in the Markov chain Monte Carlo (MCMC) method.

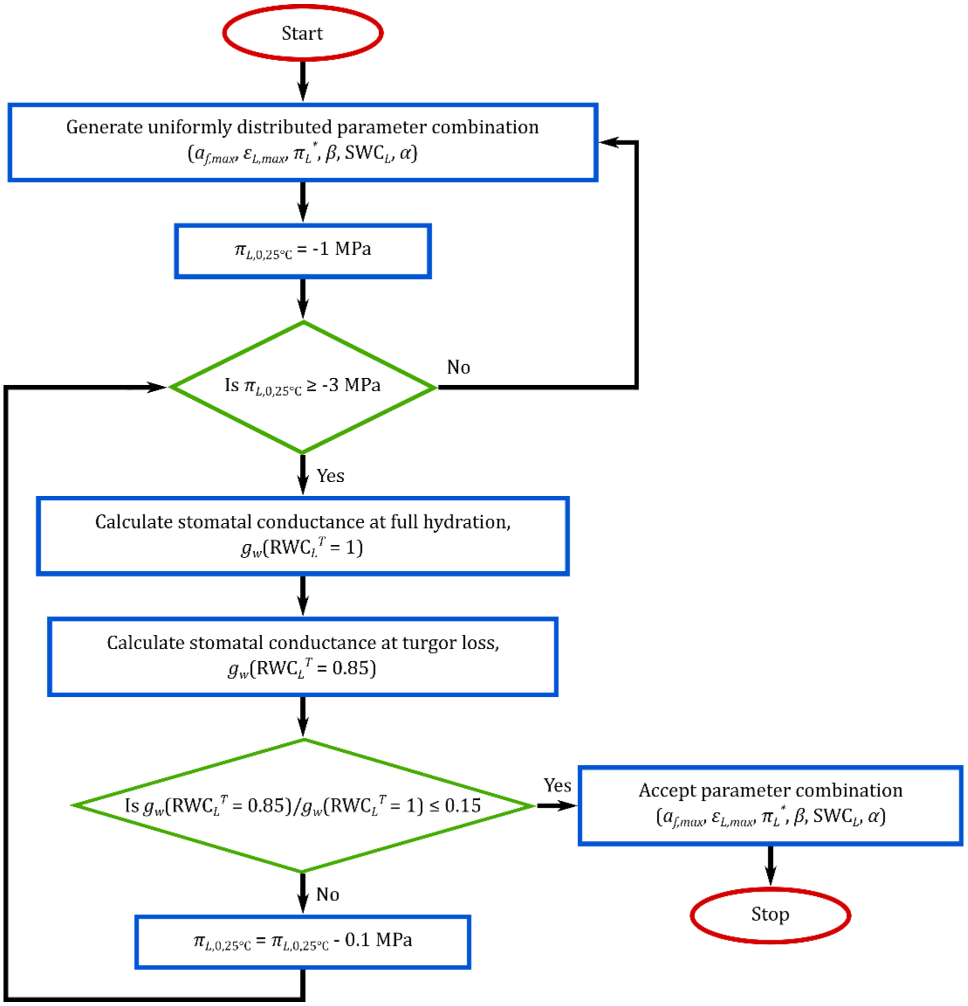

Fig. S2. Flowchart describing the algorithm used for the initialization of the prior distribution of species-specific parameters used in the Markov chain Monte Carlo (MCMC) method. Initially uniformly distributed parameter combinations were removed if they failed to result in complete or near-complete stomatal closure for any of the considered values of the osmotic potential at full hydration at a reference temperature of 25°C between -3 and -1 MPa.

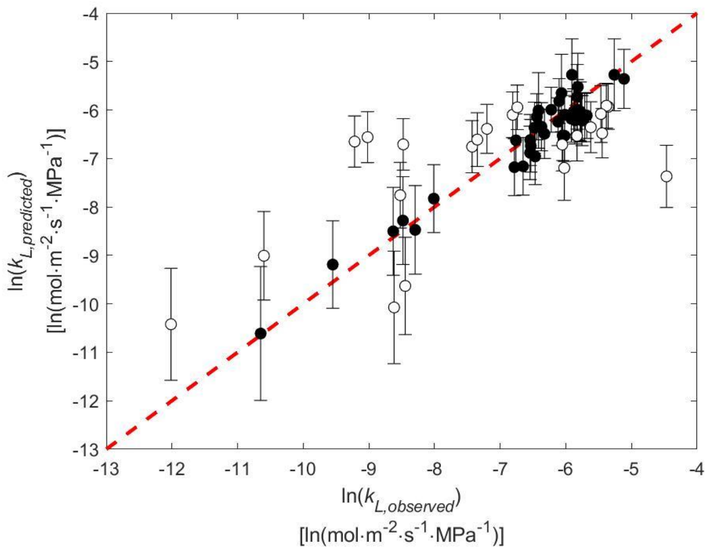

Fig. S3. Comparison of logarithmically-transformed observed and predicted leaf area-specific soil-plant conductance (k_L) for Ponderosa Pine seedlings from greenhouse experiment (Sapes & Sala, 2021). Dashed red line is the 1:1 line, black circles are predictions whose 95% confidence intervals encompass the observed value, and white circles are predictions whose 95% confidence intervals do not encompass the observed value. Predictions were made by multiple linear regression (Eq. S7.3.2). 95% confidence intervals for predicted log(k_L) were calculated by propagation of errors from the 95% confidence intervals of the regressed parameters.

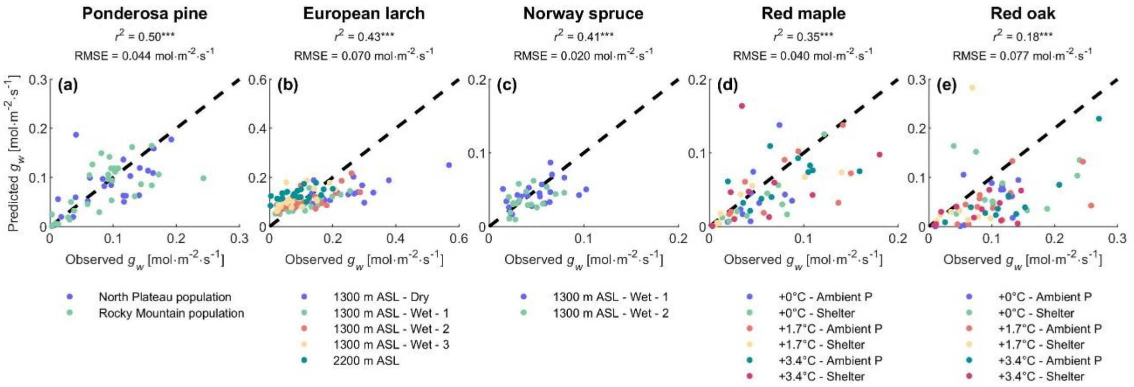

Figure S4. Comparison of observed and predicted stomatal conductance (g_w) for Ponderosa pine (a), European larch (b), Norway spruce (c), Red maple (d), and Red oak (e). Different colors reflect different treatments. Dashed black line is the 1:1 line. Predictions apply the best parameters from the MCMC (Fig. S5-S9) and the corresponding osmotic potential at full hydration at a standard temperature of 25°C ($\pi_{L,0,25℃}$; Fig. S10) for the respective treatment. The coefficient of determination is denoted by r^2, the root mean square error is denoted by RMSE, and stars indicate significance of the relationship (* for $p < 0.05$, ** for $p < 0.01$, *** for $p < 0.001$).

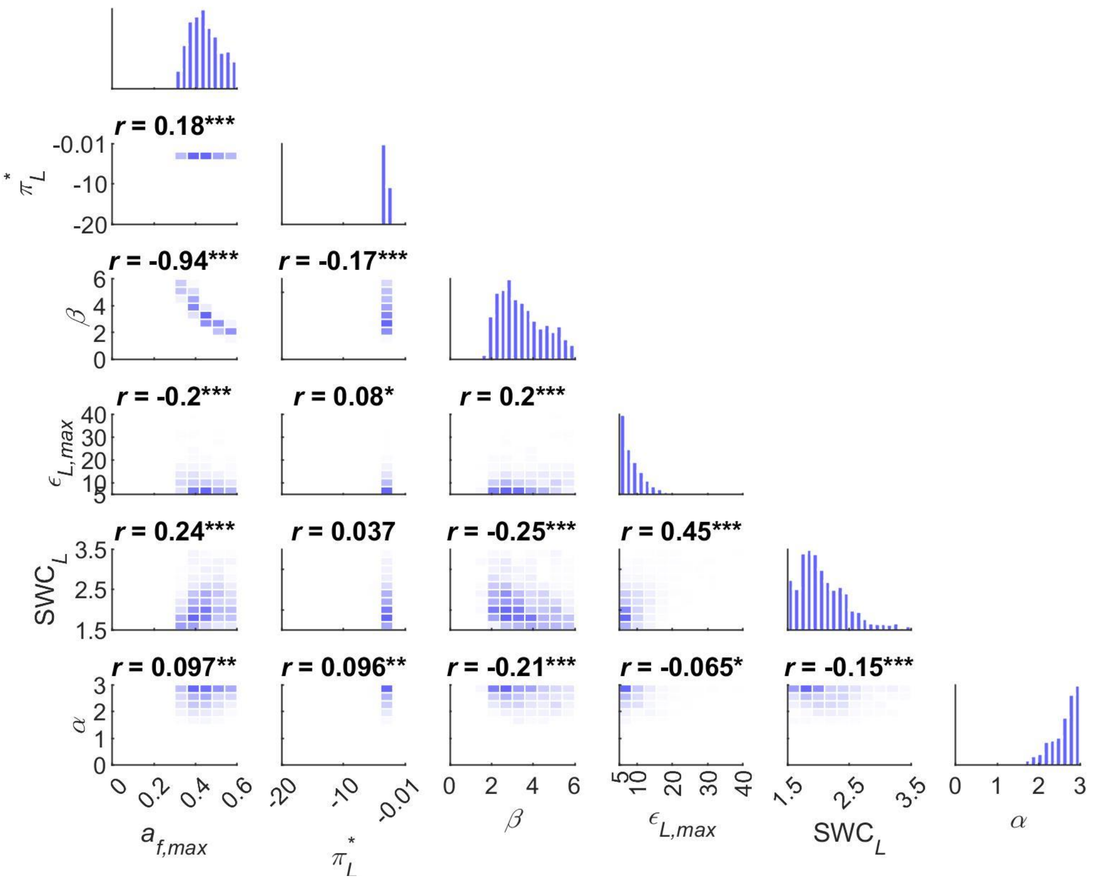

Fig. S5. MCMC results for Ponderosa pine seedlings from the greenhouse experiment. Plots along the diagonal show posterior distributions of the best 1000 fitting parameters that resulted in the lowest SSE between measured and predicted stomatal conductance. Plots off the diagonal show the covariation of each pair of the best 1000 fitting parameters. The opacity reflects the number of pairs with each bin. The axes limits represent the ranges of the parameters' prior distributions. The Pearson correlation coefficient is denoted by *r*, and stars indicate significance of the relationship (* for $p < 0.05$, ** for $p < 0.01$, *** for $p < 0.001$).

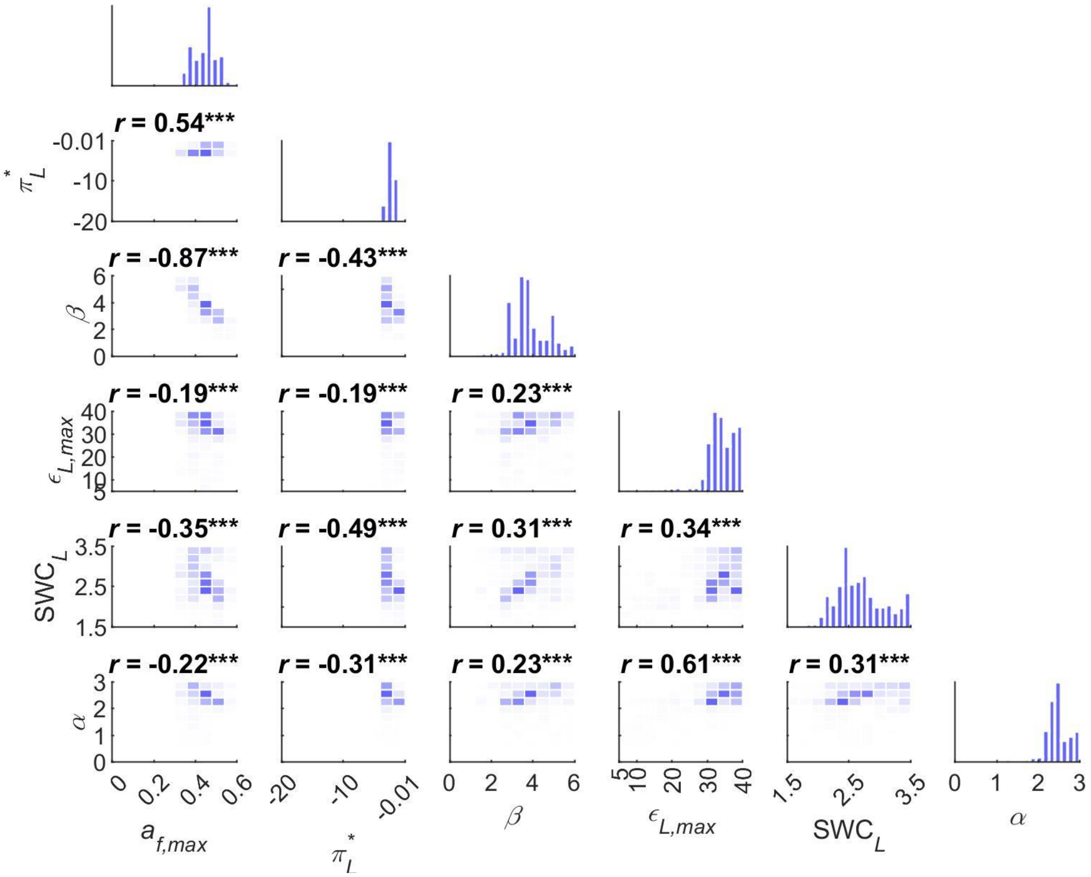

Fig. S6. MCMC results for mature European larch trees from Lötschental sites. Plots along the diagonal show posterior distributions of the best 1000 fitting parameters that resulted in the lowest SSE between measured and predicted stomatal conductance. Plots off the diagonal show the covariation of each pair of the best 1000 fitting parameters. The opacity reflects the number of pairs with each bin. The axes limits represent the ranges of the parameters' prior distributions. The Pearson correlation coefficient is denoted by *r*, and stars indicate significance of the relationship (* for $p < 0.05$, ** for $p < 0.01$, *** for $p < 0.001$).

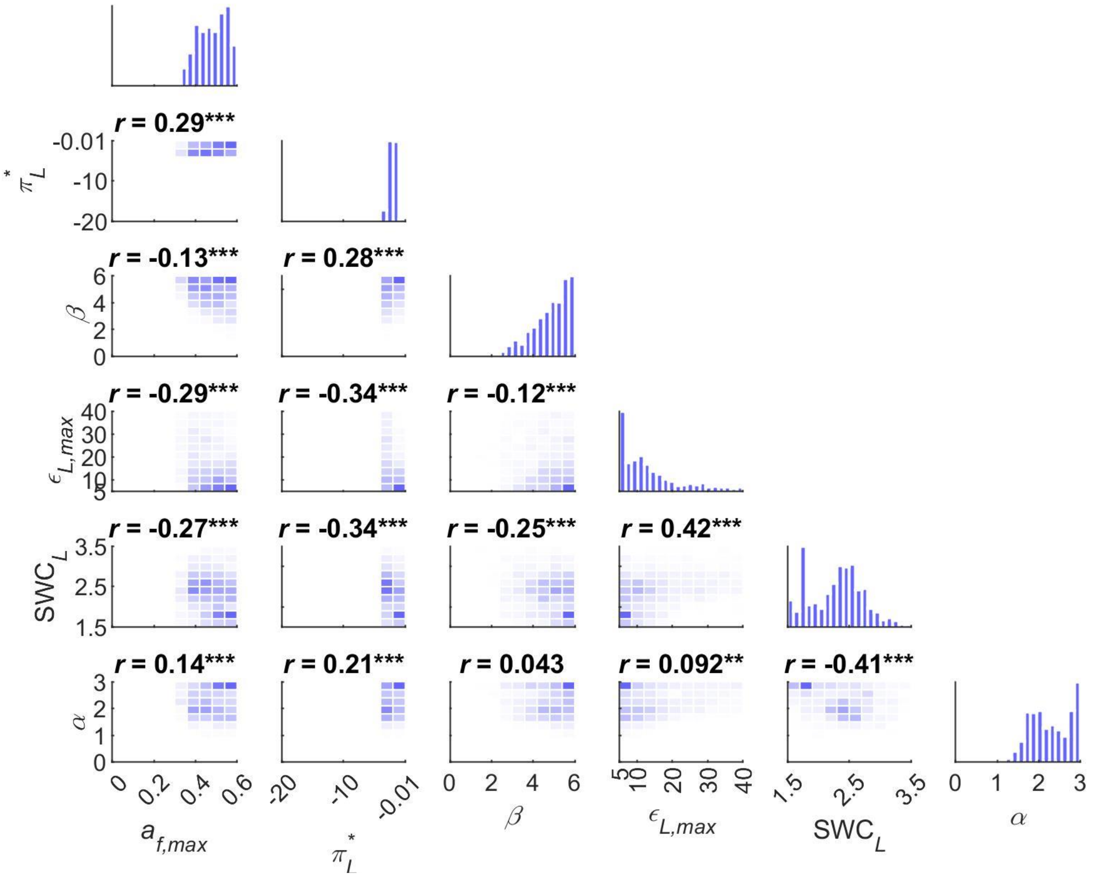

Fig. S7. MCMC results for mature Norway spruce trees from Lötschental sites. Plots along the diagonal show posterior distributions of the best 1000 fitting parameters that resulted in the lowest SSE between measured and predicted stomatal conductance. Plots off the diagonal show the covariation of each pair of the best 1000 fitting parameters. The opacity reflects the number of pairs with each bin. The axes limits represent the ranges of the parameters' prior distributions. The Pearson correlation coefficient is denoted by *r*, and stars indicate significance of the relationship (* for $p < 0.05$, ** for $p < 0.01$, *** for $p < 0.001$).

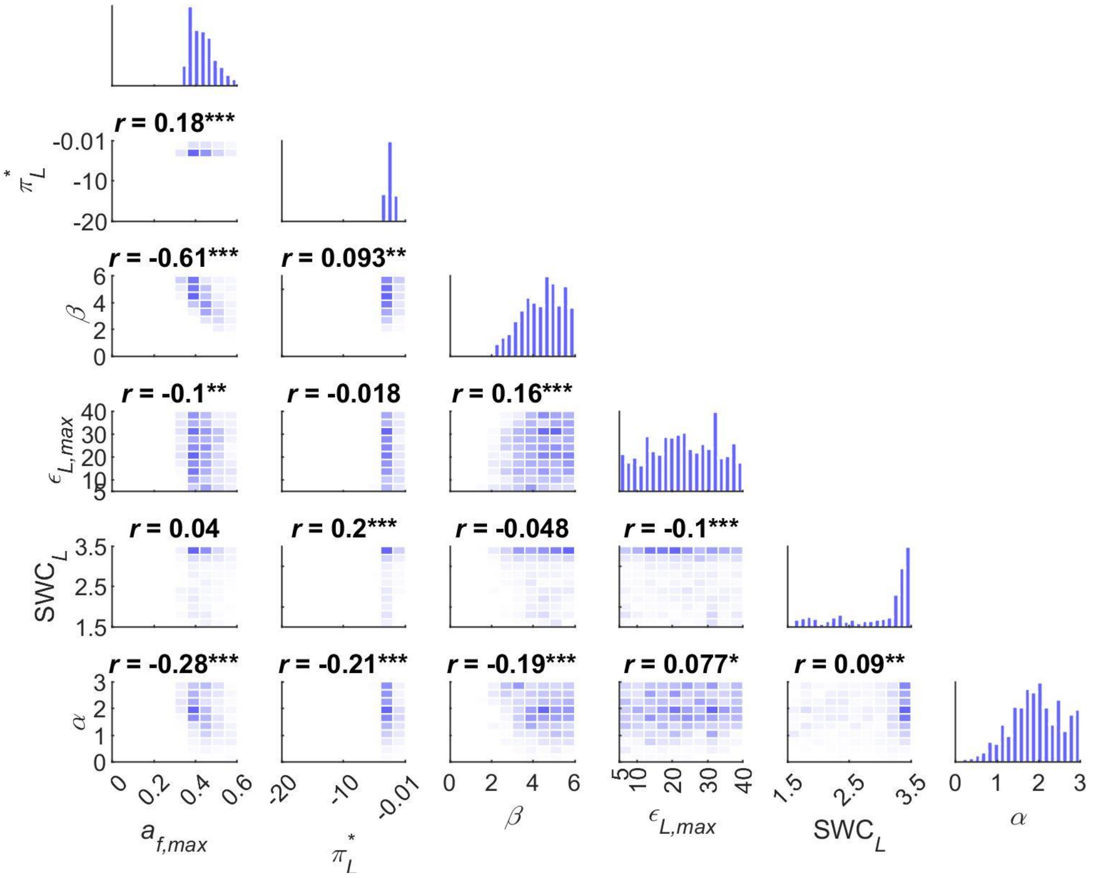

Fig. S8. MCMC results for Red maple saplings from B4Warmed sites. Plots along the diagonal show posterior distributions of the best 1000 fitting parameters that resulted in the lowest SSE between measured and predicted stomatal conductance. Plots off the diagonal show the covariation of each pair of the best 1000 fitting parameters. The opacity reflects the number of pairs with each bin. The axes limits represent the ranges of the parameters' prior distributions. The Pearson correlation coefficient is denoted by *r*, and stars indicate significance of the relationship (* for $p < 0.05$, ** for $p < 0.01$, *** for $p < 0.001$).

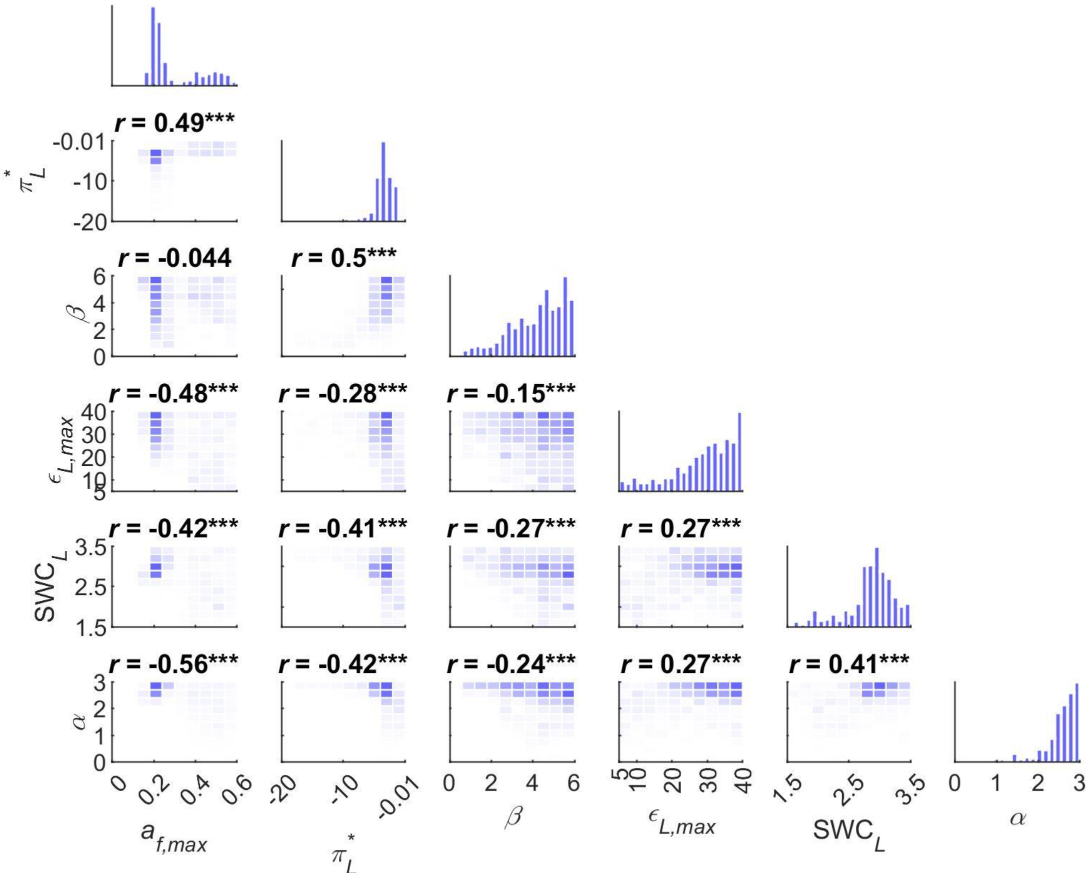

Fig. S9. MCMC results for Red oak saplings from B4Warmed sites. Plots along the diagonal show posterior distributions of the best 1000 fitting parameters that resulted in the lowest SSE between measured and predicted stomatal conductance. Plots off the diagonal show the covariation of each pair of the best 1000 fitting parameters. The opacity reflects the number of pairs with each bin. The axes limits represent the ranges of the parameters' prior distributions. The Pearson correlation coefficient is denoted by *r*, and stars indicate significance of the relationship (* for $p < 0.05$, ** for $p < 0.01$, *** for $p < 0.001$).

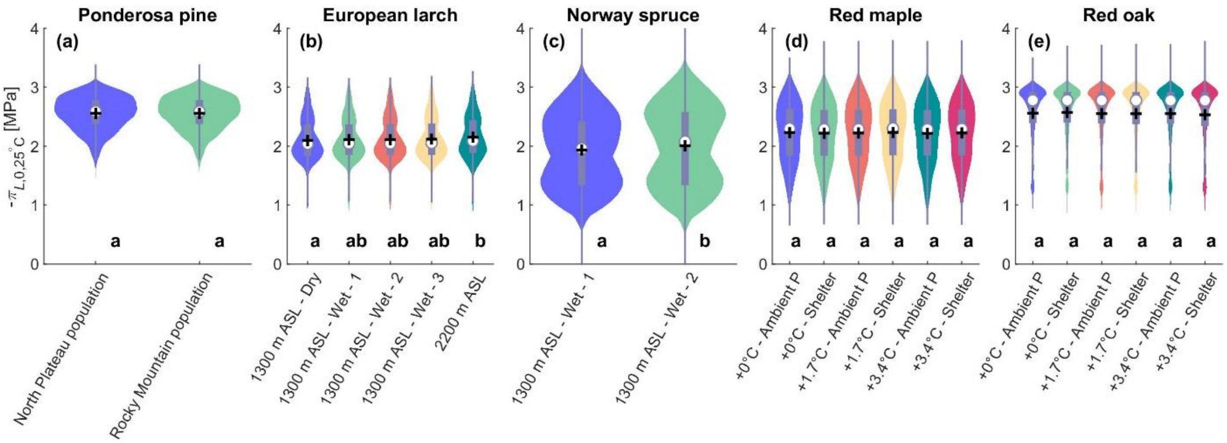

Fig. S10. Variation in osmotic potential at full hydration at a standard temperature of 25°C, $\pi_{L,0,25°C}$, among treatments for Ponderosa pine (a), European larch (b), Norway spruce (c), Red maple (d), and Red oak (e). For each treatment, violin plots show the distribution of $\pi_{L,0,25°C}$ associated with the best 1000 fitting parameters that resulted in the lowest SSE between measured and predicted stomatal conductance. Means, medians, interquartile ranges, and Tukey's fences are denoted by black crosses, white circles, thick grey lines, and thin grey lines. Bold letters indicate significant differences between treatments according to an ANOVA test.

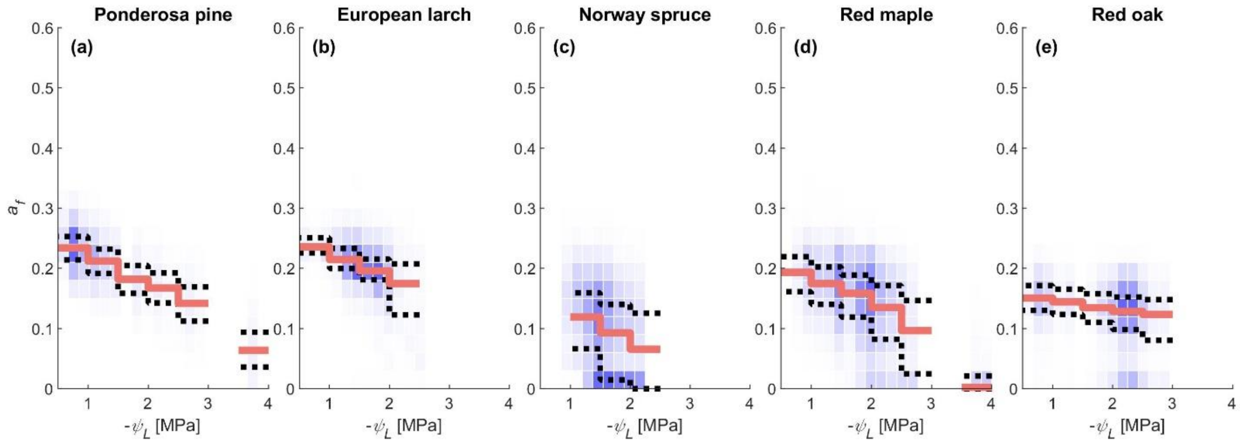

Fig. S11. Negative effect of hydraulic stress, quantified by leaf water potential, ψ_L, on apoplastic fraction, a_f, for Ponderosa pine (a), European larch (b), Norway spruce (c), Red maple (d), and Red oak (e). From the best 1000 fitting parameters that resulted in the lowest SSE between measured and predicted stomatal conductance (Fig. S5-S9) and the corresponding osmotic potential at full hydration at a standard temperature of 25°C averaged across treatments (Fig. S11), we estimated a range of apoplastic fractions for each measured leaf water potential values. The opacity of grids reflects the number of predictions of a_f within the grids. Solid pink line denotes the median apoplastic fraction within bins separated by leaf water potential in increments of 0.5 MPa. Dashed black lines denote the 25th and 75th percentiles of the estimated apoplastic fractions within bins separated by leaf water potential in increments of 0.5 MPa. Limits of *y*-axis reflects range of the prior distribution of our parameter for the maximum apoplastic fraction occurring at an osmotic potential of zero (Eq. S4.1.2), $a_{f,max}$.

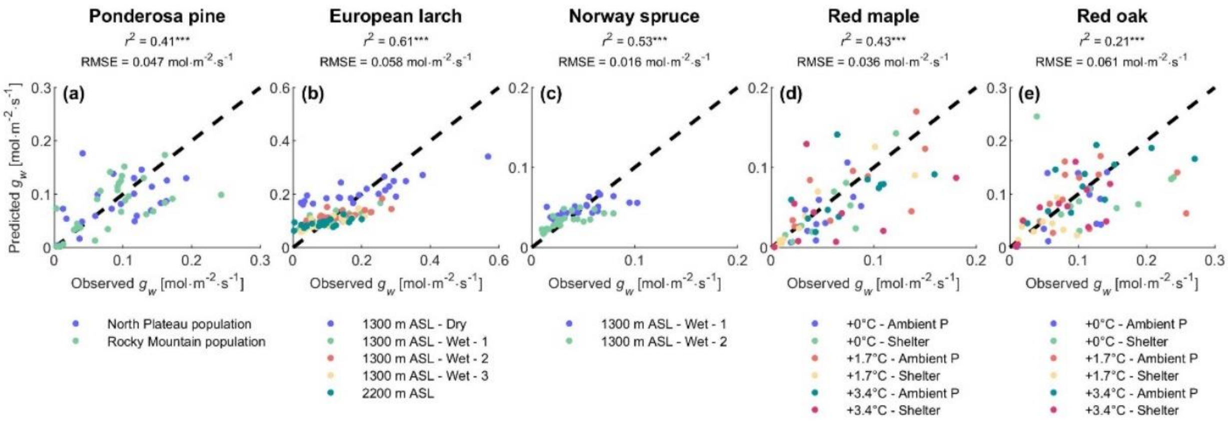

Fig. S12. Comparison of observed and predicted stomatal conductance for Ponderosa pine (a), European larch (b), Norway spruce (c), Red maple (d), and Red oak (e) using the widely used USO model with constant g_1 within each treatment (Section S6.4). Different colors reflect different treatments. Dashed black line is the 1:1 line.

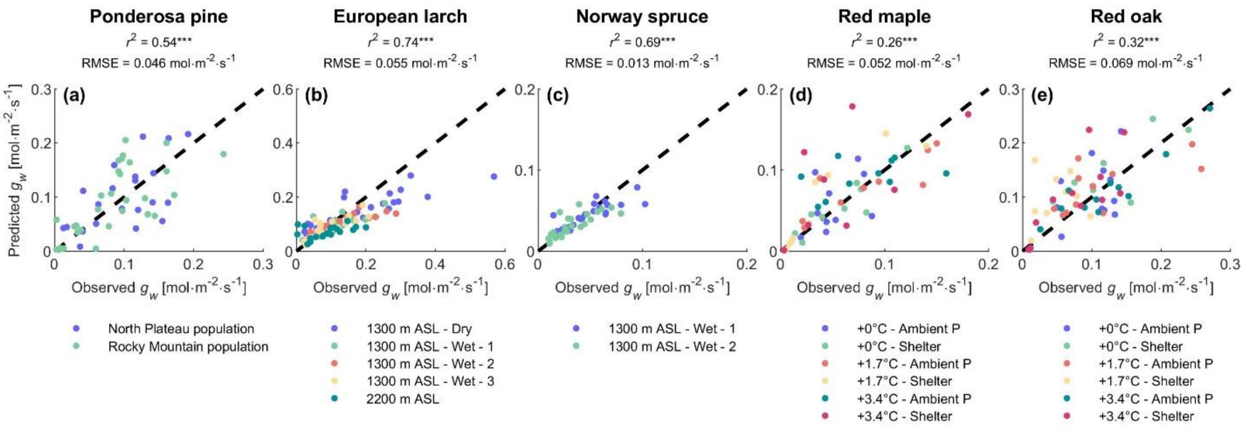

Fig. S13. Comparison of observed and predicted stomatal conductance for Ponderosa pine (a), European larch (b), Norway spruce (c), Red maple (d), and Red oak (e) using the widely used USO model with variations in the g_1 parameter predicted by linear mixed effects models (Section S6.4). Different colors reflect different treatments. Dashed black line is the 1:1 line.

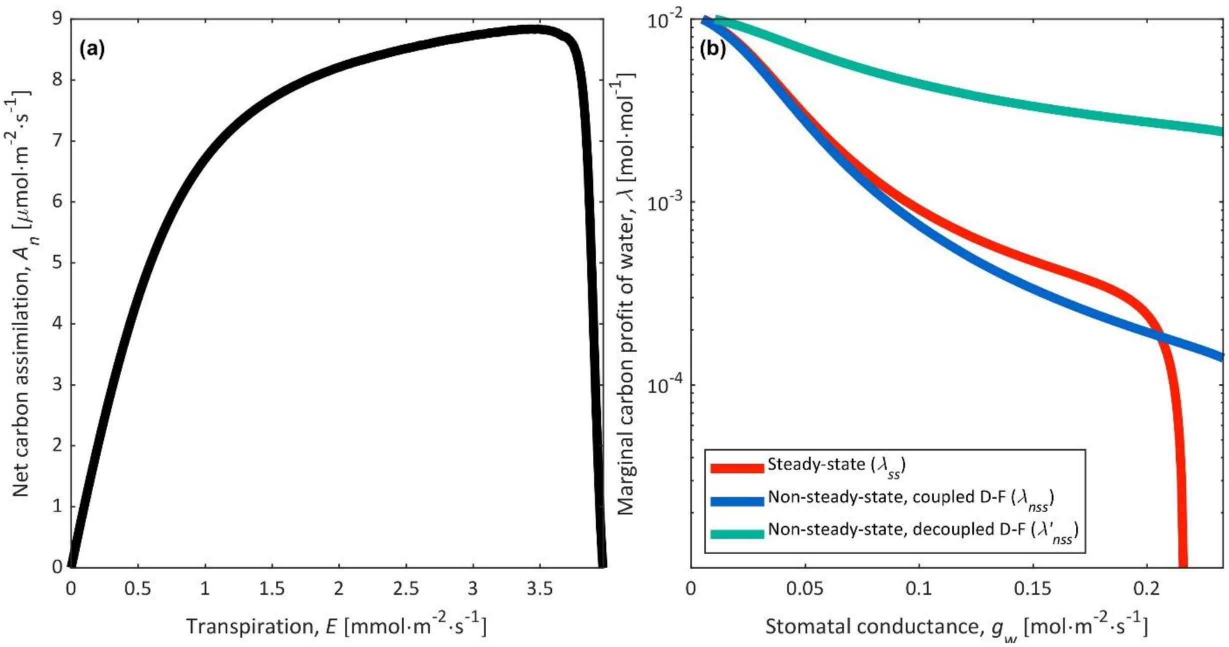

Fig. S14. Comparison of steady-state and non-steady-state values of the *marginal carbon profit of water*, derived in Section S3.1 and S3.2, for the same net carbon assimilation-transpiration curve. In (a), net carbon assimilation (A_n) was simulated for values of transpiration (E) using the steady-state leaf gas exchange model described by Potkay and Feng (2023a,b) using their parameters for Scots pine. In (a), A_n declines at high E due to declines in maximum photosynthetic capacities associated with increasingly more negative leaf water potentials (Potkay & Feng, 2023b). Simulations were performed at CO_2 and O_2 partial pressures of 410 and 207 mmol·mol^{-1}, respectively, T_a = 25 °C, RH = 0.4, and incoming irradiance of 600 W·m^{-2}. In (b), three estimates of the *marginal carbon profit of water* were calculated for the A_n-E curve shown in (a), including the steady-state *marginal profit* (λ_{ss}; Eq. S3.1.8), the non-steady-state *marginal profit* with coupled CO_2 diffusion and biological carbon fixation (λ_{nss}; Eq. S3.1.9), and the non-steady-state *marginal profit* with decoupled CO_2 diffusion and biological carbon fixation (λ'_{nss}; Eq. S3.2.3).

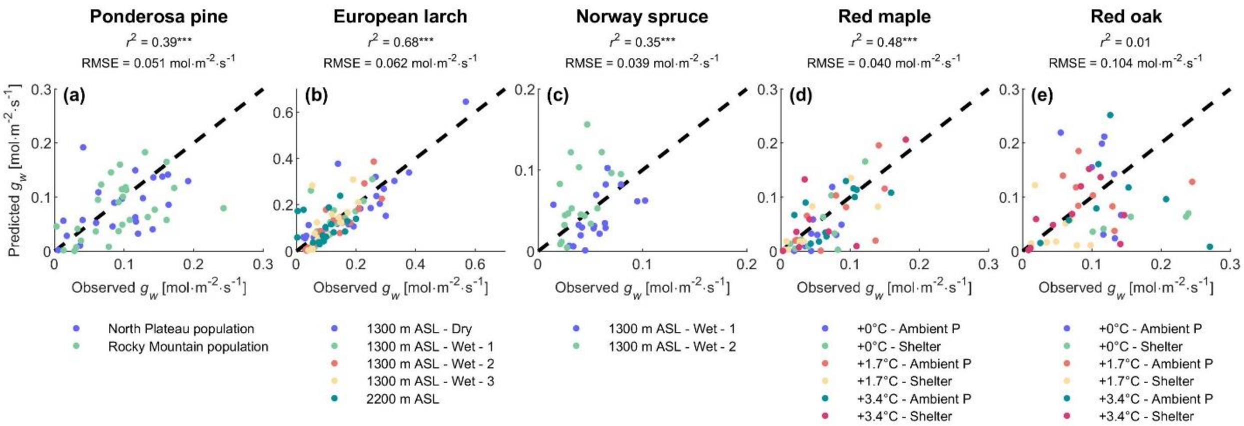

Fig. S15. Comparison of observed and predicted stomatal conductance using the best parameters from the MCMC for Ponderosa pine (a), European larch (b), Norway spruce (c), Red maple (d), and Red oak (e) evaluated with decoupled CO_2 diffusion and biological carbon fixation (Section S3.2) through the non-steady-state *marginal profit* with decoupled CO_2 diffusion and biological carbon fixation (λ'_{nss}; Eq. S3.2.3) instead of the non-steady-state *marginal profit* with coupled CO_2 diffusion and biological carbon fixation (λ_{nss}; Eq. S3.1.9). Different colors reflect different treatments. Dashed black line is the 1:1 line.

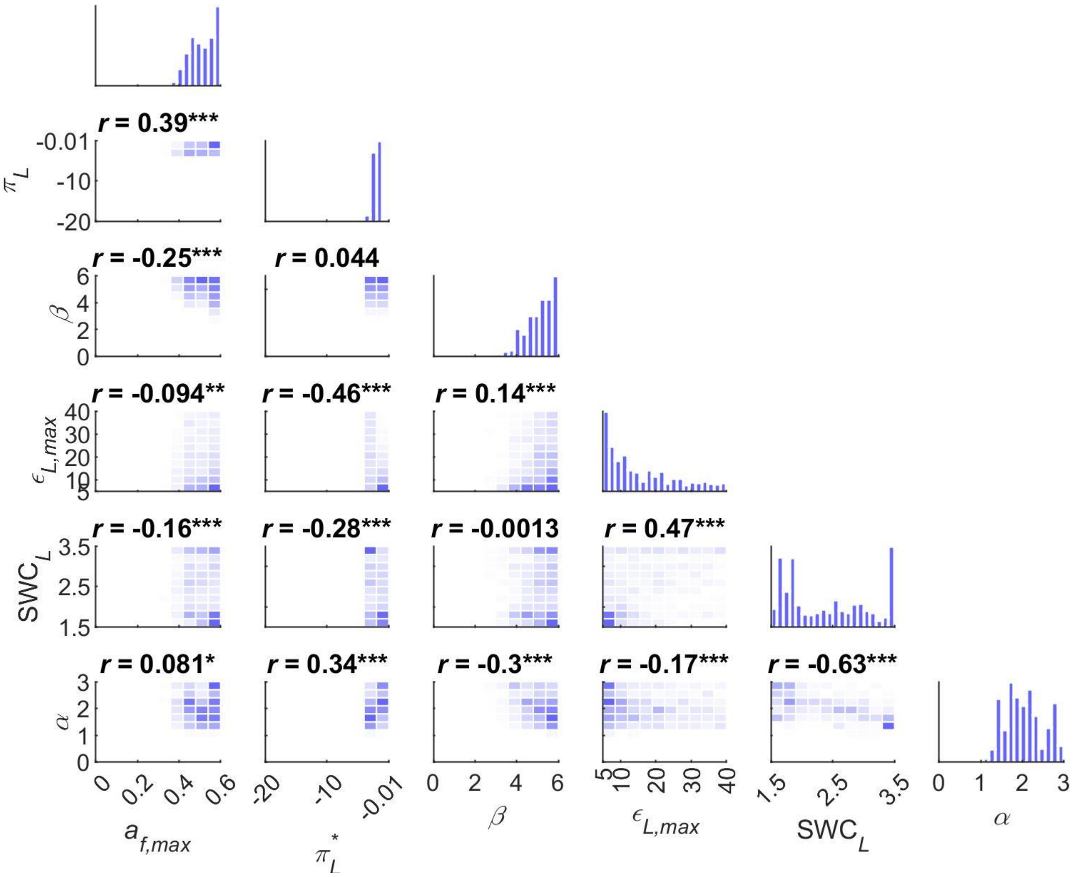

Fig. S16. MCMC results for Ponderosa pine seedlings from the greenhouse experiment evaluated with decoupled CO_2 diffusion and biological carbon fixation (Section S3.2) through the non-steady-state *marginal profit* with decoupled CO_2 diffusion and biological carbon fixation (λ'_{nss}; Eq. S3.2.3) instead of the non-steady-state *marginal profit* with coupled CO_2 diffusion and biological carbon fixation (λ_{nss}; Eq. S3.1.9). Plots along the diagonal show posterior distributions of the best 1000 fitting parameters that resulted in the lowest SSE between measured and predicted stomatal conductance. Plots off the diagonal show the covariation of each pair of the best 1000 fitting parameters. The opacity reflects the number of pairs with each bin. The axes limits represent the ranges of the parameters' prior distributions. The Pearson correlation coefficient is denoted by *r*, and stars indicate significance of the relationship (* for $p < 0.05$, ** for $p < 0.01$, *** for $p < 0.001$).

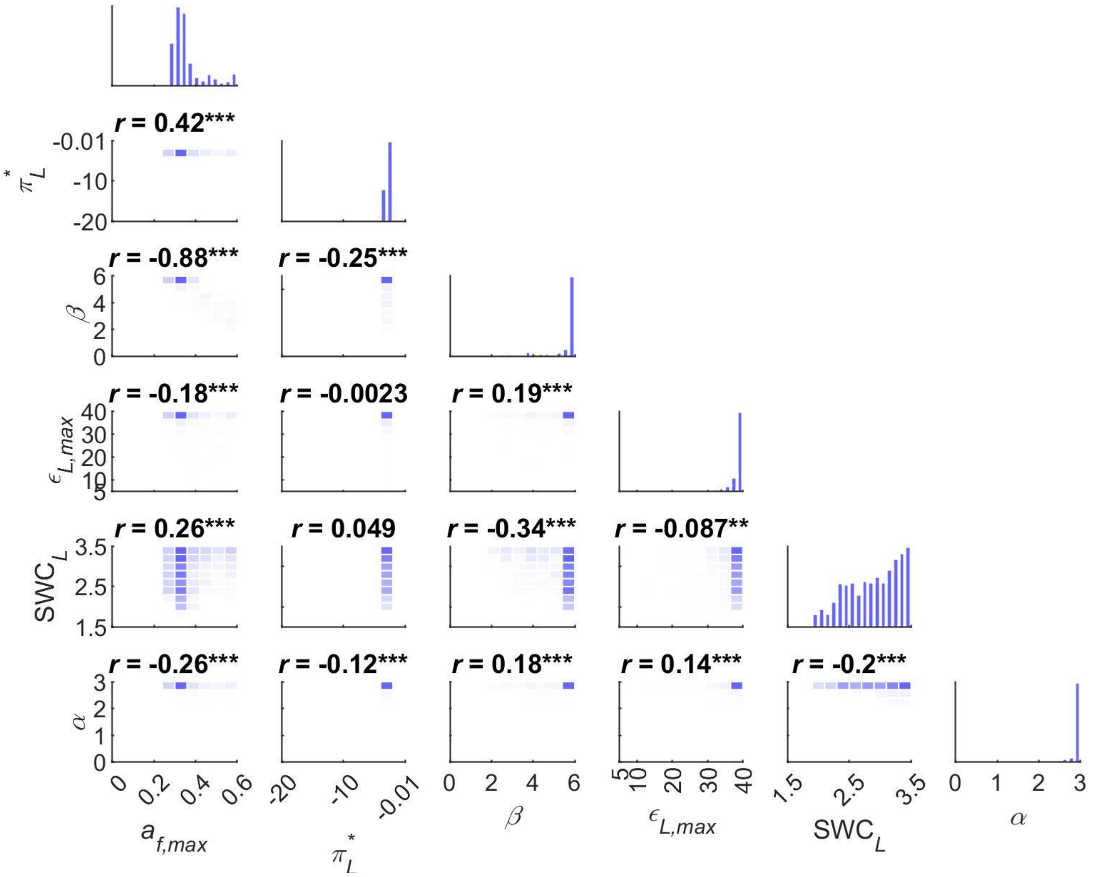

Fig. S17. MCMC results for mature European larch trees from Lötschental sites evaluated with decoupled CO_2 diffusion and biological carbon fixation (Section S3.2) through the non-steady-state *marginal profit* with decoupled CO_2 diffusion and biological carbon fixation (λ'_{nss}; Eq. S3.2.3) instead of the non-steady-state *marginal profit* with coupled CO_2 diffusion and biological carbon fixation (λ_{nss}; Eq. S3.1.9). Plots along the diagonal show posterior distributions of the best 1000 fitting parameters that resulted in the lowest SSE between measured and predicted stomatal conductance. Plots off the diagonal show the covariation of each pair of the best 1000 fitting parameters. The opacity reflects the number of pairs with each bin. The axes limits represent the ranges of the parameters' prior distributions. The Pearson correlation coefficient is denoted by *r*, and stars indicate significance of the relationship (* for $p < 0.05$, ** for $p < 0.01$, *** for $p < 0.001$).

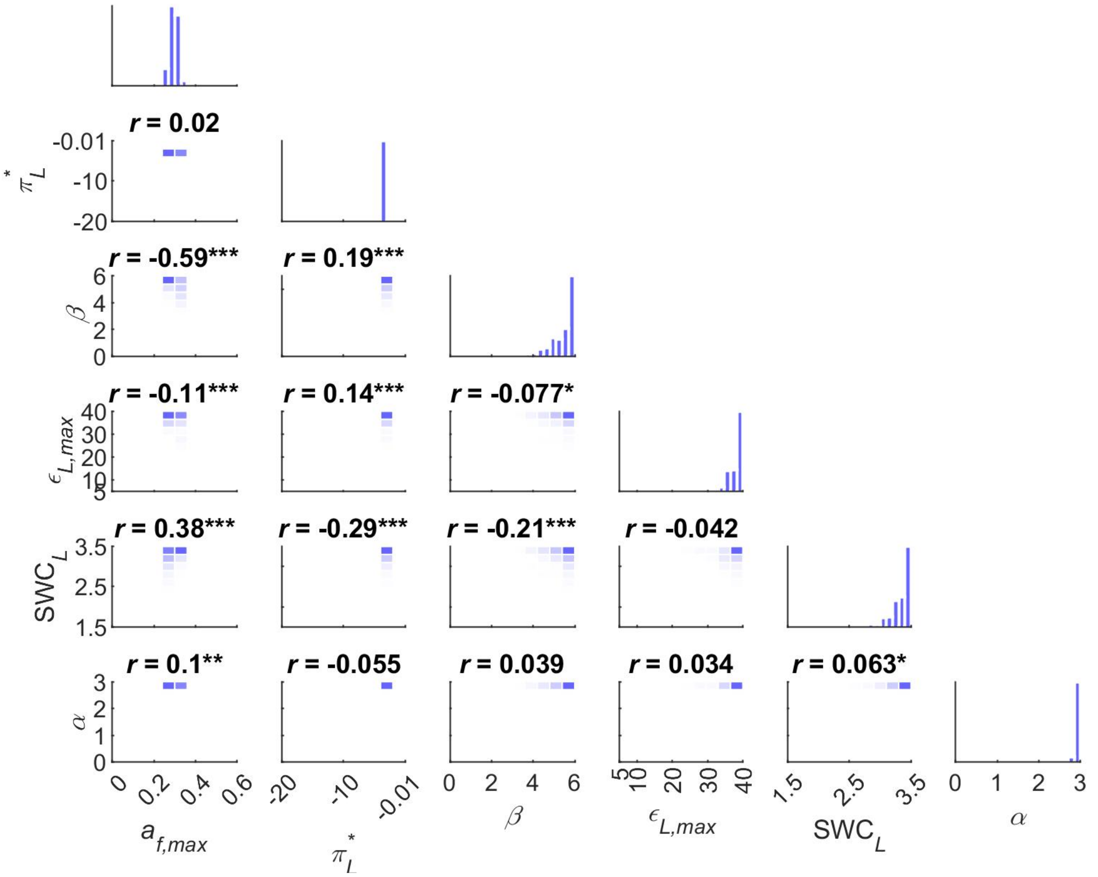

Fig. S18. MCMC results for mature Norway spruce trees from Lötschental sites evaluated with decoupled CO_2 diffusion and biological carbon fixation (Section S3.2) through the non-steady-state *marginal profit* with decoupled CO_2 diffusion and biological carbon fixation (λ'_{nss}; Eq. S3.2.3) instead of the non-steady-state *marginal profit* with coupled CO_2 diffusion and biological carbon fixation (λ_{nss}; Eq. S3.1.9). Plots along the diagonal show posterior distributions of the best 1000 fitting parameters that resulted in the lowest SSE between measured and predicted stomatal conductance. Plots off the diagonal show the covariation of each pair of the best 1000 fitting parameters. The opacity reflects the number of pairs with each bin. The axes limits represent the ranges of the parameters' prior distributions. The Pearson correlation coefficient is denoted by *r*, and stars indicate significance of the relationship (* for $p < 0.05$, ** for $p < 0.01$, *** for $p < 0.001$).

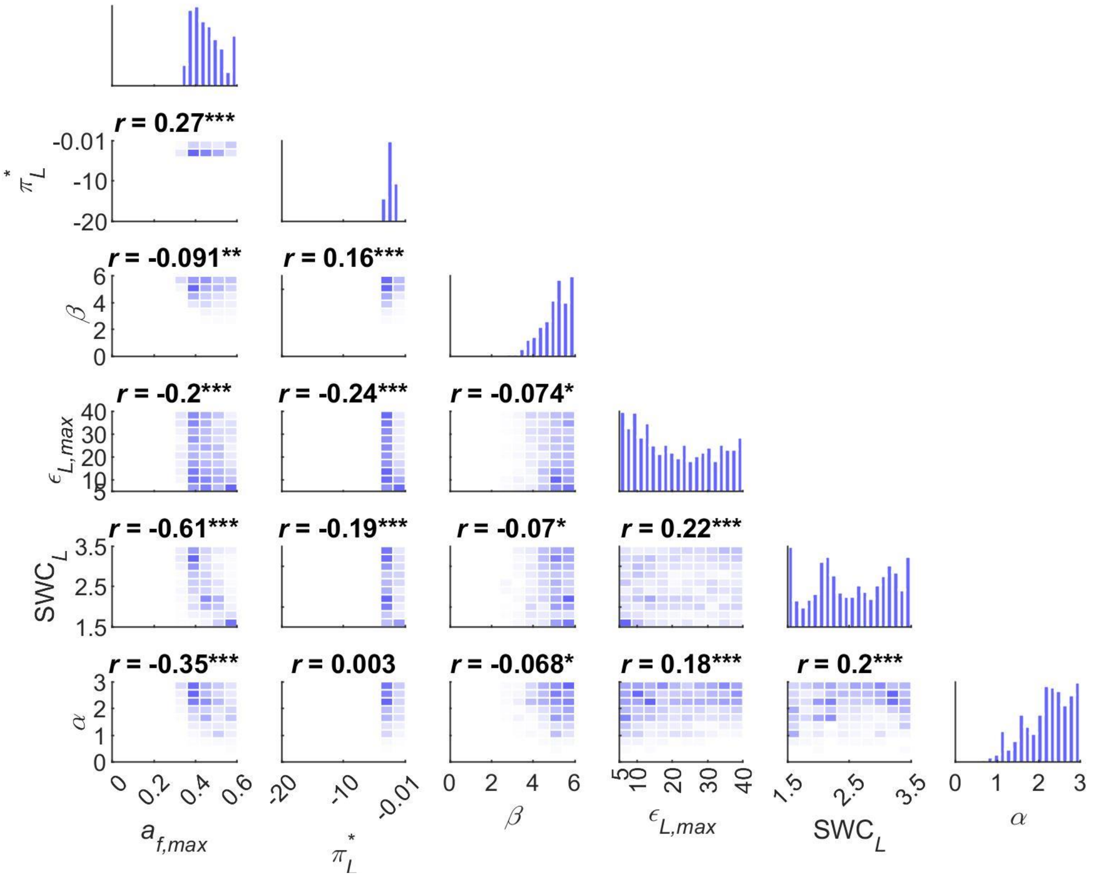

Fig. S19. MCMC results for Red maple saplings from B4Warmed sites evaluated with decoupled CO_2 diffusion and biological carbon fixation (Section S3.2) through the non-steady-state *marginal profit* with decoupled CO_2 diffusion and biological carbon fixation (λ'_{nss}; Eq. S3.2.3) instead of the non-steady-state *marginal profit* with coupled CO_2 diffusion and biological carbon fixation (λ_{nss}; Eq. S3.1.9). Plots along the diagonal show posterior distributions of the best 1000 fitting parameters that resulted in the lowest SSE between measured and predicted stomatal conductance. Plots off the diagonal show the covariation of each pair of the best 1000 fitting parameters. The opacity reflects the number of pairs with each bin. The axes limits represent the ranges of the parameters' prior distributions. The Pearson correlation coefficient is denoted by *r*, and stars indicate significance of the relationship (* for $p < 0.05$, ** for $p < 0.01$, *** for $p < 0.001$).

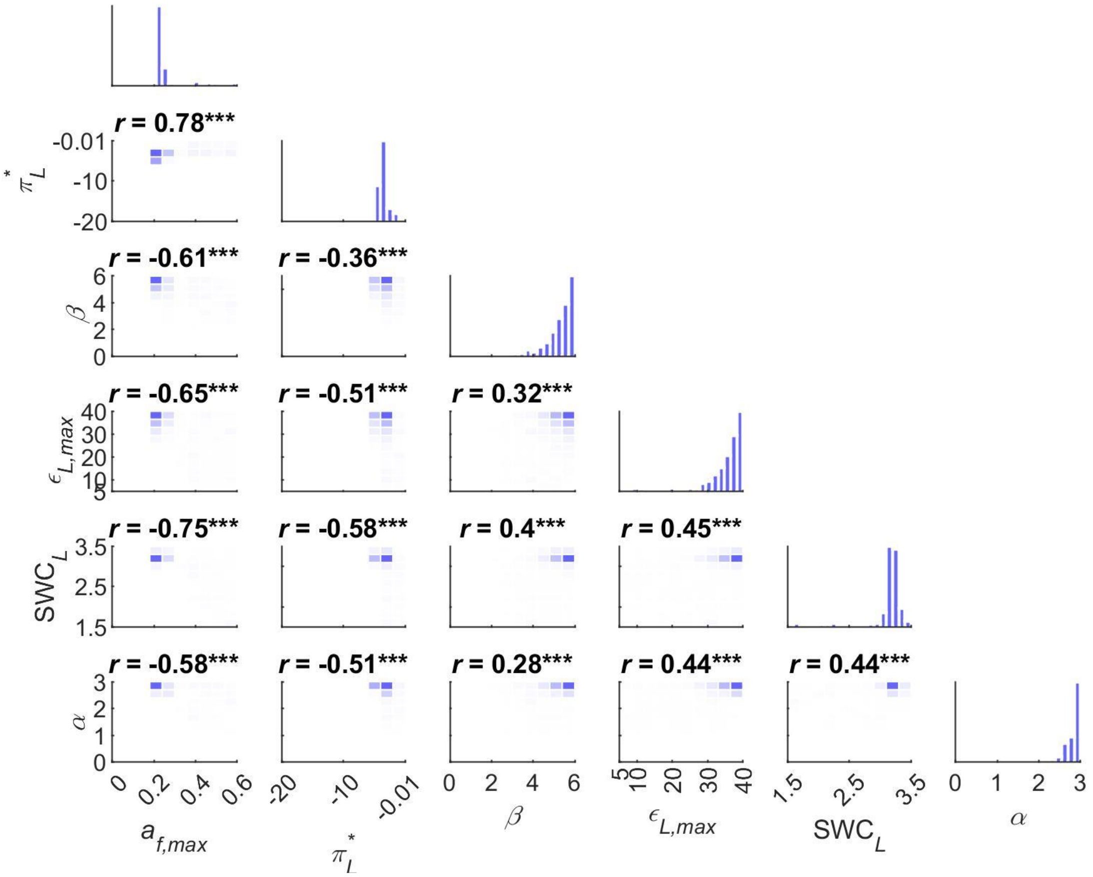

Fig. S20. MCMC results for Red oak saplings from B4Warmed sites evaluated with decoupled CO_2 diffusion and biological carbon fixation (Section S3.2) through the non-steady-state *marginal profit* with decoupled CO_2 diffusion and biological carbon fixation (λ'_{nss}; Eq. S3.2.3) instead of the non-steady-state *marginal profit* with coupled CO_2 diffusion and biological carbon fixation (λ_{nss}; Eq. S3.1.9). Plots along the diagonal show posterior distributions of the best 1000 fitting parameters that resulted in the lowest SSE between measured and predicted stomatal conductance. Plots off the diagonal show the covariation of each pair of the best 1000 fitting parameters. The opacity reflects the number of pairs with each bin. The axes limits represent the ranges of the parameters' prior distributions. The Pearson correlation coefficient is denoted by *r*, and stars indicate significance of the relationship (* for $p < 0.05$, ** for $p < 0.01$, *** for $p < 0.001$).

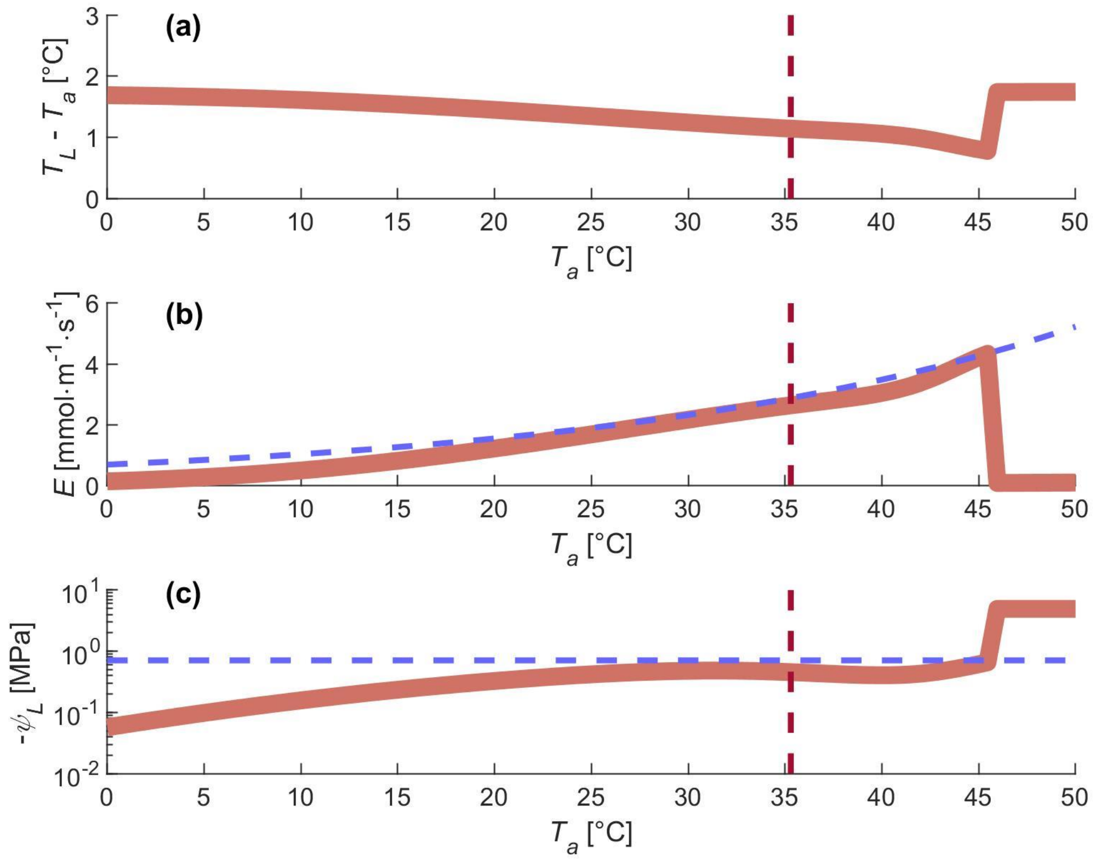

Fig. S21. Leaf-to-air temperature difference (a), transpiration (b), and leaf water potential (c) in simulation for Ponderosa pine with VPD varied by changing air temperature and constant relative humidity (Fig. 3). The red vertical dashed line denotes the air temperature at which the corresponding leaf temperature equals the thermal optimum for photosynthetic capacities (Section S7.2). In (b), the blue dashed line is the critical transpiration rate, E_{crit}, evaluated as the maximum possible value of transpiration (Sperry et al., 1998). In (c), the blue dashed line is the critical leaf water potential, $\psi_{L,crit}$, evaluated as the leaf water potential at which the transpiration rate equals E_{crit} (Sperry et al., 1998).

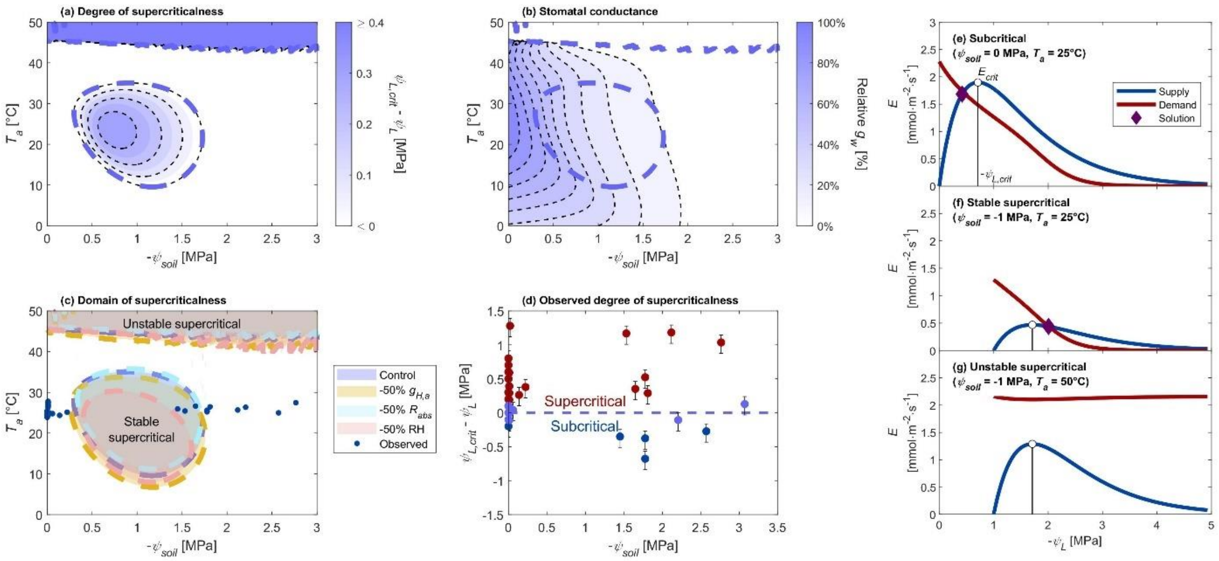

Fig. S22. Simulated degree of supercriticalness ($\psi_{L,crit} - \psi_L$) for Ponderosa Pine seedlings under baseline control conditions (same as those in Fig. 3 & Fig. S21; Section S7.2) and varied air temperature (T_a) and soil water potential (ψ_{soil}) (a), corresponding simulated stomatal conductance (g_w) relative to its maximum value simulated for the range of air temperatures and soil water potentials (b), regions of simulated supercriticalness under baseline control conditions and under varied conditions with either the boundary layer conductance to heat ($g_{H,a}$), absorbed shortwave radiation (R_{abs}), or relative humidity (RH) reduced by 50% (c), observed degree of supercriticalness ($\psi_{L,crit} - \psi_L$) for Ponderosa Pine seedlings (Section S7.3) (d), and simulated *steady-state* supply and demand transpiration (E) curves under *subcritical* (e), *stable supercritical* (f), and *unstable supercritical* (g) regimes. In (a-c), dashed purple lines encompass *supercritical* regions. In (a-b), thin dashed black lines are contour lines in increments of 0.1 MPa (a) and 10% (b). In (c), measurements of leaf temperature and soil water potential from the greenhouse experiment (Sapes & Sala, 2021) are plotted. In (d), critical leaf water potentials ($\psi_{L,crit}$) are estimated from the measured soil water potential and the regressed parameter that controls the decline of hydraulic conductance under water-stress (S_k in Eq. S7.2.5) (Section S7.3). In (d), 95% confidence intervals for critical water potentials are based on the 95% confidence intervals for S_k. In (e-g), supply transpiration curves are determined by Darcy's law (Eq. S7.2.4 & Eq. S7.2.5), while demand transpiration curves are determined by the diffusion of vapor through stomata (Eq. S3.1.4) with stomatal conductance predicted by our optimality model (Eq. S3.3.10 & Eq. S3.3.11) and with VPD predicted by the *steady-state* leaf heat balance (Eq. S7.2.1, Eq. S7.2.2, & Eq. S7.2.3). In (g), stomatal conductance was arbitrarily set as the lesser of the value predicted by our optimality model and 0.03 $mol \cdot m^{-2} \cdot s^{-1}$ so that the demand curve can be seen without adjusting the limits of the *y*-axis; otherwise, the demand curve would have exceeded the supply curve even more than already shown. In the *subcritical* regime (e), $\psi_L > \psi_{L,crit}$. In the *supercritical* regimes (f-g), $\psi_L < \psi_{L,crit}$. We differentiate between the *stable supercritical* regime (c & f), in which a *steady-state* solution is found since supply and demand curves intersect at a leaf water potential that is more negative than the critical leaf water potential, and an *unstable supercritical* regime (c & g), in which the *steady-state* demand curve exceeds the supply curve for all possible values of ψ_L, meaning that the supply can never satisfy the demand, leading to desiccation (all else being equal).

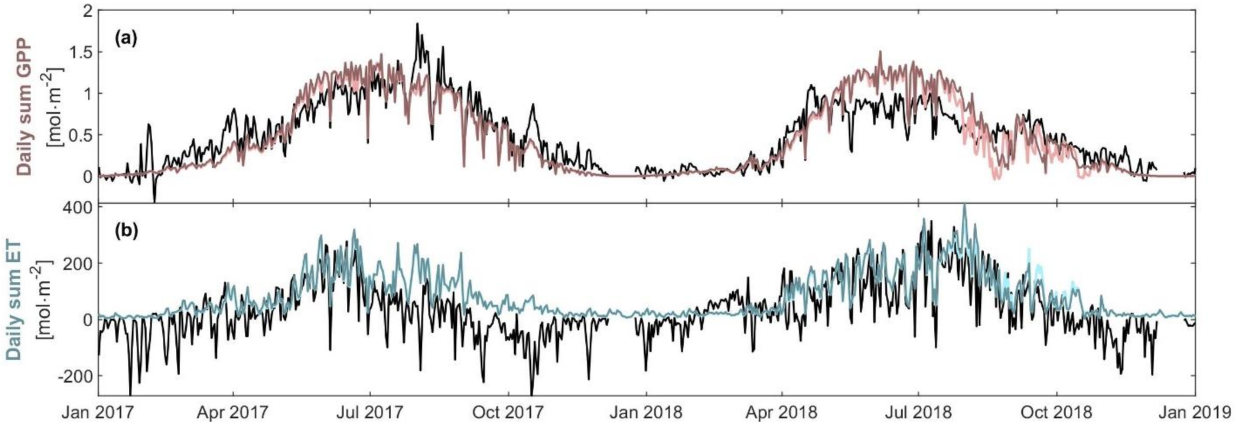

Fig. S23. Observed ground area-specific daily sum gross primary productivity (GPP; a) and evapotranspiration (ET; b) from the Oberbärenburg (DE-Obe) eddy covariance site between 2017-2018 (black lines) in comparison to Sperry et al.'s (2017) Gain-Risk model (darker lines) (Section S7.4) and to our ecosystem-scale minimalist model (lighter lines) (Section S7.5). The lines for the Gain-Risk model and our model almost entirely overlap for 2017 and early 2018 and begin to diverge from one another in June 2018. In June 2018, our model predicts slightly lower daily GPP and daily ET than the Gain-Risk model, and in July 2018 and afterwards, our model predicts slightly greater daily ET than the Gain-Risk model. In July 2018 and afterwards, our model often predicts lower daily GPP than the Gain-Risk model, although there are periods when our model predicts higher daily GPP than the Gain-Risk model.

Supporting Information Text

Contents:

S1. General solution to dynamic feedback optimization with non-steady-state conditions

S1.1. Implications for soil water saving strategies and plant competition for soil water

S2. Example model of plant water and carbon transport and storage

Table S2.1. General system of equations solved by coupled xylem-phloem models

Table S2.2. Symbols and meanings in the general coupled xylem-phloem model

S3. A note on the non-steady-state *marginal carbon profit of water*

S3.1. Comparison of the steady-state and the non-steady-state *marginal profit*

S3.2. Effect of diffusion-fixation decoupling on the non-steady-state *marginal profit*

S3.3. Derivation of stomatal conductance for known non-steady-state *marginal profit*

S4. Specific solution to dynamic feedback optimization with non-steady-state conditions through leaf pressure-volume theory

Table S4.1. Symbols and meanings in the specific solution for the *marginal cost*

S4.1. Key expressions in derivation

S4.2. Solution to the $\dot{\pi}_{L,0}$ term

S4.3. Solution to the $\dot{\pi}_L$ term

S4.4: Final solution for the *marginal carbon cost of water* with constrained osmotic potential

S4.5. The *marginal carbon cost of water* with constrained turgor pressure

S4.6. The *marginal carbon cost of water* with constrained total water potential

S4.7. Equivalence to *instantaneously* maximizing the time-rate of change of leaf solute concentration and leaf hydration

S5. Overview of input data and filtering

S6. Model calibration

S6.1. Differentiation between species-specific and treatment-specific parameters

S6.2. Calculation of the predicted stomatal conductance

S6.3. Parameters and prior distributions

Table S6.1. DREAM_(KZS)-specific parameters

S6.4. Comparison to predictions from the USO model

Table S6.2. Coefficients for full linear mixed effects model for g_1 parameter

Table S6.3. Coefficients for simplified linear mixed effects model for g_1 parameter

S7. Additional analyses

S7.1. Stomatal response to leaf water potential, leaf temperature, and osmotic potential at full hydration

S7.2. Stomatal response to VPD by varying either air temperature or relative humidity with a minimalist whole-plant plant hydraulics and leaf energy balance model

S7.3 *Supercritical* leaf water potentials

S7.4 Alternative stomata models with a minimalist whole-plant plant hydraulics and leaf heat balance model

S7.5 Ecosystem-scale predictions with a minimalist whole-plant plant hydraulics and leaf heat balance model

S7.5.1 Soil water and soil evaporation

S7.5.2 Electron transport-limitation, absorbed radiation, and boundary heat conductance

S7.5.3 LAI-corrected hydraulic conductance

S7.5.4 Calibration

S8. Guide for future implementation

S1. General solution to dynamic feedback optimization with non-steady-state conditions

Here, we derive a general solution of the optimal stomatal conductance, g_w, that ***dynamically*** maximizes an ***objective*** integrated over time under ***non-steady-state conditions***. We show that, when certain conditions are met, particularly under ***non-steady-state conditions*** for certain ***objectives*** (e.g., growth, survival, reproduction) and a single foliar ***constraint***, the ***dynamic feedback*** stomata optimization problem reduces to an ***instantaneous*** solution. Please see Box S1 for terminology. Additionally, it gives the stomatal conductance that simultaneously maximizes many ***objectives*** or proxies for evolutionary fitness, which we show below. The maximization problem is expressed mathematically as

$$\max_{g_w}\left[\int \mathbb{O}\, dt\right], \qquad \text{Eq. S1.1}$$

where $\mathbb{O}$ is the ***objective*** that is maximized over time, *t*, by the ***dynamic feedback optimization*** of stomatal conductance to water vapor, g_w. We currently leave $\mathbb{O}$ undefined to investigate the general impacts of treating the optimization problem dynamically (i.e., maximizing $\int \mathbb{O}\, dt$ instead of maximizing $\mathbb{O}$ instantaneously; Box S1) rather than the details for specific formulations for $\mathbb{O}$. In past dynamic feedback stomatal optimizations, $\mathbb{O}$ has often been treated as various forms of net carbon assimilation, A_n (Cowan. 1982, 1986; Mäkelä et al., 1996; Manzoni et al., 2013a; Lu et al., 2016; Mrad et al., 2019), including those assuming additional hydraulic shadow costs (Bartlett et al., 2019; Lu et al., 2020), while Potkay and Feng (2023a,b) treated $\mathbb{O}$ as growth. Alternatively, $\mathbb{O}$ could be associated with other physiological processes, like tissue water content or turgor (particularly of the meristem), carbohydrate storage, and hydraulic conductance of the soil-plant-atmosphere continuum, or processes linked to evolutionary fitness with less tangible links to stomatal conductance, like reproduction and survival. $\mathbb{O}$ may be defined deterministically or stochastically. For example, Lu et al. (2016, 2020) maximized mean photosynthetic carbon assimilation averaged across potential stochastic realizations of soil moisture, and Potkay and Feng (2023b) considered the maximization of the mean growth rate averaged across potential realizations of air temperature. This stochastic interpretation of the ***objective*** is appropriate when plants are assumed to be not fully 'aware' of the environmental conditions under which they operate in the current or future times and instead 'anticipate'

environmental conditions stochastically based on those that either the individual or its ancestors have experienced previously (Potkay & Feng, 2023b). $\mathbb{O}$ may also be defined broadly in a manner that integrates multiples objectives so not to assume that stomatal behavior collapses onto a single objective (Blonder et al. 2023). Virtually all past stomata optimization theories, both dynamic feedback and instantaneous, have assumed ***steady-state physics*** for leaf temperature and hydraulics (e.g., sap flux, water potentials) (with the notable exception of Bartlett et al., 2019) to mathematically relate g_w to ***objective*** functions that are defined in terms of hydraulic conductance or water potentials (Wolf et al., 2016; Sperry et al., 2017). In reality, plant hydraulics depend on internal plant water storage or hydraulic capacitance, and temperatures similarly depend on thermal capacitance. We believe that stomatal optimization theories will benefit from moving beyond simply assuming ***steady-state*** as well as from describing the full ***non-steady-state*** reality of water and thermal storage in plants. Here, we relax the steady-state assumption and consider the impacts of ***non-steady-state physics*** and thus hydraulic and thermal capacitances on the stomatal optimization problem. An example description of non-steady-state plant hydraulics is given in Section S2.

Suppose $\mathbb{O}$ is limited by the availability of many ***resources*** relevant to its maximization over time. Typically, for ***objectives*** that are relevant to fitness, these ***resources*** represent the pools of water, carbohydrates, and nutrients that are accessible to the plant, inside either plant tissues or surrounding soils. For example, growing structural biomass and reproductive tissues requires reserves of water, carbohydrates, and nutrients, and similarly, survival requires maintaining minimum levels of these reserves. These ***resources*** are relevant to the optimization problem, because current resource-use will affect the future availability of resources and thus also the future trajectory of the ***objective*** (Feng et al., 2022). ***Resources*** are not necessarily identical to the mathematical ***constraints*** on the optimization problems. We use ***resource*** as a term to denote substrates or reserves that are key to describing biological processes or physical transport, while we use ***constraint*** to specifically refer to the ***resources*** that are mathematically formulated within the optimization problem. One could propose a system in which every ***resource*** was treated as a ***constraint***; however, such a system may not be necessary, since multiple ***resources*** may be mathematically lumped into a single ***constraint*** in some instances. In past dynamic feedback stomatal optimizations, these ***constraints*** have taken the form of plant-available soil-water (Cowan, 1982, 1986; Mäkelä et al., 1996; Manzoni et al., 2013a; Lu et al., 2016, 2020; Mrad et al., 2019), internal plant water (Bartlett et al., 2019), and carbohydrate storage (Potkay & Feng, 2023a,b). For a system with N ***constraints***, we denote each ***constraint*** by X_i for $i \in R_{all}$, where R_{all} is the set of the indices for all ***constraints***, i.e., $R_{all} = \{1, 2, \ldots N\}$. For potential definitions of $\mathbb{O}$ related to plants' evolutionary fitness, relevant ***resources*** include (but are not limited to) the carbohydrates and water content of each organ distributed throughout the plant, including leaves, twigs, branches, stems, boles, and roots, or potentially exterior to the plant, such as water or nutrients stored in the soil. These resources may be further differentiated by spatial position. For example, leaf ***resources*** may be differentiated between ***resources*** in upper canopy versus ***resources*** in the lower canopy, such that the carbohydrates or water of one leaf may be considered a separate ***resource*** as the carbohydrates or water of another leaf. This logic applies to ***constraints*** as well. The simplest system of N ***resources*** is shown in Fig. 1 in the main text, including 8 ***resource*** pools: 4 carbon pools for leaves, stems, roots, and soil and 4 water pools also for leaves, stems, roots, and soil. We ignore nutrient ***resources*** here, because, though nutrients certainly impact biological functioning and thus fitness, nutrient uptake and use is beyond the control of stomata, which regulate solely transpiration, E, and photosynthetic assimilation, A_n.

To solve Eq. S1.1 for the optimal stomatal conductance to water vapor, g_w, we apply the calculus of variations (Witelski & Bowen, 2015) like other past dynamic feedback stomatal optimizations (Cowan. 1982; Mäkelä et al., 1996; Manzoni et al., 2013a; Mrad et al., 2019; Potkay & Feng, 2023a,b). Please see Potkay and Feng's (2023a) Supplementary Information for complete details of the calculus of variations. The first step is to write the augmented Lagrangian given N ***constraints***,

$$\mathcal{L} = \mathbb{O} - \sum_{i \in R_{all}} \eta_i \left(\dot{X}_i - F_i\right), \qquad \text{Eq. S1.2}$$

where for each i, η_i is an arbitrary undefined Lagrange multiplier, $\dot{X}_i$ is the time-derivative of the ***constraint***, X_i ($\dot{X}_i = \frac{\partial X_i}{\partial t}$), and F_i is an arbitrary undefined term that describes the dynamics of $\dot{X}_i$, such that together F_i and $\dot{X}_i$ form the ***constraint*** on X_i (i.e., $F_i \equiv \dot{X}_i$) The next step of the calculus of variations is to solve the *state equation* by differentiating $\mathcal{L}$ by each Lagrange multiplier, η_i, which leads to an equivalence of F_i and $\dot{X}_i$ and recovers the ***constraint***,

$$\dot{X}_i \equiv F_i(E, A_n, T_i, X_1, X_2, \cdots, X_N). \quad \text{Eq. S1.3}$$

We have written Eq. S1. 3 to show on which variables it is generally reasonable to expect $\dot{X}_i$ to depend, like transpiration, E, net carbon assimilation, A_n, temperature of the ith resource, T_i, and any of the i resources, X_i, even those at other locations. $\dot{X}_i$ may depend on the ***constraints*** at other locations (e.g., X_{i-1}, X_{i+1}), because transport processes, which may affect the mass balance of the ***constraint*** in question (X_i), often depend on spatial gradients in concentrations or thermodynamic quantities (e.g., pressure, temperature) and thus on the nearby, adjacent ***constraints***. For example, based on the non-steady-state plant hydraulics model described in Section S2, if xylem relative water content of a specific location, $\text{RWC}_x(z)$, is considered a ***constraint*** (i.e., an X_i), then its dynamic constraint on the optimization (i.e., the fact that F_i and $\dot{X}_i$ are equivalent; Eq. S1.3) is expressed in terms of $\dot{\text{RWC}}_x(z)$ (equivalent to $C_x \frac{\partial \psi_x}{\partial t}$ in Table S2.1), which depends on the difference in axial xylem water fluxes above and below that location ($\frac{\partial j_{w,x}}{\partial z}$ in Table S2.1), the radial xylem-to-phloem water flux ($q_{w,x-p}$ in Table S2.1), and potentially transpiration, E (if considering the xylem of leaves). Axial and radial water fluxes ($j_{w,x}$ and $q_{w,x-p}$) depend on the axial and radial gradients in pressure potential or total water potential ($\frac{\partial \psi_x}{\partial z}$ and $(\psi_x - \psi_p)$ in Table S2.1), respectively, which may be alternatively expressed as a dependence on the axial and radial gradients in relative water contents, since water pressures and water contents are constitutively related. Resolving the axial gradient in water content requires additional knowledge of the xylem relative water contents at points above and below the original location, $\text{RWC}_x(z + \Delta z)$ and $\text{RWC}_x(z - \Delta z)$, respectively, where Δz is an arbitrary, small distance. Similarly, the radial gradient in water content also requires the phloem relative water content at the same elevation, $\text{RWC}_p(z)$. Hence, $\dot{\text{RWC}}_x(z)$ may be expressed as a function of E (if considering relative water contents of leaf xylem) and several other resources, including $\text{RWC}_x(z + \Delta z)$, $\text{RWC}_x(z - \Delta z)$, and $\text{RWC}_p(z)$ in this example. Hence, Eq. S1.3 may be viewed as a general mathematical form for the $\dot{X}_i$ that are relevant to conserving water and carbohydrates. We have written Eq. S1.3 to denote functional dependence of F_i terms such that, for E as an example, while $\partial F_i/\partial E|_t$ may be nonzero depending on the location and nature of the ***constraint***, $\partial \dot{X}_i/\partial E|_t$ is treated as zero for all i for the sake of the calculus of variations when, and only when, differentiating the augmented Lagrangian term (Eq. S1.2; Mrad et al., 2019; Potkay & Feng, 2023a; Witelski & Bowen, 2015; e.g., $\frac{\partial \mathcal{L}}{\partial E} = \frac{\partial \mathbb{O}}{\partial E} + \sum_{i \in R_{all}} \eta_i \frac{\partial F_i}{\partial E}$). We emphasize that this approach to differentiating the augmented Lagrangian is strictly notational and would be unnecessary had we presented our derivation through simpler Hamiltonians (e.g., Box 1 & 2). Nonetheless, both approaches give identical solutions. For example, the Hamiltonian equivalent to our Eq. S1.2 would be $\mathcal{H} = \mathbb{O} + \sum_{i \in R_{all}} \eta_i \dot{X}_i$ (Box 1). When using Hamiltonian notation, differentiation of Eq. S1.2 occurs normally and provides the same solution as using the Lagrangian notation (e.g., $\frac{\partial \mathcal{H}}{\partial E} = \frac{\partial \mathbb{O}}{\partial E} + \sum_{i \in R_{all}} \eta_i \frac{\partial \dot{X}_i}{\partial E}$, which is equivalent to $\frac{\partial \mathcal{L}}{\partial E} = \frac{\partial \mathbb{O}}{\partial E} + \sum_{i \in R_{all}} \eta_i \frac{\partial F_i}{\partial E}$ from before since $\dot{X}_i \equiv F_i$ by Eq. S1.3).

The next step of the calculus of variations is to solve the *control equation* by differentiating $\mathcal{L}$ (Eq. S1.2) by the control variable (i.e., stomatal conductance, g_w) while time, t, is held constant and setting the result equal to zero. Here, we will employ a series of arguments to simplify the form of the control equation. When differentiating $\mathcal{L}$ by g_w, we first expand through the chain-rule to decompose each differential term into its differentials with respect to transpiration, E, and net carbon assimilation, A_n, since these are the only two variables that are directly controlled by stomatal conductance,

$$\left.\frac{\partial \mathcal{L}}{\partial g_w}\right|_t = \begin{bmatrix} \left.\frac{\partial \mathbb{O}}{\partial E}\right|_t \frac{\partial E}{\partial g_w} + \left.\frac{\partial \mathbb{O}}{\partial A_n}\right|_t \frac{\partial A_n}{\partial g_w} \\ + \sum_{i \in R_{all}} \eta_i \left\{ \begin{matrix} \left.\frac{\partial F_i}{\partial E}\right|_t \frac{\partial E}{\partial g_w} + \left.\frac{\partial F_i}{\partial A_n}\right|_t \frac{\partial A_n}{\partial g_w} + \left.\frac{\partial F_i}{\partial T_i}\right|_t \frac{\partial T_i}{\partial E}\frac{\partial E}{\partial g_w} \\ + \sum_{j=1}^{N} \left.\frac{\partial F_i}{\partial X_j}\right|_t \left(\left.\frac{\partial X_j}{\partial E}\right|_t \frac{\partial E}{\partial g_w} + \left.\frac{\partial X_j}{\partial A_n}\right|_t \frac{\partial A_n}{\partial g_w} \right) \end{matrix} \right\} \end{bmatrix} = 0, \text{ Eq. S1.4}$$

where the subscript of t denotes partial derivatives that are evaluated with time held constant. It is important to note that the terms in Eq. S1.4 are zero for all i ***constraints*** that are defined as carbohydrates and water (i.e., $\partial X_i/\partial E|_t = 0$; $\partial X_i/\partial A_n|_t = 0$; Table S2.1). While the dynamic change in a ***constraint***, $\dot{X}_i$, may indeed depend on transpiration or carbon assimilation, specially referring to the fact that $\partial \dot{X}_i/\partial E|_t \neq 0$ and $\partial \dot{X}_i/\partial A_n|_t \neq 0$ in leaves where transpiration and carbon assimilation take place (though $\partial \dot{X}_i/\partial E|_t = 0$ and $\partial \dot{X}_i/\partial A_n|_t = 0$ elsewhere in the plant), the ***constraint*** itself, X_i, is not functionally dependent on E or A_n. This reasoning is analogous to why, in Lagrangian mechanics, the spatial position of an object, say x, and the object's velocity, v, are independent from each other. In other words, a change in v at time t has no effect on x at time t (i.e., $\partial x/\partial v|_t = 0$), even though a change in v would affect the trajectory of future x at times greater than t. The same is true for leaf temperature such that $\partial T_i/\partial E|_t = 0$ under non-steady-state conditions. Hence, Eq. S1.4 becomes

$$\left.\frac{\partial \mathcal{L}}{\partial g_w}\right|_t = \left.\frac{\partial \mathbb{O}}{\partial E}\right|_t \frac{\partial E}{\partial g_w} + \left.\frac{\partial \mathbb{O}}{\partial A_n}\right|_t \frac{\partial A_n}{\partial g_w} + \sum_{i \in R_{all}} \eta_i \left(\left.\frac{\partial F_i}{\partial E}\right|_t \frac{\partial E}{\partial g_w} + \left.\frac{\partial F_i}{\partial A_n}\right|_t \frac{\partial A_n}{\partial g_w} \right) = 0. \quad \text{Eq. S1.5}$$

For any ***objective*** that is not explicitly defined in terms of E and A_n and thus satisfies $\partial \mathbb{O}/\partial E|_t = 0$ and $\partial \mathbb{O}/\partial A_n|_t = 0$, the first two terms on the right-hand side of Eq. S1.5 disappear. Potkay and Feng (2023a,b) previously showed that realistic stomatal behavior can be predicted by $\mathbb{O}$ defined as growth with ***steady-state*** plant hydraulics. In their model, growth was explicitly formulated as a function of E through the steady-state effect of transpiration on turgor (P_p), which limits growth. Under ***non-steady-state conditions***, however, growth ceases to depend on transpiration explicitly and instantaneously, because, though changes in transpiration will influence the trajectory of future turgor and growth, changes in transpiration will not immediately affect turgor and growth of nonfoliar tissues. That is, $\left.\frac{\partial \mathbb{O}}{\partial E}\right|_t = \left.\frac{\partial \mathbb{O}}{\partial P_p}\frac{\partial P_p}{\partial E}\right|_t = 0$, since $\left.\frac{\partial P_p}{\partial E}\right|_t = 0$ under ***non-steady-state conditions***, despite that $\left.\frac{\partial \dot{P}_p}{\partial E}\right|_t \neq 0$ when xylem and phloem are coupled (i.e., $\psi_x = \psi_p = P_p + \pi_p$ when the xylem-to-phloem radial conductance, $k_{x\text{-}p}$, is very large, which Potkay and Feng (2023a,b) had assumed), since $\left.\frac{\partial \dot{\psi}_x}{\partial E}\right|_t \neq 0$ according to Table S2.1 and variables identified in Table S2.2. In addition to growth, definitions for the ***objective*** that satisfy $\partial \mathbb{O}/\partial E|_t = 0$ and $\partial \mathbb{O}/\partial A_n|_t = 0$ include turgor, water content (and thus also hydraulic conductance), carbohydrate storage, growth, reproduction, and survival against drought-induced mortality, particularly for nonfoliar tissues. For these ***objective*** functions, Eq. S1.5 further simplifies to

$$\left.\frac{\partial \mathcal{L}}{\partial g_w}\right|_t = \sum_{i \in R_{all}} \eta_i \left(\left.\frac{\partial F_i}{\partial E}\right|_t \frac{\partial E}{\partial g_w} + \left.\frac{\partial F_i}{\partial A_n}\right|_t \frac{\partial A_n}{\partial g_w} \right) = 0. \qquad \text{Eq. S1.6}$$

From Eq. S1.6, it may be shown that only ***constraints*** in leaves are relevant to a simplified form of the *control equation*. In other words, the η_i terms for resources located in nonfoliar tissues (e.g., twigs, branches, stems, roots) may be nonzero; however, they have no impact on $\partial \mathcal{L}/\partial g_w|_t$, because $\partial F_i/\partial E|_t = 0$ and $\partial F_i/\partial A_n|_t = 0$ for all nonfoliar tissues under ***non-steady-stat conditions***. Only the water and carbon ***resources*** of leaves are directly impacted by transpiration and net carbon assimilation. In fact, if foliar ***constraints*** are distinguished among individual leaves (i.e., the water content of one leaf is considered as a separate ***constraint*** from the water content of another leaf elsewhere in the canopy), then the *control equation* describes each leaf individually, since the transpiration and net carbon assimilation of one leaf affects only its own ***constraints*** and does not directly affect the ***constraints*** of another leaf. So, not all of the original N ***constraints*** are relevant. Instead, only ***constraints*** which have indices within a foliar subset

of all the ***constraints***, $i \in R_{leaf}$, matter to the *control equation*, where $R_{leaf} \subseteq R_{all}$, and $R_{leaf} = \{i \in R_{all} \mid \text{organ}_i$ is leaf$\}$, and Eq. S1.6 is equivalent to

$$\left.\frac{\partial \mathcal{L}}{\partial g_w}\right|_t = \sum_{i \in R_{leaf}} \eta_i \left(\left.\frac{\partial F_i}{\partial E}\right|_t \frac{\partial E}{\partial g_w} + \left.\frac{\partial F_i}{\partial A_n}\right|_t \frac{\partial A_n}{\partial g_w} \right) = 0. \qquad \text{Eq. S1.7}$$

Typically, the next step of the calculus of variations would be to solve the *co-state equation* (i.e., $\partial\mathcal{L}/\partial X_i|_t - d/\mathrm{d}t(\partial\mathcal{L}/\partial \dot{X}_i|_t) = 0$) to solve for the dynamics of η_i (i.e., an expression for $\dot{\eta}_i = d\eta_i/dt$) that correspond to all of the ***constraints***, including nonfoliar ***constraints***. Even nonfoliar ***constraints*** should be considered when solving for the dynamics of each η_i, because the evolution of the Lagrange multiplier for one ***constraint***, η_i, can depend on the values of the Lagrange multiplier for other ***constraints***. In this case, the *co-state equation* would be solved *N* times, once for each of the *N* ***constraints***, leading to *N* expressions, one for each of the Lagrange multipliers. However, for a system in which each leaf has only one ***constraint*** (i.e., foliar water content, foliar carbohydrates, or a quantity that reflects both), which we will denote by the singular foliar index, $i = 1$, it would be unnecessary to solve for the *N* expressions for the time-derivatives of the Lagrange multipliers through the *co-state equation*. When considering only one foliar ***constraint***, solving the *co-state equation* would be unnecessary because Eq. S1.7 would simplify to

$$\left.\frac{\partial \mathcal{L}}{\partial g_w}\right|_t = \eta_1 \left(\left.\frac{\partial F_1}{\partial E}\right|_t \frac{\partial E}{\partial g_w} + \left.\frac{\partial F_1}{\partial A_n}\right|_t \frac{\partial A_n}{\partial g_w} \right) = 0, \qquad \text{Eq. S1.8}$$

in which case, the exact value of the Lagrange multiplier for this singular foliar ***constraint***, η_1, is unnecessary to solve for the optimal stomatal conductance, since $\left(\left.\frac{\partial F_1}{\partial E}\right|_t \frac{\partial E}{\partial g_w} + \left.\frac{\partial F_1}{\partial A_n}\right|_t \frac{\partial A_n}{\partial g_w} \right) = 0$ regardless of the value of η_1. The formulation given by Eq. S1.8 does not require that there exists one sole ***constraint*** for the optimization problem. In fact, Eq. S1.8 supports systems with many ***constraints*** ($N > 1$). Instead, the prerequisite to Eq. S1.8 is that there exists only one leaf ***constraint***; however, there may be many ***constraints*** of different types in other nonfoliar organs or even outside of the plant, such as water stored in the soil. That is, Eq. S1.8 is valid for describing a plant with one foliar ***constraint*** (i.e., foliar water content, foliar carbohydrates, or a quantity that reflects both) and many nonfoliar ***constraints*** (e.g., soil water content, stem water content, root water content, stem carbohydrates, root carbohydrates, etc.). Hence, Eq. S1.8 describes systems in which soil water content is included as a nonfoliar ***constraint***, and it thus considers the possibility that plants limit their transpiration to conserve soil-water for future water-use (Mäkelä et al., 1996; Manzoni et al., 2013a), which is often called the *water-use strategy*. The general solution to Eq. S1.8 is

$$\frac{\frac{\partial A_n}{\partial g_w}}{\frac{\partial E}{\partial g_w}} \equiv \chi_w = -\frac{\left.\frac{\partial F_1}{\partial E}\right|_t}{\left.\frac{\partial F_1}{\partial A_n}\right|_t} \equiv -\frac{\left.\frac{\partial \dot{X}_1}{\partial E}\right|_t}{\left.\frac{\partial \dot{X}_1}{\partial A_n}\right|_t}, \qquad \text{Eq. S1.9}$$

since the exact value of η_1 does not affect the solution, and where χ_w is the *marginal carbon cost of water* [mol·mol^{-1}], using the notation of Potkay and Feng (2023a,b) to be consistent with previous notations of instantaneous gain-risk stomata optimization models (e.g., Wolf et al., 2016; Wang et al., 2020). According to their notation (Wolf et al., 2016; Wang et al., 2020; Potkay & Feng, 2023a,b,), the *marginal carbon cost of water* as a term refers to the solution for $(\partial A_n/\partial g_w)/(\partial E/\partial g_w)$ that emerges *a posteriori* from the solution of the optimization problem (Eq. S1.1), while the *marginal carbon profit of water* refers to a separate solution for $(\partial A_n/\partial g_w)/(\partial E/\partial g_w)$ that is derived from the physics of gas exchange and the biochemistry of photosynthesis and respiration (Buckley et al., 2017). Stomatal conductance is optimal when the two distinct solutions are equivalent. We translate values of χ_w into predictions of g_w through either Eq. S3.1.9 or Eq. S3.2.3 (depending on whether CO_2 diffusion and carbon fixation are assumed to be coupled or decoupled, respectively; Section S3.2), both of which describe the general relationship between χ_w and g_w. These relationships are implicit functions without explicit definitions of g_w in terms of χ_w. For explicit definitions of g_w in terms of χ_w, Eq. S3.2.6 or Eq. S3.3.11 (depending on whether diffusion and fixation are decoupled or coupled, respectively) may be applied, although their derivations ignore boundary layer resistances. Please

see Section S3 for a discussion about and solution to the *marginal carbon profit of water* under non-steady-state conditions.

Interestingly, Eq. S1.9 is an effectively ***instantaneous solution*** (Box S1) to the stomatal optimization problem, since it is independent of any dynamic Lagrange multipliers (i.e., η_1), despite that the problem was initially set up as a ***dynamic feedback optimization*** (Eq. S1.1). In other words, even though our solution considers the possibility that plants limit their transpiration to conserve soil-water for future water-use (Mäkelä et al., 1996; Manzoni et al., 2013a) or open their stomata to assimilate carbohydrates to fuel later metabolic consumption (Potkay & Feng, 2023b), these possible dynamic feedback strategies are unnecessary, because stomata can affect only foliar ***resources*** under ***non-steady-state conditions***. Additionally, according to Eq. S1.9, the exact definition of the ***objective*** is irrelevant to our problem, since $\mathbb{O}$ terms and any of its derivatives are missing from Eq. S1.6-9, and since Eq. S1.9 does not depend on η_1, even though η_1 changes dynamically with a rate that would depend on the definition of the ***objective*** if we had solved the *co-state equation* for η_1. Thus, many different ***objective*** functions all lead to the same optimal stomatal conductance (Eq. S1.9) as long as they satisfy $\partial\mathbb{O}/\partial E|_t = 0$ and $\partial\mathbb{O}/\partial A_n|_t = 0$ under ***non-steady-state conditions***, which were prerequisites to simplifying Eq. S1.5 to Eq. S1.6. ***Objective***s that satisfy this requirement include turgor, water content (and thus also hydraulic conductance), carbohydrate storage, reproduction, growth, and survival of nonfoliar tissues against drought-induced mortality, regardless of whether the ***objective*** is defined deterministically or stochastically. In fact, any ***objective*** that is both distal to the leaf and depends on plants' internal water and carbon storage meet our criteria. In other words, according to Eq. S1.9, the *marginal cost* that maximizes growth is the same *marginal cost* that maximizes reproduction, and this *marginal cost* is the same as that which would maximize turgor, water content, hydraulic conductance, carbon storage, or survival. It is also the same *marginal cost* that would maximize any combination of these various proxies for fitness. All in all, Eq. S1.9 describes a stomatal strategy that holistically maximizes evolutionary fitness, rather than a single proxy for fitness, since it simultaneously maximizes each of the components of fitness. In Section S4.7, we show that Eq. S1.9 is equivalent to ***instantaneously*** maximizing the time-rate of change of the leaf solute concentration or maximizing the leaf hydration status, depending on the choice of the ***constraint***.

To apply Eq. S1.9, we must choose a single, appropriate foliar ***constraint***, X_1, for optimal stomatal behavior. In our previous work, we had theorized turgor as the ***constraint*** on optimal stomatal behavior (Potkay & Feng, 2023a,b), because turgor is constitutively linked to tissue water content (Tyree & Hammel, 1972; Campbell et al., 1979; Bartlett et al., 2012), and together they are fundamental requirements for both plant survival (Sapes et al., 2019; Sapes & Sala, 2021; Mantova et al., 2021) and growth (Lockhart, 1965; Kirkham et al., 1972; Pantin et al., 2012). Turgor also integrates the effects of both carbohydrate storage and plant hydraulics (Sapes et al., 2019; Martinez-Vilalta et al., 2019). Indeed, recent empirical studies by Peters et al. (2023a,b) support the notion that stomatal behavior is linked to plant turgor. Nonetheless, we here focus on leaf osmotic potential, π_L, for X_1 rather than leaf turgor, because all options for the single foliar ***constraint*** require analysis of the dynamics of π_L (Section S4). Our choice of π_L as the ***constraint*** is similar to other stomata theories that impose a *cost* of the turgor loss point (Deans et al., 2020), which is closely related to the osmotic potential (Bartlett et al., 2012). Hence, we express Eq. S1.9 as

$$\frac{\frac{\partial A_n}{\partial g_w}}{\frac{\partial E}{\partial g_w}} \equiv \chi_w = -\frac{\left.\frac{\partial \dot{\pi}_L}{\partial E}\right|_t}{\left.\frac{\partial \dot{\pi}_L}{\partial A_n}\right|_t}. \qquad \text{Eq. S1.10}$$

Eq. S1.10 is our general solution that emerged solely from solving the ***dynamic feedback, non-steady-state optimization problem*** for stomata conductance (Eq. S1.1) with the prerequisites that (1) the ***objective*** function does not explicitly depend on photosynthetic net carbon assimilation and transpiration (although it may in ***steady-state***), and (2) osmotic potential is an appropriate ***constraint*** on the optimization at least at the leaf level. However, Eq. S1.10 may not be applied in its current form, since the quantity on its right-hand side, i.e., $-(\partial\dot{\pi}_L/\partial E|_t)/(\partial\dot{\pi}_L/\partial A_n|_t)$, is still unknown and requires additional information to evaluate. This relevant information is provided by leaf pressure-volume theory (Tyree & Hammel, 1972;

Campbell et al., 1979; Bartlett et al., 2012), through which E and A_n may be related to $\dot{\pi}_L$. In Section S4, we derive a specific solution for the osmolyte-constrained, ***dynamic feedback***, ***non-steady-state*** stomatal optimization problem through leaf pressure-volume theory. Alternate choices for the single foliar ***constraint*** are the leaf turgor pressure, P_L, and total leaf water potential, ψ_L, both of which we also consider in Section S4.5 & S4.6.

In summary of our derivation, Eq. S1.4 is the result of expanding Eq. S1.2 given the functional dependence of F_i shown by Eq. S1.3. Eq. S1.5 is a simplification of Eq. S1.4 given the fact that $\partial T_i/\partial E|_t$, $\partial X_i/\partial E|_t$, and $\partial X_i/\partial A_n|_t$ all equal zero under ***non-steady-state conditions***. Eq. S1.6 is a further simplification of Eq. S1.5 for any ***objective*** function that is not defined explicitly in terms of photosynthesis or transpiration ($\partial\mathbb{O}/\partial E|_t = 0$; $\partial\mathbb{O}/\partial A_n|_t = 0$), which is true of any ***objective*** distal to the leaf. That is not to say that photosynthesis and transpiration are not important, because photosynthesis and transpiration are nonetheless key to maintaining the foliar ***constraint*** in the dynamic feedback optimization problem. Instead, their terms do not appear in the ***objective*** itself, which is the same as how E is not formulated in the objective function of many water-saving assimilation-maximizing theories, although E appears as a term in their ***constraint*** (Mäkelä et al., 1996; Manzoni et al., 2013a; Mrad et al., 2019). Eq. S1.7 is a simplification of Eq. S1.6, keeping only nonzero terms, since $\partial F_i/\partial E|_t = 0$ and $\partial F_i/\partial A_n|_t = 0$ for all nonfoliar tissues under ***non-steady-state conditions***. Eq. S1.8 and Eq. S1.9 are simplifications of Eq. S1.7 for a system with a single foliar ***constraint*** and any number of nonfoliar ***constraints***. Lastly, Eq. S1.10 is Eq. S1.9 for the case in which leaf osmotic potential is the single foliar ***constraint***.

S1.1. Implications for soil water saving strategies and plant competition for soil water

Our model highlights the role of carbon, instead of soil water, in controlling optimal stomatal behavior (Buckley, 2023). It suggests a *carbon-use* strategy by which stomata respond to carbohydrate storage and sink demand (Fig. 2e,f) (Kelly et al., 2013; Lawson et al., 2014). Many optimality models favor a *water-use* strategy, in which stomata are theorized to limit their current transpiration to conserve soil water for the future as part of a strategy that maximizes photosynthesis for given water availability (Cowan, 1982, 1986; Mäkelä et al., 1996; Manzoni et al., 2013a; Lu et al., 2016, 2020; Mrad et al., 2019). We explicitly included soil water as a *constraint* and its Lagrange multiplier in our *dynamic feedback* problem to account for this possibility (Fig. 1). However, like the other non-foliar *constraints* that we considered, soil water and its Lagrange multiplier do not appear in our solution for the optimal stomatal conductance (Eq. 3 & Eq. S1.10) because of our *non-steady-state* perspective and inclusion of plant water storage that decouples transpiration from soil water uptake. The absence of soil water and its Lagrange multiplier means that our resulting *instantaneous* solution does not explicitly conserve soil water is independent of the *water-use* strategy. Furthermore, our optimal stomatal conductance is independent of soil water competition with neighboring plants, since an effect of competition would require a dependence on a Lagrange multiplier for soil water that evolves with competitor's soil water uptake (Cowan, 1982; Mrad et al., 2019). Our model predicts that stomata do not open and transpire more water when faced with more competition for soil water. That is not to say that plants do not conserve soil water or increase their water use when exposed to greater competition. We simply mean that stomata are not a means for plants to do so. Plants may optimize other traits in a manner that considers soil water saving and competition, including structural traits (height, rooting depth, embolism resistance, leaf areas, sapwood areas) and even the foliar traits that control stomatal responses over shorter timescales (PV traits, SWC_L, c_{DM}; see Section S4). For example, plants could grow more leaves under greater competition to use soil water more aggressively and prevent neighbors from accessing it.

Whereas previous theories have justified *instantaneous* stomata optimization at ecosystem scales (Wolf et al., 2016), we provide, for the first time, a theoretical justification for *instantaneous* stomatal behavior at the scale of plant individuals. Whereas earlier *dynamic feedback optimization* theories were approximated as *instantaneous* to simplify mathematical solutions (Cowan & Farquhar, 1977), Wolf et al.

(2016) was the first to explicitly justify *instantaneous* stomatal optimization as the best strategy at an ecosystem scale for soil water competition. They argued that *instantaneous* behavior and nonconservative transpiration at the ecosystem level were the result of competition for soil water among entire populations, assuming that only the most abundant population depletes ecosystem-level soil water. Their premise has been used to justify many recent *instantaneous* stomata optimality models (Sperry et al., 2017; Wang et al., 2020; Eller et al., 2020; Joshi et al., 2022). However, such models often describe individuals, not ecosystems, and their assumption does not apply to individuals, which always deplete soil water regardless of population abundances. As such, recent *instantaneous* stomata optimality models are premised upon an eco-evolutionary process that is appropriate only at an ecosystem scale. By taking on a *non-steady-state* perspective, our model justifies *instantaneous* behavior at the scale of individual plants. While both we and Wolf et al. have justified *instantaneous* behavior, the theories suggest different relationships between stomatal behavior and competition for soil water. Whereas Wolf et al.'s theory posited that *instantaneous* behavior was the result of competition, our model posits that stomata do not open to allow for more transpiration when faced with greater competition because of *instantaneous* behavior.

S2. Example model of plant water and carbon transport and storage

Here, we provide an example of a general coupled xylem-phloem transport model (CXPTM) based on past CXPTMs (Daudet et al., 2002; Thompson & Holbrook, 2003; Hölttä et al., 2006, 2009; Steppe et al., 2006; Lacointe & Minchin, 2008; De Schepper & Steppe et al., 2010; Peters et al., 2021). CXPTMs describe the ***non-steady-state physics*** of water and carbon transport and storage within plants. These models generally simultaneously solve a series of equations describing the dynamics of xylem pressure (ψ_x), phloem turgor pressure (P_p), phloem sugar concentration (c_p), and starch reserves (S) that are similar to those shown in Table S2.1 with symbols explained in Table S2.2. Note that equations describing transport in Table S2.1 are expressed in terms of pressure and, when necessary, sugar concentration. Total water potentials may be subsequently estimated from pressure and osmotic potential (π_p), the latter of which depends on phloem sugar concentrations. In our CXPTM, we assume xylem are rigid, although we consider the elasticity of phloem, and we ignore the plastic deformations due to cell expansion (Lockhart, 1965) that are considered in some CXPTMs (Steppe et al., 2006; De Schepper & Steppe, 2010). In Table S2.1, we express equations of mass conservation in terms of their fluxes rather than fully simplifying them into terms of gradients of thermodynamic quantities (e.g., pressure, total water potential, concentration), because boundary conditions at the leaf and root tip alter these fluxes.

Table S2.1: General system of equations solved by coupled xylem-phloem models (CXPTMs)

Process	Mass Conservation	Flux Equation	Boundary Conditions at Leaf	Boundary Conditions at Root tip
**Axial xylem water transport**	$a_x C_x \frac{\partial \psi_x}{\partial t} = \frac{m_w}{\rho_w}\left(-\frac{\partial j_{w,x}}{\partial z} - q_{w,x-p} - T\right)$	$j_{w,x} = -K_x\left(\frac{\partial \psi_x}{\partial z} + \varpi \rho_w g\right)$	$j_{w,x} = 0$<br>$T = \frac{a_L E}{l_L}$	$j_{w,x} = k_{s-r}(\psi_s - \psi_x)$
**Radial xylem-phloem water transport**	n/a	$q_{w,x-p} = k_{x-p}(\psi_x - \psi_p) = k_{x-p}\left(\psi_x - P_p - \pi_p(c_p)\right)$	n/a	n/a
**Axial phloem water transport**	$\frac{\partial a_p}{\partial t} = \frac{a_p}{\epsilon_p}\frac{\partial P_p}{\partial t}$	$j_{w,p} = -K_p\left(\frac{\partial P_p}{\partial z} + \varpi \rho_p g\right)$	$j_{w,p} = 0$	$j_{w,p} = 0$

	$= \frac{m_w}{\rho_w}\left(-\frac{\partial j_{w,p}}{\partial z} + q_{w,x-p}\right)$			
**Axial phloem sugar transport**	$\frac{\partial}{\partial t}\left(c_p a_p\right) = a_p \frac{\partial c_p}{\partial t} + c_p \frac{a_p}{\epsilon_p} \frac{\partial P_p}{\partial t} = -\frac{\partial j_{c,p}}{\partial z} + L - U - \frac{\partial S}{\partial t}$	$j_{c,p} = c_p \frac{m_w}{\rho_w} j_{w,p}$	$j_{c,p} = 0$ $L = \frac{a_L A_n}{l_L}$	$j_{c,p} = 0$

Table S2.2: Symbols and meanings in the general coupled xylem-phloem model

Symbol	Units	Meaning
a_L	m^2	Leaf area
a_p	m^2	Cross-sectional areas of phloem
a_x	m^2	Cross-sectional areas of xylem (sapwood)
c_p	$mol \cdot m^{-3}$	Phloem sugar concentration
C_x	MPa^{-1}	Xylem capacitance
E	$mol \cdot s^{-1} \cdot m^{-2}$	Leaf area-specific transpiration rate
g	$m \cdot s^{-2}$	Downward acceleration to due to gravity
$j_{c,p}$	$mol \cdot s^{-1}$	Axial adjective sugar flux in the phloem
$j_{w,p}$	$mol \cdot s^{-1}$	Axial water flux in the phloem
$j_{w,x}$	$mol \cdot s^{-1}$	Axial water flux in the xylem
K_p	$mol \cdot s^{-1} \cdot m \cdot MPa^{-1}$	Hydraulic conductivity of the phloem
k_{s-r}	$mol \cdot s^{-1} \cdot MPa^{-1}$	Soil-to-root hydraulic conductance
K_x	$mol \cdot s^{-1} \cdot m \cdot MPa^{-1}$	Hydraulic conductivity of the xylem
k_{x-p}	$mol \cdot s^{-1} \cdot m^{-1} \cdot MPa^{-1}$	Xylem-to-phloem radial hydraulic conductance per unit length
L	$mol \cdot s^{-1} \cdot m^{-1}$	Loading of recent assimilates into the phloem of leaves per unit length
l_L	m	Effective leaf length over which transpiration and photosynthesis occur
m_w	$kg \cdot mol^{-1}$	Molar mass of water
P_p	MPa	Phloem turgor pressure
$q_{w,x-p}$	$mol \cdot s^{-1} \cdot m^{-1}$	Xylem-to-phloem radial flux per unit length
S	$mol \cdot m^{-1}$	Starch storage expressed as a number of moles of sugar equivalents per unit length
t	s	Time
T	$mol \cdot s^{-1} \cdot m^{-1}$	Transpiration of water from leaves per unit length
U	$mol \cdot s^{-1} \cdot m^{-1}$	Phloem unloading per unit length
z	m	Axial coordinate (elevation above the ground surface)
ϵ_p	MPa	Phloem elastic modulus
ϖ	$MPa \cdot Pa^{-1}$	Unit conversion factor from pascals to megapascals
π_p	MPa	Phloem osmotic potential (dependent on phloem sugar concentration)
ρ_p	$kg \cdot m^{-3}$	Density of phloem sap (dependent on phloem sugar concentration)
ρ_w	$kg \cdot m^{-3}$	Density of water
ψ_p	MPa	Phloem total water potential
ψ_s	MPa	Soil water pressure
ψ_x	MPa	Xylem water pressure

S3. A note on the non-steady-state *marginal carbon profit of water*

In this section, we discuss various interpretations of the *marginal carbon profit of water* (λ). First in Section S3.1, we introduce the ***non-steady-state*** *marginal profit* (λ_{nss}) and how it differs from past ***steady-state*** interpretations (λ_{ss}). We provide a relationship between gas exchange variables and the ***non-steady-state*** *marginal carbon profit of water*, λ_{nss}, based on Buckley et al. (2002, 2017) and Potkay and Feng (2023a,b) (notably including boundary layer conductances). In Section S3.1, we derive both λ_{ss} and λ_{nss} assuming coupling between diffusion of CO_2 through stomata and biological carbon fixation (i.e., intercellular leaf CO_2 concentrations, c_i, are in ***steady-state***). Second in Section S3.2, we explain the implications of the case in which diffusion and biological carbon fixation are decoupled (i.e., c_i is treated as in ***non-steady-state***) on the *marginal profit*; we denote this version of the *marginal profit* with diffusion-fixation decoupling as λ'_{nss}. Third in Section S3.3, we provide a relatively simple analytical expression for the stomatal conductance to water vapor, g_w, for a known a ***non-steady-state*** *marginal carbon profit of water*, λ_{nss} (with diffusion-fixation coupling). This derivation is based on Dewar et al. (2018), although we include foliar respiration, which they had not considered. Like them, we ignore boundary layer conductances for this simplified expression. Please see Table S1 for a summary of many of the symbols used throughout Section S3.

S3.1. Comparison of the steady-state and the non-steady-state *marginal profit*

Here, the *marginal carbon profit of water* refers to the ratio of the changes in leaf area-specific photosynthetic net carbon assimilation, A_n, and transpiration, E, that were to result if stomatal conductance, g_w, changed ($\lambda = (\partial A_n/\partial g_w)/(\partial E/\partial g_w)$). On one hand, the *marginal profit* is estimated from laws of gas exchange and biophysical models of photosynthesis (Buckley et al., 2017). On the other hand, in virtually all stomata optimization theories, the *marginal carbon cost of water*, χ_w, is the variable actually being specified by the optimization objective problem (see Section S1 and S3 for χ_w). If not explicitly solved in terms of χ_w, their solutions can be readily expressed in terms of χ_w (Wang et al., 2020). Once χ_w is known, and the optimal g_w is found as the value of g_w at which its corresponding λ is equivalent to χ_w ($\lambda \equiv \chi_w$). This step necessarily requires being able to transform between λ and g_w, an expression for which can be derived through the following four expressions:

(1) A biochemical expression for photosynthesis and net carbon assimilation, A_n (typically the model of Farquhar et al. (1980), in which A_n is expressed as a Michaelis-Menten function of the leaf internal CO_2 concentration, c_i [mol·mol^{-1}], with temperature-dependent parameters based on the leaf-temperature, T_L); however the exact formulation for this biochemical expression is trivial for defining λ, since the only terms from this biochemical expression that are relevant to expressing λ are $\partial A_n/\partial c_i$, which we denote by k ($k = \partial A_n/\partial c_i$), and $\partial A_n/\partial T_L$. However, as we will explain soon, the latter term, $\partial A_n/\partial T_L$, matters only in ***steady-state***. We will express this equation in a general Michaelis-Menten form, based on Dewar et al. (2018), though we also include leaf dark respiration, which Dewar et al. (2018) had not considered,

$$A_n = f_0 \frac{c_i - \Gamma^*}{c_i + \gamma} - R_d, \qquad \text{Eq. S3.1.1}$$

where f_0 is the theoretical maximum assimilation rate [mol·m^{-2}·s^{-1}], Γ^* is the CO_2 compensation point [mol·mol^{-1}], γ is a Michaelis-Menten coefficient [mol·mol^{-1}], R_d is the dark respiration rate [mol·m^{-2}·s^{-1}], and f_0, Γ^*, γ, and R_d are typically treated as functions of leaf temperature, T_L, although they have also been described as functions of either leaf total water potential, ψ_L [MPa] (Dewar et al., 2018), or leaf sugar concentration, $c_L^{S,Su}$ [mol·m^{-3}] (Hölttä et al., 2017; Dewar et al., 2022). Given Eq. S3.1.1, k is

$$k = f_0 \frac{\Gamma^* + \gamma}{(c_i + \gamma)^2}. \qquad \text{Eq. S3.1.2}$$

(2) The diffusional equation for A_n,

$$A_n = g_c(c_a - c_i), \qquad \text{Eq. S3.1.3}$$

where g_c is the total conductance to CO_2, including stomatal and boundary layer resistances [$mol \cdot m^{-2} \cdot s^{-1}$], and c_a is the atmospheric CO_2 partial pressure [$mol \cdot mol^{-1}$]. In this section, we assume that diffusion (Eq. S3.1.3) and biological carbon fixation (Eq. S3.1.1) are coupled (i.e., $g_c(c_a - c_i) = f_0 \frac{c_i - \Gamma^*}{c_i + \gamma} - R_d$); however, such coupling represents a ***steady-state*** interpretation of c_i (Hari et al., 1986). In Section S3.2, we relax this assumption of diffusion-fixation coupling in the derivation of the λ (λ'_{nss}).

(3) The diffusional equation for E,

$$E = \frac{D_L}{P_{atm}\left(\frac{1}{g_w} + \frac{1}{g_b}\right)} = \frac{e'_L - e_a}{P_{atm}\left(\frac{1}{g_w} + \frac{1}{g_b}\right)}, \qquad \text{Eq. S3.1.4}$$

where D_L is the leaf-to-air vapor pressure deficit (VPD) [kPa], P_{atm} is the atmospheric pressure [kPa], and g_b is the leaf boundary layer conductance to vapor [$mol \cdot m^{-2} \cdot s^{-1}$], e'_L is the saturation vapor pressure of the leaf at T_L [kPa], and e_a is the vapor pressure of the air given its temperature and relative humidity [kPa],

(4) An expression to relate g_c and g_w,

$$\frac{1}{g_c} = \frac{1.6}{g_w} + \frac{1.37}{g_b}. \qquad \text{Eq. S3.1.5}$$

First, we will show the general solution for the relationship between g_w and $\lambda = (\partial A_n/\partial g_w)/(\partial E/\partial g_w)$ in ***steady-state***, and afterwards we will compare the ***steady-state*** λ (λ_{ss}) to the ***non-steady-state*** λ (λ_{nss}). In the context of λ_{ss}, ***steady-state*** particularly refers to the assumption that relevant state variables (notably those that influence A_n, particularly T_L, but also ψ_L and $c_L^{S,Su}$) have reached an equilibrium with their environment and internal plant system as if infinite time had passed under constant environmental conditions (Box S1). Through this ***steady-state*** perspective, it is possible to write expressions for these state variables directly in terms of A_n and E, for which their derivatives with respect to stomatal conductance will be non-zero (e.g., $\partial T_L/\partial g_w \neq 0$, $\partial \psi_L/\partial g_w \neq 0$, and $\partial c_L^{S,Su}/\partial g_w \neq 0$ in steady-state). Under actual ***non-steady-state conditions***, the dynamics of these state variables depend on A_n and E (e.g., $\partial \dot{T}_L/\partial g_w \neq 0$, $\partial \dot{\psi}_L/\partial g_w \neq 0$, and $\partial \dot{c}_L^{S,Su}/\partial g_w \neq 0$), but the values themselves do not ($\partial T_L/\partial g_w = 0$, $\partial \psi_L/\partial g_w = 0$, and $\partial c_L^{S,Su}/\partial g_w = 0$), unlike under ***steady-state conditions***. Interestingly, only some stomatal optimization theories actually require a ***steady-state*** perspective for λ. The classic stomatal optimization theory by Cowan and Farquhar (1977) requires a ***steady-state*** perspective due to their mathematical solution to their optimization problem. Additionally, other theories that implicitly assume that A_n is instantaneously maximized (i.e., $\chi_w = 0$; Hölttä et al., 2017; Dewar et al., 2019, 2022) are meaningful only in ***steady-state***, since λ_{nss} may equal 0 at realistic values of g_w under only ***steady-state conditions*** for certain values of g_w. The fact that theories that instantaneously maximize A_n (i.e., $\chi_w = 0$) are meaningful only under ***steady-state*** conditions is demonstrated below by the function form of our solution for the ***non-steady-state*** *marginal profit* (Eq. S3.1.9), which cannot satisfactorily be zero. Yet another set of stomatal optimization theories often apply this ***steady-state assumption*** in their calculation of λ (particularly those of the instantaneous gain-risk sort; Sperry et al., 2017; Wang et al., 2020), despite that ***steady-state*** is not, strictly speaking, necessary for them.

In ***steady-state***, the *marginal carbon profit of water* is expressed by applying the chain- and product-rules to Eq. S3.1.2 and Eq. S3.1.4 as

$$\lambda_{ss} = \frac{k\frac{dc_i}{dg_c}\frac{\partial g_c}{\partial g_w} + \frac{\partial A_n}{\partial T_{L,ss}}\frac{dT_{L,ss}}{dg_w} + \frac{\partial A_n}{\partial \psi_{L,ss}}\frac{d\psi_{L,ss}}{dg_w} + \frac{\partial A_n}{\partial c_{L,ss}^{S,Su}}\frac{dc_{L,ss}^{S,Su}}{dg_w}}{\frac{\partial E}{\partial g_w} + \frac{\partial E}{\partial e'_{L,ss}}\frac{de'_{L,ss}}{dT_{L,ss}}\frac{dT_{L,ss}}{dg_w}}, \qquad \text{Eq. S3.1.6}$$

where $T_{L,ss}$ is the leaf temperature calculated in ***steady-state***, $e'_{L,ss}$ is the saturation vapor pressure of the leaf evaluated at $T_{L,ss}$ [kPa], $\psi_{L,ss}$ is the leaf total water potential calculated in ***steady-state*** [MPa], and $c_{L,ss}^{S,Su}$ is the leaf sugar concentration calculated in ***steady-state*** [$mol \cdot m^{-3}$]. We will not provide expressions for the

$dT_{L,ss}/dg_w$, $d\psi_{L,ss}/dg_w$, and $dc_{L,ss}^{S,Su}/dg_w$ terms here; however, expressions are available elsewhere (Buckley et al., 2002, 2017; Dewar et al., 2018; Potkay & Feng, 2023a,b). Instead, we emphasize their presence in Eq. S3.1.6 for the ***steady-state*** *marginal carbon profit of water*. The first two of these terms are positive, and the last is negative (Hölttä et al., 2017). To simplify Eq. S3.1.6, the dc_i/dg_c term must be further simplified by differentiating both definitions of A_n (Eq. S3.1.1 and Eq. S3.1.3) with respect to g_c, setting them equal, simplifying through Eq. S3.1.5, and rearranging for dc_i/dg_c,

$$\frac{dA_n}{dg_c} = \frac{\partial A_n}{\partial g_c} + \frac{\partial A_n}{\partial c_i}\frac{dc_i}{dg_c} = k\frac{dc_i}{dg_c} + \frac{\partial A_n}{\partial T_{L,ss}}\frac{dT_{L,ss}}{dg_w}\frac{\partial g_w}{\partial g_c} \qquad \text{Eq. S3.1.7a}$$

$$\Rightarrow \frac{dA_n}{dg_c} = \frac{A_n}{g_c} - g_c\frac{dc_i}{dg_c} = k\frac{dc_i}{dg_c} + \frac{\partial A_n}{\partial T_{L,ss}}\frac{dT_{L,ss}}{dg_w}\frac{1}{1.6}\frac{{g_w}^2}{{g_c}^2} \qquad \text{Eq. S3.1.7b}$$

$$\Rightarrow \frac{dc_i}{dg_c} = \frac{1}{k+g_c}\left(\frac{A_n}{g_c} - \frac{\partial A_n}{\partial T_{L,ss}}\frac{dT_{L,ss}}{dg_w}\frac{1}{1.6}\frac{{g_w}^2}{{g_c}^2}\right). \qquad \text{Eq. S3.1.7c}$$

Plugging Eq. S3.1.7 for dc_i/dg_c into Eq. S3.1.6 and simplifying through Eq. S3.1.4 and Eq. S3.1.5, the ***steady-state*** *marginal carbon profit of water* becomes

$$\lambda_{ss} = \frac{\frac{k}{k+g_c}\left(1.6\frac{g_c}{{g_w}^2}A_n - \frac{\partial A_n}{\partial T_{L,ss}}\frac{dT_{L,ss}}{dg_w}\right) + \frac{\partial A_n}{\partial T_{L,ss}}\frac{dT_{L,ss}}{dg_w} + \frac{\partial A_n}{\partial \psi_{L,ss}}\frac{d\psi_{L,ss}}{dg_w} + \frac{\partial A_n}{\partial c_{L,ss}^{S,Su}}\frac{dc_{L,ss}^{S,Su}}{dg_w}}{\frac{P_{atm}}{D_L}\frac{E^2}{{g_w}^2} + \frac{E}{D_L}\frac{de'_{L,ss}}{dT_{L,ss}}\frac{dT_{L,ss}}{dg_w}} \qquad \text{Eq. S3.1.8a}$$

$$\lambda_{ss} = \frac{D_L}{E}\frac{1.6\frac{kg_c}{k+g_c}A_n + {g_w}^2\left(\frac{g_c}{k+g_c}\frac{\partial A_n}{\partial T_{L,ss}}\frac{dT_{L,ss}}{dg_w} + \frac{\partial A_n}{\partial \psi_{L,ss}}\frac{d\psi_{L,ss}}{dg_w} + \frac{\partial A_n}{\partial c_{L,ss}^{S,Su}}\frac{dc_{L,ss}^{S,Su}}{dg_w}\right)}{EP_{atm} + {g_w}^2\frac{de'_{L,ss}}{dT_{L,ss}}\frac{dT_{L,ss}}{dg_w}}. \qquad \text{Eq. S3.1.8b}$$

Conversely, the ***non-steady-state*** *marginal carbon profit of water* is equivalent to Eq. S3.1.8 when the dT_L/dg_w, $d\psi_L/dg_w$, and $dc_L^{S,Su}/dg_w$ terms are set to zero, giving our final expression for the ***non-steady-state*** *marginal profit*,

$$\lambda_{nss} = 1.6\frac{kg_c}{k+g_c}\frac{D_L}{P_{atm}}\frac{A_n}{E^2}. \qquad \text{Eq. S3.1.9}$$

Because A_n has an optimum with respect to E (Fig. S14a), the ***steady-state*** *marginal profit* may be equal to or less than zero due to the negative $dT_{L,ss}/dg_w$, $d\psi_{L,ss}/dg_w$, and $\partial A_n/\partial c_{L,ss}^{S,Su}$ terms in Eq. S3.1.8 (shown by rapidly declining λ_{ss} in Fig. S14b), the ***non-steady-state*** *marginal profit* is zero only when stomatal conductance is very large ($g_w \rightarrow \infty$) according to Eq. S3.1.9 (Fig. S14b). Hence, theories that implicitly assume that A_n is instantaneously maximized and thus postulate a *marginal cost* of zero ($\chi_w = 0$; Hölttä et al., 2017; Dewar et al., 2018, 2022) are no longer meaningful in ***non-steady-state***, since the ***non-steady-state*** *marginal profit* is so rarely zero.

S3.2. Consideration of diffusion-fixation decoupling on the non-steady-state *marginal profit*

Elsewhere in Section S3, we have assumed that the diffusion of CO_2 through stomata and the biological fixation of carbon through photosynthesis are coupled. This assumption of diffusion-fixation coupling means that the internal leaf CO_2 concentration, c_i, is treated as in ***steady-state***. Since our objective is to derive a ***non-steady-state*** theory, we consider it necessary to thoroughly investigate the case of diffusion-fixation decoupling for. Hari et al. (1986) is the only past study that we are aware to consider diffusion-fixation decoupling in an optimization framework. Analogous to their Eq. 4, the mass conservation equation for c_i is

$$h\frac{dc_i}{dt} = g_c(c_a - c_i) - \left(f_0\frac{c_i - \Gamma^*}{c_i + \gamma} - R_d\right), \qquad \text{Eq. S3.2.1}$$

where h is represents the mean thickness of the intercellular leaf space in units of mol·m^{-2}, reflecting the capacitance term for the dynamics of c_i. The first set of terms on the right-hand side of Eq. S3.2.1 ($g_c(c_a - c_i)$) is the diffusion of CO_2 into the leaf (same as Eq. S3.1.3), and the second set of terms ($f_0 \frac{c_i - \Gamma^*}{c_i + \gamma} - R_d$) is the biological carbon fixation (same as Eq. S3.1.1). When c_i is in ***steady-state*** ($\frac{dc_i}{dt} = 0$), the diffusion and fixation are equal, both terms can be denoted by A_n (like in Section S3.1). Thus, stomata directly control fixation in addition to (or through) diffusion, and both diffusion and fixation may be associated with A_n. However, under ***non-steady-state conditions***, stomatal conductance controls only the diffusion term without instantaneously impacting fixation. Hence, in ***non-steady-state***, A_n should be associated only with the diffusion term for the sake of evaluating the *marginal profit*, λ. That is, $A_n = g_c(c_a - c_i)$, and c_i is no longer an explicit function of g_c. Hence, the dA_n/dg_c term is no longer described by Eq. S3.1.7 for diffusion-fixation decoupling. Instead, dA_n/dg_c is simply

$$\left(\frac{dA_n}{dg_c}\right)'_{nss} = c_a - c_i = \frac{A_n}{g_c}, \quad \text{Eq. S3.2.2}$$

where the *nss* subscript denotes ***non-steady-state conditions***, and the prime symbol (′) denotes diffusion-fixation decoupling. Deriving the *marginal profit* following the same steps as in Section S3.1 for ***non-steady-state conditions*** using Eq. S3.2.2 for dA_n/dg_c, the *marginal profit* with diffusion-fixation decoupling is

$$\lambda'_{nss} = \frac{\left(\frac{dA_n}{dg_c}\right)'_{nss} \frac{dg_c}{dg_w}}{\left(\frac{dE}{dg_c}\right)_{nss}} = 1.6 g_c \frac{D_L}{P_{atm}} \frac{A_n}{E^2}. \text{Eq. S3.2.3}$$

According to Eq. S3.2.3, λ'_{nss} is the limit of the ***non-steady-state*** *marginal profit* with diffusion-fixation coupling (λ_{nss}; Eq. S3.1.9) for k approaching infinity, because $\lambda_{nss} = \frac{k}{k+g_c} \lambda'_{nss}$. More so, $\lambda'_{nss} \geq \lambda_{nss}$, end their values are equal when g_c is zero and in the limit as k becomes very small, which happens when g_c is large under diffusion-fixation coupling. In fact, λ'_{nss} is at least an order of magnitude larger than λ_{nss} and λ_{ss} for realistic values of g_w (Fig. S14b), and g_w is more sensitive to changes in λ'_{nss} than to changes in either λ_{nss} and λ_{ss} (Fig. S14b).

Unlike in derivations for expressions for g_w in terms of λ that assume diffusion-fixation coupling (Section S3.3), finding an expression for g_w in terms of λ with diffusion-fixation decoupling is comparatively much simpler and may be done in only a few steps. If ignoring boundary layer resistances (i.e., $g_b \to \infty$), this simplicity results may be shown by simplifying Eq. S3.2.3 through Eq. S3.1.3, Eq. S3.1.4 and Eq. S3.1.5 to

$$\lambda'_{nss} = \frac{P_{atm}}{1.6 D_L} \frac{A_n}{g_c} = \frac{P_{atm}}{1.6 D_L} (c_a - c_i). \quad \text{Eq. S3.2.4}$$

Hence, c_i can easily be rearranged from Eq. S3.2.4 as $c_i = c_a - \phi$, where ϕ is unitless shorthand for

$$\phi = c_a - c_i = \frac{A_n}{g_c} = 1.6 \cdot \mathrm{iWUE} = 1.6 \frac{D_L}{P_{atm}} \lambda'_{nss}, \quad \text{Eq. S3.2.5}$$

where iWUE is the intrinsic water-use efficiency ($\mathrm{iWUE} = \frac{A_n}{g_w}$). Hence, ϕ carries information about both the *marginal profit*, λ'_{nss}, and the vapor pressure deficit, D_L. Using this solution for c_i, and assuming that the mean thickness of the intercellular leaf space (h in Eq. S3.2.1) is small such that we may approximate the rate of CO_2 diffusion into the leaf by the rate of biological carbon fixation (Eq. S3.1.1), the diffusion rate, A_n, may be approximated by

$$A_n \approx f_0 \frac{c_a - \Gamma^* - \phi}{c_a + \gamma - \phi} - R_d. \quad \text{Eq. S3.2.6}$$

Since $g_c = \frac{A_n}{\phi}$ according to Eq. S3.2.5, and since $g_w = 1.6 g_c$ when assuming negligible boundary layer resistances according to Eq. S3.1.5, g_w may be solved from the approximation for A_n given by Eq. S3.2.6,

$$g_w \approx \frac{1.6}{\phi}\left(f_0 \frac{c_a - \Gamma^* - \phi}{c_a + \gamma - \phi} - R_d\right). \qquad \text{Eq. S3.2.7}$$

The functional form of Eq. S3.2.7 for g_w results in stomatal closure to vapor pressure deficit, even for constant λ'_{nss}, since

$$\frac{\partial g_w}{\partial \phi} = -\frac{1}{\phi}\left[1.6 f_0 \frac{\gamma + \Gamma^*}{(c_a + \gamma - \phi)^2} + g_w\right], \quad \text{Eq. S3.2.8}$$

which is always negative. Conversely, to capture realistic responses of g_w and A_n to elevated atmospheric CO_2 concentrations (c_a), λ'_{nss} must increase as c_a rises. This increase in λ'_{nss} as c_a rises in contrary to the responses of λ_{ss} and λ_{nss} to c_a, since constant λ_{ss} and λ_{nss} leads to realistic stomatal responses under elevated CO_2 concentrations (Gardner et al., 2023). Assuming that ϕ is a function of c_a, the expectation that λ'_{nss} should increase as c_a rises may be shown through the total derivatives of A_n and g_w with respect to c_a, the first of which is

$$\frac{dA_n}{dc_a} = f_0 \frac{\gamma + \Gamma^*}{(c_a + \gamma - \phi)^2}\left(1 - \frac{\partial \phi}{\partial c_a}\right). \qquad \text{Eq. S3.2.9}$$

Since we expect A_n to increase under elevated CO_2 concentrations ($\frac{dA_n}{dc_a} > 0$), Eq. S3.2.9 suggests that $\frac{\partial \phi}{\partial c_a} < 1$. Similarly,

$$\frac{dg_w}{dc_a} = -\frac{1}{\phi}\left[\left(1.6 f_0 \frac{\gamma + \Gamma^*}{(c_a + \gamma - \phi)^2} + g_w\right)\frac{\partial \phi}{\partial c_a} - 1.6 f_0 \frac{\gamma + \Gamma^*}{(c_a + \gamma - \phi)^2}\right], \qquad \text{Eq. S3.2.10}$$

and since we expect stomata to close under elevated CO_2 concentrations ($\frac{dg_w}{dc_a} < 0$), $\frac{\partial \phi}{\partial c_a} > \left[1 + \frac{g_w}{1.6 f_0}\frac{(c_a + \gamma - \phi)^2}{\gamma + \Gamma^*}\right]^{-1}$. Hence, $\left[1 + \frac{g_w}{1.6 f_0}\frac{(c_a + \gamma - \phi)^2}{\gamma + \Gamma^*}\right]^{-1} < \frac{\partial \phi}{\partial c_a} < 1$ for realistic stomatal responses to elevated atmospheric CO_2 concentrations. That is, λ'_{nss} and iWUE, which are proportional to ϕ, must increase as c_a rises. According to a recent meta-analysis (Mathias & Thomas, 2021), the average $\frac{\partial \text{iWUE}}{\partial c_a} \approx 0.3$, suggesting that $\frac{\partial \phi}{\partial c_a} = 1.6 \frac{\partial \text{iWUE}}{\partial c_a} = 0.48$, the value of which fits between the lower and upper limit for $\frac{\partial \phi}{\partial c_a}$. Hence, as c_a rises, λ'_{nss} and thus also χ_w must increase, since λ and χ_w equivalent when stomata behave optimally ($\lambda = \chi_w$). Similarly, capturing realistic stomatal responses to other environmental queues (e.g., soil moisture, VPD) requires that λ'_{nss} and χ_w vary directly or indirectly due to environmental conditions.

We have considered the possibility of diffusion-fixation decoupling since our objective is to develop a *non-steady-state* theory for explaining optimal stomatal conductance. Hence, we believe it is necessary to consider the possibility that c_i is in *non-steady-state* as well. However, the formulation for stomatal conductance resulting from assuming diffusion-fixation decoupling (Eq. S3.2.7) requires stronger environmental effects on χ_w than would be required without diffusion-fixation decoupling (Eq. S3.3.11 in Section S3.3 below). For example, with diffusion-fixation coupling, the correct stomatal response to VPD can be predicted even when χ_w remains constant with respect to VPD (Katul et al., 2009; Nakad et al., 2023). However, for the case of diffusion-fixation decoupling (Eq. S3.2.7), the *marginal cost* would have to covary with VPD to produce realistic stomatal responses to VPD, which is possible since we later show that χ_w depends on the leaf water potential and thus also the leaf relative water content (Section S4), which indeed covary with VPD in *steady-state* (Fig. 3). Specifically, to capture the widely observed empirical stomatal response to VPD described by $\frac{g_w}{g_{w,ref}} = 1 - m \ln\left(\frac{D_L}{P_{atm}}\right)$ (Oren et al., 1999), where $g_{w,ref}$ is a reference conductance, and m is an approximately constant unitless parameter that ranges between 0.5 and 0.6 (Katul et al., 2009; Nakad et al., 2023), χ_w would have to rigidly vary with VPD according $\frac{d \ln(\chi_w)}{d \ln(D_L)} =$

$\frac{m g_{w,ref}}{g_w + 1.6 f_0 \frac{\gamma+\Gamma^*}{(c_a+\gamma-\phi)^2}} - 1$. This prerequisite can be derived by solving for two definitions of dE/dD_L, one from differentiating E with g_w defined by Eq. S3.2.7 and another from differentiating E with g_w defined by $\frac{g_w}{g_{w,ref}} = 1 - m \ln\left(\frac{D_L}{P_{atm}}\right)$, setting the two definitions of dE/dD_L equal to each other, and rearranging. The success of assuming diffusion-fixation coupling to capture realistic VPD responses without restrictive constraints on how χ_w must vary appears more promising than assuming diffusion-fixation decoupling.

In addition to fitting our model to gas exchange and leaf water potential data assuming coupled CO_2 diffusion and biological carbon fixation by a Markov chain Monte Carlo (MCMC) method (Section S6 and S7), we fit our model to the same data assuming that CO_2 diffusion and biological carbon fixation are decoupled (Fig. S15-S20). In this analysis, we followed the procedure outlined in Section S7, except we applied Eq. S3.2.7 to predict g_w from the ***non-steady-state*** *marginal profit* with diffusion-fixation coupling, λ'_{nss}, instead of through Eq. S3.3.11 derived in the next section. We assume that stomata behave optimally and thus that $\lambda'_{nss} = \chi_w$.

For the Ponderosa pine (r^2 = 0.39; RMSE = 0.051 mol·m^{-2}·s^{-1}) and Norway spruce (r^2 = 0.35; RMSE = 0.039 mol·m^{-2}·s^{-1}), predictions with diffusion-fixation decoupling compared about as well as our previous predictions with diffusion-fixation coupling (Fig. S4 & Fig. S15). For European larch (r^2 = 0.68; RMSE = 0.062 mol·m^{-2}·s^{-1}) and Red maple (r^2 = 0.48; RMSE = 0.040 mol·m^{-2}·s^{-1}), accounting for diffusion-fixation decoupling improved predictions for stomatal conductance. However, for Red oak (r^2 = 0.01; RMSE = 0.104 mol·m^{-2}·s^{-1}), predictions worsened. Like before, parameters were often significantly correlated with one another, and posterior distributions were often nonuniformly distributed with clear modes (Fig. S16-S20).

S3.3. Derivation of stomatal conductance for known non-steady-state *marginal profit*

Now we derive a relatively simple expression for the optimal stomatal conductance (with diffusion-fixation coupling) given a known λ_{nss} based on a similar derivation by Dewar et al. (2018; see Methods S4 in their SI). Like them, we ignore boundary layer resistances (i.e., $g_b \to \infty$), but we include leaf respiration unlike them. Dewar et al. (2018) did not consider temperature-dependences of photosynthetic parameters (f_0, Γ^*, and γ in Eq. S3.1.1), thereby implicitly assuming that $dT_{L,ss}/dg_w = 0$, and their derivation also did not consider the effects of leaf total water potentials and sugar concentrations of photosynthetic capacities, thereby implicitly assuming , $d\psi_L/dg_w = 0$ and $dc_L^{S,Su}/dg_w = 0$. Dewar et al.'s (2018) simplifying assumptions are fortunate for us, because these are the same conditions through which we derive our ***non-steady-state*** *marginal profit* (Section S3.1). Thus, their solution better describes our ***non-steady-state*** *marginal profit* (Eq. S3.1.9) than it describes the ***steady-state*** *marginal profit* (Eq. S3.1.8). Without boundary layer resistance, Eq. S3.1.4 and S3.1.5 become

$$E \approx g_w \frac{D_L}{P_{atm}} \qquad \text{Eq. S3.3.1}$$

$$g_c \approx \frac{g_w}{1.6}. \text{Eq. S3.3.2}$$

We rearrange the definition of $\lambda = (\partial A_n/\partial g_w)/(\partial E/\partial g_w)$ and simplify through Eq. S3.1.3, Eq. S3.3.1, Eq. S3.3.2 to rearrange for dc_i/dg_c,

$$\frac{\partial A_n}{\partial g_w} = \frac{dA_n}{dg_c}\frac{\partial g_c}{\partial g_w} = \lambda_{nss}\frac{\partial E}{\partial g_w} \quad \text{Eq. S3.3.3a}$$

$$\Rightarrow \frac{dA_n}{dg_c} = 1.6\lambda_{nss}\frac{D_L}{P_{atm}} \qquad \text{Eq. S3.3.3b}$$

$$\Rightarrow \frac{\partial A_n}{\partial g_c} + \frac{\partial A_n}{\partial c_i}\frac{dc_i}{dg_c} = 1.6\lambda_{nss}\frac{D_L}{P_{atm}} \qquad \text{Eq. S3.3.3c}$$

$$\Rightarrow c_a - c_i - g_c \frac{dc_i}{dg_c} = 1.6\lambda_{nss} \frac{D_L}{P_{atm}} \text{ Eq. S3.3.3d}$$

$$\Rightarrow \frac{dc_i}{dg_c} = \frac{c_a - c_i - 1.6\lambda_{nss}\frac{D_L}{P_{atm}}}{g_c} = \left(c_a - c_i - 1.6\lambda_{nss}\frac{D_L}{P_{atm}}\right)\frac{c_a - c_i}{A_n} \quad \text{Eq. S3.3.3e}$$

The dA_n/dg_c term may alternatively expressed through the chain rule and Eq. S3.1.1 as

$$\frac{dA_n}{dg_c} = k\frac{dc_i}{dg_c}. \quad \text{Eq. S3.2.4}$$

Combining Eq. S3.3.3b, Eq. S3.3.3e, and Eq. S3.3.4, we write a polynomial expression for c_i, including substituting A_n by Eq. S3.1.1 and k by Eq. S3.1.2,

$$\frac{dA_n}{dg_c} = k\left(c_a - c_i - 1.6\lambda_{nss}\frac{D_L}{P_{atm}}\right)\frac{c_a - c_i}{A_n} = 1.6\lambda_{nss}\frac{D_L}{P_{atm}} \quad \text{Eq. S3.3.5a}$$

$$\Rightarrow \frac{\Gamma^* + \gamma}{c_i + \gamma}\left(c_a - c_i - 1.6\lambda_{nss}\frac{D_L}{P_{atm}}\right)(c_a - c_i) = 1.6\lambda_{nss}\frac{D_L}{P_{atm}}\left[(c_i - \Gamma^*) - \frac{R_d}{f_0}(c_i + \gamma)\right], \text{Eq. S3.3.5b}$$

which we further transform through the dimensionless variable, $x = (c_i - \Gamma^*)/(c_a - \Gamma^*)$,

$$\Rightarrow \frac{(\Gamma^* + \gamma)(c_a - \Gamma^*)}{x(c_a - \Gamma^*) + \Gamma^* + \gamma}\left[(c_a - \Gamma^*)(1-x)^2 - 1.6\lambda_{nss}\frac{D_L}{P_{atm}}(1-x)\right] = 1.6\lambda_{nss}\frac{D_L}{P_{atm}}\left\{x(c_a - \Gamma^*) - \frac{R_d}{f_0}[x(c_a - \Gamma^*) + \Gamma^* + \gamma]\right\} \quad \text{Eq. S3.3.6a}$$

$$\Rightarrow \frac{1}{x\frac{c_a - \Gamma^*}{\Gamma^* + \gamma} + 1}\left[(1-x)^2 - \frac{1.6\lambda_{nss}\frac{D_L}{P_{atm}}}{c_a - \Gamma^*}(1-x)\right] = 1.6\lambda_{nss}\frac{D_L}{P_{atm}}\left\{\frac{x}{c_a - \Gamma^*} - \frac{R_d}{f_0}\frac{1}{(c_a - \Gamma^*)^2}[x(c_a - \Gamma^*) + \Gamma^* + \gamma]\right\} \quad \text{Eq. S3.3.6b}$$

$$\Rightarrow \frac{(1-x)^2 - y(1-x)}{\frac{z}{y}x + 1} = y\left(1 - \frac{R_d}{f_0}\right)x - \frac{y^2}{z}\frac{R_d}{f_0} \quad \text{Eq. S3.3.6c}$$

where y and z are shorthand for

$$y = \frac{1.6\lambda_{nss}\frac{D_L}{P_{atm}}}{c_a - \Gamma^*} \quad \text{Eq. S3.3.7}$$

$$z = \frac{1.6\lambda_{nss}\frac{D_L}{P_{atm}}}{\Gamma^* + \gamma}. \quad \text{Eq. S3.3.8}$$

Note that both y and z are proportional to λ_{nss}, so their ratio is independent of λ_{nss} (e.g., Eq. S3.3.11 below). Eq S3.3.6 can be further rearranged as the polynomial expression for x,

$$x^2 + (y-2)x + 1 - y = z\left(1 - \frac{R_d}{f_0}\right)x^2 + y\left(1 - 2\frac{R_d}{f_0}\right)x - \frac{R_d}{f_0}\frac{y^2}{z} \quad \text{Eq. S3.3.9a}$$

$$\Rightarrow \left[1 - z\left(1 - \frac{R_d}{f_0}\right)\right]x^2 + 2\left(y\frac{R_d}{f_0} - 1\right)x + 1 - y + \frac{R_d}{f_0}\frac{y^2}{z} = 0, \text{Eq. S3.3.9b}$$

the root of which can be solved as

$$x = \frac{1 - y\frac{R_d}{f_0} - \sqrt{\left(1 - y\frac{R_d}{f_0}\right)^2 - \left[1 - z\left(1 - \frac{R_d}{f_0}\right)\right]\left[1 + y\left(\frac{R_d y}{f_0 z} - 1\right)\right]}}{1 - z\left(1 - \frac{R_d}{f_0}\right)} \quad \text{Eq. S3.3.10a}$$

$$\Rightarrow x = \frac{1 - \frac{1.6\lambda_{nss}\frac{D_L}{P_{atm}}}{c_a - \Gamma^*}\frac{R_d}{f_0} - \sqrt{\left(1 - \frac{1.6\lambda_{nss}\frac{D_L}{P_{atm}}}{c_a - \Gamma^*}\frac{R_d}{f_0}\right)^2 - \left[1 - \frac{1.6\lambda_{nss}\frac{D_L}{P_{atm}}}{\Gamma^* + \gamma}\left(1 - \frac{R_d}{f_0}\right)\right]\left[1 + \frac{1.6\lambda_{nss}\frac{D_L}{P_{atm}}}{c_a - \Gamma^*}\left(\frac{R_d}{f_0}\frac{\Gamma^* + \gamma}{c_a - \Gamma^*} - 1\right)\right]}}{1 - \frac{1.6\lambda_{nss}\frac{D_L}{P_{atm}}}{\Gamma^* + \gamma}\left(1 - \frac{R_d}{f_0}\right)}. \text{ Eq. S3.3.10b}$$

Once x is calculated by Eq. S3.3.10, the optimal stomatal conductance may be determined from x by transforming into c_i, then A_n through Eq. S3.1.1, g_c through Eq. S3.1.3, and finally g_w through Eq. S3.3.2, providing our final expression for the optimal stomatal conductance for a known λ,

$$g_w = \frac{1.6}{(1-x)(c_a-\Gamma^*)}\left[f_0 \frac{x}{x+\frac{\Gamma^*+\gamma}{c_a-\Gamma^*}} - R_d\right] \quad \text{Eq. S3.3.11a}$$

$$\Rightarrow g_w = \frac{1.6}{(1-x)(c_a-\Gamma^*)}\left[f_0 \frac{x}{x+\frac{y}{z}} - R_d\right]. \quad \text{Eq. S3.3.11b}$$

Hence, Eq. S3.3.11 is our final solution for g_w in terms of the *marginal profit,* λ_{nss}. To apply Eq. S3.3.11 to predict g_w from the *marginal cost*, several steps must be taken:

1. The *marginal cost*, χ_w, is determined (see Sections S1 & S4; Eq. 3 in main text).

2. Stomata are assumed to be optimal, and thus the *marginal profit* is assumed equivalent to the *marginal cost* ($\lambda \equiv \chi_w$; Eq. 5 in main text).

3. Next, x is calculated by Eq. S3.3.10 from λ (Eq. 7-9 in main text).

4. Lastly, the optimal stomatal conductance to water vapor, g_w, is calculated by Eq. S3.3.11 from x (Eq. 6 in main text).

S4. Specific solution to dynamic feedback optimization with non-steady-state conditions through leaf pressure-volume theory

Here, we derive a solution for the change in leaf osmotic per unit time, $\dot{\pi}_L$, through mass balance and leaf pressure-volume theory (Tyree & Hammel, 1972; Campbell et al., 1979; Bartlett et al., 2012), which leads to an expression the *marginal carbon cost of water*, $\chi_w = \frac{\frac{\partial \pi_L}{\partial E}}{\frac{\partial \pi_L}{\partial A_n}}$ (see Section S1), in terms of the leaf pressure-volume traits. This section is subdivided into five subsections, including (S4.1) key expressions from which the rest of our derivation follows, (S4.2) a derivation of the change in the osmotic potential at full hydration per unit time, $\dot{\pi}_{L,0}$, (S4.3) a derivation of the change in the osmotic potential per unit time, $\dot{\pi}_L$, and (S4.4) our solution for χ_w and an explanation of some of its properties. In two last sections, we consider alternate definitions for χ_w with the sole foliar *constraint* being the leaf turgor pressure (S4.5) or the total water potential (S4.6) instead of the osmotic potential. We show that, regardless of the choice of the *constraint*, the mathematical formulations for χ_w are nearly identical, except for a single term. Importantly, the temperature response of χ_w is identical in all three formulations. Symbols and their meaning used in this section are summarized in Table S4.1.

Table S4.1. Symbols and meanings in the specific solution for the *marginal cost*

Symbol	Unit	Meaning
RWC_L^S	$m^3 \cdot m^{-3}$	Relative water content of symplast
RWC_L^T	$m^3 \cdot m^{-3}$	Relative water content of bulk or total leaf
SWC_L	$kg \cdot kg^{-1}$	Leaf saturated water content
Γ_L	MPa	Turgor threshold for plastic cell expansion
Λ_E	$J \cdot kg^{-1}$	Latent heat of vaporization for water
A_n	$mol \cdot m^{-2} \cdot s^{-1}$	Photosynthetic net C assimilation per unit leaf area
C_L	$J \cdot K^{-1} \cdot m^{-2}$	Leaf thermal capacitance per unit of leaf area
L^{A_n}	$mol \cdot m^{-2} \cdot s^{-1}$	Losses per unit leaf area of the first solute generated by net carbon assimilation

M_{DM}	kg	Leaf dry matter mass
$P_{L,0}$	MPa	Leaf turgor at full hydration
P_L	MPa	Leaf turgor
R_l	W·m^{-2}	Net longwave radiation balance (i.e., emitted minus absorbed)
R_s	W·m^{-2}	Absorbed shortwave radiation
T_L^K	K	Leaf temperature in Kelvin
$W_{L,0}^A$	m^3	Volume of water in the apoplastic at full hydration
$W_{L,0}^S$	m^3	Volume of water in the symplast at full hydration
$W_{L,0}^T$	m^3	Total volume of water in the bulk leaf at full hydration
W_L^A	m^3	Volume of water in the apoplastic
W_L^S	m^3	Volume of water in the symplast
W_L^T	m^3	Total volume of water in the bulk leaf
a_L	m^2	Leaf area
$a_{f,0}$	m^3·m^{-3}	Leaf apoplastic fraction at full hydration ($a_{f,0} = \frac{W_{L,0}^A}{W_{L,0}^T}$)
$a_{f,max}$	m^3·m^{-3}	Maximum leaf apoplastic fraction
a_f	m^3·m^{-3}	Leaf apoplastic fraction ($a_f = \frac{W_L^A}{W_L^T}$)
a_f^{app}	m^3·m^{-3}	Apparent leaf apoplastic fraction determined through traditional pressure-volume analysis, which assumes a constant apoplastic fraction during desiccation ($a_f^{app} \approx a_{f,0}(1-\beta)$)
c_{DM}	J·K^{-1}·kg^{-1}	Specific heat of leaf dry matter
$c_{L,0}^{S,i}$	mol·m^{-3}	Concentration of *i*th solute species in leaf symplast at full hydration
$c_L^{S,i}$	mol·m^{-3}	Concentration of *i*th solute species in leaf symplast
c_w	J·K^{-1}·kg^{-1}	Specific heat of water
m_w	kg·mol^{-1}	Molar mass of water ($m_w = 18 \times 10^{-3}\ \frac{\text{kg}}{\text{mol}}$)
$n_L^{S,i}$	mol	Number of moles of *i*th solute species in leaf symplast
$\varepsilon_{L,0}$	MPa	Elastic modulus of the symplast at full hydration
$\varepsilon_{L,max}$	MPa	Theoretical maximum elastic modulus of the symplast (as $P_L \to \infty$)
ε_L	MPa	Elastic modulus of the symplast
$\pi_{L,0}$	MPa	Osmotic potential of the leaf symplast at full hydration
$\pi_{L,0}^i$	MPa	Contribution to osmotic potential of the leaf symplast at full hydration from *i*th solute species ($\pi_{L,0} = \sum \pi_{L,0}^i$)
π_L	MPa	Osmotic potential of the leaf symplast
π_L^*	MPa	Shape parameter controlling the a_f-π_L relationship
ρ_w	kg·m^{-3}	Density of water ($\rho_w = 1000\ \frac{\text{kg}}{\text{m}^3}$)
$\chi_{w,0}$	mol·mol^{-1}	Minimum marginal cost C cost of water at full hydration
χ_w	mol·mol^{-1}	Marginal cost C cost of water
$\psi_{L,0}$	MPa	Leaf water potential at full hydration ($\psi_{L,0} = 0$ MPa by definition)
ψ_L	MPa	Leaf water potential
$\phi_{L,0}$	MPa^{-1}·s^{-1}	Leaf extensibility at full hydration
ϕ_L	MPa^{-1}·s^{-1}	Leaf extensibility
LMA	kg·m^{-2}	leaf dry matter mass per unit of leaf area
SLA	m^2·kg^{-1}	Specific leaf area
WMA	kg·m^{-2}	Water mass per unit of leaf area (sometimes referred to as the *degree of succulence*)
E	mol·m^{-2}·s^{-1}	Transpiration per unit leaf area
H	W·m^{-2}	Sensible heat flux away from the leaf
L	W·m^{-2}	Latent heat flux away from the leaf
Q	mol·m^{-2}·s^{-1}	Stem-to-leaf water influx per unit leaf area

R	J·mol[-1]·K[-1]	Universal gas constant ($R = 8.314\ \mathrm{J \cdot mol^{-1} \cdot K^{-1}}$)
k	MPa	Constant controlling shape of ε_L-P_L relationship
α	mol·mol[-1]	C-to-osmolyte conversion factor
β	-	Shape parameter controlling the a_f-π_L relationship
ϖ	MPa·Pa[-1]	Pascal to Megapascal conversion factor ($\varpi = 10^{-6}\mathrm{MPa \cdot Pa^{-1}}$)

S4.1. Key expressions in derivation

Here, we provide key expressions that will be used throughout the rest of this section to first derive an expression for the change in leaf osmotic potential per unit time, $\dot{\pi}_L$, in terms of transpiration, *E*, and net carbon assimilation, A_n, and then the *marginal carbon cost of water*, χ_w (see Section S1). The relative water content of the leaf symplast, RWC_L^S, is related to the relative water content of the bulk or total leaf, RWC_L^T:

$$\mathrm{RWC}_L^S = \frac{\mathrm{RWC}_L^T - a_f}{1 - a_{f,0}}, \qquad \text{Eq. S4.1.1}$$

where a_f is the leaf apoplastic fraction, which varies with hydration (Vos & Oyarzun, 1988; Andersen et al., 1991; Urban et al., 1993), and $a_{f,0}$ is the leaf apoplastic fraction at maximum hydration (i.e., if leaf water potentials were zero). Following Campbell et al. (1979), we predict a_f and $a_{f,0}$ functions of the osmotic potential, π_L [MPa], and the osmotic potential at maximum hydration, $\pi_{L,0}$ [MPa], based on observations that the matric potential in the cell wall nearly equals the osmotic potential of the symplast. Campbell et al. (1979) applied a power-law to describe this relationship; however, data from Urban et al. (1993) supports the use of a Weibull function, which we apply here,

$$a_f = \frac{W_L^A}{W_{L,0}^T} = a_{f,max} \exp\left[-\left(\frac{\pi_L}{\pi_L^*}\right)^{\beta}\right] \quad \text{Eq. S4.1.2}$$

$$a_{f,0} = \frac{W_{L,0}^A}{W_{L,0}^T} = a_{f,max} \exp\left[-\left(\frac{\pi_{L,0}}{\pi_L^*}\right)^{\beta}\right]. \qquad \text{Eq. S4.1.3}$$

where $a_{f,max}$ is the maximum leaf apoplastic fraction (occurring at $\pi_L = 0\ \mathrm{MPa}$), and π_L^* [MPa] and β (unitless) are shaping parameters. From Eq. S4.1.2 and Eq. S4.1.3, the derivatives of a_f with respect to osmotic potential are:

$$\frac{\partial a_f}{\partial \pi_L} = -\frac{\beta a_{f,max}}{\pi_L}\left(\frac{\pi_L}{\pi_L^*}\right)^{\beta} \exp\left[-\left(\frac{\pi_L}{\pi_L^*}\right)^{\beta}\right] = -\frac{\beta a_f}{\pi_L}\left(\frac{\pi_L}{\pi_L^*}\right)^{\beta} \quad \text{Eq. S4.1.4}$$

$$\frac{\partial a_{f,0}}{\partial \pi_{L,0}} = -\frac{\beta a_{f,max}}{\pi_{L,0}}\left(\frac{\pi_{L,0}}{\pi_L^*}\right)^{\beta} \exp\left[-\left(\frac{\pi_{L,0}}{\pi_L^*}\right)^{\beta}\right] = -\frac{\beta a_{f,0}}{\pi_{L,0}}\left(\frac{\pi_{L,0}}{\pi_L^*}\right)^{\beta}. \qquad \text{Eq. S4.1.5}$$

Past work often assumes that $\pi_{L,0}$ is constant. Here, we relax these assumptions and allow $\pi_{L,0}$ to vary according to the dynamics of osmolytes in the symplast (Section S4.2). Thus, $a_{f,0}$ also varies as $\pi_{L,0}$ varies by Eq. S4.1.3.

The leaf symplast contains different sorts of osmolytes, which we differentiate by their index, *i*. The number of solutes of the *i*th species in the leaf symplast, $n_L^{S,i}$ [mol], is the product their concentration, $c_L^{S,i}$ [mol·m^{-3}], and the volume of water in the leaf symplast, W_L^S [m^3]. Alternatively, $n_L^{S,i}$ may be expressed as the product of their concentration at full hydration, $c_{L,0}^{S,i}$ [mol·m^{-3}], and the volume of water in the leaf symplast at full hydration, $W_{L,0}^S$ [m^3],

$$n_L^{S,i} = c_L^{S,i} W_L^S = c_{L,0}^{S,i} W_{L,0}^S. \quad \text{Eq. S4.1.6}$$

The relative water content of the leaf symplast is related to these water volumes and osmolyte concentrations as

$$\mathrm{RWC}_L^S = \frac{W_L^S}{W_{L,0}^S} = \frac{c_{L,0}^{S,i}}{c_L^{S,i}}. \qquad \text{Eq. S4.1.7}$$

The relative water content of the total leaf is the ratio of the total leaf water volume, W_L^T [m³], and its value at full hydration, $W_{L,0}^T$ [m³],

$$\mathrm{RWC}_L^T = \frac{W_L^T}{W_{L,0}^T}, \qquad \text{Eq. S4.1.8}$$

where W_L^T is the sum of the volumes of water in the symplast and apoplast, W_L^S and W_L^A [m³], respectively, and $W_{L,0}^T$ is the sum of the volumes of water in the symplast and apoplast at full hydration, W_L^S and W_L^A [m³], respectively,

$$W_L^T = W_L^S + W_L^A \text{ Eq. S4.1.9}$$

$$W_{L,0}^T = W_{L,0}^S + W_{L,0}^A. \qquad \text{Eq. S4.1.10}$$

Combining Eq. S4.1.1 with Eq. S4.1.5-S4.1.8 leads to the relationship between symplastic and total water volumes and the leaf apoplastic fraction,

$$\frac{W_L^S}{W_L^T} = 1 - a_f \frac{W_{L,0}^T}{W_L^T} = 1 - \frac{a_f}{\mathrm{RWC}_L^T} \qquad \text{Eq. S4.1.11}$$

$$\frac{W_{L,0}^S}{W_{L,0}^T} = 1 - a_{f,0}. \quad \text{Eq. S4.1.12}$$

The change in the total leaf water volume at full hydration, $\dot{W}_{L,0}^S = \frac{\partial W_{L,0}^S}{\partial t}$, is given by a combination of Hooke's law and the Lockhart equation (1965). The former relates elastic changes in volume to changes in turgor. The latter relates plastic changes to volume to the magnitude of turgor. Since turgor is the difference between total and osmotic water potentials ($P_L = \psi_L - \pi_L$), and since the water potential at full hydration is zero by definition ($\psi_{L,0} = 0$), the change in turgor at full hydration equals the opposite of the change in osmotic potential at full hydration ($\dot{P}_{L,0} = -\dot{\pi}_{L,0}$),

$$\dot{W}_{L,0}^S = \frac{W_{L,0}^S}{\varepsilon_{L,0}} \dot{P}_{L,0} + W_{L,0}^S \phi_{L,0}\, \mathbb{R}\big(P_{L,0} - \Gamma_L\big) \qquad \text{Eq. S4.1.13b}$$

$$\dot{W}_{L,0}^S = -\frac{W_{L,0}^S}{\varepsilon_{L,0}} \dot{\pi}_{L,0} + W_{L,0}^S \phi_{L,0}\, \mathbb{R}\big(-\pi_{L,0} - \Gamma_L\big), \qquad \text{Eq. S4.1.13a}$$

where $\varepsilon_{L,0}$ is the elastic modulus of the leaf symplast, ε_L [MPa], at full hydration [MPa], $\mathrm{R}(x)$ is the ramp function ($\mathbb{R}(x) = \max(x,\ \ 0)$), $\phi_{L,0}$ is the extensibility at full hydration [MPa⁻¹·s⁻¹], and Γ_L is the turgor threshold required for plastic cell expansion. The change in total leaf water volume is defined by the difference between the influx of water into the leaf from the stem, Q [mol·m⁻²·s⁻¹], and the foliar transpiration, E [mol·m⁻²·s⁻¹],

$$\dot{W}_L^T = a_L \frac{m_w}{\rho_w}(Q - E), \qquad \text{Eq. S4.1.14}$$

Where a_L is the leaf area [m²], $m_w = 18 \times 10^{-3}\ \frac{\mathrm{kg}}{\mathrm{mol}}$ is the molar mass of water [kg·mol⁻¹], and $\rho_w = 10^3\, \frac{\mathrm{kg}}{\mathrm{m}^3}$ is density of water [kg·m³].

Photosynthesis creates new solutes, generating osmotic potential, which are typically discussed in terms of sugars. However, since we are developing theory for ***non-steady-state conditions***, we are

interested in the first osmolyte generated by net carbon assimilation, while sugars are the downstream results of the first products of net carbon assimilation. These non-sugar metabolites generate a significant portion of the total osmotic potential of leaves (Fu et al., 2011). The identity of this non-sugar metabolite is uncertain. If the diffusion of CO_2 through stomata into the leaf is perfectly coupled to the photosynthetic carbon fixation, then the osmolyte in question is triose phosphate (TP; Flügge, 1999). However, if the diffusion and biological carbon fixation are decoupled (Section S3.2), then the net change in dissolved gases is instead relevant to generating osmotic potential. Regardless of the exact identity of the first solute generated by net carbon assimilation, we may generally describe the change in the number of mols of this solute (subscript A_n) in the leaf symplast by the difference between the diffusive net carbon assimilation, A_n [mol·m^{-2}·s^{-1}], and a generic loss term, L^{A_n} [mol·m^{-2}·s^{-1}],

$$\dot{n}_L^{S,A_n} = a_L\left(\frac{A_n}{\alpha} - L^{A_n}\right), \quad \text{Eq. S4.1.15}$$

where α is a conversion factor between the number of moles of carbon assimilated and the number of moles of new solutes produced. If TP is the relevant solute, then the value of α further depends on the subcellular location of the osmotic potential used as the *constraint* in the optimization problem (Eq. S1.10). If the osmotic potential of the chloroplast is the relevant osmotic potential, then α is the number of moles of carbon within a mole of TP ($\alpha = 3\ \mathrm{mol}\cdot\mathrm{mol}^{-1}$). However, if the osmotic potential of the cytoplasm is the relevant osmotic potential, then α is less than the number of moles of carbon within a mole of TP ($\alpha < 3\ \mathrm{mol}\cdot\mathrm{mol}^{-1}$), since only a fraction of TP exits the chloroplasts during the day into the cytoplasm, while the remaining TP is stored as starches during the day and later broken down and transported out as glucose at night (Flügge, 1999). Due to the uncertainty in the value of α, we later treat is as a fitted parameter (Section S6).

The elastic modulus has been found to empirically follow a saturating exponential function (Steudle et al., 1977),

$$\varepsilon_L = \varepsilon_{L,max}\left[1 - \exp\left(-\frac{P_L}{k}\right)\right], \quad \text{Eq. S4.1.16}$$

where $\varepsilon_{L,max}$ is the theoretical maximum elastic modulus [MPa] ($\varepsilon_L \leq \varepsilon_{L,0} \leq \varepsilon_{L,max}$), and k is a constant controlling the steepness of Eq. S4.1.16 [MPa]. Typical values of k are small relative to the leaf turgor, P_L ($k \approx 0.1\ \mathrm{MPa}$; Campbell et al., 1979). By combining Eq. S4.1.7, Eq. S4.1.16, and Hooke's law ($\varepsilon_L = W_L^S \frac{\partial P_L}{\partial W_L^S}$), Campbell et al. (1979) derived the expression,

$$P_L = k \ln\left\{\mathrm{RWC}_L^{S\,\frac{\varepsilon_{L,max}}{k}}\left[\exp\left(-\frac{\pi_{L,0}}{k}\right) - 1\right] + 1\right\}. \quad \text{Eq. S4.1.17}$$

Note that the Lockhart equation does not need to be considered here, since Eq. S4.1.17 is derived from the definition of the elastic modulus. Since k is typically small, $\mathrm{RWC}_L^{S\,\frac{\varepsilon_{L,max}}{k}} \exp\left(-\frac{\pi_{L,0}}{k}\right) \gg 1 - \mathrm{RWC}_L^{S\,\frac{\varepsilon_{L,max}}{k}}$. Hence, Eq. S4.1.17 may be approximated as $P_L \approx -\pi_{L,0} + \varepsilon_{L,max} \ln(\mathrm{RWC}_L^S)$, which may be further approximated by its first-order Taylor-series expansion around $\mathrm{RWC}_L^S = 1$, leading to the traditional linear relationship between P_L and RWC_L^S, $P_L \approx -\pi_{L,0} + \varepsilon_{L,max}(\mathrm{RWC}_L^S - 1)$, used in common leaf pressure-volume analyses (Tyree & Hammel, 1972; Bartlett et al., 2012). Eq. S4.1.17 and the traditional linear formation differ in that Eq. S4.1.17 accounts for changes in the elastic modulus (Eq. S4.1.16), and Eq. S4.1.17 predicts $P_L = 0$ when $\mathrm{RWC}_L^S = 0$, while the linear formation predicts $P_L = 0$ at an apparent turgor loss point($1 + \frac{\pi_{L,0}}{\varepsilon_{L,0}}$). In combination with the definition of the total water potential ($\psi_L = P_L + \pi_L$), we use Eq. S4.1.17 to relate ψ_L and RWC_L^S and thus also RWC_L^T through Eq. S4.1.1.

Since osmotic potential is temperature-dependent, we must also consider the leaf thermal energy balance. Changes in leaf temperature, T_L^K [K], are described by Schymanski et al. (2013),

$$\dot{T}_L^K = \frac{R_{abs} - R_l - H - L}{C_L}, \qquad \text{Eq. S4.1.18}$$

where R_{abs} is the absorbed shortwave radiation [W·m^{-2}], R_l is the net longwave balance (i.e., emitted minus absorbed) [W·m^{-2}], H is the sensible heat flux away from the leaf [W·m^{-2}], L is the latent heat flux away from the leaf [W·m^{-2}], and C_L is the leaf thermal capacitance per unit of leaf area [J·K^{-1}·m^{-2}]. The latent heat flux is proportional to the transpiration rate, E,

$$L = m_w \Lambda_E E, \qquad \text{Eq. S4.1.19}$$

where Λ_E is the latent heat of vaporization [J·kg^{-1}]. We estimated Λ_E from leaf temperature, approximating the temperature-dependence as a linear function ($\Lambda_E = \Lambda_{E,0°\mathrm{C}} + S_{\Lambda_E} T_L$, where $\Lambda_{E,0°\mathrm{C}} = 2.5 \times 10^6\ \mathrm{J \cdot kg^{-1}}$ and $S_{\Lambda_E} = -2365\ \mathrm{J \cdot kg^{-1} \cdot °C^{-1}}$) (Friend, 1995) based on Λ_E values reported by Campbell and Norman (1998). The leaf thermal capacitance is treated as the sum of the capacitances of water and leaf dry matter,

$$C_L = \mathrm{WMA} \cdot c_w + \mathrm{LMA} \cdot c_{DM}, \qquad \text{Eq. S4.1.20}$$

where WMA is the water mass per unit of leaf area [kg·m^{-2}] (sometimes referred to as the *degree of succulence* in older literature), LMA is the leaf dry matter mass per unit of leaf area [kg·m^{-2}], and c_w and c_{DM} are the thermal capacitances per unit of mass for water and leaf dry matter [J·K^{-1}·kg^{-1}], respectively. Regardless of species, we treat c_{DM} as 2814 J·kg^{-1}·K^{-1}, based on the mean c_{DM} of seven species (Jayalakshmy & Philip, 2010), and c_w is 4181 J·kg^{-1}·K^{-1}. WMA is related to LMA, RWC_L^T, and the leaf saturated water content, SWC_L [kg·kg^{-1}], which relates the saturated water mass to the dry matter mass,

$$\mathrm{WMA} = \mathrm{LMA} \cdot \mathrm{SWC}_L \cdot \mathrm{RWC}_L^T, \qquad \text{Eq. S4.1.21}$$

through which, Eq. S4.1.20 becomes

$$C_L = \mathrm{LMA} \cdot (\mathrm{SWC}_L \cdot \mathrm{RWC}_L^T \cdot c_w + c_{DM}) = \frac{\mathrm{SWC}_L \cdot \mathrm{RWC}_L^T \cdot c_w + c_{DM}}{\mathrm{SLA}}, \text{ Eq. S4.1.22}$$

where $\mathrm{SLA} = \mathrm{LMA}^{-1}$ is the specific leaf area [m^2·kg^{-1}]. Combining Eq. S4.1.18, Eq. S4.1.29, and Eq. S4.1.22, the change in leaf temperature is expressed in terms of E as

$$\dot{T}_L^K = \mathrm{SLA} \cdot \frac{R_{abs} - R_l - H - m_w \Lambda_E E}{\mathrm{SWC}_L \cdot \mathrm{RWC}_L^T \cdot c_w + c_{DM}}. \qquad \text{Eq. S4.1.23}$$

In the following subsections, we use these definitions to derive an expression for the change in leaf osmotic potential per unit time, $\dot{\pi}_L$, in terms of transpiration, *E*, and net carbon assimilation, *A*$_n$. First, we derive expressions for the change in the osmotic potential at full hydration, $\dot{\pi}_{L,0}$ (Section S4.2) and the osmotic potential, $\dot{\pi}_L$ (Section S4.3). In the fourth subsection (Section S4.4), we use these expressions to solve for the *marginal carbon cost of water* according to Section S1 ($\chi_w = -\frac{\frac{\partial \dot{\pi}_L}{\partial E}}{\frac{\partial \dot{\pi}_L}{\partial A_n}}$).

S4.2. Solution to the $\dot{\pi}_{L,0}$ term

Since the symplastic osmotic potential is related to the symplastic solute concentrations and the leaf temperature, we write $\dot{\pi}_{L,0}$ through the chain-rule,

$$\dot{\pi}_{L,0} = \frac{\partial \pi_{L,0}}{\partial T_L^K} \dot{T}_L^K + \sum \frac{\partial \pi_{L,0}}{\partial c_{L,0}^{S,i}} \dot{c}_{L,0}^{S,i}, \qquad \text{Eq. S4.2.1}$$

Where $\sum x_i$ operator is the sum of x_i for all species of solutes. We differentiate the $n_L^{S,i} = c_{L,0}^{S,i} W_{L,0}^S$ portion of Eq. S4.1.6 and rearrange in terms of $\dot{c}_{L,0}^{S,i}$,

$$\dot{c}_{L,0}^{S,i} = \frac{\dot{n}_L^{S,i} - c_{L,0}^{S,i}\dot{W}_{L,0}^S}{W_{L,0}^S}. \qquad \text{Eq. S4.2.2}$$

We combine Eq. S4.1.13 and Eq. S4.2.2 and rearrange in terms of $\dot{c}_{L,0}^{S,i}$,

$$\dot{c}_{L,0}^{S,i} = \frac{\dot{n}_L^{S,i}}{W_{L,0}^S} - \frac{c_{L,0}^{S,i}}{W_{L,0}^S}\dot{W}_{L,0}^S \qquad \text{Eq. S4.2.3a}$$

$$\Rightarrow \dot{c}_{L,0}^{S,i} = \frac{\dot{n}_L^{S,i}}{W_{L,0}^S} + \frac{c_{L,0}^{S,i}}{\varepsilon_{L,0}}\dot{\pi}_{L,0} - c_{L,0}^{S,i}\phi_{L,0}\,\mathbb{R}\left(-\pi_{L,0} - \Gamma_L\right). \quad \text{Eq. S4.2.3b}$$

We combine Eq. S4.2.1 and Eq. S4.2.3 and rearrange for our final expression for $\dot{\pi}_{L,0}$

$$\dot{\pi}_{L,0} = \frac{\partial \pi_{L,0}}{\partial T_L^K}\dot{T}_L^K + \sum \frac{\partial \pi_{L,0}}{\partial c_{L,0}^{S,i}}\left[\frac{\dot{n}_L^{S,i}}{W_{L,0}^S} + \frac{c_{L,0}^{S,i}}{\varepsilon_{L,0}}\dot{\pi}_{L,0} - c_{L,0}^{S,i}\phi_{L,0}\,\mathbb{R}\left(-\pi_{L,0} - \Gamma_L\right)\right] \qquad \text{Eq. S4.2.4a}$$

$$\Rightarrow \dot{\pi}_{L,0} = \frac{\frac{\partial \pi_{L,0}}{\partial T_L^K}\dot{T}_L^K + \frac{1}{W_{L,0}^S}\sum\left(\frac{\partial \pi_{L,0}}{\partial c_{L,0}^{S,i}}\dot{n}_L^{S,i}\right) - \phi_{L,0}\,\mathbb{R}\left(-\pi_{L,0} - \Gamma_L\right)\sum\left(\frac{\partial \pi_{L,0}}{\partial c_{L,0}^{S,i}}c_{L,0}^{S,i}\right)}{1 - \frac{1}{\varepsilon_{L,0}}\sum\left(\frac{\partial \pi_{L,0}}{\partial c_{L,0}^{S,i}}c_{L,0}^{S,i}\right)}. \quad \text{Eq. S4.2.4b}$$

Alternatively, if all osmolytes behave the Van’t Hoff rule (i.e., $\pi_{L,0} = -\varpi R T_L^K \sum c_{L,0}^{S,i}$), then Eq. S4.2.4 simplifies to

$$\dot{\pi}_{L,0} = \pi_{L,0}\frac{\frac{\dot{T}_L^K}{T_L^K} + \frac{1}{W_{L,0}^S}\frac{\sum \dot{n}_L^{S,i}}{\sum c_{L,0}^{S,i}} - \phi_{L,0}\,\mathbb{R}\left(-\pi_{L,0} - \Gamma_L\right)}{1 - \frac{\pi_{L,0}}{\varepsilon_{L,0}}}. \qquad \text{Eq. S4.2.5}$$

S4.3. Solution to the $\dot{\pi}_L$ term

We combine Eq. S4.1.1, Eq. S4.1.7, and Eq. S4.1.8 and rearrange to get $\left(1 - a_{f,0}\right)\frac{W_L^S}{W_{L,0}^S} = \frac{W_L^T}{W_{L,0}^T} - a_f$. We differentiate this expression with respect to time, simplify through Eq. S4.1.2, Eq. S4.1.3, Eq. S4.1.12, and Eq. S4.1.13, and rearrange for $\dot{W}_L^S$,

$$\left(1 - a_{f,0}\right)\frac{1}{W_{L,0}^S}\left(\dot{W}_L^S - \frac{W_L^S}{W_{L,0}^S}\dot{W}_{L,0}^S\right) - \frac{W_L^S}{W_{L,0}^S}\dot{a}_{f,0} = \frac{1}{W_{L,0}^T}\left(\dot{W}_L^T - \frac{W_L^T}{W_{L,0}^T}\dot{W}_{L,0}^T\right) - \dot{a}_f \qquad \text{Eq. S4.3.1a}$$

$$\Rightarrow \left(1 - a_{f,0}\right)\frac{1}{W_{L,0}^S}\left(\dot{W}_L^S - \frac{W_L^S}{W_{L,0}^S}\dot{W}_{L,0}^S\right) - \frac{W_L^S}{W_{L,0}^S}\frac{\partial a_{f,0}}{\partial \pi_{L,0}}\dot{\pi}_{L,0} = \frac{1}{W_{L,0}^T}\left(\dot{W}_L^T - \frac{W_L^T}{W_{L,0}^T}\dot{W}_{L,0}^T\right) - \frac{\partial a_f}{\partial \pi_L}\dot{\pi}_L \qquad \text{Eq. S4.3.1b}$$

$$\Rightarrow \dot{W}_L^S = \dot{W}_L^T - \frac{W_L^T}{W_{L,0}^T}\dot{W}_{L,0}^T + \frac{W_L^S}{W_{L,0}^S}\dot{W}_{L,0}^S + \frac{W_L^S}{1 - a_{f,0}}\frac{\partial a_{f,0}}{\partial \pi_{L,0}}\dot{\pi}_{L,0} - \frac{W_{L,0}^S}{1 - a_{f,0}}\frac{\partial a_f}{\partial \pi_L}\dot{\pi}_L \quad \text{Eq. S4.3.1c}$$

$$\Rightarrow \dot{W}_L^S = \left\{\begin{matrix} \dot{W}_L^T - \frac{W_L^T}{W_{L,0}^T}\dot{W}_{L,0}^T \\ + W_L^S\left(\frac{1}{1 - a_{f,0}}\frac{\partial a_{f,0}}{\partial \pi_{L,0}} - \frac{1}{\varepsilon_{L,0}}\right)\dot{\pi}_{L,0} \\ - \frac{W_{L,0}^S}{1 - a_{f,0}}\frac{\partial a_f}{\partial \pi_L}\dot{\pi}_L \\ + W_L^S\phi_{L,0}\,\mathbb{R}\left(-\pi_{L,0} - \Gamma_L\right) \end{matrix}\right\}. \quad \text{Eq. S4.3.1d}$$

To simplify Eq. S4.3.1, we require expressions for $\dot{\pi}_L$ and $\dot{W}_{L,0}^T$, the latter of which further requires an expression for $\dot{W}_{L,0}^A$. To derive an expression for $\dot{W}_{L,0}^A$, we differentiate the $a_{f,0} = \frac{W_{L,0}^A}{W_{L,0}^T}$ portion of Eq. S4.1.2 with respect to time,

$$\dot{W}_{L,0}^A = a_{f,0}\dot{W}_{L,0}^T + \dot{a}_{f,0}W_{L,0}^T \qquad \text{Eq. S4.3.2a}$$

$$\Rightarrow \dot{W}_{L,0}^A = a_{f,0}\dot{W}_{L,0}^T + W_{L,0}^T\frac{\partial a_{f,0}}{\partial \pi_{L,0}}\dot{\pi}_{L,0}. \qquad \text{Eq. S4.3.2b}$$

To derive an expression for $\dot{W}_{L,0}^T$, we differentiate Eq. S4.1.10 with respect to time ($\dot{W}_{L,0}^T = \dot{W}_{L,0}^S + \dot{W}_{L,0}^A$), combine with Eq. S4.1.13 and Eq. S4.3.2b, and rearrange for $\dot{W}_{L,0}^T$,

$$\dot{W}_{L,0}^T = \dot{W}_{L,0}^S + \dot{W}_{L,0}^A \qquad \text{Eq. S4.3.3a}$$

$$\Rightarrow \dot{W}_{L,0}^T = \dot{W}_{L,0}^S + a_{f,0}\dot{W}_{L,0}^T + W_{L,0}^T\frac{\partial a_{f,0}}{\partial \pi_{L,0}}\dot{\pi}_{L,0} \quad \text{Eq. S4.3.3b}$$

$$\Rightarrow (1-a_{f,0})\dot{W}_{L,0}^T = \dot{W}_{L,0}^S + W_{L,0}^T\frac{\partial a_{f,0}}{\partial \pi_{L,0}}\dot{\pi}_{L,0} \quad \text{Eq. S4.3.3c}$$

$$\Rightarrow (1-a_{f,0})\dot{W}_{L,0}^T = -\frac{W_{L,0}^S}{\varepsilon_{L,0}}\dot{\pi}_{L,0} + W_{L,0}^T\frac{\partial a_{f,0}}{\partial \pi_{L,0}}\dot{\pi}_{L,0} + W_{L,0}^S\phi_{L,0}\,\mathbb{R}(-\pi_{L,0}-\Gamma_L) \qquad \text{Eq. S4.3.3d}$$

$$\Rightarrow \dot{W}_{L,0}^T = W_{L,0}^T\left(\frac{1}{1-a_{f,0}}\frac{\partial a_{f,0}}{\partial \pi_{L,0}} - \frac{1}{\varepsilon_{L,0}}\right)\dot{\pi}_{L,0} + W_{L,0}^T\phi_{L,0}\,\mathbb{R}(-\pi_{L,0}-\Gamma_L) \qquad \text{Eq. S4.3.3e}$$

We plug Eq. S4.3.3e into Eq. S4.3.1d and simplify to express $\dot{W}_L^S$ as

$$\Rightarrow \dot{W}_L^S = \left\{\begin{matrix} \dot{W}_L^T - \frac{W_{L,0}^S}{1-a_{f,0}}\frac{\partial a_f}{\partial \pi_L}\dot{\pi}_L \\ +W_L^S\left(\frac{1}{1-a_{f,0}}\frac{\partial a_{f,0}}{\partial \pi_{L,0}} - \frac{1}{\varepsilon_{L,0}}\right)\dot{\pi}_{L,0} - W_L^T\left(\frac{1}{1-a_{f,0}}\frac{\partial a_{f,0}}{\partial \pi_{L,0}} - \frac{1}{\varepsilon_{L,0}}\right)\dot{\pi}_{L,0} \\ +W_L^S\phi_{L,0}\,\mathbb{R}(-\pi_{L,0}-\Gamma_L) - W_L^T\phi_{L,0}\,\mathbb{R}(-\pi_{L,0}-\Gamma_L) \end{matrix}\right\} \qquad \text{Eq. S4.3.4a}$$

$$\Rightarrow \dot{W}_L^S = \left\{\begin{matrix} \dot{W}_L^T - a_f W_{L,0}^T\left(\frac{1}{1-a_{f,0}}\frac{\partial a_{f,0}}{\partial \pi_{L,0}} - \frac{1}{\varepsilon_{L,0}}\right)\dot{\pi}_{L,0} \\ -\frac{W_{L,0}^S}{1-a_{f,0}}\frac{\partial a_f}{\partial \pi_L}\dot{\pi}_L - a_f W_{L,0}^T\phi_{L,0}\,\mathbb{R}(-\pi_{L,0}-\Gamma_L) \end{matrix}\right\} \qquad \text{Eq. S4.3.4b}$$

Like in Section S4.2, we relate changes in the symplastic osmotic potential to changes in the symplastic solute concentrations and temperature for an expression for $\dot{\pi}_L$,

$$\dot{\pi}_L = \frac{\partial \pi_L}{\partial T_L^K}\dot{T}_L^K + \sum\frac{\partial \pi_L}{\partial c_L^{S,i}}\dot{c}_L^{S,i}. \text{ Eq. S4.3.5}$$

We differentiate the $n_L^{S,i} = c_L^{S,i}W_L^S$ portion of Eq. S4.1.6 and rearrange for $\dot{c}_L^{S,i}$,

$$\dot{c}_L^{S,i} = \frac{\dot{n}_L^{S,i} - c_L^{S,i}\dot{W}_L^S}{W_L^S}. \text{ Eq. S4.3.6}$$

We combine Eq. S4.3.5 and Eq. S4.3.6 for $\dot{\pi}_L$,

$$\dot{\pi}_L = \frac{\partial \pi_L}{\partial T_L^K}\dot{T}_L^K + \frac{1}{W_L^S}\sum\left(\frac{\partial \pi_L}{\partial c_L^{S,i}}\dot{n}_L^{S,i}\right) - \frac{\dot{W}_L^S}{W_L^S}\sum\left(\frac{\partial \pi_L}{\partial c_L^{S,i}}c_L^{S,i}\right). \qquad \text{Eq. S4.3.7}$$

We plug Eq. S4.3.4b into Eq. S4.3.7 and rearrange for $\dot{\pi}_L$,

$$\dot{\pi}_L = \frac{\partial \pi_L}{\partial T_L^K}\dot{T}_L^K + \frac{1}{W_L^S}\sum\left(\frac{\partial \pi_L}{\partial c_L^{S,i}}\dot{n}_L^{S,i}\right) - \frac{\sum\left(\frac{\partial \pi_L}{\partial c_L^{S,i}}c_L^{S,i}\right)}{W_L^S}\left\{\begin{array}{c} \dot{W}_L^T - a_f W_{L,0}^T\left(\frac{1}{1-a_{f,0}}\frac{\partial a_{f,0}}{\partial \pi_{L,0}} - \frac{1}{\varepsilon_{L,0}}\right)\dot{\pi}_{L,0} \\ -\frac{W_{L,0}^S}{1-a_{f,0}}\frac{\partial a_f}{\partial \pi_L}\dot{\pi}_L - a_f W_{L,0}^T\phi_{L,0}\,\mathbb{R}\left(-\pi_{L,0}-\Gamma_L\right)\end{array}\right\} \qquad \text{Eq. S4.3.8a}$$

$$\Rightarrow \dot{\pi}_L = \frac{\frac{\partial \pi_L}{\partial T_L^K}\dot{T}_L^K + \frac{1}{W_L^S}\sum\left(\frac{\partial \pi_L}{\partial c_L^{S,i}}\dot{n}_L^{S,i}\right) + \sum\left(\frac{\partial \pi_L}{\partial c_L^{S,i}}c_L^{S,i}\right)\left\{\begin{array}{c}\frac{a_f}{\frac{W_L^T}{W_{L,0}^T}-a_f}\left(\frac{1}{1-a_{f,0}}\frac{\partial a_{f,0}}{\partial \pi_{L,0}}-\frac{1}{\varepsilon_{L,0}}\right)\dot{\pi}_{L,0} \\ +\frac{a_f}{\frac{W_L^T}{W_{L,0}^T}-a_f}\phi_{L,0}\,\mathbb{R}\left(-\pi_{L,0}-\Gamma_L\right)-\frac{\dot{W}_L^T}{W_L^S}\end{array}\right\}}{1-\frac{1}{1-a_{f,0}}\frac{W_{L,0}^S}{W_L^S}\frac{\partial a_f}{\partial \pi_L}\sum\left(\frac{\partial \pi_L}{\partial c_L^{S,i}}c_L^{S,i}\right)} \qquad \text{Eq. S4.3.8b}$$

Lastly, we plug Eq. S4.2.4b into Eq. S4.3.8b for our final expression for $\dot{\pi}_L$ in terms of $\dot{T}_L^K$, $\dot{n}_L^{S,i}$, and $\dot{W}_L^T$, which can be related to A_n and E (Eq. S4.1.14, Eq. S4.1.15, Eq. S4.1.18, and Eq. S4.1.23),

$$\dot{\pi}_L = \frac{\left\{\begin{array}{c}\frac{\partial \pi_L}{\partial T_L^K}\dot{T}_L^K \\ +\frac{1}{W_L^S}\sum\left(\frac{\partial \pi_L}{\partial c_L^{S,i}}\dot{n}_L^{S,i}\right) \\ +\frac{a_f}{\frac{W_L^T}{W_{L,0}^T}-a_f}\frac{\frac{1}{1-a_{f,0}}\frac{\partial a_{f,0}}{\partial \pi_{L,0}}-\frac{1}{\varepsilon_{L,0}}}{1-\frac{1}{\varepsilon_{L,0}}\sum\left(\frac{\partial \pi_{L,0}}{\partial c_{L,0}^{S,i}}c_{L,0}^{S,i}\right)}\sum\left(\frac{\partial \pi_L}{\partial c_L^{S,i}}c_L^{S,i}\right)\left\{\frac{\partial \pi_{L,0}}{\partial T_L^K}\dot{T}_L^K+\frac{1}{W_{L,0}^S}\sum\left(\frac{\partial \pi_{L,0}}{\partial c_{L,0}^{S,i}}\dot{n}_L^{S,i}\right)-\phi_{L,0}\,\mathbb{R}\left(-\pi_{L,0}-\Gamma_L\right)\sum\left(\frac{\partial \pi_{L,0}}{\partial c_{L,0}^{S,i}}c_{L,0}^{S,i}\right)\right\} \\ +\frac{a_f}{\frac{W_L^T}{W_{L,0}^T}-a_f}\sum\left(\frac{\partial \pi_L}{\partial c_L^{S,i}}c_L^{S,i}\right)\phi_{L,0}\,\mathbb{R}\left(-\pi_{L,0}-\Gamma_L\right) \\ -\frac{\dot{W}_L^T}{W_L^S}\sum\left(\frac{\partial \pi_L}{\partial c_L^{S,i}}c_L^{S,i}\right)\end{array}\right\}}{1-\frac{1}{1-a_{f,0}}\frac{W_{L,0}^S}{W_L^S}\frac{\partial a_f}{\partial \pi_L}\sum\left(\frac{\partial \pi_L}{\partial c_L^{S,i}}c_L^{S,i}\right)} \qquad \text{Eq. S4.3.9a}$$

$$\Rightarrow \dot{\pi}_L = \frac{\left\{\begin{array}{c}\left[\frac{\partial \pi_L}{\partial T_L^K}+\frac{a_f}{\frac{W_L^T}{W_{L,0}^T}-a_f}\frac{\frac{1}{1-a_{f,0}}\frac{\partial a_{f,0}}{\partial \pi_{L,0}}-\frac{1}{\varepsilon_{L,0}}}{1-\frac{1}{\varepsilon_{L,0}}\sum\left(\frac{\partial \pi_{L,0}}{\partial c_{L,0}^{S,i}}c_{L,0}^{S,i}\right)}\sum\left(\frac{\partial \pi_L}{\partial c_L^{S,i}}c_L^{S,i}\right)\frac{\partial \pi_{L,0}}{\partial T_L^K}\right]\dot{T}_L^K \\ +\frac{1}{W_L^S}\sum\left(\frac{\partial \pi_L}{\partial c_L^{S,i}}\dot{n}_L^{S,i}\right) \\ +\frac{a_f}{\frac{W_L^T}{W_{L,0}^T}-a_f}\frac{\frac{1}{1-a_{f,0}}\frac{\partial a_{f,0}}{\partial \pi_{L,0}}-\frac{1}{\varepsilon_{L,0}}}{1-\frac{1}{\varepsilon_{L,0}}\sum\left(\frac{\partial \pi_{L,0}}{\partial c_{L,0}^{S,i}}c_{L,0}^{S,i}\right)}\frac{1}{W_{L,0}^S}\sum\left(\frac{\partial \pi_L}{\partial c_L^{S,i}}c_L^{S,i}\right)\sum\left(\frac{\partial \pi_{L,0}}{\partial c_{L,0}^{S,i}}\dot{n}_L^{S,i}\right) \\ +\frac{a_f}{\frac{W_L^T}{W_{L,0}^T}-a_f}\left[1-\frac{\frac{1}{1-a_{f,0}}\frac{\partial a_{f,0}}{\partial \pi_{L,0}}-\frac{1}{\varepsilon_{L,0}}}{1-\frac{1}{\varepsilon_{L,0}}\sum\left(\frac{\partial \pi_{L,0}}{\partial c_{L,0}^{S,i}}c_{L,0}^{S,i}\right)}\sum\left(\frac{\partial \pi_{L,0}}{\partial c_{L,0}^{S,i}}c_{L,0}^{S,i}\right)\right]\sum\left(\frac{\partial \pi_L}{\partial c_L^{S,i}}c_L^{S,i}\right)\phi_{L,0}\,\mathbb{R}\left(-\pi_{L,0}-\Gamma_L\right) \\ -\frac{\dot{W}_L^T}{W_L^S}\sum\left(\frac{\partial \pi_L}{\partial c_L^{S,i}}c_L^{S,i}\right)\end{array}\right\}}{1-\frac{1}{1-a_{f,0}}\frac{W_{L,0}^S}{W_L^S}\frac{\partial a_f}{\partial \pi_L}\sum\left(\frac{\partial \pi_L}{\partial c_L^{S,i}}c_L^{S,i}\right)} \qquad \text{Eq. S4.3.9b}$$

If all osmolytes behave the Van't Hoff rule (i.e., $\pi_L = -\varpi R T_L^K \sum c_L^{S,i}$, where $R = 8.314\ \mathrm{J\cdot mol^{-1}\cdot K^{-1}}$ is the universal gas constant, and $\varpi = 10^{-6}\mathrm{MPa\cdot Pa^{-1}}$ is a factor that converts from units of Pascals to

Megapascals), then $\frac{\partial \pi_L}{\partial c_L^{S,i}} = \frac{\partial \pi_{L,0}}{\partial c_{L,0}^{S,i}} = -\varpi R T_L^K$, $\frac{\partial \pi_L}{\partial T_L^K} = \frac{\pi_L}{T_L^K}$, $\frac{\partial \pi_{L,0}}{\partial T_L^K} = \frac{\pi_{L,0}}{T_L^K}$, $\sum\left(\frac{\partial \pi_{L,0}}{\partial c_{L,0}^{S,i}} c_{L,0}^{S,i}\right) = \pi_{L,0}$, $\sum\left(\frac{\partial \pi_L}{\partial c_L^{S,i}} c_L^{S,i}\right) = \pi_L$, and $\pi_{L,0} = \pi_L \frac{W_L^S}{W_{L,0}^S}$. Through these expression, Eq. S4.3.9b simplifies to

$$\dot{\pi}_L = \frac{\left\{\begin{matrix} \frac{\pi_L}{T_L^K}\left[1+\frac{a_f \pi_{L,0}}{\frac{W_L^T}{W_{L,0}^T}-a_f}\frac{\frac{1}{1-a_{f,0}}\frac{\partial a_{f,0}}{\partial \pi_{L,0}}-\frac{1}{\varepsilon_{L,0}}}{1-\frac{\pi_{L,0}}{\varepsilon_{L,0}}}\right]\dot{T}_L^K \\ -\frac{\varpi R T_L^K}{W_L^S}\left[1+\frac{a_f \pi_{L,0}}{\frac{W_L^T}{W_{L,0}^T}-a_f}\frac{\frac{1}{1-a_{f,0}}\frac{\partial a_{f,0}}{\partial \pi_{L,0}}-\frac{1}{\varepsilon_{L,0}}}{1-\frac{\pi_{L,0}}{\varepsilon_{L,0}}}\right]\sum\left(\dot{n}_L^{S,i}\right) \\ +\frac{a_f \pi_L}{\frac{W_L^T}{W_{L,0}^T}-a_f}\left[1-\pi_{L,0}\frac{\frac{1}{1-a_{f,0}}\frac{\partial a_{f,0}}{\partial \pi_{L,0}}-\frac{1}{\varepsilon_{L,0}}}{1-\frac{\pi_{L,0}}{\varepsilon_{L,0}}}\right]\phi_{L,0}\,\mathbb{R}\left(-\pi_{L,0}-\Gamma_L\right) \\ -\pi_L\frac{\dot{W}_L^T}{W_L^S} \end{matrix}\right\}}{1-\frac{\pi_L}{1-a_{f,0}}\frac{W_{L,0}^S}{W_L^S}\frac{\partial a_f}{\partial \pi_L}}. \quad \text{Eq. S4.3.10}$$

In the following subsection (Section S4.4), we assume that Van't Hoff rule is valid and thus apply Eq. S4.3.10 instead of Eq. S4.3.9c. This assumption is reasonable since, although the total bulk osmolarity of the leaf symplast may be large, the concentrations of individual osmolyte species in the leaf symplast are small (Fu et al., 2011), even in plants that passively load their phloem. Hence, the nonlinear portion of the π_L-$c_L^{S,i}$ relationships are avoided (Hall & Minchin, 2013), and the linear formulation given by the Van't Hoff rule is valid.

S4.4. Final solution for the *marginal carbon cost of water* with constrained osmotic potential

We apply the chain-rule to our definition of the *marginal carbon cost of water* from Section S1 (Eq. S1.10), differentiating Eq. S4.1.14, Eq. S4.1.15, and Eq. S4.1.23 with respect to E and A_n, to solve for χ_w with osmotic potential as the sole foliar *constraint* as

$$\chi_w^{\pi_L} = -\frac{\frac{\partial \dot{\pi}_L}{\partial E}}{\frac{\partial \dot{\pi}_L}{\partial A_n}} = -\frac{\frac{\partial \dot{\pi}_L}{\partial \dot{W}_L^T}\frac{\partial \dot{W}_L^T}{\partial E}+\frac{\partial \dot{\pi}_L}{\partial \dot{T}_L^K}\frac{\partial \dot{T}_L^K}{\partial E}}{\frac{\partial \dot{\pi}_L}{\partial \dot{n}_L^{S,A_n}}\frac{\partial \dot{n}_L^{S,A_n}}{\partial A_n}} \quad \text{Eq. S4.4.1a}$$

$$\Rightarrow \chi_w^{\pi_L} = \alpha m_w \frac{\frac{1}{\rho_w}\frac{\partial \dot{\pi}_L}{\partial \dot{W}_L^T}+\frac{\mathrm{SLA}}{a_L}\cdot\frac{\Lambda_E}{\mathrm{SWC}_L\cdot\mathrm{RWC}_L^T\cdot c_w+c_{DM}}\frac{\partial \dot{\pi}_L}{\partial \dot{T}_L^K}}{\frac{\partial \dot{\pi}_L}{\partial \dot{n}_L^{S,A_n}}} \quad \text{Eq. S4.4.1b}$$

$$\Rightarrow \chi_w^{\pi_L} = \alpha m_w \frac{\frac{1}{\rho_w}\frac{\partial \dot{\pi}_L}{\partial \dot{W}_L^T}+\frac{1}{M_{DM}}\cdot\frac{\Lambda_E}{\mathrm{SWC}_L\cdot\mathrm{RWC}_L^T\cdot c_w+c_{DM}}\frac{\partial \dot{\pi}_L}{\partial \dot{T}_L^K}}{\frac{\partial \dot{\pi}_L}{\partial \dot{n}_L^{S,A_n}}}, \quad \text{Eq. S4.4.1c}$$

where $M_{DM} = \frac{a_L}{\mathrm{SLA}}$ is the leaf dry matter mass [kg]. Since the terms related to plastic expansion (i.e., $\phi_{L,0}, \Gamma_L$) in Eq. S.3.10 are not multiplied by either $\dot{W}_L^T$, $\dot{T}_L^K$, or $\dot{n}_L^{S,A_n}$, leaf expansion does not affect χ_w and stomatal behavior, except independently through dynamic changes in osmotic potential (Eq. S4.3.10) and its value at full hydration (Eq. S4.2.4 & Eq. S4.2.5). We differentiate Eq. S4.3.10 with respect to $\dot{W}_L^T$, $\dot{T}_L^K$, and $\dot{n}_L^{S,A_n}$ and plug into Eq. S4.4.1, simplifying through $\rho_w W_{L,0}^T = \mathrm{SWC}_L M_{DM}$, to define χ_w as

$$\chi_w^{\pi_L} = -\frac{\alpha m_w \pi_L}{\varpi R T_L^K}\left\{\frac{W_L^S}{M_{DM}}\frac{1}{\mathrm{SWC}_L \cdot \mathrm{RWC}_L^T \cdot c_w + c_{DM}} \cdot \frac{\Lambda_E}{T_L^K} - \frac{1}{\rho_w}\left[1 + \frac{a_f \pi_{L,0}}{\mathrm{RWC}_L^T - a_f}\frac{\frac{1}{1-a_{f,0}}\frac{\partial a_{f,0}}{\partial \pi_{L,0}} - \frac{1}{\varepsilon_{L,0}}}{1-\frac{\pi_{L,0}}{\varepsilon_{L,0}}}\right]^{-1}\right\} \quad \text{Eq. S4.4.2a}$$

$$\Rightarrow \chi_w^{\pi_L} = -\left(\mathrm{RWC}_L^T - a_f\right)\frac{\alpha m_w \pi_L}{\rho_w \varpi R T_L^K}\left\{\frac{\mathrm{SWC}_L}{\mathrm{SWC}_L \cdot \mathrm{RWC}_L^T \cdot c_w + c_{DM}} \cdot \frac{\Lambda_E}{T_L^K} - \left[\left(\mathrm{RWC}_L^T - a_f\right) + a_f \pi_{L,0}\frac{\frac{1}{1-a_{f,0}}\frac{\partial a_{f,0}}{\partial \pi_{L,0}} - \frac{1}{\varepsilon_{L,0}}}{1-\frac{\pi_{L,0}}{\varepsilon_{L,0}}}\right]^{-1}\right\} \quad \text{Eq. S4.4.2b}$$

$$\Rightarrow \chi_w^{\pi_L} = -\left(\mathrm{RWC}_L^T - a_f\right)\frac{\alpha m_w \pi_L}{\rho_w \varpi R T_L^K}\left\{\frac{\mathrm{SWC}_L}{\mathrm{SWC}_L \cdot \mathrm{RWC}_L^T \cdot c_w + c_{DM}} \cdot \frac{\Lambda_E}{T_L^K} - \left[\mathrm{RWC}_L^T + \frac{a_f \varepsilon_{L,0}}{\varepsilon_{L,0} - \pi_{L,0}}\left(\frac{\pi_{L,0}}{1-a_{f,0}}\frac{\partial a_{f,0}}{\partial \pi_{L,0}} - 1\right)\right]^{-1}\right\}. \quad \text{Eq. S4.4.2c}$$

$$\Rightarrow \chi_w^{\pi_L} = -\left(\mathrm{RWC}_L^T - a_f\right)\frac{\alpha m_w \pi_L}{\rho_w \varpi R T_L^K}\left[\frac{\mathrm{SWC}_L}{\mathrm{SWC}_L \cdot \mathrm{RWC}_L^T \cdot c_w + c_{DM}} \cdot \frac{\Lambda_E}{T_L^K} - \frac{1}{\mathrm{RWC}_L^T + a_f \Delta}\right] \quad \text{Eq. S4.4.2d}$$

$$\Rightarrow \chi_w^{\pi_L} = -\left(1 - a_{f,0}\right)\frac{\alpha m_w \pi_{L,0}}{\rho_w \varpi R T_L^K}\left[\frac{\mathrm{SWC}_L}{\mathrm{SWC}_L \cdot \mathrm{RWC}_L^T \cdot c_w + c_{DM}} \cdot \frac{\Lambda_E}{T_L^K} - \frac{1}{\mathrm{RWC}_L^T + a_f \Delta}\right], \quad \text{Eq. S4.4.2d}$$

where Δ is unitless shorthand for

$$\Delta = \frac{\varepsilon_{L,0}}{\varepsilon_{L,0} - \pi_{L,0}}\left(\frac{\pi_{L,0}}{1-a_{f,0}}\frac{\partial a_{f,0}}{\partial \pi_{L,0}} - 1\right). \quad \text{Eq. S4.4.3}$$

Given our use of a Weibull function to describe the π_L-a_f relationship (Eq. S4.1.2 and Eq. S4.1.4) and $\pi_{L,0}$-$a_{f,0}$ relationship (Eq. S4.1.3 and Eq. S4.1.5), Eq. S4.4.3 simplifies to

$$\Delta = -\frac{\varepsilon_{L,0}}{\varepsilon_{L,0} - \pi_{L,0}}\left[\frac{\beta a_{f,0}}{1-a_{f,0}}\left(\frac{\pi_{L,0}}{\pi_L^*}\right)^{\beta} + 1\right]. \quad \text{Eq. S4.4.4}$$

Since (1) $\chi_w^{\pi_L}$ must be positive for realistic stomatal behavior, (2) the terms outside of the square brackets on the righthand side of Eq. S4.4.2d are positive ($-\pi_{L,0} > 0$, since $\pi_{L,0}$ is negative), and (3) both quotients inside the square brackets ($\frac{\mathrm{SWC}_L}{\mathrm{SWC}_L \cdot \mathrm{RWC}_L^T \cdot c_w + c_{DM}} \cdot \frac{\Lambda_E}{T_L^K}$ & $-\frac{1}{\mathrm{RWC}_L^T + a_f \Delta}$) are positive and negative, respectively, for realistic PV trait values, it is apparent that the cooling effects of transpiration that cause π_L to increase (represented by $\frac{\mathrm{SWC}_L}{\mathrm{SWC}_L \cdot \mathrm{RWC}_L^T \cdot c_w + c_{DM}} \cdot \frac{\Lambda_E}{T_L^K}$ in Eq. S4.4.2d) are larger than and opposite in sign to the concentrating effects of transpiration that cause π_L to decrease (represented by $-\frac{1}{\mathrm{RWC}_L^T + a_f \Delta}$ in Eq. S4.4.2d).

Eq. S4.4.2 suggest that the optimal stomatal conductance as represented by the *marginal carbon cost of water*, χ_w, is controlled primarily and immediately by the leaf total water potential, ψ_L, which affects the RWC_L^S, RWC_L^T, and ε_L terms in Eq. S4.5.2. The *marginal cost* also experiences slower responses to environmental perturbations upon acclimation of the osmotic potential at full hydration, $\pi_{L,0}$, which itself is dynamic (Eq. S4.2.4 and Eq. S4.2.5), although changes during drought events are expected to be generally small (average ~16% among species; Bartlett et al., 2014). The osmotic potential at full hydration is immediately present in Eq. S4.5.2 and Eq. S4.5.3, but it also indirectly affects χ_w by influencing the relationship between pressure and leaf volume (i.e., RWC_L^S, RWC_L^T, and ε_L for a given ψ_L) as well as through the $a_{f,0}$ and $\varepsilon_{L,0}$ terms in Eq. S4.5.2 and Eq. S4.5.3, which depend on $\pi_{L,0}$ (Eq. S4.1.3 and Eq. S4.1.16). While ψ_L represents a control on the *marginal cost* by xylem hydraulics, the additional role of $\pi_{L,0}$ emphasizes osmoregulation, phloem sugar transport, and foliar sugar-starch metabolism.

S4.5. The *marginal carbon cost of water* with constrained turgor pressure

In this section, we derive an alternative definition for χ_w in which the turgor pressure is the sole foliar *constraint*, instead of the osmotic potential. Analogous to Eq. S4.4.1,

$$\chi_w^{P_L} = -\frac{\frac{\partial \dot{P}_L}{\partial E}}{\frac{\partial \dot{P}_L}{\partial A_n}} = -\frac{\frac{\partial \dot{P}_L}{\partial \dot{W}_L^T}\frac{\partial \dot{W}_L^T}{\partial E} + \frac{\partial \dot{P}_L}{\partial \dot{T}_L^K}\frac{\partial \dot{T}_L^K}{\partial E}}{\frac{\partial \dot{P}_L}{\partial \dot{n}_L^{S,An}}\frac{\partial \dot{n}_L^{S,An}}{\partial A_n}}. \qquad \text{Eq. S4.5.1a}$$

$$\Rightarrow \chi_w^{P_L} = \alpha m_w \frac{\frac{1}{\rho_w}\frac{\partial \dot{P}_L}{\partial \dot{W}_L^T} + \frac{\mathrm{SLA}}{a_L}\cdot\frac{\Lambda_E}{\mathrm{SWC}_L\cdot \mathrm{RWC}_L^T\cdot c_w + c_{DM}}\frac{\partial \dot{P}_L}{\partial \dot{T}_L^K}}{\frac{\partial \dot{P}_L}{\partial \dot{n}_L^{S,An}}} \qquad \text{Eq. S4.5.1b}$$

$$\Rightarrow \chi_w^{P_L} = \alpha m_w \frac{\frac{1}{\rho_w}\frac{\partial \dot{P}_L}{\partial \dot{W}_L^T} + \frac{1}{M_{DM}}\cdot\frac{\Lambda_E}{\mathrm{SWC}_L\cdot \mathrm{RWC}_L^T\cdot c_w + c_{DM}}\frac{\partial \dot{P}_L}{\partial \dot{T}_L^K}}{\frac{\partial \dot{P}_L}{\partial \dot{n}_L^{S,An}}}, \qquad \text{Eq. S4.5.1c}$$

Evaluating Eq, S4.5.1 requires an equation for the change in leaf turgor, $\dot{P}_L$, in terms of gas exchange variables. We begin by finding an expression for $\dot{P}_L$ based on Hooke's law and the Lockhart equation similar to Eq. S4.1.13,

$$\dot{W}_L^S = \frac{W_L^S}{\varepsilon_L}\dot{P}_L + W_L^S\phi_L\,\mathbb{R}(P_L - \Gamma_L) \qquad \text{Eq. S4.5.2a}$$

$$\Rightarrow \dot{P}_L = \varepsilon_L\frac{\dot{W}_L^S}{W_L^S} - \varepsilon_L\phi_L\,\mathbb{R}(P_L - \Gamma_L), \qquad \text{Eq. S4.5.2b}$$

where ϕ_L is the extensibility [$\mathrm{MPa}^{-1}\cdot\mathrm{s}^{-1}$]. We plug Eq. S4.3.4b into Eq. S4.5.2b and simplify,

$$\dot{P}_L = \frac{\varepsilon_L}{W_L^S}\left\{\begin{matrix} \dot{W}_L^T - a_f W_{L,0}^T\left(\frac{1}{1-a_{f,0}}\frac{\partial a_{f,0}}{\partial \pi_{L,0}} - \frac{1}{\varepsilon_{L,0}}\right)\dot{\pi}_{L,0} \\ -\frac{W_{L,0}^S}{1-a_{f,0}}\frac{\partial a_f}{\partial \pi_L}\dot{\pi}_L - a_f W_{L,0}^T\phi_{L,0}\,\mathbb{R}\left(-\pi_{L,0} - \Gamma_L\right) \end{matrix}\right\} - \varepsilon_L\phi_L\,\mathbb{R}(P_L - \Gamma_L) \qquad \text{Eq. S4.5.3a}$$

$$\Rightarrow \dot{P}_L = \varepsilon_L\left\{\begin{matrix} \frac{\dot{W}_L^T}{W_L^S} - \frac{a_f}{\frac{W_L^T}{W_{L,0}^T} - a_f}\left(\frac{1}{1-a_{f,0}}\frac{\partial a_{f,0}}{\partial \pi_{L,0}} - \frac{1}{\varepsilon_{L,0}}\right)\dot{\pi}_{L,0} \\ -\frac{W_{L,0}^S}{W_L^S}\frac{1}{1-a_{f,0}}\frac{\partial a_f}{\partial \pi_L}\dot{\pi}_L \\ -\frac{a_f}{\frac{W_L^T}{W_{L,0}^T} - a_f}\phi_{L,0}\,\mathbb{R}\left(-\pi_{L,0} - \Gamma_L\right) - \phi_L\,\mathbb{R}(P_L - \Gamma_L) \end{matrix}\right\}. \text{Eq. S4.5.3b}$$

We plug Eq. S4.2.4b and Eq. S4.3.9b into Eq. S4.5.3b and simplify,

$$\dot{P}_L = \varepsilon_L \left\{ \begin{gathered} \frac{\dot{W}_L^T}{W_L^S} - \frac{a_f}{\frac{W_L^T}{W_{L,0}^T} - a_f} \frac{\frac{1}{1-a_{f,0}}\frac{\partial a_{f,0}}{\partial \pi_{L,0}} - \frac{1}{\varepsilon_{L,0}}}{1 - \frac{1}{\varepsilon_{L,0}} \sum\left(\frac{\partial \pi_{L,0}}{\partial c_{L,0}^{S,i}} c_{L,0}^{S,i}\right)} \left[\begin{gathered} \frac{\partial \pi_{L,0}}{\partial T_L^K} \dot{T}_L^K + \frac{1}{W_{L,0}^S} \sum \left(\frac{\partial \pi_{L,0}}{\partial c_{L,0}^{S,i}} \dot{n}_L^{S,i}\right) \\ -\phi_{L,0}\, \mathbb{R}(-\pi_{L,0} - \Gamma_L) \sum \left(\frac{\partial \pi_{L,0}}{\partial c_{L,0}^{S,i}} c_{L,0}^{S,i}\right) \end{gathered} \right] \\ - \frac{\frac{W_{L,0}^S}{W_L^S} \frac{1}{1-a_{f,0}} \frac{\partial a_f}{\partial \pi_L}}{1 - \frac{1}{1-a_{f,0}} \frac{W_{L,0}^S}{W_L^S} \frac{\partial a_f}{\partial \pi_L} \sum\left(\frac{\partial \pi_L}{\partial c_L^{S,i}} c_L^{S,i}\right)} \left[\begin{gathered} \left[\frac{\partial \pi_L}{\partial T_L^K} + \frac{a_f}{\frac{W_L^T}{W_{L,0}^T} - a_f} \frac{\frac{1}{1-a_{f,0}}\frac{\partial a_{f,0}}{\partial \pi_{L,0}} - \frac{1}{\varepsilon_{L,0}}}{1 - \frac{1}{\varepsilon_{L,0}} \sum\left(\frac{\partial \pi_{L,0}}{\partial c_{L,0}^{S,i}} c_{L,0}^{S,i}\right)} \sum \left(\frac{\partial \pi_L}{\partial c_L^{S,i}} c_L^{S,i}\right) \frac{\partial \pi_{L,0}}{\partial T_L^K} \right] \dot{T}_L^K \\ + \frac{1}{W_L^S} \sum \left(\frac{\partial \pi_L}{\partial c_L^{S,i}} \dot{n}_L^{S,i}\right) \\ + \frac{a_f}{\frac{W_L^T}{W_{L,0}^T} - a_f} \frac{\frac{1}{1-a_{f,0}}\frac{\partial a_{f,0}}{\partial \pi_{L,0}} - \frac{1}{\varepsilon_{L,0}}}{1 - \frac{1}{\varepsilon_{L,0}} \sum\left(\frac{\partial \pi_{L,0}}{\partial c_{L,0}^{S,i}} c_{L,0}^{S,i}\right)} \frac{1}{W_{L,0}^S} \sum \left(\frac{\partial \pi_L}{\partial c_L^{S,i}} c_L^{S,i}\right) \sum \left(\frac{\partial \pi_{L,0}}{\partial c_{L,0}^{S,i}} \dot{n}_L^{S,i}\right) \\ + \frac{a_f}{\frac{W_L^T}{W_{L,0}^T} - a_f} \left[1 - \frac{\frac{1}{1-a_{f,0}}\frac{\partial a_{f,0}}{\partial \pi_{L,0}} - \frac{1}{\varepsilon_{L,0}}}{1 - \frac{1}{\varepsilon_{L,0}} \sum\left(\frac{\partial \pi_{L,0}}{\partial c_{L,0}^{S,i}} c_{L,0}^{S,i}\right)} \sum \left(\frac{\partial \pi_{L,0}}{\partial c_{L,0}^{S,i}} c_{L,0}^{S,i}\right) \right] \sum \left(\frac{\partial \pi_L}{\partial c_L^{S,i}} c_L^{S,i}\right) \phi_{L,0}\, \mathbb{R}(-\pi_{L,0} - \Gamma_L) \\ - \frac{\dot{W}_L^T}{W_L^S} \sum \left(\frac{\partial \pi_L}{\partial c_L^{S,i}} c_L^{S,i}\right) \end{gathered} \right] \\ - \frac{a_f}{\frac{W_L^T}{W_{L,0}^T} - a_f} \phi_{L,0}\, \mathbb{R}(-\pi_{L,0} - \Gamma_L) - \phi_L\, \mathbb{R}(P_L - \Gamma_L) \end{gathered} \right\} \quad \text{Eq. S4.5.4a}$$

$$\Rightarrow \dot{P}_L = \varepsilon_L \left\{ \begin{gathered} \left[1 + \Xi \sum \left(\frac{\partial \pi_L}{\partial c_L^{S,i}} c_L^{S,i}\right)\right] \frac{\dot{W}_L^T}{W_L^S} \\ - \left\{ \Xi \frac{\partial \pi_L}{\partial T_L^K} + \frac{a_f \Theta}{\frac{W_L^T}{W_{L,0}^T} - a_f} \left[1 + \Xi \sum \left(\frac{\partial \pi_L}{\partial c_L^{S,i}} c_L^{S,i}\right)\right] \frac{\partial \pi_{L,0}}{\partial T_L^K} \right\} \dot{T}_L^K \\ - \frac{a_f \Theta}{\frac{W_L^T}{W_{L,0}^T} - a_f} \frac{1}{W_{L,0}^S} \left[1 + \Xi \sum \left(\frac{\partial \pi_L}{\partial c_L^{S,i}} c_L^{S,i}\right)\right] \sum \left(\frac{\partial \pi_{L,0}}{\partial c_{L,0}^{S,i}} \dot{n}_L^{S,i}\right) \\ - \frac{\Xi}{W_L^S} \sum \left(\frac{\partial \pi_L}{\partial c_L^{S,i}} \dot{n}_L^{S,i}\right) \\ + \frac{a_f}{\frac{W_L^T}{W_{L,0}^T} - a_f} \left\{ \Theta \sum \left(\frac{\partial \pi_{L,0}}{\partial c_{L,0}^{S,i}} c_{L,0}^{S,i}\right) - \Xi \left[1 - \Theta \sum \left(\frac{\partial \pi_{L,0}}{\partial c_{L,0}^{S,i}} c_{L,0}^{S,i}\right)\right] \sum \left(\frac{\partial \pi_L}{\partial c_L^{S,i}} c_L^{S,i}\right) - 1 \right\} \phi_{L,0}\, \mathbb{R}(-\pi_{L,0} - \Gamma_L) \\ - \phi_L\, \mathbb{R}(P_L - \Gamma_L) \end{gathered} \right\}, \quad \text{Eq. S4.5.4b}$$

where Θ [MPa^{-1}] and Ξ [MPa^{-1}] are shorthand for

$$\Theta = \frac{\frac{1}{1-a_{f,0}}\frac{\partial a_{f,0}}{\partial \pi_{L,0}} - \frac{1}{\varepsilon_{L,0}}}{1 - \frac{1}{\varepsilon_{L,0}} \sum\left(\frac{\partial \pi_{L,0}}{\partial c_{L,0}^{S,i}} c_{L,0}^{S,i}\right)} \quad \text{Eq. S4.5.5}$$

$$\Xi = \frac{\frac{1}{1-a_{f,0}} \frac{W_{L,0}^S}{W_L^S} \frac{\partial a_f}{\partial \pi_L}}{1 - \frac{1}{1-a_{f,0}} \frac{W_{L,0}^S}{W_L^S} \frac{\partial a_f}{\partial \pi_L} \sum\left(\frac{\partial \pi_L}{\partial c_L^{S,i}} c_L^{S,i}\right)}. \quad \text{Eq. S4.5.6}$$

If all osmolytes behave the Van't Hoff rule, then Eq. S4.5.4 simplifies to

$$\dot{P}_L = \varepsilon_L \left\{ \begin{array}{c} (1+\Xi\pi_L)\dfrac{\dot{W}_L^T}{W_L^S} \\ -\dfrac{\pi_L}{T_L^K}\left[\Xi + \dfrac{a_f\Theta}{\frac{W_L^T}{W_{L,0}^T}-a_f}\dfrac{W_L^S}{W_{L,0}^S}(1+\Xi\pi_L)\right]\dot{T}_L^K \\ +\dfrac{\varpi R T_L^K}{W_L^S}\left[\Xi + \dfrac{a_f\Theta}{\frac{W_L^T}{W_{L,0}^T}-a_f}\dfrac{W_L^S}{W_{L,0}^S}(1+\Xi\pi_L)\right]\sum\left(\dot{n}_L^{S,i}\right) \\ +\dfrac{a_f}{\frac{W_L^T}{W_{L,0}^T}-a_f}\left(\Theta\pi_{L,0}-1\right)(1+\Xi\pi_L)\phi_{L,0}\,\mathbb{R}\left(-\pi_{L,0}-\Gamma_L\right) \\ -\phi_L\,\mathbb{R}(P_L-\Gamma_L) \end{array} \right\} \qquad \text{Eq. S4.5.7a}$$

$$\Rightarrow \dot{P}_L = \varepsilon_L \left\{ \begin{array}{c} \dfrac{1}{1-\frac{\pi_L}{1-a_{f,0}}\frac{W_{L,0}^S}{W_L^S}\frac{\partial a_f}{\partial \pi_L}}\dfrac{\dot{W}_L^T}{W_L^S} \\ -\dfrac{\pi_L}{T_L^K}\dfrac{\frac{1}{1-a_{f,0}}\frac{W_{L,0}^S}{W_L^S}\frac{\partial a_f}{\partial \pi_L}+\frac{a_f\Theta}{1-a_{f,0}}}{1-\frac{\pi_L}{1-a_{f,0}}\frac{W_{L,0}^S}{W_L^S}\frac{\partial a_f}{\partial \pi_L}}\dot{T}_L^K \\ +\dfrac{\varpi R T_L^K}{W_L^S}\dfrac{\frac{1}{1-a_{f,0}}\frac{W_{L,0}^S}{W_L^S}\frac{\partial a_f}{\partial \pi_L}+\frac{a_f\Theta}{1-a_{f,0}}}{1-\frac{\pi_L}{1-a_{f,0}}\frac{W_{L,0}^S}{W_L^S}\frac{\partial a_f}{\partial \pi_L}}\sum\left(\dot{n}_L^{S,i}\right) \\ +\dfrac{a_f}{\frac{W_L^T}{W_{L,0}^T}-a_f}\left(\Theta\pi_{L,0}-1\right)(1+\Xi\pi_L)\phi_{L,0}\,\mathbb{R}\left(-\pi_{L,0}-\Gamma_L\right) \\ -\phi_L\,\mathbb{R}(P_L-\Gamma_L) \end{array} \right\}. \qquad \text{Eq. S4.5.7b}$$

Differentiating Eq. S.4.5.7b, plugging into Eq. S4.5.1c, and simplifying through our Δ notation (Eq. S4.4.3), χ_w in which the turgor pressure is the sole foliar *constraint* is

$$\chi_w^{P_L} = -\left(1-a_{f,0}\right)\frac{\alpha m_w \pi_L}{\rho_w \varpi R T_L^K}\left[\frac{\mathrm{SWC}_L\cdot \mathrm{RWC}_L^S}{\mathrm{SWC}_L\cdot \mathrm{RWC}_L^T\cdot c_w + c_{DM}}\cdot\frac{\Lambda_E}{T_L^K} - \frac{1}{\pi_L}\left(\frac{1}{\mathrm{RWC}_L^S}\frac{\partial a_f}{\partial \pi_L} + a_f\Theta\right)^{-1}\right] \qquad \text{Eq. S4.5.8a}$$

$$\chi_w^{P_L} = -\left(1-a_{f,0}\right)\frac{\alpha m_w \pi_{L,0}}{\rho_w \varpi R T_L^K}\left\{\frac{\mathrm{SWC}_L}{\mathrm{SWC}_L\cdot \mathrm{RWC}_L^T\cdot c_w + c_{DM}}\cdot\frac{\Lambda_E}{T_L^K} - \left[\pi_L\frac{\partial a_f}{\partial \pi_L} + a_f\left(1+\frac{\varepsilon_{L,0}}{\varepsilon_{L,0}-\pi_{L,0}}\left(\frac{\pi_{L,0}}{1-a_{f,0}}\frac{\partial a_{f,0}}{\partial \pi_{L,0}}-1\right)\right)\right]^{-1}\right\}, \qquad \text{Eq. S4.5.8b}$$

$$\chi_w^{P_L} = -\left(1-a_{f,0}\right)\frac{\alpha m_w \pi_{L,0}}{\rho_w \varpi R T_L^K}\left[\frac{\mathrm{SWC}_L}{\mathrm{SWC}_L\cdot \mathrm{RWC}_L^T\cdot c_w + c_{DM}}\cdot\frac{\Lambda_E}{T_L^K} - \frac{1}{\pi_L\frac{\partial a_f}{\partial \pi_L} + a_f(1+\Delta)}\right], \qquad \text{Eq. S4.5.8c}$$

which is functionally almost identical to χ_w for the case with osmotic potential as the sole foliar *constraint* (Eq. S4.4.2d), except for the final term in square brackets in Eq. S4.5.8c. The temperature response, controlled by the first term in the square brackets, is identical to that for the case with osmotic potential as the sole foliar *constraint*.

S4.6. The *marginal carbon cost of water* with constrained total water potential

In this section, we derive an alternative definition for χ_w in which the total water potential is the constraint, which we denote by $\chi_w^{\psi_L}$. It may be shown that $\chi_w^{\psi_L}$ is a weighted average of the previous

alternative definitions for χ_w in which osmotic potential and turgor are the sole foliar *constraints* ($\chi_w^{\pi_L}$ & $\chi_w^{P_L}$; Eq. S4.4.2d & Eq. S4.5.8b),

$$\chi_w^{\psi_L} = -\frac{\frac{\partial\dot{\psi}_L}{\partial E}}{\frac{\partial\dot{\psi}_L}{\partial A_n}} = -\frac{\frac{\partial\dot{\pi}_L}{\partial E}+\frac{\partial\dot{P}_L}{\partial E}}{\frac{\partial\dot{\pi}_L}{\partial A_n}+\frac{\partial\dot{P}_L}{\partial A_n}} = \frac{\frac{\partial\dot{\pi}_L}{\partial A_n}\chi_w^{\pi_L}+\frac{\partial\dot{P}_L}{\partial A_n}\chi_w^{P_L}}{\frac{\partial\dot{\pi}_L}{\partial A_n}+\frac{\partial\dot{P}_L}{\partial A_n}} \text{ Eq. S4.6.1a}$$

$$\Rightarrow \chi_w^{\psi_L} = \frac{\frac{\partial\dot{\pi}_L}{\partial\dot{n}_L^{S,An}}\frac{\partial\dot{n}_L^{S,An}}{\partial A_n}\chi_w^{\pi_L}+\frac{\partial\dot{P}_L}{\partial\dot{n}_L^{S,An}}\frac{\partial\dot{n}_L^{S,An}}{\partial A_n}\chi_w^{P_L}}{\frac{\partial\dot{\pi}_L}{\partial\dot{n}_L^{S,An}}\frac{\partial\dot{n}_L^{S,An}}{\partial A_n}+\frac{\partial\dot{P}_L}{\partial\dot{n}_L^{S,An}}\frac{\partial\dot{n}_L^{S,An}}{\partial A_n}} = \frac{\frac{\partial\dot{\pi}_L}{\partial\dot{n}_L^{S,An}}\chi_w^{\pi_L}+\frac{\partial\dot{P}_L}{\partial\dot{n}_L^{S,An}}\chi_w^{P_L}}{\frac{\partial\dot{\pi}_L}{\partial\dot{n}_L^{S,An}}+\frac{\partial\dot{P}_L}{\partial\dot{n}_L^{S,An}}} = \frac{\chi_w^{\pi_L}+\frac{\frac{\partial\dot{P}_L}{\partial\dot{n}_L^{S,An}}}{\frac{\partial\dot{\pi}_L}{\partial\dot{n}_L^{S,An}}}\chi_w^{P_L}}{1+\frac{\frac{\partial\dot{P}_L}{\partial\dot{n}_L^{S,An}}}{\frac{\partial\dot{\pi}_L}{\partial\dot{n}_L^{S,An}}}}, \quad \text{Eq. S4.6.1b}$$

where the weights are $\frac{\partial\dot{\pi}_L}{\partial\dot{n}_L^{S,An}}$ and $\frac{\partial\dot{P}_L}{\partial\dot{n}_L^{S,An}}$ for averaging $\chi_w^{\pi_L}$ and $\chi_w^{P_L}$, respectively. Differentiating Eq. S4.3.10 and Eq. S4.5.7b for $\frac{\partial\dot{\pi}_L}{\partial\dot{n}_L^{S,An}}$ and $\frac{\partial\dot{P}_L}{\partial\dot{n}_L^{S,An}}$, respectively, rearranging, and simplifying through our Δ notation (Eq. S4.4.3), the ratio of $\frac{\partial\dot{P}_L}{\partial\dot{n}_L^{S,An}}$ to $\frac{\partial\dot{\pi}_L}{\partial\dot{n}_L^{S,An}}$ is

$$\frac{\frac{\partial\dot{P}_L}{\partial\dot{n}_L^{S,An}}}{\frac{\partial\dot{\pi}_L}{\partial\dot{n}_L^{S,An}}} = -\varepsilon_L\frac{\frac{\partial a_f}{\partial\pi_L}+\frac{a_f}{\pi_L}(1+\Delta)}{\mathrm{RWC}_L^T+a_f\Delta}. \text{ Eq. S4.6.2}$$

Plugging Eq. S4.4.2d, Eq. S4.5.8c, and Eq. S4.6.2 into Eq. S4.6.1b and simplifying provides our definition for χ_w in which the total water potential as the sole foliar *constraint*,

$$\chi_w^{\psi_L} = -\left(1-a_{f,0}\right)\frac{\alpha m_w\pi_{L,0}}{\rho_w\varpi RT_L^K}\left[\frac{\mathrm{SWC}_L}{\mathrm{SWC}_L\cdot\mathrm{RWC}_L^T\cdot c_w+c_{DM}}\cdot\frac{\Lambda_E}{T_L^K}-\frac{1}{\frac{\pi_L}{\pi_L-\varepsilon_L}\left(\mathrm{RWC}_L^T-a_f-\varepsilon_L\frac{\partial a_f}{\partial\pi_L}\right)+a_f(1+\Delta)}\right]. \quad \text{Eq. S4.6.3}$$

Hence, whether the sole foliar *constraint* is the osmotic potential (S4.4.2d), turgor pressure (Eq. S4.5.8c), or total water potential (Eq. S4.6.3), the functional form of χ_w is conserved with only slight modifications to the final term inside the square brackets. The temperature response, controlled by the first term in the square brackets, is identical to those from the two previous cases with osmotic potential and turgor pressure as the sole foliar *constraint*.

S4.7. Equivalence to *instantaneously* maximizing the time-rate of change of leaf solute concentration and leaf hydration

Our optimization problem is equivalent to ***instantaneously*** maximizing or minimizing the time-derivative of the ***constraint***; however, our analyses in Section S1 do not tell us whether the time-derivative of the ***constraint*** is being maximized or minimized. In this section, we show that our optimization problem is equivalent to maximizing the time-derivative of the leaf turgor pressure, the total water potential, or the absolute value of the osmotic potential (i.e., it minimizes the time-derivative of the osmotic potential), depending on the choice of the ***constraint***. Hence, our stomatal strategy prioritizes maintaining leaf hydration and large leaf solute concentrations.

First, we show that our optimization problem is equivalent to ***instantaneously*** maximizing or minimizing the time-derivative of the ***constraint*** ($\dot{X}_1$). To ***instantaneously*** maximize or minimize $\dot{X}_1$,

$$\frac{\partial\dot{X}_1}{\partial g_w} = 0. \qquad \text{Eq. S4.7.1}$$

Eq. S4.7.1 may be expanded by the chain-rule as

$$\frac{\partial \dot{X}_1}{\partial A_n}\frac{\partial A_n}{\partial g_w} + \frac{\partial \dot{X}_1}{\partial E}\frac{\partial E}{\partial g_w} = 0, \qquad \text{Eq. S4.7.2}$$

which may be rearranged to achieve our previous solution from Section S1 (Eq. S1.9). Hence, the strategy that ***instantaneously*** maximizes or minimizes $\dot{X}_1$ is equivalent to the strategy derived in Section S1.

To answer whether $\dot{X}_1$ is being maximized or minimized, we analyze the sign of the second-derivative of $\dot{X}_1$ with respect to g_w. A positive second-derivative would imply that the stomatal strategy minimizes $\dot{X}_1$, and a negative second-derivative would imply that $\dot{X}_1$ is maximized. The second-derivative is

$$\frac{\partial^2 \dot{X}_1}{\partial {g_w}^2} = \frac{\partial}{\partial g_w}\left(\frac{\partial \dot{X}_1}{\partial A_n}\frac{\partial A_n}{\partial g_w} + \frac{\partial \dot{X}_1}{\partial E}\frac{\partial E}{\partial g_w}\right). \qquad \text{Eq. S4.7.3}$$

Since the $\frac{\partial \dot{X}_1}{\partial A_n}$ and $\frac{\partial \dot{X}_1}{\partial E}$ terms in Eq. S4.7.3 are not defined in terms of g_w, A_n, or E terms (based on Eq. S4.3.10 for $\dot{X}_1 = \dot{\pi}_L$, Eq. S4.5.7b for $\dot{X}_1 = \dot{P}_L$, and because $\dot{\psi}_L = \dot{\pi}_L + \dot{P}_L$ for $\dot{X}_1 = \dot{\psi}_L$), Eq. S4.7.3 may be rewritten as

$$\frac{\partial^2 \dot{X}_1}{\partial {g_w}^2} = \frac{\partial \dot{X}_1}{\partial A_n}\frac{\partial^2 A_n}{\partial {g_w}^2} + \frac{\partial \dot{X}_1}{\partial E}\frac{\partial^2 E}{\partial {g_w}^2}, \qquad \text{Eq. S4.7.4}$$

which may be further simplified, since $\frac{\partial^2 E}{\partial {g_w}^2} = 0$ (Eq. S3.3.1), as

$$\frac{\partial^2 \dot{X}_1}{\partial {g_w}^2} = \frac{\partial \dot{X}_1}{\partial A_n}\frac{\partial^2 A_n}{\partial {g_w}^2}. \quad \text{Eq. S4.7.5}$$

The sign of $\frac{\partial^2 A_n}{\partial {g_w}^2}$ is negative, because A_n is a saturating sigmoidal function of g_w, and so the sign of the second-derivative is

$$\operatorname{sgn}\left(\frac{\partial^2 \dot{X}_1}{\partial {g_w}^2}\right) = -\operatorname{sgn}\left(\frac{\partial \dot{X}_1}{\partial A_n}\right), \qquad \text{Eq. S4.7.6}$$

the identity of which depends on the choice of ***constraint***.

For constrained osmotic potential, $\frac{\partial \dot{\pi}_L}{\partial A_n}$ is determined by Eq. S4.1.15 and Eq. S4.3.10,

$$\frac{\partial \dot{\pi}_L}{\partial A_n} = \frac{\partial \dot{\pi}_L}{\partial \dot{n}_L^{S,A_n}}\frac{\partial \dot{n}_L^{S,A_n}}{\partial A_n} = -\frac{a_L \varpi R T_L^K}{\alpha W_L^S}\frac{1+\frac{a_f \pi_{L,0}}{\mathrm{RWC}_L^T - a_f}\frac{\frac{1}{1-a_{f,0}}\frac{\partial a_{f,0}}{\partial \pi_{L,0}} - \frac{1}{\varepsilon_{L,0}}}{1-\frac{\pi_{L,0}}{\varepsilon_{L,0}}}}{1-\frac{1}{1-a_{f,0}}\frac{\pi_L}{\mathrm{RWC}_L^S}\frac{\partial a_f}{\partial \pi_L}}. \qquad \text{Eq. S4.7.7}$$

For realistic parameters that define the shape of the a_f-π_L relationship (positive β, negative π_L^*), the $\frac{\partial a_f}{\partial \pi_L}$ and $\frac{\partial a_{f,0}}{\partial \pi_{L,0}}$ terms in Eq. S4.7.7 are positive (Eq. S4.1.4 & Eq. S4.1.5) Hence, the sign of $\frac{\partial \dot{\pi}_L}{\partial A_n}$ may be simplified, by ignoring the positive multiplicative terms, which do not affect the sign, to

$$\operatorname{sgn}\left(\frac{\partial \dot{\pi}_L}{\partial A_n}\right) = -\operatorname{sgn}\left(1 + \frac{a_f \pi_{L,0}}{\mathrm{RWC}_L^T - a_f}\frac{\frac{1}{1-a_{f,0}}\frac{\partial a_{f,0}}{\partial \pi_{L,0}} - \frac{1}{\varepsilon_{L,0}}}{1-\frac{\pi_{L,0}}{\varepsilon_{L,0}}}\right). \qquad \text{Eq. S4.7.8}$$

For the 1,000 best parameter sets (Fig. S5-S10), the sign of $\frac{\partial \dot{\pi}_L}{\partial A_n}$ is always negative according to Eq. S4.7.8. Hence, the sign of $\frac{\partial^2 \dot{\pi}_L}{\partial {g_w}^2}$ is always positive according to Eq. S4.7.6, meaning that the solution to our

optimization problem is the one that minimizes $\dot{\pi}_L$. Since osmotic potential is negative, minimizing $\dot{\pi}_L$ is the same as strategy as maximizing $|\dot{\pi}_L|$, and our solution represents a strategy that prioritizes maintaining large concentrations of leaf solutes. A stomatal strategy that maximizes the rate of concentrating osmolytes emphasized the role of osmolytes and osmotic potential for loading sugars into phloem to be used later by the rest of the plant for growth, survival, and reproduction. For active loaders, greater sugar concentrations enhances phloem loading (Borstlap & Schuurmans, 2004), while for passive loaders, a more negative osmotic potential draws more water into the symplast, increasing leaf turgor pressure and thereby enhancing phloem loading rates (Jensen, 2018).

For constrained turgor pressure, $\frac{\partial \dot{P}_L}{\partial A_n}$ is determined by Eq. S4.1.15, Eq. S4.5.5, and Eq. S4.5.7b,

$$\frac{\partial \dot{P}_L}{\partial A_n} = \frac{\partial P_L}{\partial \dot{n}_L^{S,A_n}} \frac{\partial \dot{n}_L^{S,A_n}}{\partial A_n} = \varepsilon_L \frac{a_L \varpi R T_L^K}{\alpha W_L^S} \frac{\frac{1}{1-a_{f,0}} \frac{1}{\mathrm{RWC}_L^S} \frac{\partial a_f}{\partial \pi_L} + \frac{a_f}{1-a_{f,0}} \frac{\frac{1}{1-a_{f,0}} \frac{\partial a_{f,0}}{\partial \pi_{L,0}} - \frac{1}{\varepsilon_{L,0}}}{1-\frac{\pi_{L,0}}{\varepsilon_{L,0}}}}{1-\frac{1}{1-a_{f,0}} \frac{\pi_L}{\mathrm{RWC}_L^S} \frac{\partial a_f}{\partial \pi_L}}. \quad \text{Eq. S4.7.9}$$

The sign of $\frac{\partial \dot{P}_L}{\partial A_n}$ may be simplified, ignoring the multiplicative positive terms, to

$$\operatorname{sgn}\left(\frac{\partial \dot{P}_L}{\partial A_n}\right) = \operatorname{sgn}\left(\frac{1}{\mathrm{RWC}_L^S} \frac{\partial a_f}{\partial \pi_L} + a_f \frac{\frac{1}{1-a_{f,0}} \frac{\partial a_{f,0}}{\partial \pi_{L,0}} - \frac{1}{\varepsilon_{L,0}}}{1-\frac{\pi_{L,0}}{\varepsilon_{L,0}}}\right). \quad \text{Eq. S4.7.10}$$

For the 1,000 best parameter sets (Fig. S5-S10), the sign of $\frac{\partial \dot{P}_L}{\partial A_n}$ is virtually always positive according to Eq. S4.7.10 (>99.9%). Recall that the best parameter sets were fitted using the version of our stomata model that used osmotic potential as the ***constraint***; and we expect that all of our estimates of $\frac{\partial \dot{P}_L}{\partial A_n}$ would be positive had we used parameter sets fitted using the alternative version of the stomata model that used turgor pressure as the ***constraint***. Hence, the sign of $\frac{\partial^2 \dot{P}_L}{\partial {g_w}^2}$ is negative according to Eq. S4.7.6, representing a stomatal strategy that prioritizes leaf turgor pressure. Such a strategy would maintain hydration and prevent desiccation of leaves.

For constrained total water potential, $\frac{\partial \dot{\psi}_L}{\partial A_n}$ is determined as the sum of Eq. S4.7.7 and Eq. S4.7.9,

$$\frac{\partial \dot{\psi}_L}{\partial A_n} = \frac{\partial \dot{\pi}_L}{\partial A_n} + \frac{\partial \dot{P}_L}{\partial A_n} = \frac{a_L \varpi R T_L^K}{\alpha W_L^S} \frac{\frac{1}{1-a_{f,0}} \frac{\varepsilon_L}{\mathrm{RWC}_L^S} \frac{\partial a_f}{\partial \pi_L} - 1 + a_f \left(\frac{\varepsilon_L}{1-a_{f,0}} - \frac{\pi_{L,0}}{\mathrm{RWC}_L^T - a_f}\right) \frac{\frac{1}{1-a_{f,0}} \frac{\partial a_{f,0}}{\partial \pi_{L,0}} - \frac{1}{\varepsilon_{L,0}}}{1-\frac{\pi_{L,0}}{\varepsilon_{L,0}}}}{1-\frac{1}{1-a_{f,0}} \frac{\pi_L}{\mathrm{RWC}_L^S} \frac{\partial a_f}{\partial \pi_L}} \quad \text{Eq. S4.7.11a}$$

$$\Rightarrow \frac{\partial \dot{\psi}_L}{\partial A_n} = \frac{a_L \varpi R T_L^K}{\alpha W_L^S} \frac{\frac{1}{1-a_{f,0}} \frac{\varepsilon_L}{\mathrm{RWC}_L^S} \frac{\partial a_f}{\partial \pi_L} - 1 + \frac{a_f}{1-a_{f,0}} \frac{\varepsilon_L - \pi_L}{\varepsilon_{L,0} - \pi_{L,0}} \left(\frac{\varepsilon_{L,0}}{1-a_{f,0}} \frac{\partial a_{f,0}}{\partial \pi_{L,0}} - 1\right)}{1-\frac{1}{1-a_{f,0}} \frac{\pi_L}{\mathrm{RWC}_L^S} \frac{\partial a_f}{\partial \pi_L}}. \quad \text{Eq. S4.7.11b}$$

The sign of $\frac{\partial \dot{\psi}_L}{\partial A_n}$ may be simplified, ignoring the multiplicative positive terms, to

$$\operatorname{sgn}\left(\frac{\partial \dot{\psi}_L}{\partial A_n}\right) = \operatorname{sgn}\left[\frac{1}{1-a_{f,0}} \frac{\varepsilon_L}{\mathrm{RWC}_L^S} \frac{\partial a_f}{\partial \pi_L} - 1 + \frac{a_f}{1-a_{f,0}} \frac{\varepsilon_L - \pi_L}{\varepsilon_{L,0} - \pi_{L,0}} \left(\frac{\varepsilon_{L,0}}{1-a_{f,0}} \frac{\partial a_{f,0}}{\partial \pi_{L,0}} - 1\right)\right]. \quad \text{Eq. S4.7.12}$$

For the 1,000 best parameter sets (Fig. S5-S10), the sign of $\frac{\partial \dot{\psi}_L}{\partial A_n}$ is typically positive according to Eq. S4.7.12 (>84%). Again, recall that the best parameter sets were fitted using the version of our stomata model that used osmotic potential as the ***constraint***; and we expect that all of our estimates of $\frac{\partial \dot{\psi}_L}{\partial A_n}$ would be positive

had we used parameter sets fitted using the alternative version of the stomata model that used the total water potential as the ***constraint***. Hence, we expect that the sign of $\frac{\partial^2 \dot{\psi}_L}{\partial {g_w}^2}$ is negative according to Eq. S4.7.6, representing a stomatal strategy that maximizes total leaf water potential. Since the leaf water potential is negative, this strategy seeks to maintain less-negative total water potentials. Like our solution with turgor pressure as the ***constraint***, the solution with total water potential as the ***constraint*** prioritizes leaf hydration and avoids desiccation.

S5. Overview of input data and filtering

In this section, we describe the sources of data used to validate our stomata optimality model. Represented species include Red maple (*Acer rubrum L.*) and Red oak (*Quercus rubra L.*) saplings from the B4Warmed experiment (Rich et al., 2015; Stefanski et al., 2023) in northern Minnesota, USA, Norway spruce (*Picea abies*) and European larch (*Larix decidua*) trees growing at five sites in the Swiss Alps (hereafter called the Lötschental sites), and Ponderosa pine (*Pinus ponderosa*) seedlings from a greenhouse experiment.

The five Lötschental sites are located in the Swiss Alps (46°23’040” N, 7°45’035” E) and are distributed along an elevational gradient (at c. 1300, 1600, 1900 and 2200 m asl) from the valley bottom to the tree line. For full description for the Lötschental sites and measurements, please see Peters et al. (2021). We evaluated Lötschental data from all five sites; however, after filtering data for quality (see below), only trees from the 1300 and 2200 m asl stands remained. Trees in the 1300 m asl stands included trees grown in both wet and dry stands. Midday leaf water potentials, ψ_L, were measured with a Scholander pressure chamber (Boyer, 1967). Stomata conductance, g_w, was estimated from sap flow measurement following Peters et al. (2019) by normalizing sap flow rates by leaf areas that were estimated from measured sapwood areas (i.e., Huber values). Micrometeorological conditions (T_a and RH) were monitored (002 Pro; Onset, MA, USA) and used to calculate VPD (WMO, 2008). Sap flow measurements were measured continuously for individual trees at each site, and hence we consider each tree to be a distinct treatment when fitting our model (Section S6). Since T_L was not measured at the Lötschental sites, T_L was approximated as T_a, and thus leaf-to-air VPD, D_L, was estimated as the VPD. The maximum carboxylation capacity, $V_{c,max}$, at 23°C was estimated from A-c_i curves measured in July 2022 using a LI-6800 portable system (LI-COR Inc., Lincoln, NE, USA) equipped with the Large Leaf and Needle Chamber (36 cm^2), with light set to saturation, and relative humidity to set ambient conditions. We used the rapid A-C_i approach (Coursolle et al., 2019), imposing CO_2 concentrations between 50 and 1010 ppm. The A-C_i data was processing according to Duursma (2015).

The B4Warmed experiment (Rich et al., 2015; Stefanski et al., 2023) was first established in 2008. Its sites are located in northern Minnesota, USA at the Cloquet Forestry Center (CFC; 46°40′46″N, 92°31′12″W, 382 m asl) near Cloquet, MN, and the Hubachek Wilderness Research Center (HWRC; 47°56′42″ N, 91°45′29″ W, 415 m asl) near Ely, MN in the ecotone of the boreal-temperate forest. We evaluated B4Warmed data from six combinations of two rainfall manipulations (ambient and reduced) and three temperature treatments (ambient, +1.7°C, and +3.4°C). The greenhouse drought experiment (Sapes & Sala, 2021) collected data for 1-year-old Ponderosa pine from two genetically differentiated populations known as the North Plateau race (NP) (42.6 N 122.8 W) and the Northern Rocky Mountain race (RM) (45.9 N 104.5 W), which we treated as separate treatments when fitting our model (Section S6). In both the B4Warmed and greenhouse experiments, midday leaf water potentials, ψ_L, were measured using a pressure chamber (PMS Instrument Company, Corvallis, OR) following methods by Kaufmann (1968), and gas exchange measurements (g_w and A_n) were performed at light saturation using a Li-6400XT infrared gas exchange analyzers (LICOR). In the B4Warmed experiment, temperature, relative humidity, and VPD inside the LICOR chamber were set to track the ambient conditions, while in the greenhouse experiment, temperature relative humidity, and light conditions, which were set at 25°C and 50%, 1000 $\mu mol \cdot m^{-2} \cdot s^{-1}$, respectively.

For both the B4Warmed and greenhouse experiments, we estimated the maximum carboxylation capacity, $V_{c,max}$, by the 'one-point method' (De Kauwe et a., 2016) from gas-exchange measurements. In these calculations, the dark respiration rate, R_d, at 25°C was assumed proportional to $V_{c,max}$ at 25°C with a $R_{d,25°C}$:$V_{c,max,25°C}$ ratio of 0.015 based on Collatz et al. (1991). Temperature dependencies of Farquhar et al. (1980) parameters (R_d, $V_{c,max}$, Γ^*, K_c, K_o) and values for Γ^*, K_c, K_o at 25°C were based on Bernacchi et al. (2001; their Table 1), and o_i was assumed to be 210 mmol·mol^{-1}.

Data was filtered for quality before calibrating the model. Data points were removed if they resulted in a ***non-steady-state** marginal carbon profit of water*, λ_{nss}, that was negative. We estimated λ_{nss} by Eq. S3.1.9 and assumed that photosynthesis was carboxylation limited, that boundary layer resistances were negligible, and thus that $k = V_{c,max}\frac{\Gamma^*+K_c\left(1+\frac{o_i}{K_o}\right)}{\left[c_i+K_c\left(1+\frac{o_i}{K_o}\right)\right]^2}$ according to Eq. S3.1.2. Additionally, we filtered out data points if the internal leaf CO_2 partial pressure, c_i, was less than the CO_2 compensation point, Γ^* (predicted by Bernacchi et al., 2001), and if the interpreted atmospheric CO_2 partial pressure, c_a ($c_a = c_i + \frac{A_n}{g_c}$), was smaller than c_i. For data with maximum carboxylation capacities at 25°C, $V_{c,max,25°C}$, that we interpreted by the 'one-point method' (i.e., for data from the B4Warmed and greenhouse experiments), data points were also removed if their corresponding $V_{c,max,25°C}$ was either negative or greater than an upper bound of realistic values of 400 μmol·m^{-2}·s^{-1}.

S6. Model calibration

We estimated six fitting parameters ($a_{f,max}$, $\varepsilon_{L,max}$, π_L^*, β, SWC_L, α) for each species using a Markov chain Monte Carlo (MCMC) method, particularly the DREAM_(KZS) algorithm (Zhang et al., 2020). The DREAM_(KZS) algorithm is a modified version of the DiffeRential Evolution Adaptive Metropolis (DREAM) algorithm (Vrugt, 2016) with enhanced efficiency for highly parameterized system models. In particular, DREAM_(KZS) incorporates a new proposal distribution suggested in Vrugt et al. (2013) that was inspired by the Kalman filter (Kalman, 1960).

S6.1. Differentiation between species-specific and treatment-specific parameters

The six fitting parameters ($a_{f,max}$, $\varepsilon_{L,max}$, π_L^*, β, SWC_L, α) were assumed to be constant among treatments (i.e., individual mature trees from Lötschental sites, the six combinations of two rainfall manipulations and three temperature treatments for saplings from the B4Warmed experiment, and the two populations in the greenhouse experiment) for a species, and we attributed the intraspecific variation in g_w among treatments to variation in the observed T_L, ψ_L, and photosynthetic parameters, as well as another parameter, $\pi_{L,0,25℃}$, which is the osmotic potential at full hydration at a standard temperature of 25°C. We allowed $\pi_{L,0,25℃}$ to vary among treatments while estimating our other fitting parameters. Fig. S1 shows the steps of our algorithm that determined the $\pi_{L,0,25℃}$ for each treatment. For a combination of the six other parameters, we varied $\pi_{L,0,25℃}$ between -3 and -1 MPa for each individual treatment, the range of which is based on a meta-analysis of its interspecies variation (Bartlett et al., 2012), and we chose the value of $\pi_{L,0,25℃}$ that resulted in the lowest sum square error for that treatment (Fig. S1), calculated from the error between the measured g_w and the predicted g_w (Section S6.2).

S6.2. Calculation of the predicted stomatal conductance

Midday g_w was predicted, assuming that midday photosynthesis is carboxylation-limited (i.e., f_0 and γ in Eq. S3.1.1 are $V_{c,max}$ and $K_c\left(1+\frac{o_i}{K_o}\right)$, respectively). First, we calculated χ_w by Eq. S4.4.2 using the

six species-specific fitting parameters ($a_{f,max}$, $\varepsilon_{L,max}$, π_L^*, β, SWC_L, α) and a single treatment-specific parameter ($\pi_{L,0,25℃}$) and the measured environmental conditions (T_L, D_L, c_a, P_{atm}). Applying Eq. S4.4.2 to estimate χ_w requires determining the leaf relative water contents (RWC_L^T and RWC_L^S), which we estimated from the measured ψ_L from the given leaf pressure-volume parameters ($a_{f,max}$, $\varepsilon_{L,max}$, π_L^*, β, $\pi_{L,0}$) through inversion of the definition of the total water potential ($\psi_L = P_L + \pi_L$) in combination with Eq. S4.1.1 and Eq. S4.1.17 (Section S4.1). Second, we assume that stomata behave optimally and thus that the *marginal profit* and *marginal cost* are equal ($\lambda_{nss} = \chi_w$). Third, we predict g_w from λ_{nss} through Eq. S3.3.10 and Eq. S3.3.11 (when assuming diffusion-fixation coupling or we predict g_w from λ'_{nss} through Eq. S3.2.7 when assuming diffusion-fixation decoupling; Section S3.2) using the estimated photosynthetic parameters (Section S5) and the measured environmental conditions. For the photosynthetic parameters (R_d, $V_{c,max}$, Γ^*, K_c, K_o), we used the same temperature-responses that were used in the 'one-point method' to estimate $V_{c,max}$ (Bernacchi et al., 2001; Section S5).

Predictions of g_w were replaced with an arbitrary value of 9999 mol·m^{-2}·s^{-1} to force large errors and thus to effectively discount the predictions from the MCMC when either of two conditions were met. The first condition is that the predicted g_w values contained imaginary numbers. The second condition is that the parameter combination ($a_{f,max}$, $\varepsilon_{L,max}$, π_L^*, β, SWC_L, α, $\pi_{L,0,25℃}$) would fail to predict complete or near-complete stomatal closure at the leaf wilting point. Stomatal closure at the wilting point was determined by comparing the predicted g_w at full hydration ($\mathrm{RWC}_L^T = 1$) to the predicted g_w at a bulk leaf relative water content at turgor loss of 0.85 ($\mathrm{RWC}_L^T = 0.85$), since the bulk leaf relative water content at turgor loss varies little among species and climatic conditions and is rarely less than 0.85 (Bartlett et al., 2012). Complete or near complete stomatal closure at the leaf wilting point was considered if the ratio of the g_w at turgor loss to the g_w at full hydration was less than or equal to 0.15. Both the g_w at full hydration and the g_w at turgor loss were calculated for the same reference environmental conditions ($T_L = 25℃$, $D_L = 1\ \mathrm{kPa}$, $c_a = 400\ \mu\mathrm{mol} \cdot \mathrm{mol}^{-1}$, $P_{atm} = 101.325\ \mathrm{kPa}$).

S6.3. Parameters and prior distributions

In our MCMC, the prior distributions were selected to reflect reasonable and physically realistic ranges of each parameter. $a_{f,max}$ was bound between 0 and 0.6. The maximum possible value of $a_{f,max}$ is 1 since it is a fraction. An upper bound of 0.6 was chosen for $a_{f,max}$ based on two lines of thought. First, traditional leaf pressure-volume analyses that assume constant apoplastic fraction estimate apparent apoplastic fractions that are typically less than 0.4 in a meta-analysis of its interspecies variation (Bartlett et al., 2012). Second, according to the few studies that either compare apoplastic fractions determined by traditional methods that assume constant apoplastic fraction to advanced methods that allow the apoplastic fraction to vary or present the data necessary to perform such comparison (Campbell et al., 1979; Andersen et al., 1991; Urban et al., 1993), the ratio of the maximum apoplastic fraction to apparent apoplastic fraction varies among the studied species, and its largest value reported in the literature is ~1.5 (Campbell et al., 1979; Urban et al., 1993). Hence, we estimated the upper bound for $a_{f,max}$ as 0.4 · 1.5 = 0.6. $\varepsilon_{L,max}$ was bound between 5 and 40 MPa, based on a meta-analysis of its interspecies variation (Bartlett et al., 2012), and SWC_L was bound between 1.5 and 3.5, based on reported values and measured values for the species considered here (Sapes & Sala, 2021; Belluau et al., 2021). Little is known about the range of π_L^* and β, considering the few studies that have investigated the a_f-π_L relationship (Campbell et al., 1979; Cortes & Sinclair, 1985; Andersen et al., 1991; Urban eta l., 1993). Hence, we bound π_L^* and β broadly between -20 MPa to -0.01 MPa and 0 and 6, respectively. α was bound between 0 and 3, where 3 is number of moles of carbon within a mole of triphosphate, which is the maximum possible value of α that occurs in the case that the chloroplast, rather than the cytoplasm, is the location of ***constraint*** (Section S4.1).

Prior distributions for the six fitting parameters ($a_{f,max}$, $\varepsilon_{L,max}$, π_L^*, β, SWC_L, α) were initially uniformly distributed; however, each combination of parameters was checked for complete or near-complete stomatal

closure at the leaf wilting point. Fig. S2 is a flowchart of our algorithm used to generate an initial parameter combination. If the parameter combination did not meet this criterion, then the parameter combination was replaced with another uniformly distributed parameter combination until the criterion was satisfied. Stomatal closure at the wilting point was determined by comparing the predicted g_w at full hydration ($\mathrm{RWC}_L^T = 1$) to the predicted g_w at a bulk leaf relative water content at turgor loss of 0.85 ($\mathrm{RWC}_L^T = 0.85$), since the bulk leaf relative water content at turgor loss varies little among species and climatic conditions and is rarely less than 0.85 (Bartlett et al., 2012). Complete or near complete stomatal closure at the leaf wilting point was considered if the ratio of the g_w at turgor loss to the g_w at full hydration was less than or equal to 0.15. Both the g_w at full hydration and the g_w at turgor loss were calculated for the same reference environmental conditions ($T_L = 25℃$, $D_L = 1\ \mathrm{kPa}$, $c_a = 400\ \mathrm{\mu mol \cdot mol^{-1}}$, $P_{atm} = 101.325\ \mathrm{kPa}$). Whether this criterion is satisfied also depends on the value of $\pi_{L,0,25℃}$, which was not specified during parameter initialization but rather later in our algorithm (Section S6.1). Since $\pi_{L,0,25℃}$ was not yet determined during parameter initialization, for a given combination of six parameters, we determined whether the criterion was satisfied for a range of $\pi_{L,0,25℃}$ values between -3 and -1 MPa. This range was chosen to be a realistic range in the parameter based on a meta-analysis (Bartlett et al., 2012). If the criterion was satisfied for any the values of $\pi_{L,0,25℃}$, then the parameter combination was accepted.

Other parameters necessary to run the DREAM_(KZS) algorithm (Vrugt et al., 2016; Zhang et al., 2020) are shown in Table S6.1. When not measured, the standard deviation of measurement errors was assumed to be proportional to the observed g_w by a factor of 0.35 (Venturas et al., 2018).

Table S6.1. DREAM_(KZS)-specific parameters

DREAM_(KZS)-specific parameters	Value
Number of parallel chains in MCMC (*N*)	20
Number of iterations (*T*)	1e4
Iteration after which the Kalman proposal is applied (t_1)	100
Iteration after which the Kalman proposal is no longer applied (t_2)	3e3

S6.4. Comparison to predictions from the USO model

We compare our model's ability to capture observations of stomatal conductance to that of the well-established and widely used Unified Stomata Optimization (USO) model (Medlyn et al., 2011) (Fig. S12 & Fig. S13). The USO model is an approximate solution to the classic optimization problem of Cowan and Farquhar (1977). The USO model predicts the stomata conductance to water vapor, in the absence of a minimum conductance, as

$$g_w = 1.6\left(1 + \frac{g_1}{\sqrt{D_L}}\right)\frac{A_n}{c_a}, \quad \text{Eq. S6.4.1}$$

where g_1 is an empirical constant [$\mathrm{kPa}^{0.5}$] that is negatively related to the *marginal carbon cost of water* (Medlyn et al., 2011). Theoretically, g_1 should be constant (Cowan & Farquhar, 1977); however, empirically, g_1 varies with environmental conditions, including leaf temperature and leaf water potentials (Gimeno et al., 2016; Aparecido et al., 2020; Davidson et al., 2023; Marchin et al., 2023). Hence, we fit the USO model twice.

In the first calibration, we fit a constant g_1 to each treatment by finding the treatment-specific g_1 that minimized the root-mean square error (RMSE) between observed and predicted stomatal conductances. We used MATLAB's fminsearch function for this RMSE-minimizing calibration instead of the DREAM_(KZS) algorithm that was used to calibrate our model (Section S6.3) because the USO model has only one parameter and thus does not require a calibration method as intensive as DREAM_(KZS). Nonetheless, by minimizing RMSE, this calibration is consistent with DREAM_(KZS) because DREAM_(KZS) maximizes

likelihood, which is inversely proportional to RMSE under certain conditions (Hodson, 2022). Specifically, predictions of stomatal conductance were made from Eq. S6.4.2e below. Eq. S6.4.2e is derived by solving for the internal leaf CO_2 partial pressure (c_i) that allowed for carbon fixation (Eq. S3.1.1) and CO_2 diffusion (Eq. S3.1.2) to be equal, thereby providing an equation for A_n, which is plugged back into Eq. S6.4.1 for g_w,

$$g_c(c_a - c_i) - \left(f_0 \frac{c_i - \Gamma^*}{c_i + \gamma} - R_d\right) = 0 \quad \text{Eq. S6.4.2a}$$

$$\Rightarrow \left(1 + \frac{g_1}{\sqrt{D_L}}\right)\left(f_0 \frac{c_i - \Gamma^*}{c_i + \gamma} - R_d\right)\left(1 - \frac{c_i}{c_a}\right) - \left(f_0 \frac{c_i - \Gamma^*}{c_i + \gamma} - R_d\right) = 0 \quad \text{Eq. S6.4.2b}$$

$$\Rightarrow \left(1 + \frac{g_1}{\sqrt{D_L}}\right)\left(1 - \frac{c_i}{c_a}\right) - 1 = 0 \quad \text{Eq. S6.4.2c}$$

$$\Rightarrow c_i = \frac{g_1 c_a}{g_1 + \sqrt{D_L}} \quad \text{Eq. S6.4.2d}$$

$$\Rightarrow g_w = \frac{1.6}{c_a}\left(1 + \frac{g_1}{\sqrt{D_L}}\right)\left(f_0 \frac{\frac{g_1 c_a}{g_1 + \sqrt{D_L}} - \Gamma^*}{\frac{g_1 c_a}{g_1 + \sqrt{D_L}} + \gamma} - R_d\right), \quad \text{Eq. S6.4.2e}$$

where f_0 and γ are $V_{c,max}$ and $K_c\left(1 + \frac{o_i}{K_o}\right)$, respectively, using the measured $V_{c,max}$ for data from the Lötschental sites and the $V_{c,max}$ estimated by the 'one point' method for data from the B4Warmed and greenhouse experiments (Section S5).

In the second calibration, we attempted to describe the environmental control of variations in g_1 through species-specific linear mixed effect models with fixed effects from the atmospheric CO_2 partial pressure, leaf temperature, and leaf water potential and treatment as a random effect. For this second calibration, we first made point estimates for g_1 by inversion of Eq. S6.4.1,

$$g_1 = \sqrt{D_L}\left(\frac{g_c c_a}{A_n} - 1\right). \quad \text{Eq. S6.4.3}$$

Next, we describe the structure of the linear mixed-effects modeling applied in the second calibration of the USO model accounting for the environmental effects on g_1 (Fig. S13). The *marginal carbon cost of water* is expected to vary with atmospheric CO_2 partial pressures (Manzoni et al.,2011; Nakad et al., 2023), and since g_1 is related to the *marginal cost*, g_1 may also vary with atmospheric CO_2 partial pressures. We assume that the *marginal cost* depends on the multiplicative product of absolute leaf temperature (in Kelvin; T_L^K), atmospheric CO_2 partial pressures, and an exponential function of leaf water potential (Manzoni et al., 2011; Zhou et al., 2013),

$$\chi_w \propto c_a {T_L^K}^{\sigma} \exp(-\beta\psi_L), \quad \text{Eq. S6.4.4}$$

where σ [-] and β [MPa^{-1}] are the sensitivities of the *marginal cost* to leaf temperature and leaf water potential, respectively. Since $g_1 \propto {\chi_w}^{-\frac{1}{2}}$ (Medlyn et al., 2011), we expect that g_1 follows the form,

$$g_1 \propto {c_a}^{-\frac{1}{2}} {T_L^K}^{-\frac{\sigma}{2}} \exp\left(\frac{\beta}{2}\psi_L\right). \quad \text{Eq. S6.4.5}$$

By taking a log-transformation of Eq. S6.4.5, the empirical environmental controls of g_1 may be determined by linear mixed-effects modeling,

$$\log(g_1) = \log(g_1^*) + \gamma_{c_a}\log(c_a) + \gamma_{T_L}\log(T_L^K) + \gamma_{\psi_L}\psi_L, \quad \text{Eq. S6.4.6}$$

where g_1^* is an intercept term [$kPa^{0.5}$], and γ_{c_a}, γ_{T_L}, and γ_{ψ_L}, are the fixed effect slopes of $\log(c_a)$, $\log(T_L^K)$, and leaf water potential, respectively, on g_1. Theoretically, according to Eq. S6.4.5, γ_{c_a}, γ_{T_L}, and γ_{ψ_L} should

equal $-\frac{1}{2}$, $-\frac{\sigma}{2}$, and $\frac{\beta}{2}$, respectively; however, we allow the linear mixed-effects modeling to determine their values.

For each species, we regressed the logarithmically transformed g_1 point estimates through linear mixed-effects modeling with atmospheric CO_2 partial pressure (log transformed), absolute leaf temperature (log transformed), and midday leaf water potentials as fixed effects and experimental treatment as a categorical random effect on the intercept term (i.e., $\log(g_1) \sim 1 + \log(c_a) + \log(T_L^K) + \psi_L + (1|\text{Treatment})$; Eq. S6.4.6) using MATLAB's fitlme function. This choice for the structure of the linear mixed-effects models was chosen to be consistent with how we parameterized our model (Section S6.1), in which model parameters were assumed constant among treatments, except for the osmotic potential at full hydration, which we varied among treatments. The osmotic potential at full hydration affects the maximum χ_w in our model (Eq. S4.4.2d), and so allowing the osmotic potential at full hydration to vary among treatments is consistent with allowing the intercept term in the linear mixed-effects models to vary among treatments. Predictions of g_1 from the species-specific linear mixed-effects models were used to predict stomatal conductance (Eq. S6.4.2) using their established values (Section S5).

We compare our model's fit to that of the widely used Universal Stomata Optimization (USO) model (Medlyn et al., 2011) that we calibrated with a constant value of its sole parameter (g_1) per treatment in manner consistent with how we calibrated our model (Fig. S12). In terms of r^2, the USO performed worse than our model for Ponderosa pine (r^2 = 0.41) from the greenhouse experiment, better for European larch (r^2 = 0.68) and Norway spruce (r^2 = 0.53) from the Lötschental sites, and comparably well for Red maple (r^2 = 0.43) and Red oak (r^2 = 0.21) from the B4Warmed field experiment. In terms of RMSE, the USO model performed comparably well for Ponderosa pine (RMSE = 0.047 $mol{\cdot}m^{-2}{\cdot}s^{-1}$), European Larch (RMSE = 0.058 $mol{\cdot}m^{-2}{\cdot}s^{-1}$), Norway spruce (RMSE = 0.016 $mol{\cdot}m^{-2}{\cdot}s^{-1}$), Red maple (RMSE = 0.036 $mol{\cdot}m^{-2}{\cdot}s^{-1}$), and Red oak (RMSE = 0.061 $mol{\cdot}m^{-2}{\cdot}s^{-1}$). In a secondary analysis with the USO model, we attempted to capture environmental variations in the USO model's g_1 parameter, which is related to our model's *marginal cost* term (χ_w in Eq. 2) (Medlyn et al., 2011), through mixed effects linear modeling (Fig. S13). For Ponderosa pine (r^2 = 0.54; RMSE = 0.046 $mol{\cdot}m^{-2}{\cdot}s^{-1}$), Red maple ($r^2$ = 0.26; RMSE = 0.052 $mol{\cdot}m^{-2}{\cdot}s^{-1}$), and Red oak ($r^2$ = 0.32; RMSE = 0.069 $mol{\cdot}m^{-2}{\cdot}s^{-1}$), this version of the USO model performed comparably to our model and performed better than our model for European larch (r^2 = 0.74; RMSE = 0.055 $mol{\cdot}m^{-2}{\cdot}s^{-1}$), and Norway spruce ($r^2$ = 0.69; RMSE = 0.013 $mol{\cdot}m^{-2}{\cdot}s^{-1}$). For all species except Red maple, the second calibration that accounted for environmental variations in g_1 performed better than the first USO model that did not in terms of r^2; however, the RMSE of the two USO models were similar for most species (all except Red maple and Red oak, for which the first calibration performed better), suggesting that variations in g_1 (and thus χ_w) were often only weakly correlated with environmental conditions in our datasets. The coefficients for the fixed effects from the linear mixed effects used in the second round of fitting the USO model's g_1 parameter are shown in Table S6.2.

The effect of CO_2 partial pressures was significant for only the Ponderosa Pine seedlings, for which species the effect of CO_2 was more much negative than expected (i.e., theoretical value of –½; Eq. S6.4.4), and for which species it was the only significant fixed effect. The large size of the negative effect of CO_2 on the g_1 of Ponderosa pine cannot be explained as a spurious correlation between c_a and g_1 due to g_1 being calculated from c_a (Eq. S6.4.1) because, if the correlation were spurious, the effect would be positive, not negative, since the effect of c_a on g_1 in Eq. S6.4.1 is positive ($\partial g_1/\partial c_a > 0$).

In the remaining species, the effect of leaf temperature was always large and significant, although the effect was negative at the Lötschental site (European Larch, Norway Spruce) and positive at the B4Warmed site (Red Maple, Red Oak). The difference in sign for the effect of leaf temperature on g_1 between the Lötschental site and the B4Warmed site can be explained through how leaf temperatures were estimated at those sites. Leaf temperatures and VPD were measured at the B4Warmed site, while leaf temperatures were approximated by air temperatures, and VPD were thus calculated from solely air temperatures at the Lötschental site (Section S5). Since g_1 is calculated from VPD (Eq. S6.4.1), a spurious correlation between leaf temperature and g_1 could exist at the Lötschental site, and it would be a negative,

like observed at the Lötschental site (Table S6.2), since the effect of leaf temperature on g_1 through D_L by Eq. S6.4.1 is negative ($\partial g_1/\partial T_L = \partial g_1/\partial D_L \cdot \partial D_L/\partial T_L \equiv \partial g_1/\partial D_L \cdot \partial D_L/\partial T_a < 0$, where $T_L = T_a$ at Lötschental, $\partial D_L/\partial T_a > 0$, and $\partial g_1/\partial D_L < 0$ because Eq. S6.4.2 is equivalent to $g_1 = \sqrt{D_L}\left(\frac{g_w c_a}{1.6 A_n} - 1\right) \approx \sqrt{D_L}\left(\frac{E c_a}{1.6 D_L A_n} - 1\right) = \frac{E c_a}{1.6\sqrt{D_L} A_n} - \sqrt{D_L}$; note that g_c is calculated from measurements of E and D_L). Hence, the correlation between leaf temperature and g_1 at the Lötschental site is likely spurious. The large positive effect of leaf temperature on g_1 at the B4Warmed site cannot be explained by leaf temperatures and leaf water potentials being correlated there despite that the greatest amount of hydraulic stress in the B4Warmed experiment was produced by simultaneous heating and drought. Warmer leaf temperatures were only weakly correlated with more negative leaf water potentials ($r^2 \leq 0.120$) and were not statistically significant (except for European Larch and Norway Spruce from the Lötschental site; however, their leaf temperatures were not measured and assumed equal to air temperature).

The effect of leaf water potential was significant for European Larch, Norway Spruce, and Red Oak; however, the effect of leaf water potential was negative for all species, meaning that the correlation suggests that more negative leaf water potentials caused stomata to open, contrary to past studies (Manzoni et al., 2011; Zhou et al., 2013) and common knowledge. We interpret these unexpected results as suggesting measurement errors in our data sources, temporal mismatches between measurements of midday leaf water potentials and leaf gas exchange, limited number of infrequent leaf water potential measurements during experiments and field campaigns, our estimation of stomatal conductance values from sap flux measurements and our estimation of leaf temperature as the air temperature at the Lötschental site, and correlations between leaf water potentials and leaf and air temperatures.

Table S6.2. Coefficients for full linear mixed effects model for g_1 parameter.

	Ponderosa Pine	European Larch[1,2]	Norway Spruce[1,2]	Red Maple	Red Oak
γ_{c_a} (lower, upper)	**-18.03 (-30.70, -5.37)**	-	-	6.25 (-5.01, 17.51)	-4.56 (-12.55, 3.43)
p-value for γ_{c_a}	**$6.1 \cdot 10^{-3}$**	-	-	$2.7 \cdot 10^{-1}$	$0.26 \cdot 10^{-1}$
γ_{T_L} (lower, upper)	6.74 (-25.67, 39.15)	**-15.69 (-21.44, -9.93)**	**-8.05 (-13.66, -2.45)**	**18.35 (8.01, 28.69)**	**17.07 (7.77, 26.38)**
p-value for γ_{T_L}	$6.8 \cdot 10^{-1}$	**$3.4 \cdot 10^{-7}$**	**$5.9 \cdot 10^{-3}$**	**$7.8 \cdot 10^{-4}$**	**$5.1 \cdot 10^{-4}$**
γ_{ψ_L} (lower, upper)	-0.17 (-0.43, 0.08)	**-0.35 (-0.68, -0.01)**	**-0.52 (-0.84, -0.20)**	-0.07 (-0.28, 0.14)	**-0.23 (-0.43, -0.03)**
p-value for γ_{ψ_L}	$1.7 \cdot 10^{-1}$	**$4.5 \cdot 10^{-2}$**	**$1.8 \cdot 10^{-3}$**	$5.0 \cdot 10^{-1}$	**$2.3 \cdot 10^{-2}$**

[1]Linear mixed effect models were created without the fixed effects of atmospheric CO_2 partial pressure for European Larch and Norway Spruce because atmospheric CO_2 partial pressures were not measured at the Lötschental site.

[2]Relationship between leaf temperature and g_1 may be spurious due to leaf temperatures being estimated as air temperatures, creating a confounding effect on g_1 through VPD (see text in Section S6.4).

In an attempt to avoid potentially spurious relationships, we performed a third USO model calibration by regressing each species' g_1 point estimates through mixed effects models using leaf water potential as the only fixed effect, excluding leaf temperature and CO_2 concentration, and experimental treatment as a categorical random effect on the intercept term (i.e., $\log(g_1) \sim 1 + \psi_L + (1|\text{Treatment})$; Table S6.3). In comparison to the previous full mixed effect model (Fig. S13), this simplified mixed effect model led to more poorly predicted predicting stomatal conductance both in terms of reduced r^2 for all species (Ponderosa pine: $r^2 = 0.41$; European larch: $r^2 = 0.67$; Norway spruce: $r^2 = 0.63$; Red Oak; $r^2 = 0.29$) except Red maple ($r^2 = 0.38$) and in terms of slightly increased RMSE for all species (Ponderosa pine: RMSE = 0.048 $mol \cdot m^{-2} \cdot s^{-1}$; European larch: RMSE = 0.067 $mol \cdot m^{-2} \cdot s^{-1}$; Norway spruce: RMSE = 0.015 $mol \cdot m^{-2} \cdot s^{-1}$; Red maple; RMSE = 0.055 $mol \cdot m^{-2} \cdot s^{-1}$) except Red oak (RMSE = 0.067 $mol \cdot m^{-2} \cdot s^{-1}$). The effect of leaf water

potential was significant for only Norway spruce and Red oak, and the effect of leaf water potential was negative for all species (Table S6.3), meaning that more negative leaf water potentials caused stomata to open (at least for Norway spruce and Red oak) like the full mixed effects model and unlike expectations.

Table S6.3. Coefficients for simplified linear mixed effects model for g_1 parameter.

	Ponderosa Pine	European Larch	Norway Spruce	Red Maple	Red Oak
γ_{ψ_L} (lower, upper)	-0.04 (-0.16, 0.08)	-0.11 (-0.29, 0.07)	**-0.54 (-0.71, -0.37)**	-0.07 (-0.19, 0.05)	**-0.34 (-0.44, -0.23)**
p-value for γ_{ψ_L}	$7.3 \cdot 10^{-1}$	$5.4 \cdot 10^{-1}$	**$2.7 \cdot 10^{-3}$**	$5.4 \cdot 10^{-1}$	**$1.9 \cdot 10^{-3}$**

S7. Additional analyses

In this section, we detail how we predicted the responses of stomatal conductance to leaf water potential, leaf temperature, and the osmotic potential at full hydration at a standard temperature of 25°C for Ponderosa pine seedlings (Fig. 2; Section S7.1) as well as the responses of stomatal conductance to leaf-to-air vapor pressure deficit (VPD) from leaf temperatures and leaf relative water contents that are either constant or calculated from the heat and water flux balance equations in ***steady-state*** for Ponderosa pine with a minimalist whole-plant plant hydraulics and leaf heat balance model (Fig. 3; Section S7.2). Our minimalist model predicts *supercritical* water potentials under certain environmental conditions, in which the leaf water potential is more negative than the critical water potential at which transpiration is maximal. We discuss these *supercritical* water potentials and explain how we estimated the critical leaf water potential for the Ponderosa Pine seedlings (Section S7.3). Additionally, we describe our application of the same minimalist whole-plant plant hydraulics and leaf heat balance model to alternative stomatal models (Section S7.4) and to ecosystem-scale predictions for Norway Spruce-dominated forests (Section S7.5).

While the value of the maximum carboxylation capacity, $V_{c,max}$, was interpreted from measurements in our other analyses (Section S5), we require predicting how $V_{c,max}$ varies with environmental conditions for these analyses (Fig. 2-4). Here, we predicted the maximum carboxylation capacity at 25°C, $V_{c,max,25°C}$, assuming that it varies with leaf water potential, ψ_L, according to the sigmoidal function proposed by Tuzet et al. (2003),

$$V_{c,max,25℃} = V_{c,max,25℃,0} \frac{1+\exp(S_f \psi_f)}{1+\exp[S_f(\psi_f - \psi_L)]} \quad \text{Eq. S7.1}$$

where $V_{c,max,25°C,0}$ is the maximum carboxylation capacity at 25°C at full hydration (ψ_L = 0 MPa) [mol·m^{-2}·s^{-1}], and S_f [MPa^{-1}] and ψ_f [MPa] are shaping parameters. We fit Eq. S7.1 to the values of $V_{c,max,25°C}$ estimated for Ponderosa pine by iterative least squares estimation (MATLAB's nlinfit function). The parameters were found to be $V_{c,max,25°C,0}$ = 5.80 μmol·m^{-2}·s^{-1}, S_f = 2.98 MPa^{-1}, and ψ_f = -1.98 MPa for the Ponderosa pine seedlings. Like in Section S5, leaf dark respiration at 25°C was assumed proportional to $V_{c,max}$ at 25°C with a $R_{d,25°C}$:$V_{c,max,25°C}$ ratio of 0.015 based on Collatz et al. (1991). Values of other photosynthetic parameters (Γ^*, K_c, K_o) as well as the $V_{c,max}$:$V_{c,max,25°C}$ and R_d:$R_{d,25°C}$ ratios were predicted from leaf temperature according to Bernacchi et al. (2001; their Table 1) as previously described in Section S5 unless noted elsewhere.

S7.1. Stomatal response to leaf water potential, leaf temperature, and osmotic potential at full hydration

For Fig. 2, observations of stomatal conductance for Ponderosa pine were first binned into groups of low VPD (1 ≤ D_L ≤ 2 kPa) and high VPD (2 ≤ D_L ≤ 3 kPa). Each bin's observed stomatal conductance

values were plotted against their corresponding observed leaf water potentials (Fig. 2a,b) and leaf temperatures (Fig. 2c,d). Predictions of stomatal conductance were made at constant VPD with D_L = 1.5 kPa for comparison to observations in the low VPD bin and with D_L = 2.5 kPa for comparison to observations in the high VPD bin. Predictions of stomatal conductance were made with varied leaf water potential and constant leaf temperature and osmotic potential at full hydration at a standard temperature of 25°C, $\pi_{L,0,25°C}$ (Fig. 2a,b), varied leaf temperature and constant leaf water potential and a $\pi_{L,0,25°C}$ (Fig. 2c,d), and varied $\pi_{L,0,25°C}$ and constant leaf water potential and leaf temperature. (Fig. 2e,f). These predictions were made with the best parameters from the MCMC with the lowest sum square error for Ponderosa pine (Fig. S5). Unless specified elsewhere, $\pi_{L,0,25°C}$ was set to its baseline value taken as the mean of the best $\pi_{L,0,25°C}$ values averaged among treatments (Fig. S10). When a variable was varied (e.g., ψ_L in Fig. 2a,b, T_L in Fig. 2c,d), the range of variation corresponded to that variable's observed variation within the VPD bin, except for $\pi_{L,0,25°C}$, which was varied between 0.8-1.2 times its baseline value, in which the range is based the variable's natural range observed between pre- and post-drought conditions (Bartlett et al., 2014). When a variable was taken to be constant (e.g., T_L in Fig. 2a,b, ψ_L in Fig. 2c,d,e,f), values were taken as either the 10th and 90th quantiles (Fig. 2a,b) or the 10th, 50th, and 90th quantiles (Fig. 2c,d,e,f) of the observed values within the bin to compare to range of the bin-specific data. Predictions were made by first calculating χ_w from Eq. S4.4.2 using the chosen values of leaf water potential, leaf temperature, and $\pi_{L,0,25°C}$. Then we set $\lambda_{nss} = \chi_w$ and solved for g_w from λ_{nss} through Eq. S3.3.10 and Eq. S3.3.11 using the value of $V_{c,max}$ that was adjusted for temperature from the $V_{c,max,25°C,0}$ solved by Eq. S7.1 from the leaf water potential.

S7.2. Stomatal response to VPD by varying either air temperature or relative humidity with a minimalist whole-plant plant hydraulics and leaf heat balance model

Additional theoretical responses of g_w to VPD were predicted for the Ponderosa pine seedlings from the greenhouse experiment (Fig. 3). We made predictions with constant leaf temperature (21, 24, and 27°C) and full leaf hydration ($\mathrm{RWC}_L^T = 1$) as well as with calculated leaf temperatures and relative water contents that we estimated from a minimalist model with ***steady-state*** leaf heat balance and ***steady-state*** plant hydraulics. In these predictions, parameters were chosen from the MCMC for Ponderosa pine as the mode of the posterior distribution (Fig. S5), and $\pi_{L,0,25°C}$ was set to its baseline value taken as the mean of the best $\pi_{L,0,25°C}$ values averaged among treatments (Fig. S10). Stomatal conductance was predicted as already described in Sections S6.2 and S7.1 (i.e., Eq. S4.4.2, Eq. S3.3.10-S3.3.11). When we estimated leaf temperature from environmental conditions, we either varied air temperature and kept relative humidity constant (RH = 0.5) or varied RH and kept air temperature constant (T_a = 24°C) to investigate their differing effects on the model's prediction. In these calculations, we set the absorbed shortwave radiation, R_{abs}, to 300 W·m^{-2}, the boundary layer conductance, $g_{H,a}$, to 3 mol·m^{-2}·s^{-1}, the leaf emissivity, ϵ_L, to 0.97, the atmospheric pressure, P_{atm}, to 101.325 kPa, the atmospheric CO_2 partial pressure, c_a, to 400 µmol·mol^{-1}, and the soil water potential, ψ_{soil}, to 0 MPa.

Since we vary air temperature to values far higher than experienced by the Ponderosa pine seedlings the in the greenhouse experiment (50 vs. 32°C), we modify our calculation of the temperature response of $V_{c,max}$ to include a realistic negative response at high temperatures. This negative response is captured by a multiplying our temperature response used elsewhere (Bernacchi et al., 2001) by $\frac{1+\exp\left(\frac{\Delta S T_{25°C}^K - H_d}{R T_{25°C}^K}\right)}{1+\exp\left(\frac{\Delta S T_L^K - H_d}{R T_L^K}\right)}$ to recover the temperature response equation of Kattge and Knorr (2007), where ΔS is the entropy term [J·mol^{-1}·K^{-1}], H_d is the deactivation energy [J·mol^{-1}], R is the universal gas constant [J·mol^{-1}·K^{-1}], and $T_{25°C}^K$ is the standard temperature of 25°C in units of Kelvin [K]. We assume reasonable values for these parameters and set ΔS to 640 J·mol^{-1}·K^{-1} and H_d to 2·10^5 J·mol^{-1} (Kattge & Knorr, 2007), which results in a temperature optimum of 36.4°C. Since the temperature optimum exceeds the warmest observed leaf temperatures (32°C), this modification has a negligible effect on our $V_{c,max}$ predictions below this

temperature optimum compared to our previous temperature response predictions without negative response at high temperatures.

When we estimated leaf temperature from environmental conditions, we determined it according to the leaf heat balance equation (Eq. S4.1.18) in ***steady-state*** ($\dot{T}_L^K = 0$). In ***steady-state***, the leaf thermal capacitance, C_L, does not affect the predicted leaf temperature, T_L^K, and the heat balance equation is equivalent to

$$0 = R_{abs} - R_l - H - L. \qquad \text{Eq. S7.2.1}$$

The emitted net longwave radiation was calculated as

$$R_l = 2\sigma\left(\epsilon_L {T_L^K}^4 - {T_a^K}^4\right), \quad \text{Eq. S7.2.2}$$

where σ is the Stefan–Boltzmann constant [$W \cdot m^{-2} \cdot K^{-4}$] ($\sigma = 5.67 \cdot 10^{-8}$ $W \cdot m^{-2} \cdot K^{-4}$), ϵ_L is the leaf emissivity [-], and T_L^K and T_a^K are the leaf and air temperatures, respectively in Kelvin [K]. The sensible heat loss, H, was calculated as

$$H = 2c_p g_{H,a}(T_L^K - T_a^K), \quad \text{Eq. S7.2.3}$$

where c_p is the specific heat capacity of air [$J \cdot mol^{-1} \cdot K^{-1}$] ($c_p$ = 29.3 $J \cdot mol^{-1} \cdot K^{-1}$), and $g_{H,a}$ is the boundary layer conductance to heat [$mol \cdot m^{-2} \cdot s^{-1}$]. In Eq. S7.2.2 and Eq. S7.2.3, the factor of 2 represents the two sides of a leaf (Schymanski et al., 2013). The latent heat flux, L, was calculated from the transpiration, E, according to Eq. S4.1.19, where E was calculated by Eq. S3.1.4 given by g_w.

Simultaneously, we solved for the leaf relative water content in ***steady-state***, in which transpiration may be defined analogously to Darcy's law,

$$E = k_L(\psi_{soil} - \psi_L), \qquad \text{Eq. S7.2.4}$$

where k_L is the leaf area-specific soil-plant conductance [$mol \cdot m^{-2} \cdot s^{-1} \cdot MPa^{-1}$], ψ_{soil} is the soil water potential [MPa], and ψ_L is the aforementioned leaf water potential [MPa]. We estimated k_L for the Ponderosa pine seedlings and found that it followed an exponential relationship with ψ_L,

$$k_L = k_{L,max,25°C}\exp(S_k\psi_L)\,Q_{10}^{\frac{T_a^K - T_{25°C}^K}{10}}, \qquad \text{Eq. S7.2.5}$$

where $k_{L,max,25°C}$ is the maximum leaf area-specific soil-plant conductance at a standard temperature of 25°C [$mol \cdot m^{-2} \cdot s^{-1} \cdot MPa^{-1}$], S_k is a shape parameter for the relationship of k_L with ψ_L [MPa^{-1}], and Q_{10} describes the relationship between k_L and air temperature. Other formulations for fitting k_L, including those incorporating soil water potential (e.g., Kirchoff transformations of various sigmoidally shaped vulnerability curve functions; Sperry et al., 1998), were attempted but they did not improve the prediction of k_L. We assume that Q_{10} = 1.5, which was chosen as an intermediate value between the Q_{10} for stem conductance (1.25; Cochard et al., 2000) and leaf conductance (1.6; Matzner & Comstock, 2001; Sack et al., 2004). We fit Eq. S7.2.5 to the values of k_L estimated for Ponderosa pine log-transformed linear regression. The parameters were found to be $k_{L,max,25°C} = 7.22 \cdot 10^{-3}$ $mol \cdot m^{-2} \cdot s^{-1} \cdot MPa^{-1}$ and S_k = 1.40 MPa^{-1} (Fig. S3; Section S7.3 for more detail).

Hence, this ***steady-state*** analysis requires simultaneously solving for five unknowns (g_w, E, T_L, ψ_L, RWC_L^T) from five sets of equations. These sets of equations are (1) the pressure-volume equations for relating ψ_L to RWC_L^T (Eq. S4.1.1, Eq. S4.1.16-17, the Van't Hoff rule, the definition of total water potential), (2) the optimality-based equations for relating T_L and RWC_L^T to g_w (Eq. S4.4.2, Eq. S3.3.10-11) through $V_{c,max}$ (Eq. S7.1), (3) the diffusion equation for relating g_w to E (Eq. S3.1.4), (4) the ***steady-state*** heat balance equations for relating E to T_L^K (Eq. S4.1.19, Eq. S7.2.1-3), and (5) the ***steady-state*** water balance equation for relating E to ψ_L (Eq. S7.2.4-5).

S7.3 *Supercritical* leaf water potentials

Our minimalist whole-plant plant hydraulics and leaf heat balance model predicts *supercritical* leaf water potentials for certain combinations of soil water potential and leaf temperature (Fig. S21c & Fig. S22). By *supercritical*, we refer to leaf water potentials being more negative than the critical leaf water potential ($\psi_{L,crit}$), which is the leaf water potential at which transpiration is maximal (Fig. S22e; Sperry et al., 1998). At extreme air temperatures, our minimalist model predicts the transpiration demand exceeds its supply (Fig. S22g), which would lead to complete desiccation and which we refer to as an *unstable supercritical* regime. Under moderate temperatures and moderate water-stress, however, our model predicts that the supply satisfies the demand at leaf water potentials that are more negative that the critical leaf water potential (Fig. S22f), which we refer to as a *stable supercritical* regime.

It is novel that our optimality model can predict *supercritical* leaf water potentials, since many other stomatal optimality models can predict only *subcritical* leaf water potentials (i.e., leaf water potentials that are less negative that the critical water potential). Other models predict only *subcritical* water potentials for a several reasons. The first reason is that they define their ***objectives*** in terms of plant hydraulic conductance or similar terms (Sperry et al., 2017; Wang et al., 2020; Eller et al., 2020), which highly disincentivizes *supercritical* water potentials, which coincide with low hydraulic conductances. The second reason is that many models do not distinguish between water supply and demand. Many models deal only with the supply curve (i.e., a hypothetical relationship between E and ψ_L; Fig. S22e-g), which is determined by Darcy's law (Eq. S7.2.4 & Eq. S7.2.5; Sperry et al., 2017; Wang et al., 2020). Hence, they effectively treat the entire demand curve as identical to the supply curve. Conversely, our minimalist model determines supply and demand separately. We determine supply from Darcy's Law (Eq. S7.2.4 & Eq. S7.2.5) and determine demand from the diffusion of vapor through stomata (Eq. S3.1.4) with stomatal conductance predicted by our optimality model (Eq. S3.3.10 & Eq. S3.3.11) and with VPD predicted by the *steady-state* leaf heat balance (Eq. S7.2.1, Eq. S7.2.2, & Eq. S7.2.3) (Fig. S22e-g). Our ***steady-state*** solution lies where the two curves intersect (Fig. S22e-f). The third reason is that their numerical solvers consider only *subcritical* leaf water potentials when searching for the solution to their optimization problems (Sperry et al., 2017; Wang et al., 2020). That is, even if their ***objective*** mathematically allowed for *supercritical* water potentials, their numerical algorithms would ignore the possibility.

We tested whether *supercritical* leaf water potentials can be detected in observations using data from the greenhouse experiment for the Ponderosa Pine seedlings like our minimalist model suggests. We indeed found *supercritical* leaf water potentials measurements (Fig. S22d). The critical leaf water potential ($\psi_{L,crit}$) is solved by differentiating transpiration (E; Eq. S7.2.4 & Eq. S7.2.5) with respect to leaf water potential (ψ_L), setting the derivative to zero, and rearranging for the critical water potential,

$$\psi_{L,crit} = \psi_{soil} - \frac{1}{S_k}, \qquad \text{Eq. S7.3.1}$$

where S_k is the parameter that controls the decline in hydraulic conductance under water-stress [MPa^{-1}] (Eq. S7.2.5). Hence, we estimated the critical water potential by first estimating S_k and then applying Eq. S7.3.1 with the measured soil water potentials from the greenhouse experiment and the estimated value of S_k. To estimate S_k, we first made point estimates of the leaf area-specific soil-plant conductance (k_L) by inverting Eq. S7.2.4. Then, through multiple linear regression, S_k can be determined as the slope between the logarithmically-transformed k_L and ψ_L,

$$\ln(k_L) = \ln\left(k_{L,max,25℃}\right) + S_k\psi_L + \frac{T_a^K - T_{25℃}^K}{10}\ln(Q_{10}). \quad \text{Eq. S7.3.2}$$

Eq. S7.3.2 is a rearrangement of the log-transformation of Eq. S7.2.5. Comparison of the predicted ln(k_L) by multiple linear regression and observed values for the Ponderosa Pine seedlings is shown in Fig. S3.

S7.4 Alternative stomata models with a minimalist whole-plant plant hydraulics and leaf heat balance model

For comparison, we apply the same minimalist whole-plant plant hydraulics and leaf heat balance model (Section S7.2) with alternate formulations for the optimal stomatal conductance. Alternate stomatal conductance formulations included the constant *marginal carbon cost of water* (χ_w) formulation from the classic Cowan and Farquhar (1977) model and the Gain-Risk model of Sperry et al. (2017). When applying the Cowan and Farquhar (1977) model, we set the *marginal cost* to a constant value equal to the average of its value that we determined from our optimality model (Eq. S4.4.2 & Eq. S4.4.3) from the previous simulations with full leaf hydration. We fed this constant *marginal cost* into Eq. S3.3.10 & Eq. S3.3.11 to predict stomatal conductance.

For Sperry et al.'s (2017) Gain-Risk model, we numerically found the stomatal conductance that ***instantaneously*** maximized their objective,

$$\max_{g_w}\left(A_n + A_{max}\frac{k_c}{k_{c,max}}\right), \quad \text{Eq. S7.4.1}$$

where A_n is the net carbon assimilation rate [mol·m^{-2}·s^{-1}] (Eq. S3.1.1 & Eq. S3.1.3), A_{max} is the maximum value of A_n to occur in ***steady-state*** for leaf water potentials that are less negative than the critical water potential, k_c is the so-called canopy conductance [mol·m^{-2}·s^{-1}·MPa^{-1}],

$$k_c = -\frac{dE}{d\psi_L}, \qquad \text{Eq. S7.4.2}$$

and $k_{c,max}$ is the maximum value of k_c, occurring at $\psi_L = \psi_{soil}$. Due to how we modeled hydraulic conductance (Eq. S7.2.5), the canopy conductance is

$$k_c = k_{L,max,25°C}\exp(S_k\psi_L)\,Q_{10}^{\frac{T_a^K - T_{25°C}^K}{10}}[S_k(\psi_L - \psi_{soil}) + 1], \quad \text{Eq. S7.4.3}$$

and the maximum canopy conductance is

$$k_{c,max} = k_{L,max,25°C}\exp(S_k\psi_{soil})\,Q_{10}^{\frac{T_a^K - T_{25°C}^K}{10}}. \qquad \text{Eq. S7.4.4}$$

Hence, the $\frac{k_c}{k_{c,max}}$ term in Eq. S7.4.1 is independent of the $k_{L,max,25°C}$ term and of temperature,

$$\frac{k_c}{k_{c,max}} = \exp[S_k(\psi_L - \psi_{soil})]\,[S_k(\psi_L - \psi_{soil}) + 1]. \quad \text{Eq. S7.4.5}$$

S7.5 Ecosystem-scale predictions with a minimalist whole-plant plant hydraulics and leaf heat balance model

We applied the same minimalist whole-plant plant hydraulics and leaf heat balance model described in Section S7.2 to make ecosystem-scale predictions for Norway Spruce during the 2018 European drought (Fig. 4 & Fig. S23). Summer 2018 was exceptionally hot and dry in northwestern Europe (Buras et al., 2020; Peters et al., 2020), during which time ecosystem level carbon fluxes were often reduced (Smith et al., 2020), and widespread forest mortality was observed (Schuld et al., 2020). We make predictions during the non-drought and drought years of 2017 and 2018, respectively, for the Oberbärenburg (DE-Obe; Bernhofer et al., 2022) eddy covariance site in eastern Germany. We simulated diurnal changes using a time-step of ½ hour (1800 s). The Oberbärenburg site was chosen for its similar climate (i.e., mean annual precipitation, mean annual temperature) as the Lötschental site in Switzerland, for which we parameterized our stomatal optimality model for Norway Spruce (Section S5). Like the Lötschental site, the Oberbärenburg site is also needle-leaf dominated, particularly spruce dominated. We had originally considered the Davos (CH-Dav; Buchmann et al., 2022) eddy covariance site in Switzerland

for our ecosystem-scale simulations because the Davos site is more similar to Lötschental site geographically and climatically; however, the Davos site reported unrealistic values and temporal trends for environmental conditions, gross primary productivity (GPP), and latent heat between 2017-2018. We use environmental conditions measured between 2017-2018 at the Oberbärenburg site (precipitation, temperature, relative humidity, atmospheric pressure, shortwave radiation, friction velocity, CO_2 concentration) to force our model. We use additional flux tower measurements (GPP, latent heat) from the non-drought year of 2017 at the Oberbärenburg site and Terra-MODIS/Aqua-MODIS/VIIRS-derived leaf area index (LAI) (Pu et al., 2023a,b) to select reasonable model parameters. Model parameters were selected to result in reasonable predictions of canopy-averaged leaf area-specific net carbon assimilation ($\tilde{A}_n$) and ground area-specific total evapotranspiration ($\widetilde{\mathrm{ET}}$; sum of plant transpiration and soil evaporation), although we did not perform an extensive calibration to match eddy covariance-based estimates and our model predictions. Eddy covariance-based estimates of $\tilde{A}_n$ [mol·m^{-2}·s^{-1}] and $\widetilde{\mathrm{ET}}$ [mol·m^{-2}·s^{-1}] were determined as

$$\tilde{A}_n = \frac{\mathrm{GPP}}{\mathrm{LAI}} \qquad \text{Eq. S7.5.1}$$

$$\widetilde{\mathrm{ET}} = \frac{L}{m_w \Lambda_E}, \qquad \text{Eq. S7.5.2}$$

where GPP is the gross primary productivity data product from the Oberbärenburg site [mol·m^{-2}·s^{-1}], LAI is the Terra-MODIS/Aqua-MODIS/VIIRS-derived leaf area index [m^2·m^{-2}], L is the latent heat energy data product from the Oberbärenburg site [W·m^{-2}], $m_w = 18 \times 10^{-3}\ \frac{\mathrm{kg}}{\mathrm{mol}}$ is the molar mass of water [kg·mol^{-1}], and Λ_E is the latent heat of vaporization [J·kg^{-1}] ($\Lambda_E = \Lambda_{E,0°\mathrm{C}} + S_{\Lambda_E}\tilde{T}_A$, where $\Lambda_{E,0°\mathrm{C}} = 2.5 \times 10^6\ \mathrm{J \cdot kg^{-1}}$ and $S_{\Lambda_E} = -2365\ \mathrm{J \cdot kg^{-1} \cdot °C^{-1}}$; as described in Section S4.1) Additionally, we calculated the Standardized Precipitation Evapotranspiration Index (SPEI) according to Vicente-Serrano et al. (2010) to identify drought periods in 2018. In this section, we describe modifications to our minimalist model (Section S7.2) that we made to apply it for Norway Spruce forests at an ecosystem scale.

Throughout this section, we use tildes over symbols to denote measurements, eddy covariance-based estimates, or variables that depend exclusively on measurements (e.g., $\tilde{e}'_a$ in Eq. S7.5.5 to denote the saturation vapor pressure evaluated at the measured air temperature).

S7.5.1 Soil water and soil evaporation

In our previous analyses, soil water potentials (ψ_{soil}) were either unnecessary or their value values were set (e.g., Section S7.2). For ecosystem-scale predictions, we predict changes in the volumetric soil water content (θ) and soil water potential (ψ_{soil}) using a simple soil water bucket model,

$$\dot{\theta} = \frac{1}{Z_R}\left(\tilde{P} - \frac{m_w}{\rho_w}\mathrm{ET}\right), \qquad \text{Eq. S7.5.3}$$

where Z_R is the effective rooting depth [m], P is the precipitation data product from the Oberbärenburg site [m·s^{-1}], $\rho_w = 10^3\ \frac{\mathrm{kg}}{\mathrm{m^3}}$ is density of water [kg·m^3], and ET is the predicted ground area-specific total evapotranspiration [mol·m^{-2}·s^{-1}]. Z_R was set to 0.8 m based on the median rooting depth for Norway Spruce from Fan et al. (2017). ET is the sum of the LAI-corrected predicted leaf area-specific plant transpiration (E; [mol·m^{-2}·s^{-1}]; predicted by minimalist model; Section S7.2; using the best PV parameters for Norway Spruce and the mean of the different treatment's best osmotic potential at full hydration; Section S6) and the predicted ground area-specific soil evaporation (E_{soil}) [mol·m^{-2}·s^{-1}],

$$\mathrm{ET} = \widetilde{\mathrm{LAI}} \cdot E + E_{soil} \qquad \text{Eq. S7.5.4}$$

$$E_{soil} = g_{soil}\frac{\tilde{e}'_a}{\tilde{P}_{atm}}\left(\mathrm{RH}_{soil} - \widetilde{\mathrm{RH}}\right), \quad \text{Eq. S7.5.5}$$

where P_{atm} is the atmospheric pressure [kPa], and g_{soil} is the ground area-specific conductance [mol·m^{-2}·s^{-1}], e'_a is the saturation vapor pressure [kPa] evaluated at the air temperature (T_a), and RH$_{soil}$ and RH are the relative humidities of the soil and the air, respectively. Eq. S7.5.5 assumes that the soil temperature may be approximated by the air temperature. We selected a constant value for g_{soil} that predicted reasonable total evapotranspiration (ET) during the non-drought year of 2017 (Section S7.5.4). RH$_{soil}$ is calculated according to Philip and de Vries' equation (1957),

$$\mathrm{RH}_{soil} = \exp\left(\frac{m_w \psi_{soil}}{\rho_w R \tilde{T}_a^K}\right), \quad \text{Eq. S7.5.6}$$

where ψ_{soil} is the soil water potential [MPa], R is the universal gas constant [J·K^{-1}·mol^{-1}], and T_a^K is the absolute air temperature (in Kelvin; [K]). Soil water potentials were calculated from volumetric soil water contents (θ) through the soil water retention curve of Van Genuchten (1980),

$$\psi_{soil} = -\frac{1}{\alpha_{VG}}\left[\left(\frac{\theta-\theta_{min}}{\theta_{max}-\theta_{min}}\right)^{\frac{n_{VG}}{1-n_{VG}}} - 1\right]^{\frac{1}{n_{VG}}}, \quad \text{Eq. S7.5.7}$$

where θ_{min} and θ_{max} are respectively the minimum and maximum volumetric soil water contents [m^3·m^{-3}], and α_{VG} [MPa^{-1}] and n_{VG} [-] are shape parameters. These soil parameters (θ_{min}, θ_{max}, α_{VG}, n_{VG}) were estimated by pedotransfer functions developed for German forest soils (Teepe et al., 2003) from sand, silt, and clay content and soil bulk density. We used values of 40%, 45%, 15%, and 1.4 g·cm^{-3} for sand, silt, and clay content and soil bulk density, respectively, which were taken from SoilGrids (https://soilgrids.org/) for the pixels closest to the Oberbärenburg site (50.78666°N, 13.72129° W). These soil physical properties lead to predicted values for θ_{min}, θ_{max}, α_{VG}, n_{VG} of 0 m^3·m^{-3}, 0.465 m^3·m^{-3}, 80.9 MPa^{-1}, and 1.1874, respectively. In addition to predicting soil evaporation (Eq. S7.5.5 & Eq. S7.5.6), Eq. S7.5.7 was used to predict changes in soil water potential from changes in volumetric soil water content (Eq. S7.5.3). Eq. S7.5.3 was solved by simple explicit finite difference using a time-step of ½ hour (1800 s).

S7.5.2 Electron transport-limitation, absorbed radiation, and boundary heat conductance

In our previous analyses, we modeled midday photosynthesis, which we assumed to be carboxylation-limited and did not consider electron transport-limitations due to low irradiance. Since we simulate diurnal changes in our ecosystem predictions, we consider the possibility of electron transport-limitations due to low irradiance here. Net carbon assimilation (A_n) was set as the minimum of carboxylation-limited and electron-transport limited rates ($A_{n,c}$ & $A_{n,j}$, respectively),

$$A_n = \min(A_{n,c}, \quad A_{n,j}), \quad \text{Eq. S7.5.8}$$

where $A_{n,c}$ and $A_{n,j}$ are both Michaelis-Menten functions (Eq. S3.1.1) of the leaf internal CO_2 partial pressure (c_i) with different Michaelis-Menten coefficients. For $A_{n,c}$, f_0 is $V_{c,max}$, calculated using the peaked temperature-response curve described previously (Kattge & Knorr, 2007; Section S7.2), and γ is $K_c\left(1+\frac{o_i}{K_o}\right)$, using the values and temperature-dependences described previously for K_c, K_o, and o_i (Bernacchi et al., 2001; Section S5). For these ecosystem predictions of Norway Spruce, we did not model the downregulation of $V_{c,max}$ under water-stress (Eq. S7.1), because the $V_{c,max}$ was not measured frequently enough at the Lötschental site to regress $V_{c,max,25°C}$ as a function of leaf water potentials. We selected a constant value for $V_{c,max,25°C}$ that predicted reasonable peak growing season net caron assimilation (A_n) during the non-drought year of 2017 (Section S7.5.4). For $A_{n,j}$, f_0 is J/4, where J is the electron transport rate [mol·m^{-2}·s^{-1}], and γ is $2\Gamma^*$, using the values and temperature-dependences described previously for Γ^* (Bernacchi et al., 2001; Section S5). We model leaf respiration in the same manner as described previously (Section S5). In practice, we solved g_w twice by Eq. S3.3.10 and Eq. S3.3.11 using the two different sets of values of f_0 and γ, and we used the lesser of the two g_w predictions, since it coincides with the lesser of the two A_n predictions (Eq. S7.5.8).

The electron transport rate (J) is modeled as the minimum of a maximum electron transport rate (J_{max}) and the φ·PAR, where PAR is the photon flux density [mol·m^{-2}·s^{-1}], and φ is the quantum yield (φ = 0.3 mol·mol^{-1}; Medlyn et al., 2002).

$$J = \min(\varphi \cdot \mathrm{PAR},\quad J_{max}). \qquad \text{Eq. S7.5.9}$$

J_{max} was estimated from its temperature-corrected value at 25°C ($J_{max,25°C}$), which was assumed to be proportional to the temperature-corrected value of $V_{c,max}$ at 25°C ($V_{c,max,25°C}$) by a factor of 1.67 (Medlyn et al., 2002), and the temperature response reported by Bernacchi et al. (2003). PAR was estimated from the absorbed radiation (R_{abs}) with a proportionality factor of 2.1·10^{-6} mol·m^{-2}·s^{-1}·W^{-1}·m^{2} (Potkay et al., 2021). The absorbed radiation was modeled following Beer's law from LAI and the incident radiation above the canopy (R_0; [W·m^{-2}]) measured at the Oberbärenburg site,

$$R_{abs} = \tilde{R}_0 \frac{1-\exp(-k_{\mathrm{LAI}} \cdot \widetilde{\mathrm{LAI}})}{\widetilde{\mathrm{LAI}}}, \qquad \text{Eq. S7.5.10}$$

where k_{LAI} = 0.5 is an extinction coefficient [m^{2}·m^{-2}]. In addition to predicting PAR and thus J (Eq. S7.5.9), this value of R_{abs} (Eq. S7.5.10) was used in the leaf energy balance to predict leaf temperatures (Eq. S7.2.1). When calculating the leaf energy balance, the boundary layer conductance to heat term ($g_{H,a}$; [mol·m^{-2}·s^{-1}]) in Eq. S7.2.3 was estimated from the measured friction velocity (u^*; [m·s^{-1}]) at the Oberbärenburg site,

$$g_{H,a} = \frac{1000 \cdot \tilde{P}_{atm}}{R \tilde{T}_a^K} C_v \sqrt{\frac{\tilde{u}^*}{d_L}}, \qquad \text{Eq. S7.5.11}$$

where C_v = 0.01 m·s$^{-½}$ is a turbulent transfer coefficient [m·s$^{-½}$], d_L is the characteristic leaf dimension [m], and the $\frac{1000 \cdot \tilde{P}_{atm}}{R \tilde{T}_a^K}$ term converts from units of m·s^{-1} to mol·m^{-2}·s^{-1} (Barber et al., 2004). We set d_L = 10^{-3} m based on Niinements & Kull (1995) for Norway Spruce needles.

S7.5.3 LAI-corrected hydraulic conductance

Leaves are major contributors to the hydraulic resistance of the soil-to-plant hydraulic pathway (Wolfe et al., 2023), and thus changes in leaf area influence plants' total hydraulic resistance. We modified the leaf area-specific soil-plant conductance term (k_L) used in Eq. S7.2.4 to account for LAI seasonality. As leaf area increases, its contribution to resistance also rises, and k_L should decline. Hence, in place of Eq. S7.2.5, we modeled k_L as

$$k_L = \frac{k_{L,max,25°C,ref} \exp(S_k \psi_L) Q_{10}^{\frac{\tilde{T}_a^K - T_{25°C}^K}{10}}}{f_{L,ref} + (1-f_{L,ref}) \frac{\widetilde{\mathrm{LAI}}}{\mathrm{LAI}_{ref}}}, \qquad \text{Eq. S7.5.12}$$

where $k_{L,max,25°C,ref}$ is the maximum leaf area-specific soil-plant conductance at a standard temperature of 25°C and at a reference LAI [mol·m^{-2}·s^{-1}·MPa^{-1}], LAI_{ref} is the reference LAI (m^{2}·m^{-2}), S_k is a shape parameter for the relationship of k_L with ψ_L [MPa^{-1}], Q_{10} describes the relationship between k_L and air temperature, and $f_{L,ref}$ is the fraction of soil-to-leaf hydraulic resistance in leaves at the reference LAI. We assume that Q_{10} = 1.5 as previously described (Section S7.2). We also assume that $f_{L,ref}$ = 0.3 at LAI_{ref} = 2.5 m^{2}·m^{-2}, where 2.5 m^{2}·m^{-2} was chosen as the approximate minimum LAI observed between May and September in 2017, and 0.3 was chosen for $f_{L,ref}$ based on reported trends between tree height and the fraction of resistance in leaves of trees from temperate forests (Wolfe et al., 2023), assuming maximum Norway Spruce heights (35–55 m). Values of $k_{L,max,25°C,ref}$ and S_k were chosen to predict realistic leaf water potentials and percent loss conductance during the non-drought year of 2017 (Section S7.5.4).

S7.5.4 Calibration

We calibrated 4 parameters (g_{soil}, $V_{c,max,25°C}$, $k_{L,max,25°C,ref}$, S_k) for our ecosystem-scale prediction of the Norway Spruce-dominated forests at the Oberbärenburg site. We did not rigorously calibrate their values (in comparison to Section S6). Instead, we simply chose values that resulted in reasonable predictions, particularly in comparison to the eddy covariance-based estimates of net carbon assimilation ($\tilde{A}_n$; Eq. S7.5.1) and evapotranspiration ($\widetilde{\mathrm{ET}}$; Eq. S7.5.2) for the non-drought year of 2017. The parameters were successively calibrated in the following order: (1) $V_{c,max,25°C}$, (2) $k_{L,max,25°C,ref}$ and S_k simultaneously, and (3) g_{soil}. $V_{c,max,25°C}$ was calibrated first because the year of 2017 was relatively wet, and hence the model's predictions of A_n were most sensitive to $V_{c,max,25°C}$ and relatively insensitive to the remaining hydraulic and hydrologic parameters ($k_{L,max,25°C,ref}$, S_k, g_{soil}), especially g_{soil}, which is the reason why we calibrated it last. Simulations during the drought year of 2018 were sensitive to g_{soil}; however, the parameter had a weak effect on the non-drought year of 2017 for which we calibrated our model.

First, $V_{c,max,25°C}$ and thus also $J_{max,25°C}$, since $J_{max,25°C}$ is assumed proportional to $V_{c,max,25°C}$ (Section S7.5.2), were adjusted until the predicted midday net carbon assimilation (A_n) at the peak of the growing season in 2017 reasonably well matched the eddy covariance-based estimate ($\tilde{A}_n$) at the same time (Eq. S7.5.1). While adjusting $V_{c,max,25°C}$, we set the whole plant-soil conductance ($k_{L,max,25°C,ref}$ in Eq. S7.5.12) arbitrarily large with no percent loss conductance (S_k = 0 MPa^{-1}), and we forced zero soil evaporation (g_{soil} = 0 $mol \cdot m^{-2} \cdot s^{-1}$), so that simulated leaf water potentials were close to zero and thus also that predictions of A_n were controlled most by the parameterization of $V_{c,max,25°C}$. We adjusted the value of $V_{c,max,25°C}$ until we were satisfied with $V_{c,max,25°C}$ = 73.31 $\mu mol \cdot m^{-2} \cdot s^{-1}$, which is double the mean value measured at the Lötschental site for Norway Spruce.

Second, we adjusted the whole plant-soil conductance ($k_{L,max,25°C,ref}$ in Eq. S7.5.12) until the model predicted realistic leaf water potentials using the value of $V_{c,max,25°C}$ determined in the previous step and forcing zero soil evaporation (g_{soil} = 0 $mol \cdot m^{-2} \cdot s^{-1}$). While adjusting $k_{L,max,25°C,ref}$, we forced the model to prevent loss of conductance due to hydraulic stress (S_k = 0 MPa^{-1}). Hence, we refer to this value of $k_{L,max,25°C,ref}$ as the *in-situ* $k_{L,max,25°C,ref}$ ($k_{L,max,25°C,ref,in\text{-}situ}$), which approximates the mean of the product of $k_{L,max,25°C,ref}\,\exp(S_k \psi_{L,min})$ in Eq. S7.5.12, where $\psi_{L,min}$ is the most negative leaf water potential [MPa]. We adjusted $k_{L,max,25°C,ref,in\text{-}situ}$ until the predicted midday leaf water potentials at the peak of the growing season in 2017 approximately equaled the mean observed value at the Lötschental site ($\psi_{L,min} \approx$ -1.4 MPa), which was nearly satisfied when $k_{L,max,25°C,ref,in\text{-}situ} \approx 10^3$ $\mu mol \cdot m^{-2} \cdot s^{-1} \cdot MPa^{-1}$. We then reintroduced non-zero, positive values S_k, while maintaining the value of $k_{L,max,25°C,ref,in\text{-}situ}$. That is, we simultaneously adjusted S_k and $k_{L,max,25°C,ref}$ while ensuring that their values produced the same $k_{L,max,25°C,ref,in\text{-}situ}$ (i.e., $k_{L,max,25°C,ref} = k_{L,max,25°C,ref,in-situ}\,\exp(-S_k \psi_{L,min})$), stopping when the model predicted a realistic maximum percent loss conductance (PLC, $\mathrm{PLC} = 1 - \exp(-S_k \psi_{L,min})$) for temperate conifers of ~25% (Manzoni et al., 2013b). We were reasonably satisfied with our final values of $k_{L,max,25°C,ref}$ = $1.3 \cdot 10^3$ $\mu mol \cdot m^{-2} \cdot s^{-1} \cdot MPa^{-1}$ and S_k = 0.2 MPa^{-1}.

Third, for simplicity, we estimated a constant value of g_{soil} from simulations of the 2017 non-drought year using the values of $V_{c,max,25°C}$, $k_{L,max,25°C,ref}$, and S_k determined in the previous two steps and forcing zero soil evaporation (g_{soil} = 0 $mol \cdot m^{-2} \cdot s^{-1}$). The predicted plant transpiration (E) from this simulation and the eddy covariance-based total evapotranspiration ($\widetilde{\mathrm{ET}}$) were used to estimate g_{soil} as its temporally averaged value by inversion of Eq. S7.5.4 and Eq. S7.5.5,

$$g_{soil} = \frac{\int_{\text{January 1st,2017}}^{\text{December 31,2017}} \frac{\tilde{P}_{atm}}{\tilde{e}'_a} \frac{\widetilde{\mathrm{ET}} - \widetilde{\mathrm{LAI}} \cdot E}{(\mathrm{RH}_{soil} - \widetilde{\mathrm{RH}})}\, dt}{365 \text{ days}}, \qquad \text{Eq. S7.5.13}$$

giving g_{soil} = 0.172 $mol \cdot m^{-2} \cdot s^{-1}$. We recognize that there is an inconsistency in using a simulation without soil evaporation to estimate g_{soil}. Since the original simulation lacks soil evaporation, the predicted soil moisture would be wetter than had we performed the simulation with soil evaporation. Fortunately, the 2017 non-

drought year had a surplus of precipitation, allowing similar soil moisture predictions regardless of the presence of simulated soil evaporation and value of g_{soil}.

When applying the Sperry et al. (2017) Gain-Risk model (Section S7.4) for the Norway Spruce-dominated ecosystem predictions at the Oberbärenburg site, we used these same parameters, even though they were calibrated for our stomatal model. We use the same parameters for the two different optimality models to focus on differences in their predictions that emerge solely from differences in their optimization problems (e.g., their time scales of optimization, their ***objectives***).

S8: Guide for future implementation

We encourage others to test our model against other data sets. Ideally, our stomata optimality model would be tested against coinciding measurements of stomatal conductance, g_w, relative water content, RWC_L^T and RWC_L^S, leaf temperature, T_L, since the *marginal carbon cost of water*, χ_w, is defined in terms of the latter two (Eq. S4.4.2). Other relevant terms like the saturated water content, SWC_L, the osmotic potential at full hydration, $\pi_{L,0}$, apoplastic fraction, a_f, and the elastic modulus, ε_L, can be determined through pressure-volume experiments (Tyree & Hammel, 1972). For such pressure-volume analyses, the data should be interpreted by methods that identify how the apoplastic fraction, a_f, varies with hydration status (Campbell et al., 1979; Andersen et al., 1991; Urban et al., 1993) for two reasons. The first reason is that the traditional methods that assume constant apoplastic fraction (Tyree & Hammel, 1972) can underestimate (Campbell et al., 1979; Andersen et al., 1991) or overestimate (Urban et al., 1993) the magnitude of $\pi_{L,0}$, which is a highly sensitivity parameter for our model (Fig. 2e,f). The second reason is that our theory is premised on observations that the apoplastic fraction varies. Either leaf mass could be measured immediately after measuring g_w to later interpret relative water content or leaf water potential and g_w could be measured at the same time. In the latter approach, relative water contents may be interpreted from the measured leaf water potentials through pressure-volume parameters. In either approach, pressure-volume analyses should be performed to directly estimate several of the parameters. Alternatively, any to all of the parameters may be estimated by model inversion as we have done here (Section S6). One parameter, α, can be determined only by fitting the model predictions to observations.

For prediction of the optimal stomatal conductance, the *marginal carbon cost of water*, χ_w, should first be predicted according to our Eq. S4.4.2. The osmotic potential term in Eq. S4.4.2, π_L, may be calculated from the osmotic potential at full hydration, $\pi_{L,0}$, from the Van't Hoff rule ($\pi_L = \frac{\pi_{L,0}}{\mathrm{RWC}_L^S}$). The a_f terms in Eq. S4.4.2 should be estimated in a manner that accounts for the variation of a_f with hydration status (e.g., Eq. S4.1.2). That is, a_f should not be treated as a constant. The apoplastic fraction, a_f, is expected to vary sigmoidally with osmotic status (Eq. S4.1.2; Andersen et al., 1991; Urban et al., 1993) over large ranges in the osmotic potential (Campbell et al., 1979. However, osmotic potential is known to change little between pre- and post-drought (Bartlett et al., 2014), and we found that it varies little between treatments of differing hydraulic stress (Fig. S10). Hence, osmotic potential likely does not vary enough during drought to observe the full nonlinear behavior of a_f. Instead, a_f may be approximated as a linear function of ψ_L (Fig. S11) or symplastic relative water content, RWC_L^S (Campbell et al., 1979), over the range of conditions typically experiences by plants. Treating a_f as a linear function of either ψ_L or RWC_L^S would be a satisfying simplification that would reduce the number of parameters to describe a_f. For example, based on Fig. 7, a_f could be approximated with only two parameters ($a_{f,0}$, S_ψ) as $a_f \approx a_{f,0}\left(1 + S_\psi \psi_L\right)$, where $a_{f,0}$ is the apoplastic fraction at full hydration [$m^3 \cdot m^{-3}$], and S_ψ is a slope parameter [MPa^{-1}], in comparison to our Eq. S4.1.2, which has three parameters to describe a_f ($a_{f,max}$, β, π_L^*). However, approximating a_f as a linear function of ψ_L or RWC_L^S does not change the total number of parameters in the model, since the Δ term in Eq. S4.4.2 would no longer be able to be solved from Eq. S4.4.3 and would have to be treated as a parameterized fitting constant. Yet another option is to approximate a_f as a two parameter ($a_{f,max}$, S_π) linear function of osmotic potential, $a_f \approx a_{f,max}(1 + S_\pi \pi_L)$, where S_π is a slope parameter [MPa^{-1}]. According to

this formulation, $a_{f,0} \approx a_{f,max}(1 + S_\pi \pi_{L,0})$, and thus Δ term in Eq. S4.4.2 may be calculated as $\Delta = \frac{\varepsilon_{L,0}}{\varepsilon_{L,0}-\pi_{L,0}}\left(\frac{\pi_{L,0} a_{f,max} S_\pi}{1-a_{f,0}} - 1\right)$ by Eq. S4.4.3 instead of being treated as a separate parameter. This last option would thereby reduce the total number of parameters in the model by one.

Once χ_w has been calculated by Eq. S4.4.2, the next step is to assume that stomata behave optimally and thus that the *marginal cost* and *marginal profit* are equal ($\lambda = \chi_w$). It remains unclear if our ***non-steady-state*** perspective extends to changes in internal leaf CO_2 concentrations, c_i (Section S3.2), and so both versions of the model with and without coupling between diffusion of CO_2 through stomata and the biological fixation of carbon should be tested. Two predictions of stomatal conductance should be made and tested. The first prediction is made by setting the ***non-steady-state*** *marginal profit* with diffusion-fixation coupling equal to the *marginal cost* ($\lambda_{nss} = \chi_w$) and predicting g_w from Eq. S3.3.10-1, assuming that c_i is in ***steady-state***. The second prediction is made by setting the ***non-steady-state*** *marginal profit* with diffusion-fixation decoupling equal to the *marginal cost* ($\lambda'_{nss} = \chi_w$) and predicting g_w from Eq. S3.2.5-7, assuming that c_i is ***non-steady-state***.

SI References

Andersen, M. N., Jensen, C. R., & Losch, R. (1991). Derivation of pressure-volume curves by a non-linear regression procedure and determination of apoplastic water. *Journal of Experimental Botany, 42*(2), 159-165.

Aparecido, L. M., Woo, S., Suazo, C., Hultine, K. R., & Blonder, B. (2020). High water use in desert plants exposed to extreme heat. *Ecology Letters, 23*(8), 1189-1200.

Barber, J. L., Thomas, G. O., Kerstiens, G., & Jones, K. C. (2004). Current issues and uncertainties in the measurement and modelling of air–vegetation exchange and within-plant processing of POPs. *Environmental pollution, 128*(1-2), 99-138.

Bartlett, M. K., Detto, M., & Pacala, S. W. (2019). Predicting shifts in the functional composition of tropical forests under increased drought and CO 2 from trade-offs among plant hydraulic traits. *Ecology Letters, 22*(1), 67-77.

Bartlett, M. K., Scoffoni, C., & Sack, L. (2012). The determinants of leaf turgor loss point and prediction of drought tolerance of species and biomes: a global meta-analysis. *Ecology Letters, 15*(5), 393-405.

Bartlett, M. K., Zhang, Y., Kreidler, N., Sun, S., Ardy, R., Cao, K., & Sack, L. (2014). Global analysis of plasticity in turgor loss point, a key drought tolerance trait. *Ecology Letters, 17*(12), 1580-1590.

Belluau, M., Paquette, A., Gravel, D., Reich, P. B., Stefanski, A., & Messier, C. (2021). Exotics are more complementary over time in tree biodiversity–ecosystem functioning experiments. *Functional Ecology, 35*(11), 2550-2561.

Bernacchi, C. J., Pimentel, C., & Long, S. P. (2003). In vivo temperature response functions of parameters required to model RuBP-limited photosynthesis. *Plant, Cell & Environment, 26*(9), 1419-1430.

Bernacchi, C. J., Singsaas, E. L., Pimentel, C., Portis, A. R., & Long, S. P. (2001). Improved temperature response functions for models of Rubisco-limited photosynthesis. *Plant, Cell & Environment, 24*, 253–259.

Bernhofer, C., Gruenwald, J., & ICOS Ecosystem Thematic Centre. (2022). *Warm winter 2020 ecosystem eddy covariance flux product from Oberbärenburg.* Retrieved March 2024, from https://doi.org/10.18160/05Y0-QP67

Blonder, B. W., Aparecido, L. M., Hultine, K. R., Lombardozzi, D., Michaletz, S. T., Posch, B. C., . . . Winter, K. (2023). Plant water use theory should incorporate hypotheses about extreme environments, population ecology, and community ecology. *New Phytologist, 238*(2), 2271-2283.

Borstlap, A. C., & Schuurmans, J. A. (2004). Sucrose transport into plasma membrane vesicles from tobacco leaves by H+ symport or counter exchange does not display a linear component. *The Journal of membrane biology, 198*, 31-42.

Boyer, J. S. (1967). Leaf water potential measure with a pressure chamber. *Plant Physiology, 42*, 133–137.

Buchmann, N., Hörtnagl, L., Merbold, L., Gharun, M., & ICOS Ecosystem Thematic Centre. (2022). *Warm winter 2020 ecosystem eddy covariance flux product from Davos.* Retrieved March 2024, from https://doi.org/10.18160/1JA9-VJEV

Buckley, T. N. (2023). Is carbon, not water, the resource that limits stomatal opening? *New Phytologist, 238*(2), 457-460.

Buckley, T. N., Miller, J. M., & Farquhar, G. D. (2002). The mathematics of linked optimisation for water and nitrogen use in a canopy. *Silva Fennica, 36*(3), 639-669.

Buckley, T. N., Sack, L., & Farquhar, G. D. (2017). Optimal plant water economy. *Plant, Cell & Environment, 40*(6), 881-896.

Buras, A., Rammig, A., & Zang, C. S. (2020). Quantifying impacts of the 2018 drought on European ecosystems in comparison to 2003. *Biogeosciences, 17*(6), 1655-1672.

Campbell, G. S., & Norman, J. N. (1998). *An Introduction to Environmental Biophysics 2nd edn.* New York: Springer.

Campbell, G. S., Papendick, R. I., Rabie, E., & Shayo-Ngowi, A. J. (1979). A Comparison of Osmotic Potential, Elastic Modulus, and Apoplastic Water in Leaves of Dryland Winter Wheat 1. *Agronomy Journal, 71*(1), 31-36.

Cochard, H., Martin, R., Gross, P., & Bogeat-Triboulot, M. B. (2000). Temperature effects on hydraulic conductance and water relations of Quercus robur L. . *Journal of Experimental Botany, 51*(348), 1255-1259.

Collatz, G. J., Ball, J. T., Grivet, C., & Berry, J. A. (1991). Physiological and environmental regulation of stomatal conductance, photosynthesis and transpiration: a model that includes a laminar boundary layer. *Agricultural and Forest meteorology, 54*, 107-136.

Cortes, P. M., & Sinclair, T. R. (1985). Extraction of Apoplastic Water during Pressure-Volume Dehydrations. *Agronomy Journal, 77*(5), 798-802.

Coursolle, C., Otis Prud'homme, G., Lamothe, M., & Isabel, N. (2019). Measuring rapid A–Ci curves in boreal conifers: black spruce and balsam fir. *Frontiers in plant science, 10*, 1276.

Cowan, I. (1982). Regulation of water use in relation to carbon gain in higher plants. In *Physiological plant ecology II* (pp. 589-613). Berlin, Heidelberg: Springer.

Cowan, I. (1986). Economics of carbon fixation in higher plants. In *On the economy of plant form and function* (pp. 133-170). Cambridge: Cambridge University Press.

Cowan, I., & Farquhar, G. (1977). Stomatal function in relation to leaf metabolism. *Symposium of the Society for Experimental Biology, 471–505*, 31.

Daudet, F. A., Lacointe, A., Gaudillere, J. P., & Cruiziat, P. (2002). Generalized Münch coupling between sugar and water fluxes for modelling carbon allocation as affected by water status. *Journal of Theoretical Biology, 214*(3), 481-498.

Davidson, K. J., Lamour, J., McPherran, A., Rogers, A., & Serbin, S. P. (2023). Seasonal trends in leaf-level photosynthetic capacity and water use efficiency in a North American Eastern deciduous forest and their impact on canopy-scale gas exchange. *New Phytologist, 240*(1), 138-156.

De Kauwe, M. G., Lin, Y. S., Wright, I. J., Medlyn, B. E., Crous, K. Y., Ellsworth, D. S., . . . Togashi, H. F. (2016). A test of the 'one-point method'for estimating maximum carboxylation capacity from field-measured, light-saturated photosynthesis. *New Phytologist, 210*(3), 1130-1144.

De Schepper, V., & Steppe, K. (2010). Development and verification of a water and sugar transport model using measured stem diameter variations. *Journal of experimental botany, 61*(8), 2083-2099.

Deans, R. M., Brodribb, T. J., Busch, F. A., & Farquhar, G. D. (2020). Optimization can provide the fundamental link between leaf photosynthesis, gas exchange and water relations. *Nature Plants, 6*(9), 1116-1125.

Dewar, R., Hölttä, T., & Salmon, Y. (2022). Exploring optimal stomatal control under alternative hypotheses for the regulation of plant sources and sinks. *New Phytologist, 233*(2), 639-654.

Dewar, R., Mauranen, A., Mäkelä, A., Hölttä, T., Medlyn, B., & Vesala, T. (2018). New insights into the covariation of stomatal, mesophyll and hydraulic conductances from optimization models incorporating nonstomatal limitations to photosynthesis. *New Phytologist, 217*(2), 571-585.

Duursma, R. A. (2015). Plantecophys-an R package for analysing and modelling leaf gas exchange data. *PloS one, 10*(11), e0143346.

Eller, C. B., Rowland, L., Mencuccini, M., Rosas, T., Williams, K., Harper, A., . . . Cox, P. M. (2020). Stomatal optimization based on xylem hydraulics (SOX) improves land surface model simulation of vegetation responses to climate. *New Phytologist, 226*(6), 1622-1637.

Fan, Y., Miguez-Macho, G., Jobbágy, E. G., Jackson, R. B., & Otero-Casal, C. (2017). Hydrologic regulation of plant rooting depth. *Proceedings of the National Academy of Sciences, 114*(40), 10572-10577.

Farquhar, G. D., von Caemmerer, S. V., & Berry, J. A. (1980). A biochemical model of photosynthetic CO 2 assimilation in leaves of C 3 species. *Planta, 149*, 78-90.

Feng, X., Lu, Y., Jiang, M., Katul, G., Manzoni, S., Mrad, A., & Vico, G. (2022). Instantaneous stomatal optimization results in suboptimal carbon gain due to legacy effects. *Plant, Cell & Environment, 45*(11), 3189-3204.

Flügge, U. I. (1999). Phosphate translocators in plastids. *Annual review of plant biology, 50*(1), 27-45.

Friend, A. (1995). PGEN: an integrated model of leaf photosynthesis, transpiration, and conductance. *Ecological Modelling, 77*(2-3), 233-255.

Fu, Q., Cheng, L., Guo, Y., & Turgeon, R. (2011). Phloem loading strategies and water relations in trees and herbaceous plants. *Plant Physiology, , 157*(3), 1518-1527.

Gardner, A., Jiang, M., Ellsworth, D. S., MacKenzie, A. R., Pritchard, J., Bader, M. K., . . . Medlyn, B. E. (2023). Optimal stomatal theory predicts CO2 responses of stomatal conductance in both gymnosperm and angiosperm trees. *New Phytologist, 237*(4), 1229-1241.

Gimeno, T. E., Crous, K. Y., Cooke, J., O'Grady, A. P., Ósvaldsson, A., Medlyn, B. E., & Ellsworth, D. S. (2016). Conserved stomatal behaviour under elevated CO2 and varying water availability in a mature woodland. *Functional EcologY, 30*(5), 700-709.

Hall, A. J., & Minchin, P. E. (2013). A closed-form solution for steady-state coupled phloem/xylem flow using the L ambert-W function. *Plant, Cell & Environment, 36*(23), 2150-2162.

Hari, P., Mäkelä, A., Korpilahti, E., & Holmberg, M. (1986). Optimal control of gas exchange. *Tree physiology, 2*, 169-175.

Hölttä, T., Lintunen, A., Chan, T., Mäkelä, A., & Nikinmaa, E. (2017). A steady-state stomatal model of balanced leaf gas exchange, hydraulics and maximal source–sink flux. *Tree Physiology, 7*(851-868), 37.

Hölttä, T., Mencuccini, M., & Nikinmaa, E. (2009). Linking phloem function to structure: analysis with a coupled xylem–phloem transport model. *Journal of theoretical biology, 259*(2), 325-337.

Hölttä, T., Vesala, T., Sevanto, S., Perämäki, M., & Nikinmaa, E. (2006). Modeling xylem and phloem water flows in trees according to cohesion theory and Münch hypothesis. *Trees, 20*, 67-78.

Hodson, T. O. (2022). Root mean square error (RMSE) or mean absolute error (MAE): When to use them or not. *Geoscientific Model Development Discussions*, *2022*, 1-10.

Jayalakshmy, M. S., & Philip, J. (2010). Thermophysical properties of plant leaves and their influence on the environment temperature. *International journal of Thermophysics, 31*, 2295-2304.

Jensen, K. H. (2018). Phloem physics: mechanisms, constraints, and perspectives. *Current opinion in plant biology, 43*, 96-100.

Joshi, J., Stocker, B. D., Hofhansl, F., Zhou, S., Dieckmann, U., & Prentice, I. C. (2022). Towards a unified theory of plant photosynthesis and hydraulics. *Nature Plants, 8*(11), 1304-1316.

Kalman, R. E. (1960). A New Approach to Linear Filtering and Prediction Problems. *Journal of Basic Engineering, 82*(1), 35–45.

Kattge, J., & Knorr, W. (2007). Temperature acclimation in a biochemical model of photosynthesis: a reanalysis of data from 36 species. *Plant, cell & environment, 30*(9), 1176-1190.

Katul, G. G., Palmroth, S., & Oren, R. A. (2009). Leaf stomatal responses to vapour pressure deficit under current and CO2-enriched atmosphere explained by the economics of gas exchange. *Plant, Cell & Environment, 32*(8), 968-979.

Kaufmann, M. (1968). Evaluation of the pressure chamber technique for estimating plant water potential of Forest tree species. *Forest Science, 14*, 369-374.

Kelly, G., Moshelion, M., David-Schwartz, R., Halperin, O., Wallach, R., Attia, Z., . . . Granot, D. (2013). Hexokinase mediates stomatal closure. *The Plant Journal, 75*(6), 977-988.

Kirkham, M., Gardner, W. R., & Gerloff, G. C. (1972). Regulation of cell division and cell enlargement by turgor pressure. *Plant Physiology, 49*(6), 961-962.

Lacointe, A., & Minchin, P. E. (2008). Modelling phloem and xylem transport within a complex architecture. *Functional Plant Biology, 35*(10), 772-780.

Lawson, T., Simkin, A. J., Kelly, G., & Granot, D. (2014). Mesophyll photosynthesis and guard cell metabolism impacts on stomatal behaviour. *New Phytologist, 203*(4), 1064-1081.

Lockhart, J. (1965). An analysis of irreversible plant cell elongation. *Journal of Theoretical Biology, 8*(2), 264-275.

Lu, Y., Duursma, R. A., Farrior, C. E., Medlyn, B. E., & Feng, X. (2020). Optimal stomatal drought response shaped by competition for water and hydraulic risk can explain plant trait covariation. *New Phytologist, 225*(3), 1206-1217.

Lu, Y., Duursma, R., & Medlyn, B. (2016). Optimal stomatal behaviour under stochastic rainfall. *Journal of Theoretical Biology, 394*, 160-171.

Mäkelä, A., Berninger, F., & Hari, P. (1996). Optimal control of gas exchange during drought: theoretical analysis. *Annals of Botany, 77*(5), 461-468.

Mantova, M., Herbette, S., Cochard, H., & Torres-Ruiz, J. M. (2021). Hydraulic failure and tree mortality: from correlation to causation. *Trends in Plant Science, 27*(4), 335-345.

Manzoni, S., Vico, G., Katul, G., Fay, P. A., Polley, W., Palmroth, S., & Porporato, A. (2011). Optimizing stomatal conductance for maximum carbon gain under water stress: a meta-analysis across plant functional types and climates. *Functional Ecology, 25*(3), 456-467.

Manzoni, S., Vico, G., Katul, G., Palmroth, S., Jackson, R. B., & Porporato, A. (2013b). Hydraulic limits on maximum plant transpiration and the emergence of the safety–efficiency trade-off. *New Phytologist, 198*(1), 169-178.

Manzoni, S., Vico, G., Palmroth, S., Porporato, A., & Katul, G. (2013a). Optimization of stomatal conductance for maximum carbon gain under dynamic soil moisture. *Advances in Water Resources, 62*, 90-105.

Marchin, R. M., Medlyn, B. E., Tjoelker, M. G., & Ellsworth, D. S. (2023). Decoupling between stomatal conductance and photosynthesis occurs under extreme heat in broadleaf tree species regardless of water access. *Global Change Biology, 29*(22), 6319-6335.

Martinez-Vilalta, J., Anderegg, W. R., Sapes, G., & Sala, A. (2019). Greater focus on water pools may improve our ability to understand and anticipate drought-induced mortality in plants. *New Phytologist, 223*(1), 22-32.

Mathias, J. M., & Thomas, R. B. (2021). Global tree intrinsic water use efficiency is enhanced by increased atmospheric CO2 and modulated by climate and plant functional types. *Proceedings of the National Academy of Sciences, 118*(7), e2014286118.

Matzner, S., & Comstock, J. (2001). The temperature dependence of shoot hydraulic resistance: implications for stomatal behaviour and hydraulic limitation. *Plant, Cell & Environment, 24*(12), 1299-1307.

Medlyn, B. E., Dreyer, E., Ellsworth, D., Forstreuter, M., Harley, P. C., Kirschbaum, M. U., . . . Loustau, D. (2002). Temperature response of parameters of a biochemically based model of photosynthesis. II. A review of experimental data. *Plant, Cell & Environment, 25*(9), 1167-1179.

Medlyn, B. E., Duursma, R. A., Eamus, D., Ellsworth, D. S., Prentice, I. C., Barton, C. V., . . . Wingate, L. (2011). Reconciling the optimal and empirical approaches to modelling stomatal conductance. *Global Change Biology, 17*(6), 2134-2144.

Mrad, A., Sevanto, S., Domec, J. C., Liu, Y., Nakad, M., & Katul, G. (2019). A dynamic optimality principle for water use strategies explains isohydric to anisohydric plant responses to drought. *Frontiers in Forests and Global Change, 2*.

Nakad, M., Sevanto, S., Domec, J. C., & Katul, G. (2023). Linking the Water and Carbon Economies of Plants in a Drying and Warming Climate. *Current Forestry Reports, 9*(6), 383-400.

Niinemets, Ü., & Kull, O. (1995). Effects of light availability and tree size on the architecture of assimilative surface in the canopy of Picea abies: variation in needle morphology. *Tree physiology, 15*(5), 307-315.

Oren, R., Sperry, J. S., Katul, G. G., Pataki, D. E., Ewers, B. E., Phillips, N., & Schäfer, K. V. (1999). Survey and synthesis of intra-and interspecific variation in stomatal sensitivity to vapour pressure deficit. *Plant, cell & environment, 22*(12), 1515-1526.

Pantin, F., Simonneau, T., & Muller, B. (2012). Coming of leaf age: control of growth by hydraulics and metabolics during leaf ontogeny. *New Phytologist, 196*(2), 349-366.

Peters, R. L., Kaewmano, A., Fu, P. L., Fan, Z. X., Sterck, F., Steppe, K., & Zuidema, P. A. (2023b). High vapour pressure deficit enhances turgor limitation of stem growth in an Asian tropical rainforest tree. *Plant, Cell & Environment, 46*(9), 2747-2762.

Peters, R. L., Speich, M., Pappas, C., Kahmen, A., von Arx, G., Pannatier, E. G., . . . Fonti, P. (2019). Contrasting stomatal sensitivity to temperature and soil drought in mature alpine conifers. *Plant, Cell & Environment, 42*(5), 1674-1689.

Peters, R. L., Steppe, K., Pappas, C., Zweifel, R., Babst, F., Dietrich, L., . . . P., F. (2023a). Daytime stomatal regulation in mature temperate trees prioritizes stem rehydration at night. *New Phytologist, 239*, 533–546.

Peters, R., Steppe, K., Cuny, H., De Pauw, D., Frank, D., Schaub, M., . . . Fonti, P. (2021). Turgor–a limiting factor for radial growth in mature conifers along an elevational gradient. *New Phytologist, 229*(1), 213-229.

Peters, W., Bastos, A., Ciais, P., & Vermeulen, A. (2020). A historical, geographical and ecological perspective on the 2018 European summer drought. *Philosophical transactions of the royal society B, 1810*(1810), 20190505.

Philip, J. R., & De Vries, D. D. (1957). Moisture movement in porous materials under temperature gradients. *Eos, Transactions American Geophysical Union, 38*(2), 222-232.

Potkay, A., & Feng, X. (2023a). Do stomata optimize turgor-driven growth?: A new framework for integrating stomata response with whole-plant hydraulics and carbon balance. *New Phytologist*. doi: https://doi.org/10.1111/nph.18620

Potkay, A., & Feng, X. (2023b). Dynamically optimizing stomatal conductance for maximum turgor-driven growth over diel and seasonal cycles. *AoB PLANTS*.

Potkay, A., Trugman, A. T., Wang, Y., Venturas, M. D., Anderegg, W. R., Mattos, C. R., & Fan, Y. (2021). Coupled whole-tree optimality and xylem hydraulics explain dynamic biomass partitioning. *New Phytologist, 230*(6), 2226-2245.

Pu, J., Roy, S., Knyazikhin, Y., & Myneni, R. (2023b). *Sensor-Independent LAI/FPAR CDR [Data set]. Zenodo.* Retrieved 3 21, 2024, from https://doi.org/10.5281/zenodo.8076540

Pu, J., Yan, K., Roy, S., Zhu, Z., Rautiainen, M., Knyazikhin, Y., & Myneni, R. B. (2023a). Sensor-independent LAI/FPAR CDR: reconstructing a global sensor-independent climate data record of MODIS and VIIRS LAI/FPAR from 2000 to 2022. *Earth System Science Data Discussions*, 1-29.

Rich, R. L., Stefanski, A., Montgomery, R. A., Hobbie, S. E., Kimball, B. A., & Reich, P. B. (2015). Design and performance of combined infrared canopy and belowground warming in the B4Warm ED (Boreal Forest Warming at an Ecotone in Danger) experiment. *Global Change Biology, 21*(6), 2334-2348.

Sack, L., Streeter, C. M., & Holbrook, N. M. (2004). Hydraulic analysis of water flow through leaves of sugar maple and red oak. *Plant Physiology, 134*(4), 1824-1833.

Sapes, G., & Sala, A. (2021). Relative water content consistently predicts drought mortality risk in seedling populations with different morphology, physiology and times to death. *Plant, Cell & Environment, 44*(10), 3322-3335.

Sapes, G., & Sala, A. (2021). Relative water content consistently predicts drought mortality risk in seedling populations with different morphology, physiology and times to death. *Plant, Cell & Environment, 44*(10), 3322-3335.

Sapes, G., Roskilly, B., Dobrowski, S., Maneta, M., Anderegg, W. R., Martinez-Vilalta, J., & Sala, A. (2019). Plant water content integrates hydraulics and carbon depletion to predict drought-induced seedling mortality. *Tree Physiology, 39*(8), 1300-1312.

Schuldt, B., Buras, A., Arend, M., Vitasse, Y., Beierkuhnlein, C., Damm, A., . . . Kahmen, A. (2020). A first assessment of the impact of the extreme 2018 summer drought on Central European forests. *Basic and Applied Ecology, 45*, 86-103.

Schymanski, S. J., Or, D., & Zwieniecki, M. (2013). Stomatal control and leaf thermal and hydraulic capacitances under rapid environmental fluctuations. *PloS one, 8*(1), e54231.

Smith, N. E., Kooijmans, L. M., Koren, G., Van Schaik, E., van Der Woude, A. M., Wanders, N., . . . Peters, W. (2020). Spring enhancement and summer reduction in carbon uptake during the 2018 drought in northwestern Europe. *philosophical Transactions of the Royal Society B, 375*(1810), 20190509.

Sperry, J. S., Adler, F. R., Campbell, G. S., & Comstock, J. P. (1998). Limitation of plant water use by rhizosphere and xylem conductance: results from a model. *Plant, Cell & Environment, 21*(4), 347-359.

Sperry, J. S., Venturas, M. D., Anderegg, W. R., Mencuccini, M., Mackay, D. S., Wang, Y., & Love, D. M. (2017). Predicting stomatal responses to the environment from the optimization of photosynthetic gain and hydraulic cost. *Plant, cell & environment, 40*(6), 816-830.

Stefanski, A., Butler, E. E., Bermudez, R., Montgomery, R. A., & Reich, P. B. (2023). Stomatal behaviour moderates the water cost of CO2 acquisition for 21 boreal and temperate species under experimental climate change. *Plant, Cell & Environment, 46*(10), 3102-3119.

Steppe, K., De Pauw, D. J., Lemeur, R., & Vanrolleghem, P. A. (2006). A mathematical model linking tree sap flow dynamics to daily stem diameter fluctuations and radial stem growth. *Tree physiology, 26*(3), 257-273.

Steudle, E., Zimmermann, U., & Lüttge, U. (1977). Effect of turgor pressure and cell size on the wall elasticity of plant cells. *Plant Physiology, 59*(2), 285-289.

Teepe, R., Dilling, H., & Beese, F. (2003). Estimating water retention curves of forest soils from soil texture and bulk density. *Journal of Plant Nutrition and Soil Science, 166*(1), 111-119.

Thompson, M. V., & Holbrook, N. M. (2003). Application of a single-solute non-steady-state phloem model to the study of long-distance assimilate transport. *Journal of Theoretical Biology, 220*(4), 419-455.

Tuzet, A., Perrier, A., & Leuning, R. (2003). A coupled model of stomatal conductance, photosynthesis and transpiration. *Plant, Cell & Environment, 26*(7), 1097-1116.

Tyree, M., & Hammel, H. (1972). The measurement of the turgor pressure and the water relations of plants by the pressure-bomb technique. *Journal of Experimental Botany, 23*(1), 267-282.

Urban, L., Jaffrin, A., & Chraibi, A. (1993). Analysis of pressure-volume curves of leaves of Rosa hybrida cv. Sonia. *Journal of Experimental Botany, 44*(3), 605-613.

Van Genuchten, M. T. (1980). A closed-form equation for predicting the hydraulic conductivity of unsaturated soils. *Soil science society of America journal, 44*(5), 892-898.

Venturas, M. D., Sperry, J. S., Love, D. M., Frehner, E. H., Allred, M. G., Wang, Y., & Anderegg, W. R. (2018). A stomatal control model based on optimization of carbon gain versus hydraulic risk predicts aspen sapling responses to drought. *New Phytologist, 220*(3), 836-850.

Vicente-Serrano, S. M., Beguería, S., & López-Moreno, J. I. (2010). A multiscalar drought index sensitive to global warming: the standardized precipitation evapotranspiration index. *Journal of climate, 23*(7), 1696-1718.

Vos, J., & Oyarzun, P. J. (1988). Water Relations of Potato Leaves. II. Pressure—Volume Analysis and Inferences About the Constancy of the Apoplastic Fraction. *Annals of Botany, 62*(5), 449-454.

Vrugt, J. A. (2016). Markov chain Monte Carlo simulation using the DREAM software package: Theory, concepts, and MATLAB implementation. *Environmental Modelling & Software, 75*, 273-316.

Vrugt, J. A., ter Braak, C. J., Diks, C. G., & Schoups, G. (2013). Hydrologic data assimilation using particle Markov chain Monte Carlo simulation: Theory, concepts and applications. *Advances in Water Resources, 51*, 457-478.

Wang, Y., Anderegg, W. R., Venturas, M. D., Trugman, A. T., Yu, K., & Frankenberg, C. (2021). Optimization theory explains nighttime stomatal responses. *New Phytologist, 230*(3), 1550-1561.

Wang, Y., Sperry, J. S., Anderegg, W. R., Venturas, M. D., & Trugman, A. T. (2020). A theoretical and empirical assessment of stomatal optimization modeling. *New Phytologist, 227*(2), 311-325.

Witelski, T., & Bowen, M. (2015). Variational principles. In *Methods of Mathematical Modelling* (pp. 47-83). Cham Heidelberg New York Dordrecht London: Springer.

WMO. (2008). *Guide to meteorological instruments and methods of observation, appendix 4B, WMO-No. 8 (CIMO guide).* Geneva, Switzerland: World Meteorological Organization.

Wolf, A., Anderegg, W. R., & Pacala, S. W. (2016). Optimal stomatal behavior with competition for water and risk of hydraulic impairment. *Proceedings of the National Academy of Sciences, 113*(46), E7222-E7230.

Wolfe, B. T., Detto, M., Zhang, Y. J., Anderson-Teixeira, K. J., Brodribb, T., Collins, A. D., . . . Wright, S. (2023). Leaves as bottlenecks: The contribution of tree leaves to hydraulic resistance within the soil-plant-atmosphere continuum. *Plant, Cell & Environment, 46*(3), 736-746.

Zhang, J., Vrugt, J. A., Shi, X., Lin, G., Wu, L., & Zeng, L. (2020). Improving simulation efficiency of MCMC for inverse modeling of hydrologic systems with a Kalman-inspired proposal distribution. *Water Resources Research, 56*(3), e2019WR025474.

Zhou, S., Duursma, R. A., Medlyn, B. E., Kelly, J. W., & Prentice, I. C. (2013). How should we model plant responses to drought? An analysis of stomatal and non-stomatal responses to water stress. *Agricultural and Forest Meteorology, 182*, 204-214.
